# A computational analysis of dynamic, multi-organ inflammatory crosstalk induced by endotoxin in mice

**DOI:** 10.1371/journal.pcbi.1006582

**Published:** 2018-11-06

**Authors:** Ruben Zamora, Sebastian Korff, Qi Mi, Derek Barclay, Lukas Schimunek, Riccardo Zucca, Xerxes D. Arsiwalla, Richard L. Simmons, Paul Verschure, Timothy R. Billiar, Yoram Vodovotz

**Affiliations:** 1 Department of Surgery, University of Pittsburgh, Pittsburgh, Pennsylvania, United States of America; 2 Center for Inflammation and Regenerative Modeling, McGowan Institute for Regenerative Medicine, University of Pittsburgh, Pittsburgh, Pennsylvania, United States of America; 3 Pittsburgh Liver Research Center, University of Pittsburgh, Pittsburgh, Pennsylvania, United States of America; 4 Department of Orthopedics and Traumatology, Universität Heidelberg, Heidelberg, Germany; 5 Department of Sports Medicine and Nutrition, University of Pittsburgh, Pittsburgh, Pennsylvania, United States of America; 6 SPECS, ETIC, Universitat Pompeu Fabra, Barcelona, Spain; 7 IBEC, BIST, Barcelona, Spain; 8 ICREA, Barcelona, Spain; Rutgers University, UNITED STATES

## Abstract

Bacterial lipopolysaccharide (LPS) induces an acute inflammatory response across multiple organs, primarily via Toll-like receptor 4 (TLR4). We sought to define novel aspects of the complex spatiotemporal dynamics of LPS-induced inflammation using computational modeling, with a special focus on the timing of pathological systemic spillover. An analysis of principal drivers of LPS-induced inflammation in the heart, gut, lung, liver, spleen, and kidney to assess organ-specific dynamics, as well as in the plasma (as an assessment of systemic spillover), was carried out using data on 20 protein-level inflammatory mediators measured over 0-48h in both C57BL/6 and TLR4-null mice. Using a suite of computational techniques, including a time-interval variant of Principal Component Analysis, we confirm key roles for cytokines such as tumor necrosis factor-α and interleukin-17A, define a temporal hierarchy of organ-localized inflammation, and infer the point at which organ-localized inflammation spills over systemically. Thus, by employing a systems biology approach, we obtain a novel perspective on the time- and organ-specific components in the propagation of acute systemic inflammation.

## Author summary

Gram-negative bacterial lipopolysaccharide (LPS) is both a central mediator of sepsis and a canonical inducer of acute inflammation via Toll-like receptor 4 (TLR4). Sepsis involves the systemic spillover of inflammation that normally remains localized in individual organs. The goal of this study was to gain insights into 1) early vs. later drivers of LPS-induced inflammation in various compartments, and 2) the systemic spillover from affected organs vs. local production of inflammatory mediators in the blood. This study involved a large number of data points on the dynamics of inflammatory mediators at the protein level, data-driven computational modeling of principal characteristics and cross-correlations, and validation of key hypotheses. In addition to verifying key mechanisms in LPS/TLR4-driven acute inflammation, this approach yielded key insights into the progression of inflammation across tissues, and also suggested the presence of TLR4-independent pathways (especially in the gut). This is, to our knowledge, the first study examining the dynamic evolution of some key inflammatory mediators and their interactions with each other in both the systemic circulation and within a number of targeted parenchymal organs in mice.

## Introduction

Bacterial sepsis is a complex process in which a rapidly-evolving systemic and uncontrolled immune activation is initiated and perpetuated by microbial invasion [1]. This inflammatory response can lead to a shock state and severe organ dysfunction, at times culminating in death. Despite intense preclinical and clinical research over the past several decades, sepsis remains the most common cause of death in intensive care units (ICU), with increasing incidence and high mortality rates worldwide [2, 3]. There are currently no approved therapies for sepsis-induced inflammation [4]. Thus, a better understanding of the complex pathogenesis of bacterially induced acute inflammation is needed in order to develop novel sepsis treatments.

Endotoxin, the lipopolysaccharide (LPS) on the outer membrane of Gram negative bacteria, has been long recognized as a potent microbial mediator in the pathogenesis of systemic inflammation in gram negative sepsis and septic shock [5, 6]. Although the acute administration of LPS into rodents does not fully mimic the more gradual evolving inflammatory dynamics of a replicating and disseminating bacterial infection in humans, it does serve as a means of inducing a quantifiable systemic acute inflammation that shares many of the hallmarks of bacterial sepsis [7].

Lipopolysaccharide interacts with a number of host soluble and cell-surface molecules, including complement, LPS binding protein (LBP), CD14, MD-2, and Toll-like Receptor 4 (TLR4). Extracellular LPS binds specifically to the cell surface TLR4/MD2 receptor complex, followed by initiation of the MyD88 and TRIF signaling pathways, ultimately leading to an explosive cascade of a large number of inflammatory mediators produced by multiple organs and detected in the systemic circulation, which is commonly termed a “cytokine storm.” These inflammatory mediators can together lead to host toxic shock and sepsis [6].

Despite the extensive molecular-, cellular-, and genetic-based studies in inflammation, there is limited understanding of the multi-organ and systemic dynamic changes and their interactions in acute inflammation *in vivo*. Systems biology approaches such as data-driven and mechanistic mathematical models have been used as valuable tools to gain insight into the dynamic responses in inflammation [8, 9]. While mathematical modeling of acute inflammation and sepsis has a decades-long history [10, 11], the use of mechanistic (equation- or agent-based) computational modeling to yield insights into the systemic, acute inflammatory response induced by LPS is more recent [12–17]. Parallel studies have employed data-driven modeling to define dynamic molecular networks in the setting of both experimental and clinical trauma, a condition that, like sepsis and endotoxemia, induces acute systemic inflammation [18–23].

In the present study, we sought to define, in both space [multi-organ] and time [over 48 h], the evolution of systemic, acute inflammation initiated by the interaction of LPS with TLR4 cell surface receptors. There are, of course, numerous reports confirming the production of individual molecular inflammatory mediators as a result of LPS/TLR4 interactions in mouse models of LPS-induced systemic inflammation. There is, however, no previous study examining the dynamic evolution of the key inflammatory mediators and the interactions among them. Furthermore, there are no studies of the molecular networks emerging from LPS/TLR4 interaction which take place simultaneously, or sequentially, within the various parenchymal organs, and that may be reflected in the plasma. We hypothesized that insights into those key interactions and the role of TLR4 could be gleaned from data-driven methods such as Principal Component Analysis (PCA) [24, 25]. We also sought to define the specific role of LPS/TLR4 interactions on these dynamic changes in inflammation, by comparing inflammatory mediator responses in control C57BL/6 mice with those in mice lacking TLR4. In this way, we aimed to distinguish between TLR4-dependent and TLR4–independent dynamic programs of systemic and organ specific inflammation induced by endotoxemia.

## Results

### Endotoxemia results in dynamic systemic inflammatory changes in mice

**Fig 1:**
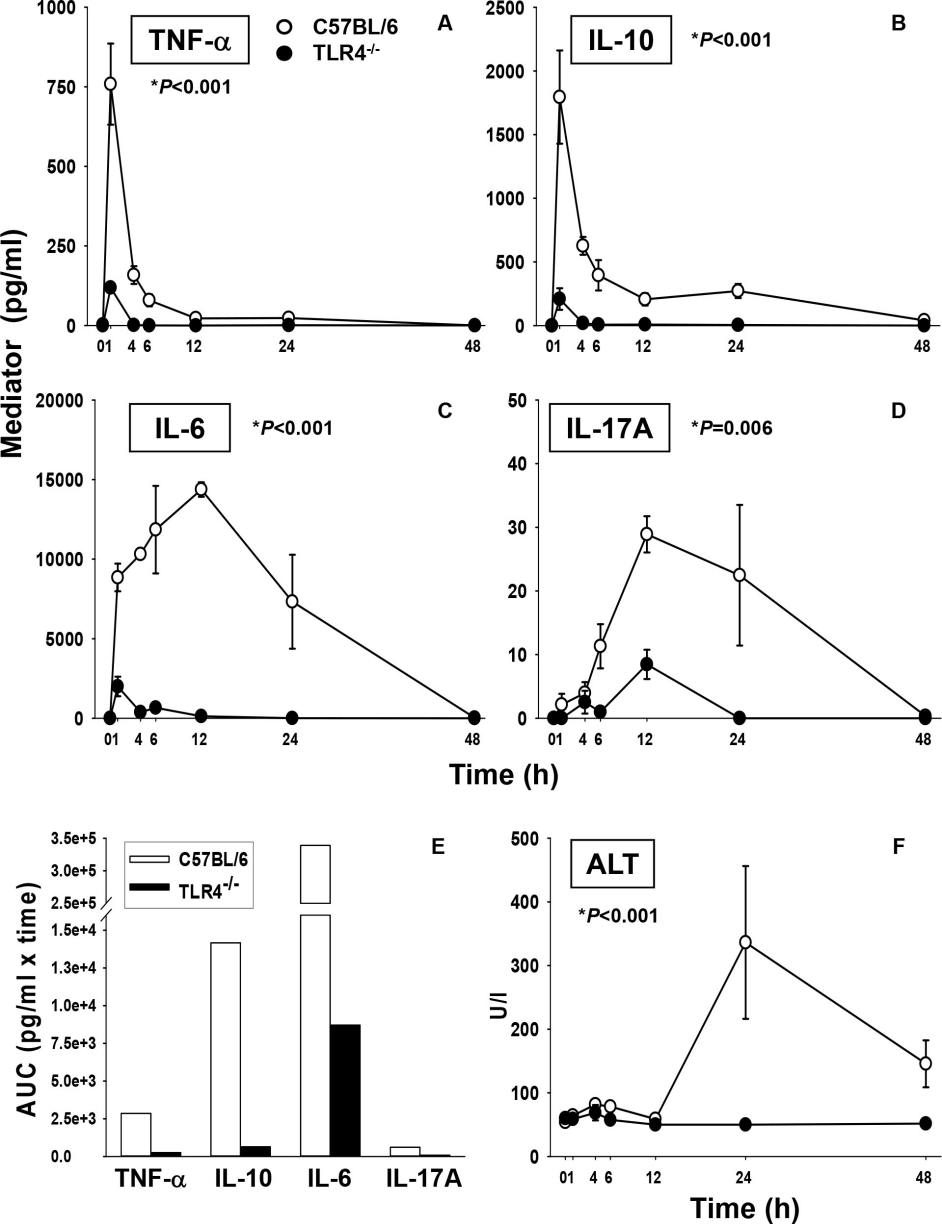
Blunted response to LPS in TLR4^-/-^ mice. C57BL/6 and TLR4^-/-^ mice were injected with LPS (3 mg/kg, i.p.). At different time points (0, 1, 4, 6, 12, 24 and 48 h) upon sacrifice, blood was collected and inflammatory mediators were measured by Luminex as described in *Materials and Methods*. Figure shows plasma concentrations of (**A**): TNFα, (**B**): IL-10, (**C**): IL-6, (**D**): IL-17A, and their respective AUCs (**E**) in both C57BL/6 and TLR4^-/-^ mice. The significant reduction in damage to parenchymal cells is reflected in lower ALT concentrations in the TLR4^-/-^ animals (**F**). Results represent the mean ± SEM from n = 5–8 (C57BL/6) and n = 4 (TLR4^-/-^) animals for each experimental group (C57BL/6 vs. TLR4^-/-^, analyzed by Two-Way ANOVA as indicated).

In order to induce an acute inflammatory response, TLR4^+/+^ C57BL/6 (generally considered a Th1-dominant mouse strain) [26] and TLR4^-/-^ mice were challenged with an intraperitoneal bolus injection of a non-lethal dose of LPS. As expected, significantly elevated concentrations of multiple circulating mediators were observed in the plasma of C57BL/6 mice as compared to TLR4^-/-^ animals. The mediator peaks in C57BL/6 mice were not only higher, but appeared earlier, generally within 12 h, when compared with TLR4^-/-^ mice. As representative examples, we show the changes in plasma TNFα, IL-10, IL-6, and IL-17A in Fig 1A to 1E. All measured mediators are depicted in S1 Fig (see also S1 Luminex Data). The significant reduction in systemic inflammation in TLR4^-/-^ mice was associated with lower circulating concentrations of ALT (Fig 1F), reflective of a lesser degree of organ (predominantly liver) damage in TLR^-/-^ mice.

### LPS-induced inflammation exhibits differential spatiotemporal dynamics in C57BL/6 vs. TLR4^-/-^ mice

Markedly elevated circulating concentrations of inflammatory mediators are a key hallmark of sepsis and endotoxemia. Inflammatory mediators can appear in the blood either due to production by circulating inflammatory cells or due to systemic spillover from inflamed organs and tissues. Accordingly, we set out to define the spatiotemporal sequence of LPS-induced inflammation, and the role of TLR4 therein, by examining the concentrations of mediators across time in multiple organs in both C57BL/6 and TLR4^-/-^ mice. The individual time-courses of inflammatory mediators in the circulation (plasma), as well as in various organs (liver, heart, terminal ileum, lung, spleen and kidney), along with the corresponding *P* values for comparison of the changes in C57BL/6 vs. TLR4^-/-^ mice by Two-Way ANOVA can be found in S1 Fig. This analysis demonstrated that most mediators were present at significantly higher concentrations in nearly all organs, across nearly all time-points, in mice expressing TLR4 (C57BL/6; Fig 1 and S1 Fig). In some organs of TLR4^-/-^ mice, however, a few mediators transiently exceeded levels than those found in C57BL/6 mice at a comparable time point (e.g. GM-CSF and IL-10 in the kidney, IL-1β in the lung and kidney, VEGF in the gut and kidney, and IL-13 in the lung and spleen; S1 Fig). Those few inflammatory mediators expressed transiently and at modestly higher concentrations in TLR4^-/-^ mice most often reached these levels at 24h or later following LPS challenge.

### LPS/TLR4-induced, organ-specific, dominant mediators over time inferred from Time-Interval Principal Component Analysis (TI-PCA)

We next sought to define how the global response to LPS/TLR4-driven inflammation is coordinated among organs over time, and how this process was reflected in the systemic circulation. Principal Component Analysis (PCA) and related tools, such as Partial Least Squares Decomposition, have been used by multiple groups to define the core characteristics of a multivariate, time-varying biological response [18, 24, 25, 27–29], and in particular, multiway variants of supervised principal components have been reported for time courses of inflammatory signals [30]. First, we performed standard PCA over the whole time-period (0-48h) (S2 Fig). This analysis suggested that, in general, the systemic response predominates in C57Bl/6 mice (7 of the top 10 principal mediators are in the plasma) but not TLR4-deficient animals (3 out of the top 10 mediators are in the plasma), supporting a well-established concept that systemic spillover of inflammatory mediators is a key hallmark of sepsis and endotoxemia. However, this analysis did not reveal the spatiotemporal sequence of organ-localized inflammation associated with this systemic spillover.

**Fig 2:**
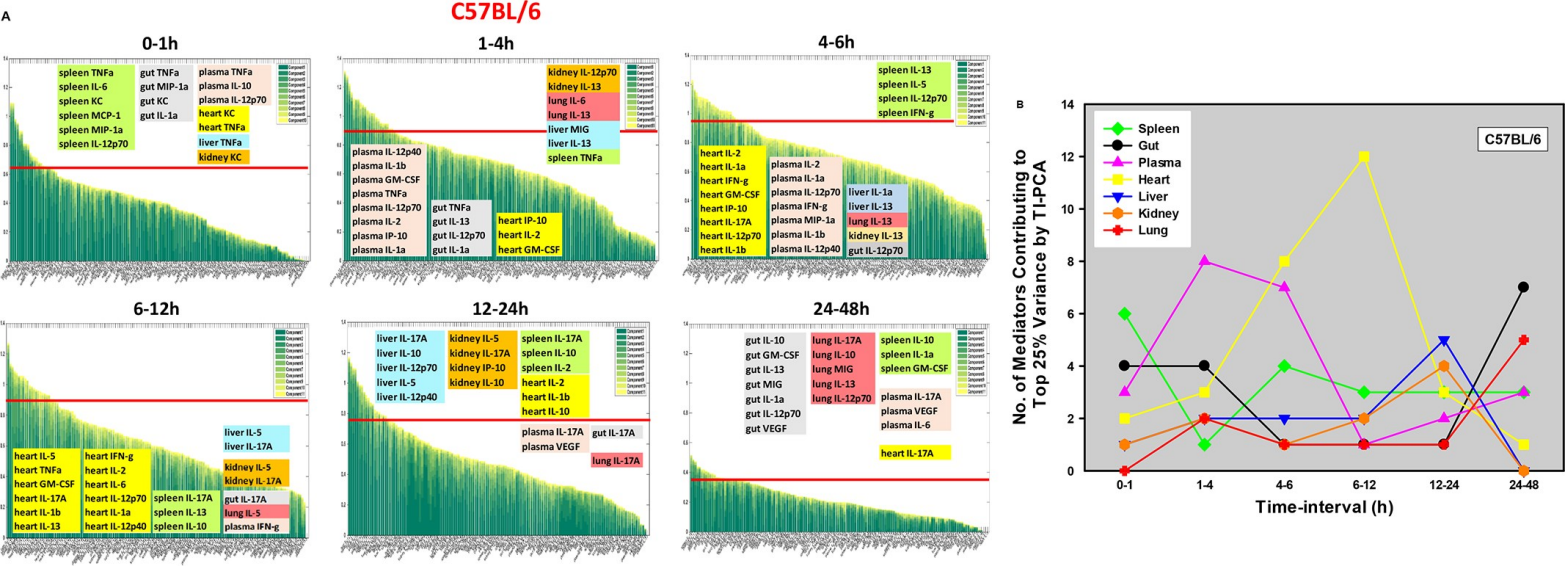
Analysis of endotoxemic C57BL/6 mice by Time-Interval PCA (TI-PCA). Animals (n = 5–8 for each experimental group) were injected with LPS (3 mg/kg, i.p.). At different time points upon sacrifice, the inflammatory mediators in blood and different organs (liver, heart, gut, lung, spleen and kidney) were measured by Luminex as described in *Materials and Methods*. Identification of the inflammatory mediators contributing to the top 25% variance of the inflammatory response (shown above the red line in the PCA graph) in all organs together during each of the following six time frames: 0-1h, 1-4h, 4-6h, 6-12h, 12-24h, and 24-48h (**A**) was performed using Time-Interval PCA as described in *Materials and Methods*. The number of mediators contributing to the top 25% variance of the inflammatory response in each organ as well as in plasma during each time-interval is represented in Panel **B**.

**Fig 3:**
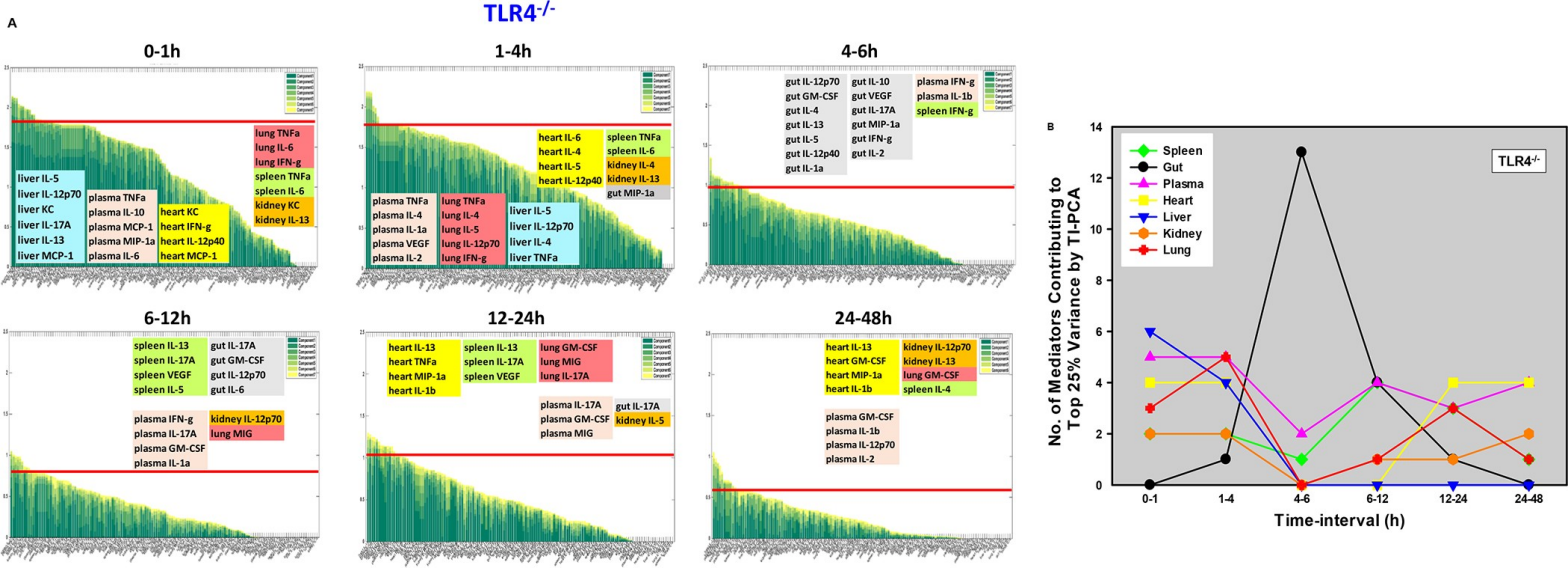
Analysis of endotoxemic TLR4^-/-^ mice by Time-Interval PCA (TI-PCA). Animals (n = 4 for each experimental group) were injected with LPS (3 mg/kg, i.p.). At different time points upon sacrifice, the inflammatory mediators in blood and different organs were measured by Luminex, and identification (**A**) as well as quantification (**B**) of the inflammatory mediators contributing to the top 25% variance of the inflammatory response was performed as described in **Fig 2** legend.

To better define the dynamic inflammatory response to LPS and the role that TLR4 plays in this inflammatory coordination, i.e. when inflammation peaks in any given organ, and when it shifts from being local to being systemic, we sought to utilize PCA in a more granular fashion across distinct time intervals in tissue samples from C57BL/6 and TLR4^-/-^ mice. We utilized a more granular method (Time-Interval PCA; TI-PCA) to identify those inflammatory mediators that contributed the most to the overall variance of the inflammatory response in tissues from both mouse strains over six distinct, consecutive time-intervals (0-1h, 1-4h, 4-6h, 6-12h, 12-24h, and 24-48h). We therefore compared C57BL/6 and TLR4^-/-^ mice with respect to the inflammatory mediators contributing to the top 25% of variance in each organ as well as in plasma within a given time-interval. TI-PCA revealed distinct inflammatory patterns in all organs, and clear differences between C57BL/6 (Fig 2) and TLR4^-/-^ (Fig 3) mice both in the organs studied and principal mediators. TNF-α was the dominant mediator in the spleen, gut, plasma, heart, and liver of C57BL/6 mice during the initial [0-1h] time frame (Fig 2A). By contrast, in TLR4^-/-^ mice, TNF-α was the dominant initial cytokine only in plasma, lung and spleen, and IL-5 was the largest contributor in the liver (Fig 2B). Analysis of the intermediate time-intervals [4-12h] suggested a dominant role for the heart in C57BL/6 mice (Fig 2A and 2B) vs. the gut in TLR4^-/-^ mice (Fig 3A and 3B), while at 12-24h liver and kidney mediators were predominant in C57BL/6 mice (Fig 2A) vs. multiple mediators in the heart, spleen, lung, and plasma in TLR4^-/-^ mice (Fig 3A). Analysis of the final time-interval [24-48h] showed the opposite pattern, with a dominant role for multiple mediators in the gut in C57BL/6 mice (Fig 2A and 2B) vs. the heart in TLR4^-/-^ mice (Fig 3A and 3B).

**Fig 4:**
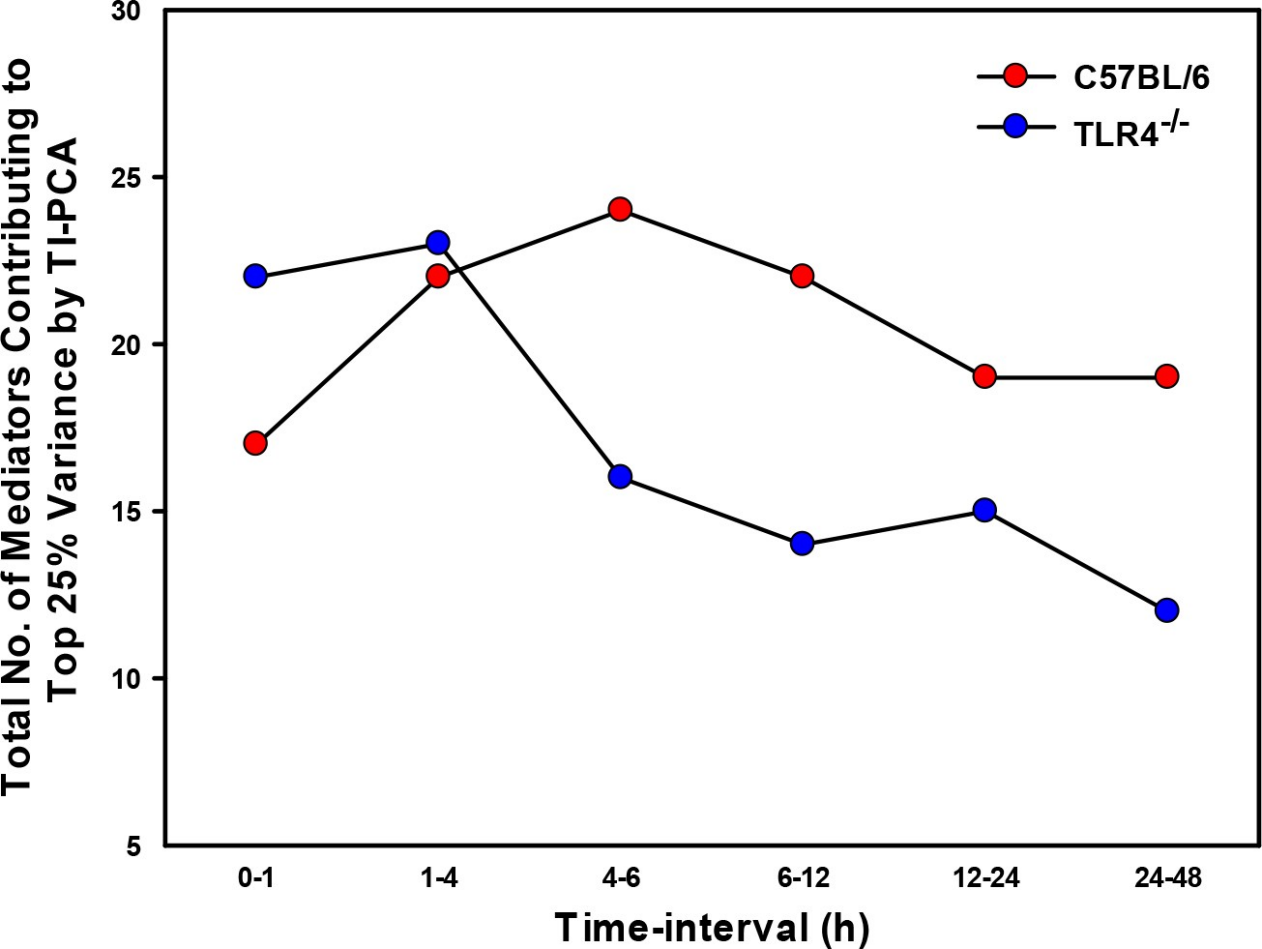
Total number of mediators contributing to the top 25% variance by Time-Interval PCA (TI-PCA) in C57BL/6 vs. TLR4^-/-^ mice. Animals (C57BL/6, n = 5–8 and TLR4^-/-^, n = 4 for each experimental group) were injected with LPS (3 mg/kg, i.p.). At different time points upon sacrifice, the inflammatory mediators in blood and different organs (liver, heart, gut, lung, spleen and kidney) were measured by Luminex and Time-Interval PCA was performed as described in *Materials and Methods*. Figure shows the total number of mediators contributing to the top 25% variance of the inflammatory response in each organ as well as in plasma of C57BL/6 vs. TLR4^-/-^ mice during the indicated time-intervals.

The total number of mediators contributing to the top 25% of variance was generally higher in C57BL/6 vs. TLR4^-/-^ mice [1-48h] (Fig 4), except in the initial time-interval [0-1h], raising the possibility of non-TLR4 LPS signaling pathways in the early response to LPS in some organs. In C57BL/6 mice, the heart was the organ with the greatest number of inflammatory mediators that contribute to the top 25% of the variance in C57BL/6 mice (Fig 2B) vs. the gut in TLR4^-/-^ mice (Fig 3B). Furthermore, based on analysis of peak total variance, inflammation appeared to spread across compartments in C57BL/6 mice in the following order: spleen → plasma → heart → liver → gut/lung (Fig 2A). In contrast, the sequence of inflammatory activation in TLR4^-/-^ mice appeared to be as follows: liver/plasma → lung → gut → heart (Fig 3A).

### Correlations among concentrations of organ-localized inflammatory mediators suggests differential and organ-specific inflammatory patterns in C57BL/6 vs. TLR4^-/-^ mice

**Fig 5:**
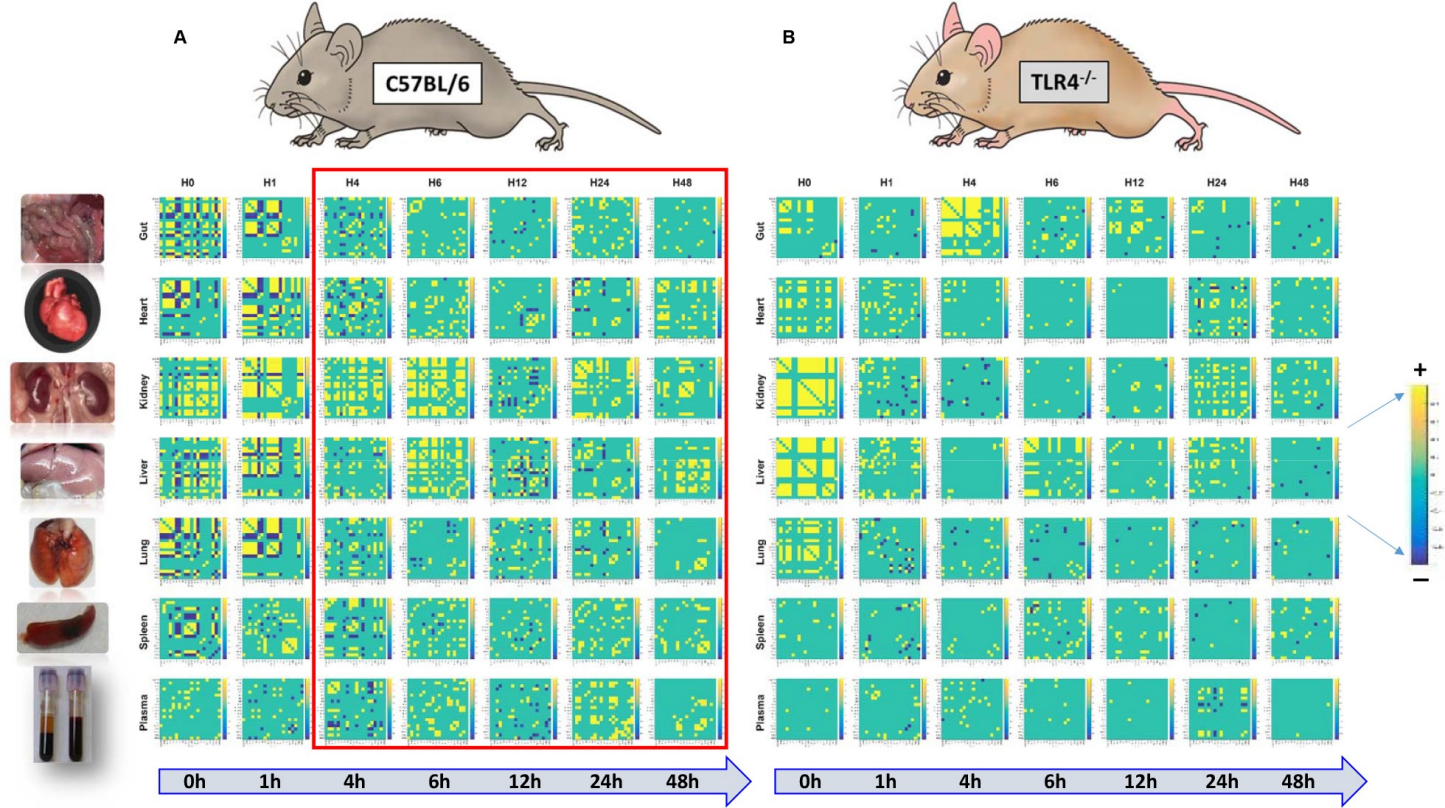
Pearson’s correlation between concentrations of inflammatory mediators in C57BL/6 and TLR4^-/-^ mice. Animals (C57BL/6, n = 5–8 and TLR4^-/-^, n = 4 for each experimental group) were injected with LPS (3 mg/kg, i.p.). At different time points upon sacrifice, the inflammatory mediators in blood and different organs (liver, heart, gut, lung, spleen and kidney) were measured by Luminex and Pearson’s correlation was calculated as described in *Materials and Methods*. Figure shows the 20x20 correlation matrix of mediators' interactions in all organs as well as in plasma for each time-point in C57BL/6 (Panel **A**) and TLR4^-/-^ (Panel **B**) mice. The time-point at which these results suggest the systemic spillover begins to occur in C57BL/6 mice is shown by a red box in Panel **A**.

We next employed a multiple correlation analysis to determine the TLR4-dependent spatiotemporal evolution of LPS-induced inflammation and to define the time interval at which inflammation spills over from individual organs to the systemic circulation (Fig 5). This analysis revealed a more homogeneous pattern of distribution of both positive (yellow squares) and negative (blue squares) correlations across all organs and time-points in C57BL/6 (Fig 5A) vs. TLR4^-/-^ (Fig 5B) mice. Interestingly, the analysis of the individual matrices at each time-point singled out the liver and kidney at 0h, and the gut at 4h, as having a higher association between inflammatory mediators in TLR4^-/-^ as compared to C57BL/6 mice (Fig 5B). Furthermore, we found that correlation matrices in the plasma of C57BL/6 mice were largely distinct from those TLR4^-/-^ mice in the early time-points (1-6h), confirming our results by TI-PCA (see Fig 2B). However, from 4h on, the correlation matrices in plasma of C57Bl/6 mice began to resemble qualitatively those of various organs (especially the liver, kidney, heart, and gut), suggesting that this is the time frame at which systemic spillover begins to occur. In contrast, systemic spillover in TLR4^-/-^ mice appeared to be less extensive than in C57BL/6 mice, with no clear pattern of similarity between plasma and other tissues (Fig 5B)

## Discussion

Our goals in the present study were to gain insights into 1) early vs. later drivers of inflammation in various compartments, and 2) the systemic spillover from affected organs vs. local production of inflammatory mediators in the blood. We carried out an iterative approach involving 10,220 data points on the dynamics of inflammatory mediators at the protein level, data-driven computational modeling of principal characteristics and cross-correlations, and validation of key hypotheses. This approach verified well-established mechanisms in LPS/TLR4-driven acute inflammation (e.g. the known early role of TNF-α and the central role of TLR4 in systemic responses to LPS). The data yielded key insights into the progression of inflammation across tissues, and the presence of TLR4-independent pathways (especially in the gut).

We focused on endotoxemia as a quantitative, reproducible model of acute inflammation, with hallmarks of both sepsis and sterile inflammation. There have been numerous prior studies of endotoxemia in multiple species, including humans, documenting tissue expression and systemic elevation of multiple inflammatory mediators. We note that it has been previously suggested that the systemic inflammatory response initiates disruption of communication and uncoupling that progresses into multiple organ dysfunction syndrome in sepsis [31]. However, few studies have examined the dynamic propagation of inflammatory networks in response to LPS [32, 33], and none have done so at a systems level using a number of inflammatory mediators measured simultaneously in both the systemic circulation and key organs. The primary goal in the present study was to gain insights into early vs. later drivers of inflammation in various compartments. We sought to unify disparate data such as those described in the prior studies mentioned above (as well as many others) into a single, unified whole. Given the complexity of this task, we needed to develop a computational technique that would point to specific mediator(s), in specific compartment(s), and at specific time ranges as being central to the multivariate, dynamic inflammatory response to LPS. We therefore developed a variant on PCA, which we define here for the first time as Time-Interval PCA (TI-PCA) carried out over specific time intervals rather than across the entire time range of a given data set. This analysis has face validity in that it pointed to TNF-α as one of the key early mediators coordinating LPS-induced inflammation in C57BL/6 mice, a reassuring finding that is concordant with decades of prior work. TI-PCA further suggested an important role for the TLR4 in the heart at intermediate time points following administration of LPS.

We also confirmed and extended many findings regarding the effects of LPS and the role of TLR4 demonstrated previously separately in various organs in the context of experimental endotoxemia in mice. Early studies more than two decades ago showed that macrophages from C3H/HeJ mice do not produce inflammatory mediators such as TNF-α and IL-1 [34], a phenomenon later shown to be due to mutations in the TLR4 gene [35]. At the systems biology level, using DNA microarray analysis of whole liver, a number of genes were identified (e.g. the chemokine KC/CXCL1) that were significantly reduced in response to LPS in TLR4^-/-^ livers as compared to C57BL/6 [36]. TLR4-mediated, complex gene expression alterations in hepatic stellate cells (HSCs), the primary fibrogenic cell type in the liver, and gene expression profiles were markedly different between HSCs from C57BL/6 and TLR4^-/-^ mice under basal conditions or following stimulation with LPS [37]. In the lung, LPS strongly stimulated the release of KC and IL-6 in TLR4^+/+^ pulmonary epithelial cells, while those mediators were absent in epithelial supernatants isolated from TLR4^-/-^ animals [38]. In the kidney, TLR4^-/-^ mice were shown to be resistant to endotoxin-induced acute renal failure, a phenomenon associated with a lack of a systemic TNF-α response [39]. Indeed, it is likely that TLRs including TLR4 are involved in many if not all types of renal inflammation [40]. However, a detailed multiplex analysis of inflammatory mediators in the gut of C57BL/6 and TLR4^-/-^ mice exposed to LPS has not been reported previously.

Our findings implicate the heart as an important target or signaling organ in the TLR4-dependent response to LPS, in agreement with prior studies. C57BL/6 mice challenged with LPS displayed reduced cardiac function, increased myocardial levels of IL-1β and TNF-α, and upregulation of mRNA encoding TLR4 prior to myocardial leukocyte infiltration. In contrast, TLR4^-/-^ mice had unaffected cardiac function and sustained significantly smaller infarctions as compared to control mice at comparable areas at risk, suggesting that cardiomyocyte TLR4 is involved in acute myocardial dysfunction following septic shock [41]. Furthermore, in a mouse model of myocardial infarction, TNF-α mRNA, IL-1α, IL-2, IL-4, IL-5, IL-6, IL-10, IL-17A, TNF-α, IFN-γ, and GM-CSF expression were all lower in the infarct area of TLR4-deficient mice compared with wild-type mice [42]. In contrast, our studies suggest a role for pathways other than TLR4 in gut inflammation. This is in agreement with prior studies showing that TLR4 does not affect the intestinal microbiota composition in mice [43], as well as studies showing a minimal effect of TLR deficiency on the composition of the intestinal microbiota under homeostatic conditions and after recovery from antibiotic treatment [44].

Taken together, these findings suggest that there is a rotation of centrality or dominance of LPS-induced inflammation from the spleen to the circulation to the heart to the liver to gut as the response evolves, a process that is clearly mediated by TLR4 as shown by the different pattern of central organ/mediators in TLR4^-/-^ mice. We are aware of the difficulty of translating these TI-PCA biologically because of inherent limitations in the computational techniques used [28]. It is also difficult to identify (and quantify) the presence and activity of specific inflammatory or parenchymal cells during specific time periods, as is the identification of potential crosstalk among dynamic inflammation programs. However, our data-driven computational analyses point unequivocally to both common and divergent processes in C57BL/6 vs. TLR4^-/-^ mice, and to a non-intuitive hypothesis regarding the role of cardiac inflammatory pathways in the multi-system propagation of LPS-induced inflammation.

Our second goal in this study was to define the role of systemic spillover from affected organs vs. local production of inflammatory mediators in the blood. Based on TI-PCA, we infer that during the early time-intervals assessed (1-6h), in C57BL/6 mice, the degree to which plasma factors dominated the overall response exceeded the degree that any other compartment predominated. This, coupled with the finding that inflammatory matrices in the plasma of C57BL/6 mice were less correlated than those in some organs such as liver and kidney, leads us to suggest that the overall character of the systemic inflammatory response to LPS might be shaped by systemic spillover from inflamed organs rather than by production of inflammatory mediators in the blood, starting after approximately 4 h after LPS challenge. Testing this hypothesis will require a more detailed analysis of data obtained from other tissues and organs.

We arrived at these conclusions through the use of a suite of complementary data-driven modeling tools which, in and of themselves, represent an overarching hypothesis about how the inflammatory response progresses. In this hypothesis, both parenchymal and inflammatory cells (resident and infiltrating) sense the presence of LPS and, in response, elaborate chemokines that form defined networks. As the presence of signals regarding the original stress (in the form of LPS), along with the development and actions of these chemokine pathways, early regulatory cytokines such as TNF-α begin to be secreted. Due to their dependence on the ongoing dynamic flow of information through chemokine networks at early time points, these mediators are present at low levels, often with high variance, and thus may be considered “insignificant” using standard statistical analyses. However, their presence and effect may be inferred using computational techniques such as PCA [45].

It is important to mention that the observation that TLR4-deficient cells lose most of the canonical responses to LPS, such as expression of pro-inflammatory cytokines, initially led to the assumption that TLR4 is the sole receptor for LPS and accounts exclusively for all of its host responses [46]. However, in 2013 it was shown that priming the caspase-11 pathway *in vivo* resulted in extreme sensitivity to subsequent LPS challenge in both C57BL/6 and TLR4-deficient mice, whereas caspase11-deficient mice were relatively resistant, revealing a new pathway for detecting cytoplasmic LPS [47]. Simultaneously, another study unveiled a similar TLR4-independent mechanism for innate immune recognition of LPS [48]. Future studies will be aimed at defining dynamic networks and principal characteristics driven by such alternative pathways.

Furthermore, although we did not study the peritoneal cavity itself directly, a previous study in Balb/c mice showed increased levels of TNF-α, IFN-γ, and IL-10 in the peritoneal lavage after LPS stimulation for 2h [49]; furthermore, a major TNF-α response has recently been associated with fat-associated lymphoid clusters after exposure to LPS [50]. Whether or not the early LPS-induced TNF-α response (or other mediators) derives from peritoneal production in our study as well as the effect of the LPS/TLR4 interaction in this process, if any, remains to be investigated. Similarly, no samples were taken from skin, muscle, bone, interstitium, or circulating leukocytes, which could alter our conclusions. Finally, in the same study with Balb/c mice stimulated with LPS for 2h [49], increased levels of TNF-α, IFN-γ and IL-10 were detected in brain tissue, which suggests that the central role of the brain in regulating LPS-induced acute inflammation [51, 52] also must be integrated into the emerging picture presented in the present study.

In summary, we report on novel aspects of the complex spatiotemporal dynamics of LPS/TLR4-induced inflammation using computational modeling. This is, to our knowledge, the first study examining the dynamic evolution of some key inflammatory mediators and their interactions with each other in both the systemic circulation and within a number of targeted parenchymal organs in mice. Our results suggest that LPS-induced inflammation in TLR4^-/-^ mice is beneficial or adaptive, whereas inflammation in C57BL/6 mice is exaggerated or pathological. Thus, by employing a systems biology approach we obtain a novel perspective on the time- and organ specific components and the propagation of acute systemic inflammation, and this methodology may be useful for numerous other applications.

## Materials and methods

### Ethics statement

All procedures involving animals complied with the regulations regarding the care and use of experimental animals published by the National Institutes of Health, and were approved by the Institutional Animal Care and Use Committee of the University of Pittsburgh.

### Experimental procedures

Male TLR4^+/+^ C57BL/6 mice were purchased from Jackson Laboratory (Bar Harbor, ME, USA). TLR4-null (TLR4^-/-^) mice were bred at our facility on a C57BL/6 background [53]. Mice were allowed access to rodent chow and water *ad libitum* and used at the age of 8 and 12 weeks. Since numerous commercial LPS preparations contain measurable contaminating proteins, for this study we utilized ultra-purified LPS (from Escherichia coli O111:B4) purchased from List Biological Laboratories, Inc. (Campbell, CA). Littermate mice (C57BL/6: n = 5–8 animals; TLR4^-/-^: n = 4 animals for each experimental group) were injected with LPS using 3 mg/kg (i.p.). At different time-points (0, 1, 4, 6, 12, 24 and 48 h), the animals were anesthetized with isoflurane, cardiac puncture was performed, blood was collected into heparinized tubes, and then centrifuged to obtain plasma; the mice were then euthanized by cervical dislocation while under anesthesia. Mice were then perfused with ice-cold PBS followed by RNALater (Thermo Fisher Scientific, Waltham, MA), which we have previously shown to be a preservation method compatible with Luminex analysis and equivalent to flash-freezing in liquid nitrogen [54]. A small section (approx. 100 mg) of each tissue (liver [left lobe], heart, gut [terminal ileum], lung [left lobe], spleen, and kidney [left]) was collected and stored at -80°C until analysis. Total protein isolation and determination was done as previously described [55].

### Analysis of inflammatory mediators

Mouse inflammatory mediators were measured using a Luminex 100 IS apparatus (Luminex, Austin, TX) and the BioSource 20-plex mouse cytokine bead kit (BioSource-Invitrogen, San Diego, CA) as per manufacturer’s specifications. The antibody bead kit included: Granulocyte-Macrophage Colony-Stimulating Factor (**GM-CSF**), Interferon-γ (**IFN-γ**), Interleukin (**IL**)**-1α, IL-1β, IL-2, IL-4, IL-5, IL-6, IL-10, IL-12p40, IL-12p70, IL-13, IL-17A,** Interferon-γ-inducible Protein 10 (**IP-10/CXCL10**), Keratinocyte-derived Cytokine (**KC/CXCL1**), Monocyte Chemoattractant Protein-1 (**MCP-1/CCL2**), Monokine induced by Interferon-γ (**MIG/CXCL9**), Macrophage Inflammatory Protein-1α (**MIP-1α/CCL3**), Tumor Necrosis Factor-α (**TNF-α**), and Vascular Endothelial Growth Factor (**VEGF**). The final mediator concentrations are expressed in pg/ml for plasma samples, and in pg/mg total protein for tissue samples. Experimental data are presented as mean ± SEM.

### Assays of liver damage

Serum alanine aminotransferase (ALT) was measured with HESKA Dri-Chem 4000 Chemistry Analyzer System (HESKA; slides from Fujifilm Japan).

### Statistical and computational analyses

Our analytic strategy was to apply a stepwise series of data-driven modeling techniques aimed at discovering principal drivers based on data on systemic and organ-specific acute inflammation. We detail these analyses below:

1. **Two-Way Analysis of Variance (ANOVA)** was carried out to analyze the time-dependent changes in inflammatory mediators in C57BL/6 vs. TLR4^-/-^ mice in all organs as well as in plasma, using *SigmaPlot* (Systat Software, San Jose, CA) as indicated.
2. **Time-Interval Principal Component Analysis (TI-PCA)** was carried out in order to identify those inflammatory mediators that contributed to the top 25% variance of the response to LPS in C57BL/6 vs. TLR4^-/-^ mice (all organs as well as in the systemic circulation) over six consecutive time periods (0-1h, 1-4h, 4-6h, 6-12h, 12-24h, and 24-48h) using MATLAB software (The MathWorks, Inc., Natick, MA) [18] (see S1 MATLAB code).
3. **Pearson’s correlation** was carried out to measure the strength of association among tissue-specific inflammatory mediator data in all organs as well as in plasma in C57BL/6 vs. TLR4^-/-^ mice. Luminex data were first standardized to have mean zero and scaled to have standard deviation 1 (z-scored). At each time point, the Pearson's correlation coefficients were calculated for each 20x20 correlation matrix of mediators' interactions, and only the statistically significant (P<0.05) correlations were used in the analysis.

## Supporting information

S1 Fig Time-dependent release of inflammatory mediators in LPS-treated mice. C57BL/6 (open circles, n = 5–8 for each experimental group) and TLR4^-/-^ (closed circles, n = 4 for each experimental group) mice were injected with LPS (3 mg/kg, i.p.). At different time points (0, 1, 4, 6, 12, 24 and 48 h) upon sacrifice, the inflammatory mediators in blood and different organs (liver, heart, gut, lung, spleen and kidney) were measured by Luminex as described in *Materials and Methods*. Values are mean ± SEM (*P <0.05, C57BL/6 vs. TLR4^-/-^, analyzed by Two-Way ANOVA followed by Holm-Sidak method).
(PDF)

S2 Fig Analysis of endotoxemic mice by PCA. C57BL/6 (n = 5–8 for each experimental group) and TLR4^-/-^ (n = 4 for each experimental group) mice were injected with LPS (3 mg/kg, i.p.). At different time points (0, 1, 4, 6, 12, 24 and 48 h) upon sacrifice, the inflammatory mediators in blood and different organs (liver, heart, gut, lung, spleen and kidney) were measured by Luminex and PCA analysis during the entire time-course (0-48h) was performed as described in *Materials and Methods*.
(PDF)

S1 Luminex Data (PDF)

S1 MATLAB Code (DOCX)
