# Supplementary material for: A computational analysis of dynamic, multi-organ inflammatory crosstalk induced by endotoxin in mice
Source: PLoS Comput Biol. 2018 Nov 6;14(11):e1006582. doi: 10.1371/journal.pcbi.1006582 (PMC6239343; doi:10.1371/journal.pcbi.1006582)
Supplement: S1 Fig — C57BL/6 (open circles, n = 5–8 for each experimental group) and TLR4-/- (closed circles, n = 4 for each experimental group) mice were injected with LPS (3 mg/kg, i.p.). At different time points (0, 1, 4, 6, 12, 24 and 48 h) upon sacrifice, the inflammatory mediators in blood and different organs (liver, heart, gut, lung, spleen and kidney) were measured by Luminex as described in Materials and Methods. Values are mean ± SEM (*P <0.05, C57BL/6 vs. TLR4-/-, analyzed by Two-Way ANOVA followed by Holm-Sidak method). (PDF) [file pcbi.1006582.s001.pdf]

S1 Fig.

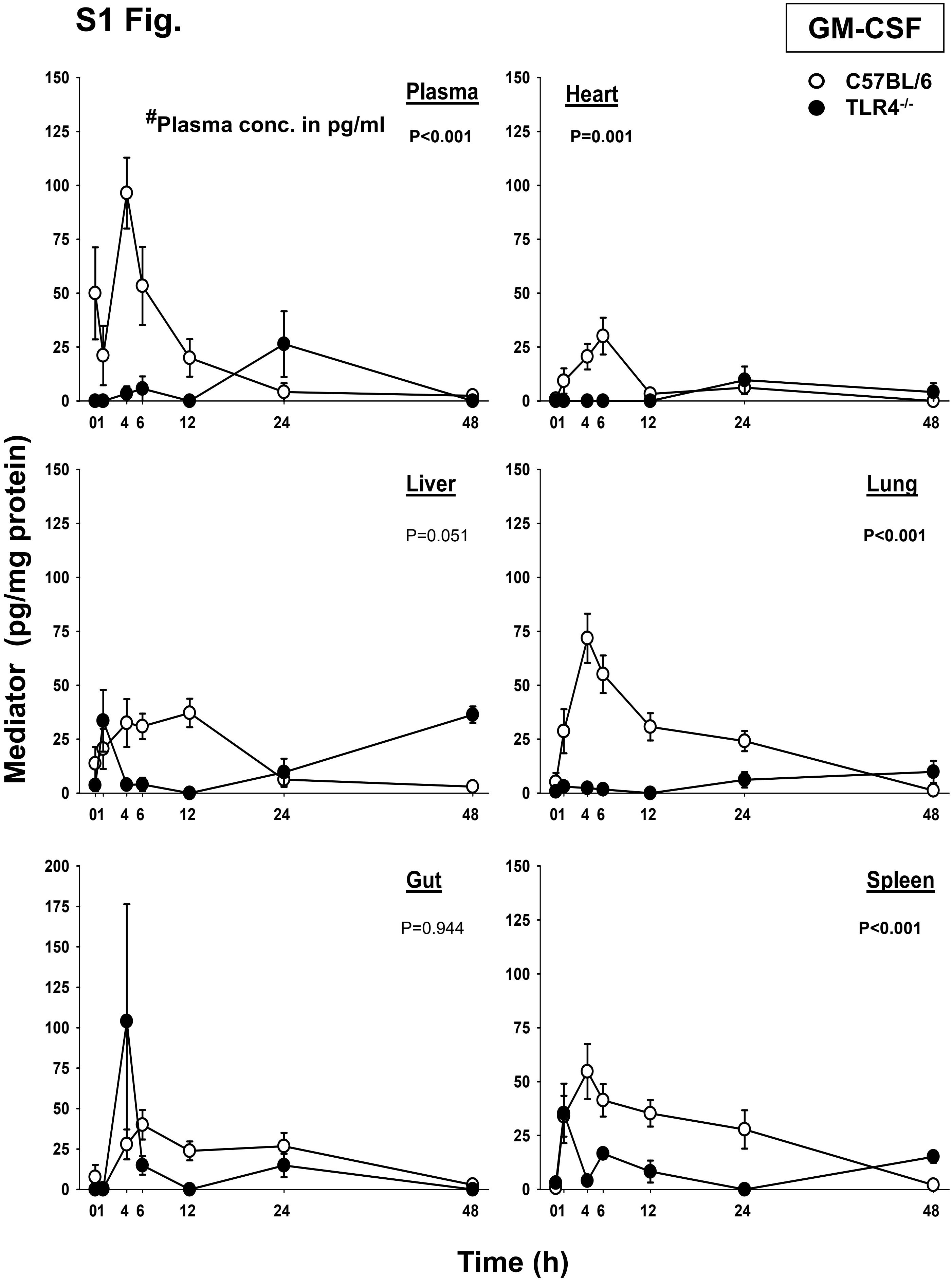
GM-CSF
C57BL/6
TLR4-/-
Plasma
#Plasma conc. in pg/ml
P<0.001
Heart
P=0.001
Liver
P=0.051
Lung
P<0.001
Gut
P=0.944
Spleen
P<0.001
Mediator (pg/mg protein)
Time (h)
0 1 4 6 12 24 48

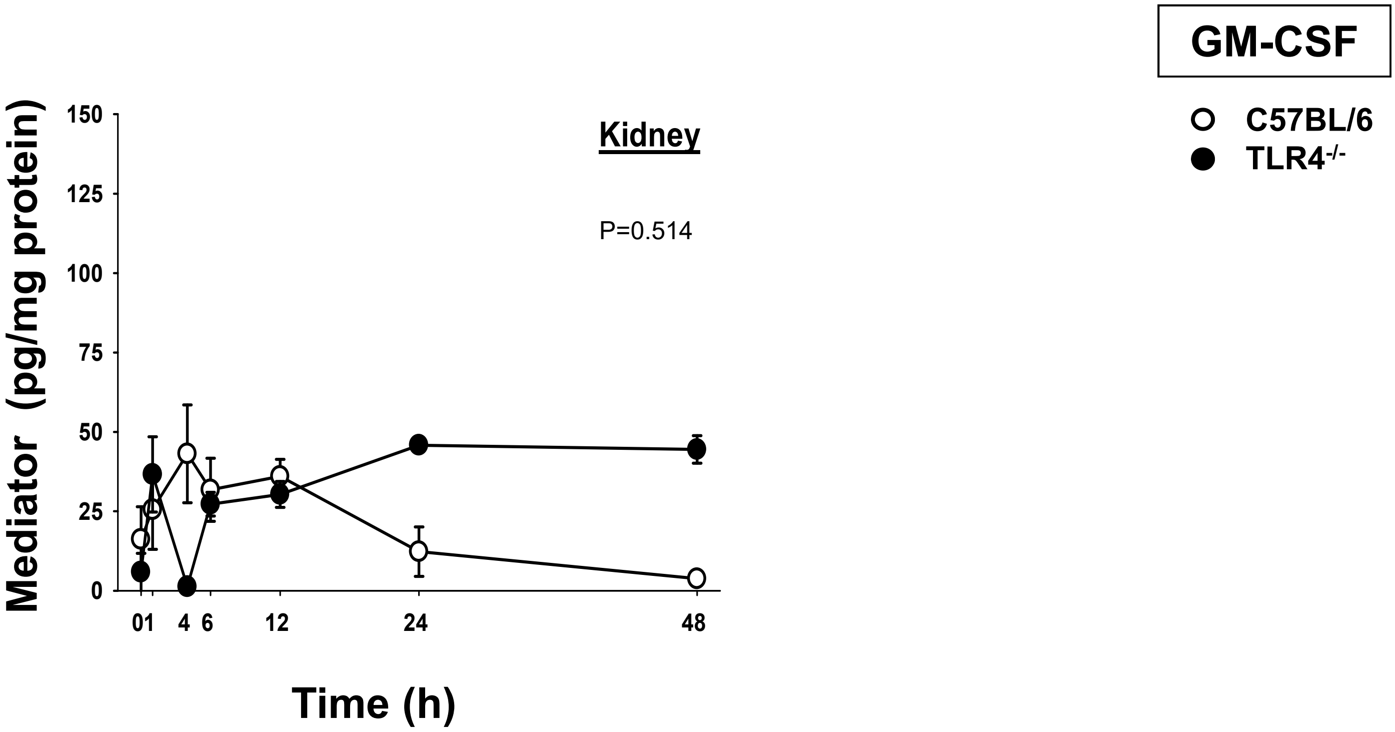
GM-CSF
C57BL/6
TLR4-/-
Kidney
P=0.514
Mediator (pg/mg protein)
150
125
100
75
50
25
0
01
4 6
12
24
48
Time (h)

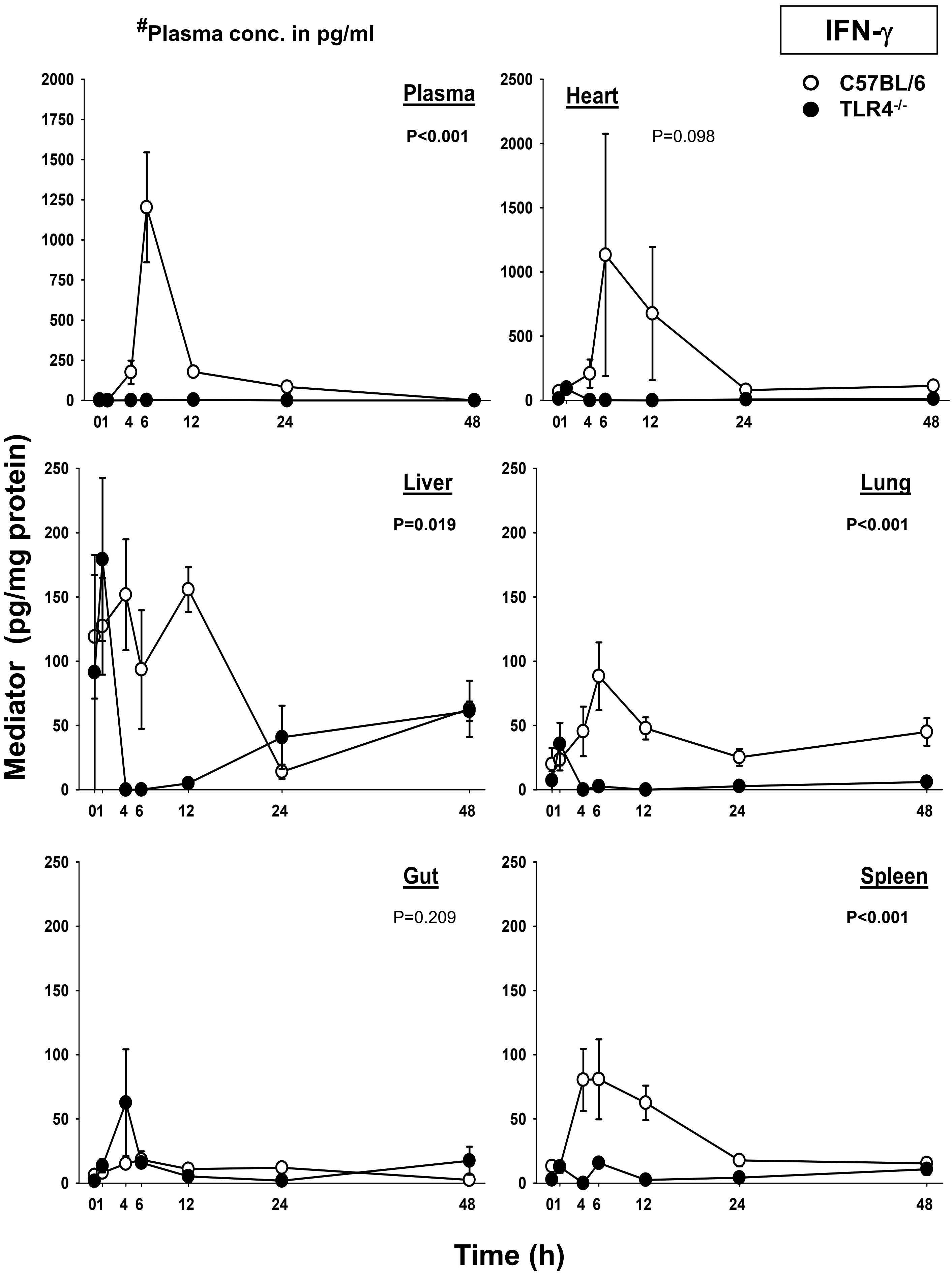

#Plasma conc. in pg/ml
IFN-γ
C57BL/6
TLR4-/-
Plasma
P<0.001
Heart
P=0.098
Liver
P=0.019
Lung
P<0.001
Gut
P=0.209
Spleen
P<0.001
Mediator (pg/mg protein)
Time (h)
0 1 4 6 12 24 48

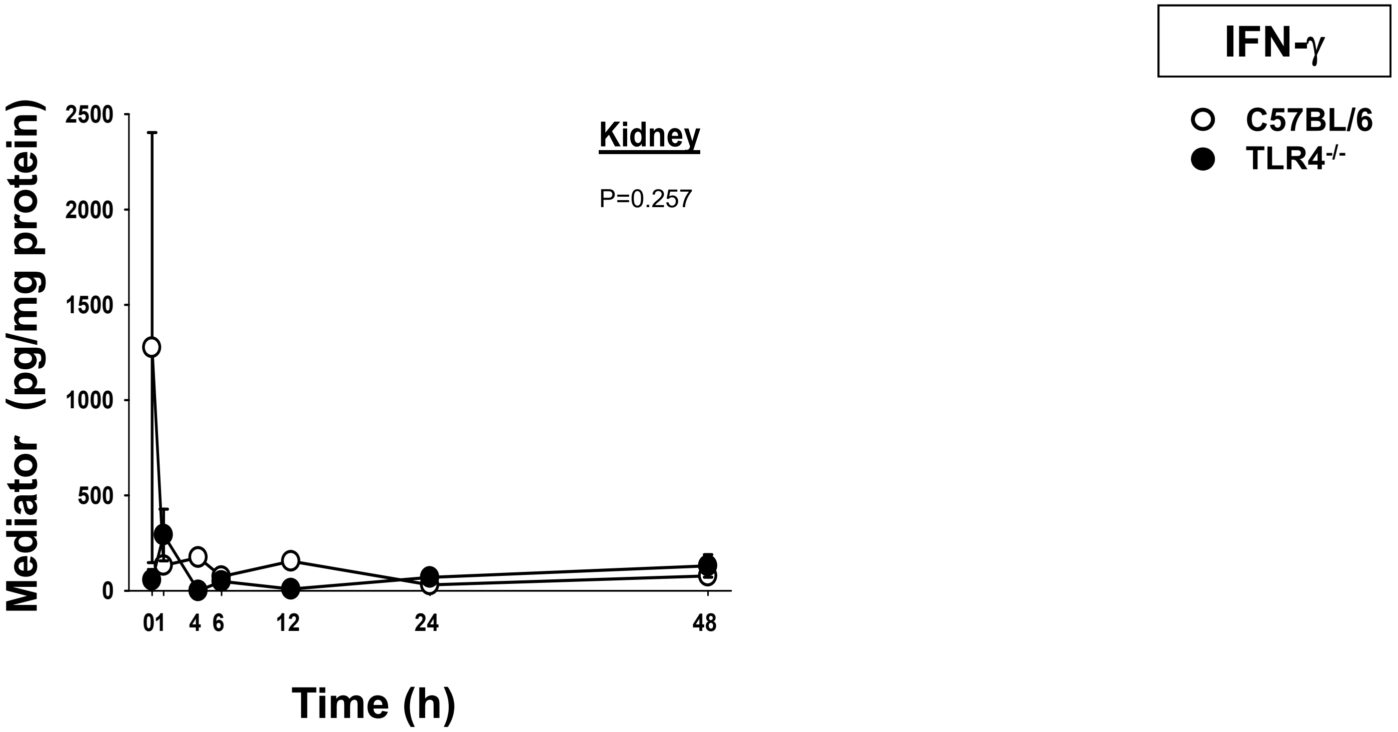

IFN-γ
C57BL/6
TLR4-/-
Kidney
P=0.257
Mediator (pg/mg protein)
2500
2000
1500
1000
500
0
0 1
4 6
12
24
48
Time (h)

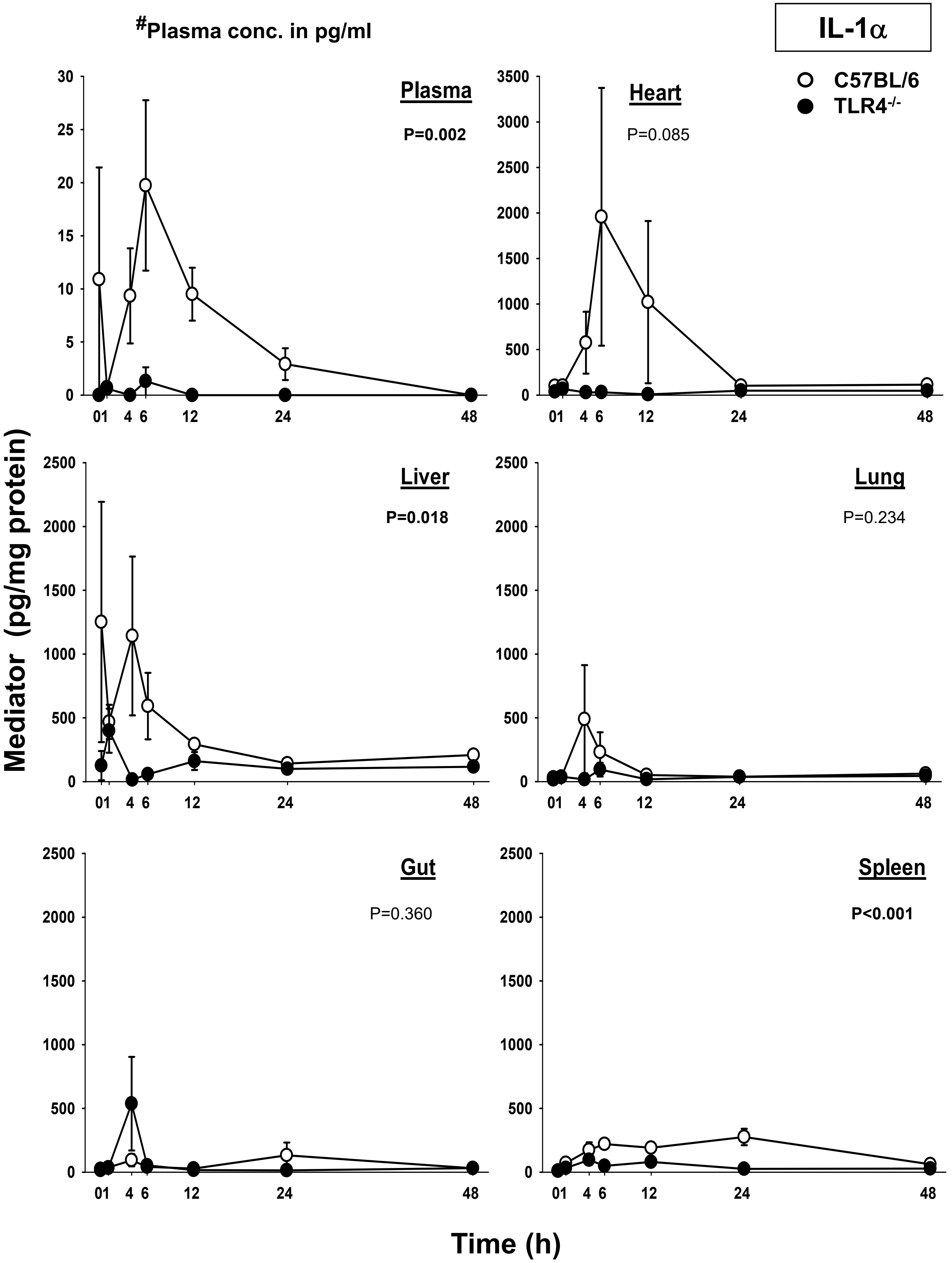

#Plasma conc. in pg/ml
IL-1α
C57BL/6
TLR4-/-
Plasma
P=0.002
Heart
P=0.085
Liver
P=0.018
Lung
P=0.234
Gut
P=0.360
Spleen
P<0.001
Mediator (pg/mg protein)
Time (h)

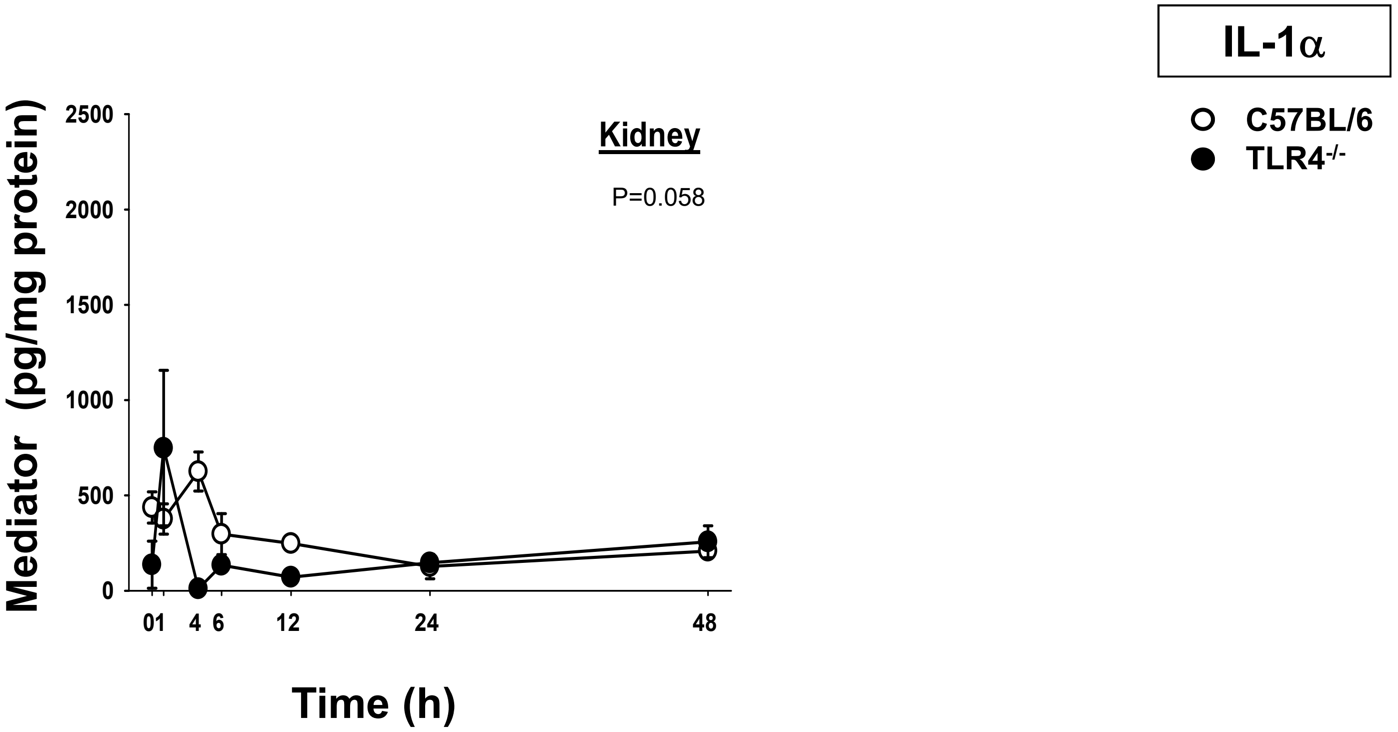
IL-1α
C57BL/6
TLR4-/-
Kidney
P=0.058
Mediator (pg/mg protein)
2500
2000
1500
1000
500
0
0 1 4 6 12 24 48
Time (h)

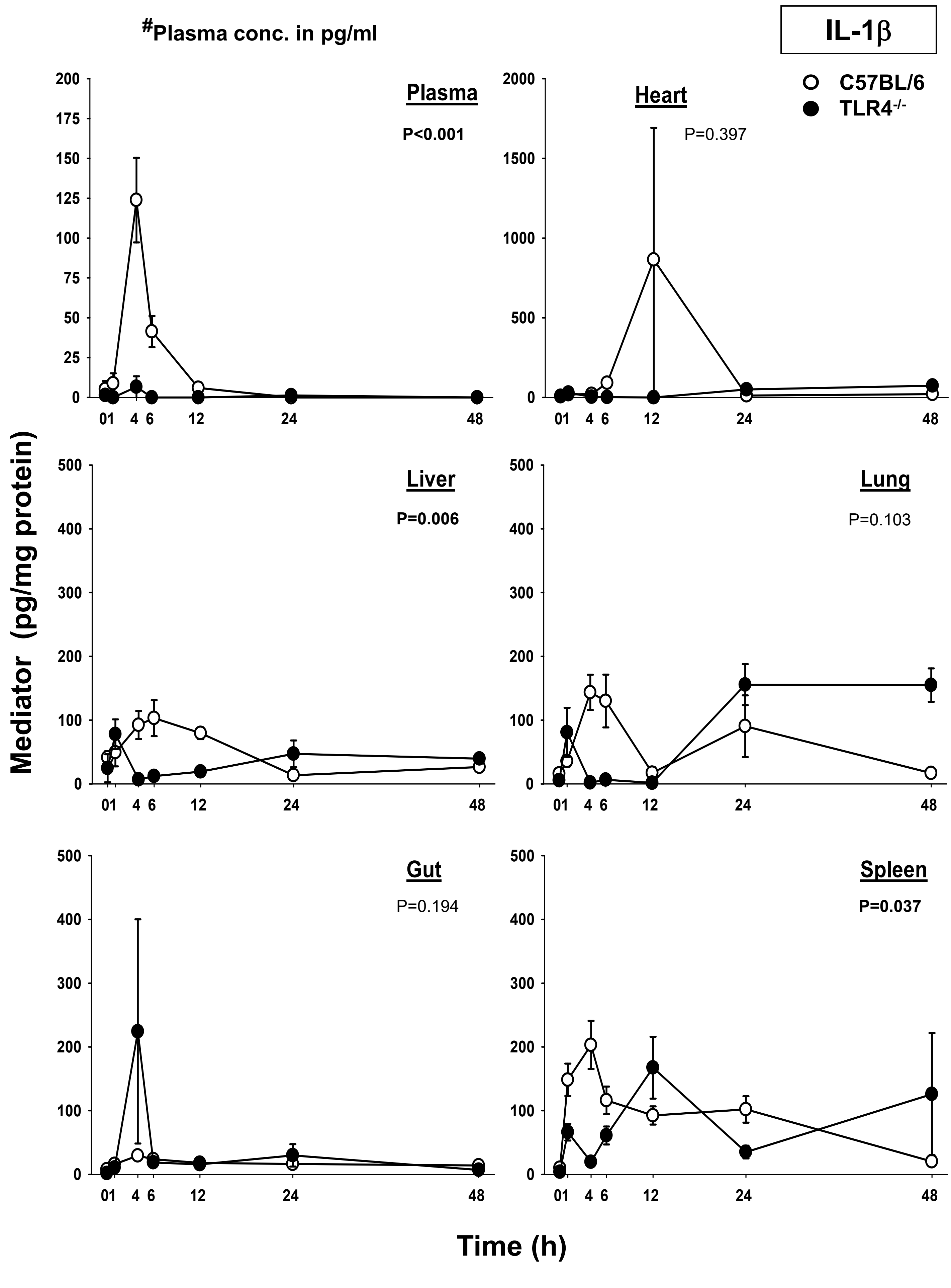

#Plasma conc. in pg/ml
IL-1β
C57BL/6
TLR4-/-
Plasma
P<0.001
Heart
P=0.397
Liver
P=0.006
Lung
P=0.103
Gut
P=0.194
Spleen
P=0.037
Mediator (pg/mg protein)
Time (h)

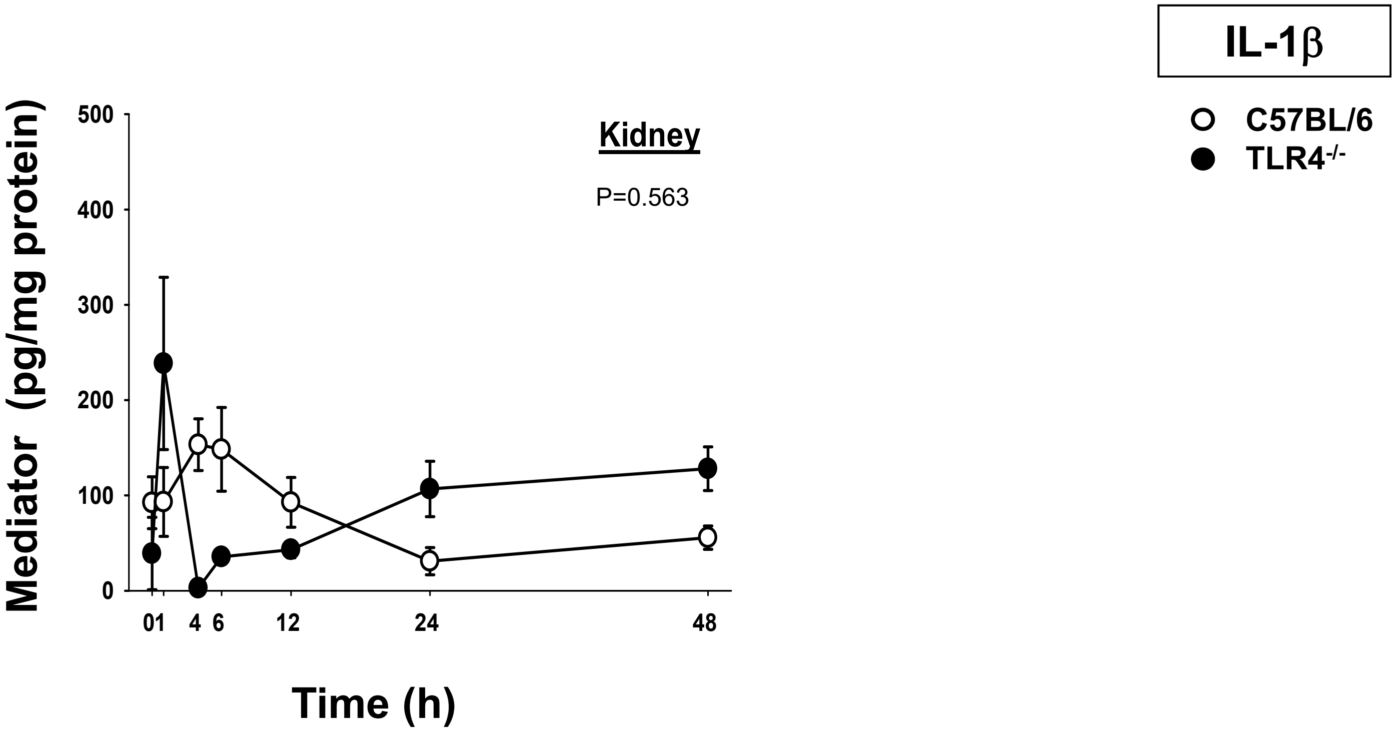

IL-1β
C57BL/6
TLR4-/-
Kidney
P=0.563
Mediator (pg/mg protein)
500
400
300
200
100
0
0 1 4 6 12 24 48
Time (h)

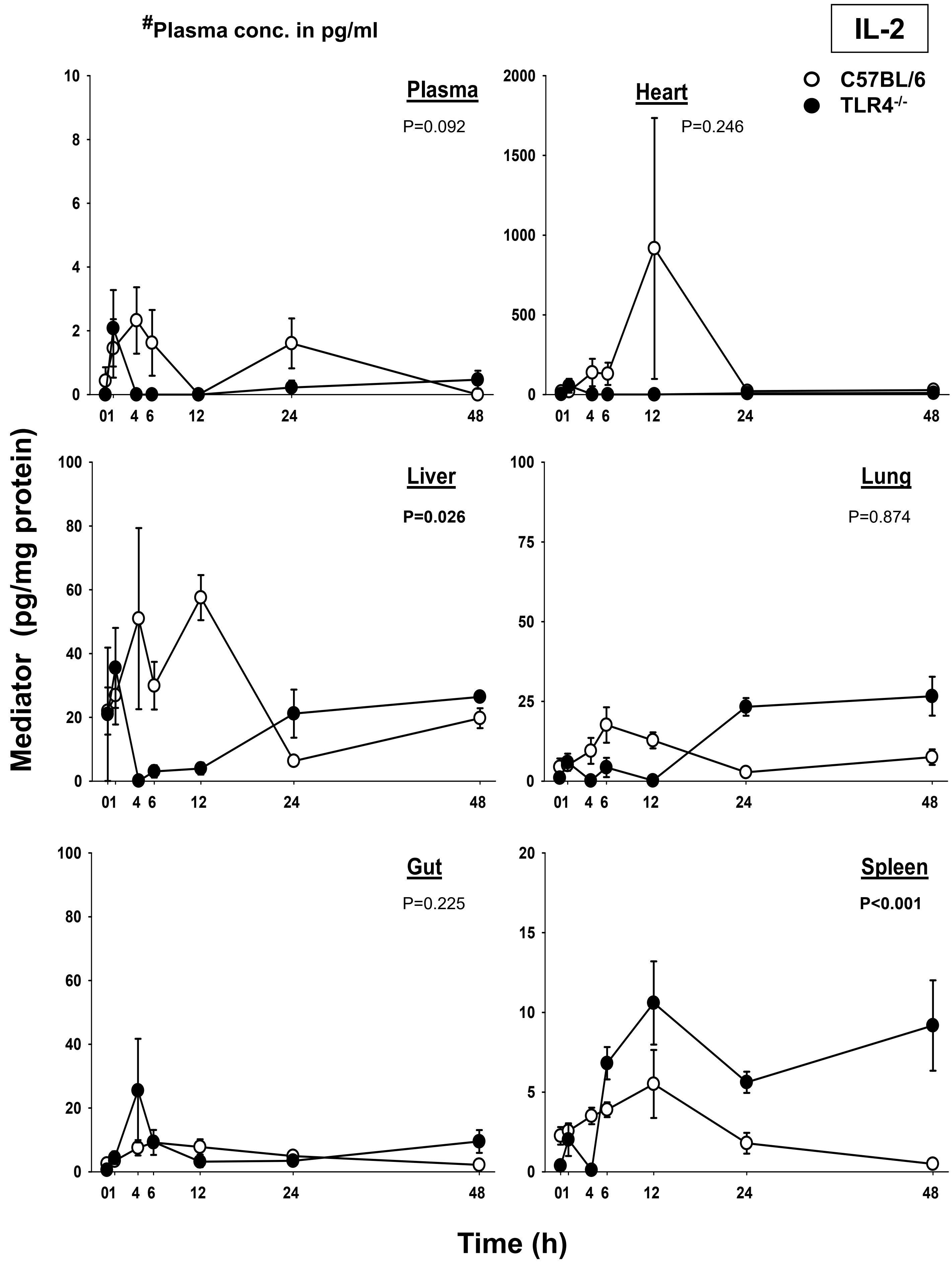

#Plasma conc. in pg/ml
IL-2
C57BL/6
TLR4-/-
Plasma
P=0.092
Heart
P=0.246
Liver
P=0.026
Lung
P=0.874
Gut
P=0.225
Spleen
P<0.001
Mediator (pg/mg protein)
Time (h)

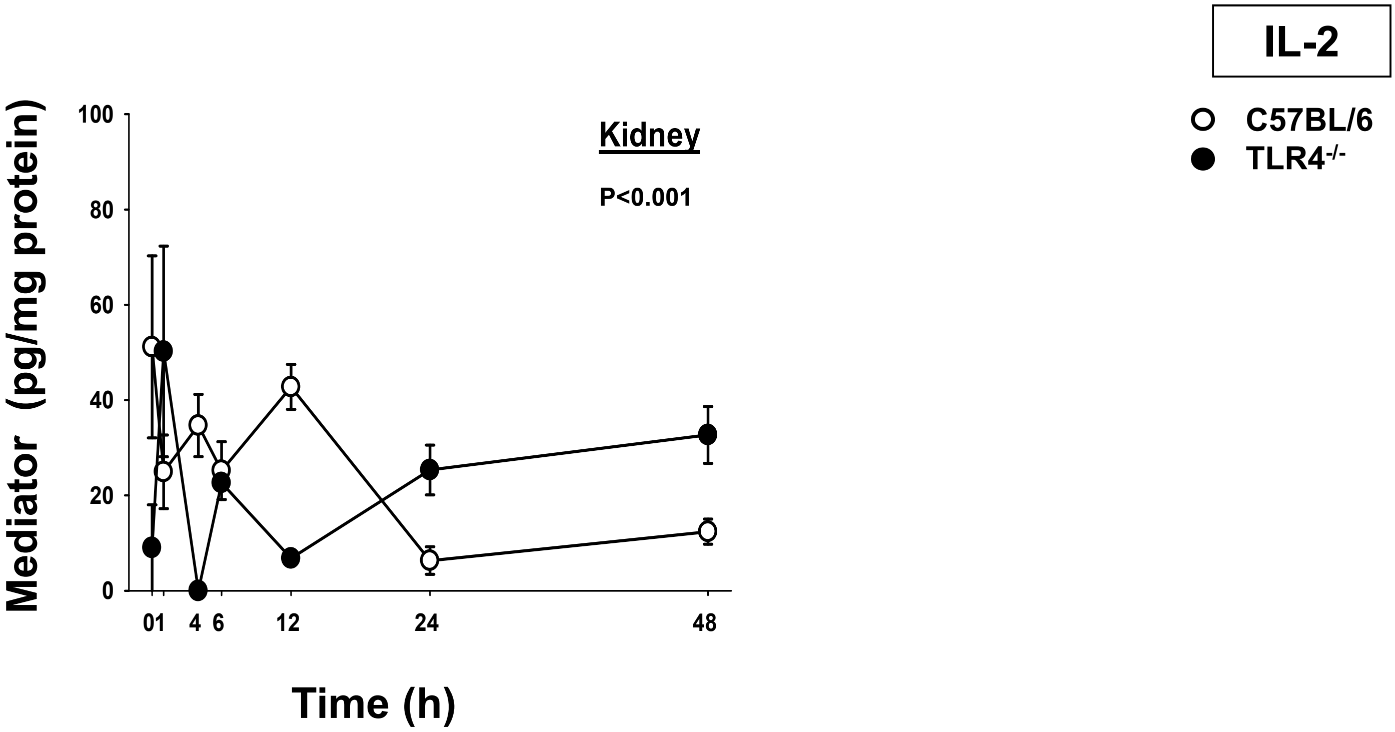

IL-2
C57BL/6
TLR4-/-
Kidney
P<0.001
Mediator (pg/mg protein)
100
80
60
40
20
0
01
4
6
12
24
48
Time (h)

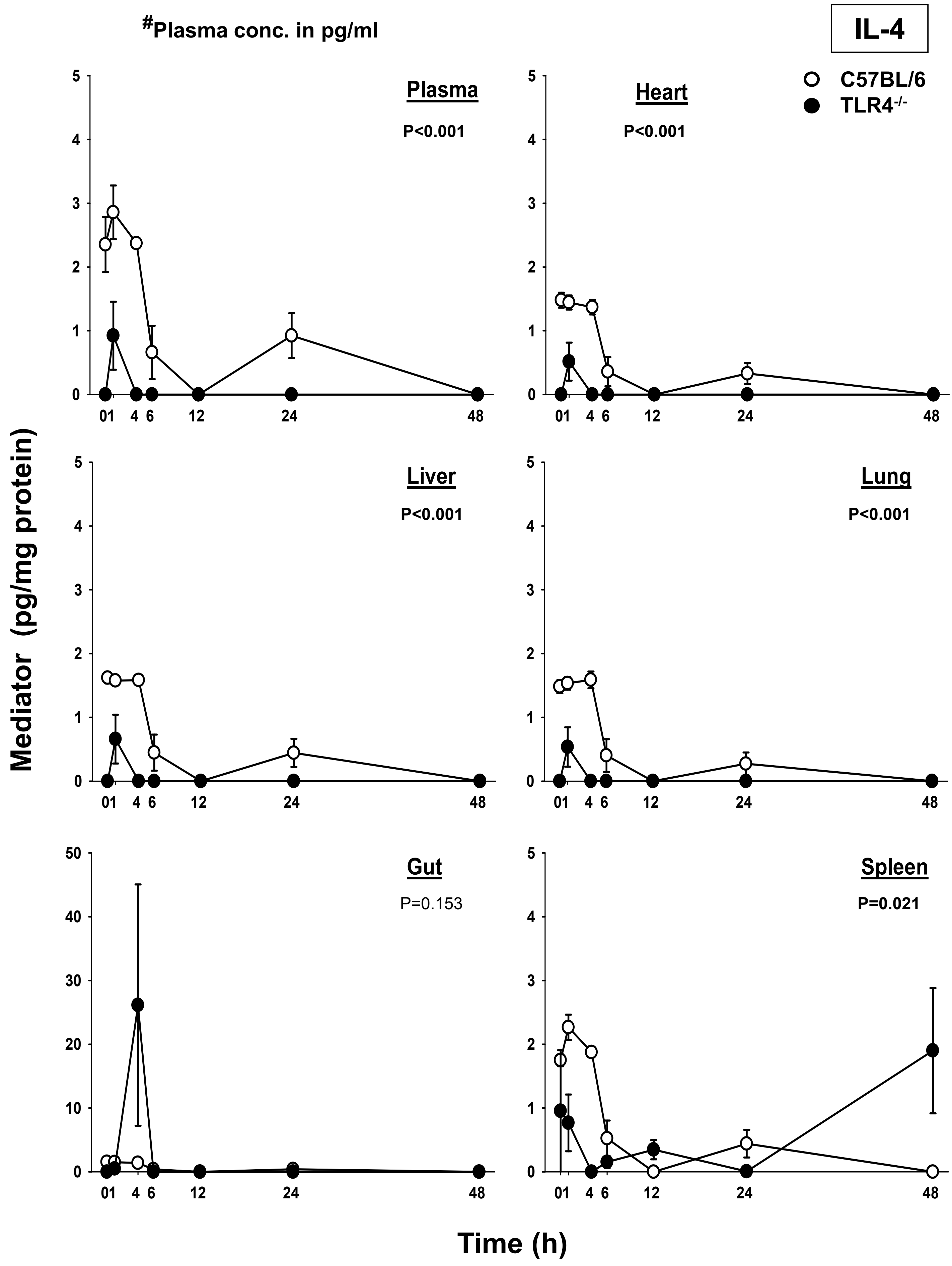

#Plasma conc. in pg/ml
IL-4
C57BL/6
TLR4-/-
Plasma
P<0.001
Heart
P<0.001
Liver
P<0.001
Lung
P<0.001
Gut
P=0.153
Spleen
P=0.021
Mediator (pg/mg protein)
Time (h)

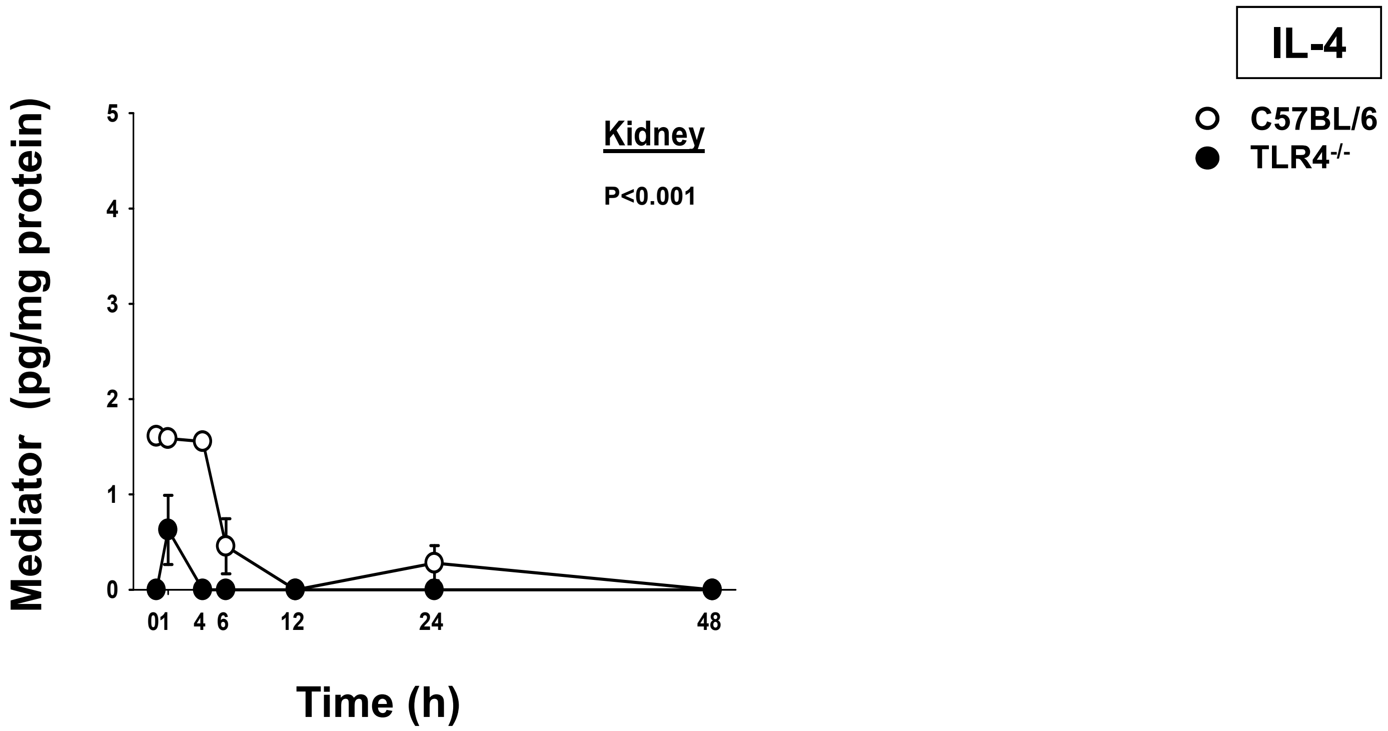

IL-4
C57BL/6
TLR4-/-
Kidney
P<0.001
Mediator (pg/mg protein)
5
4
3
2
1
0
01
4
6
12
24
48
Time (h)

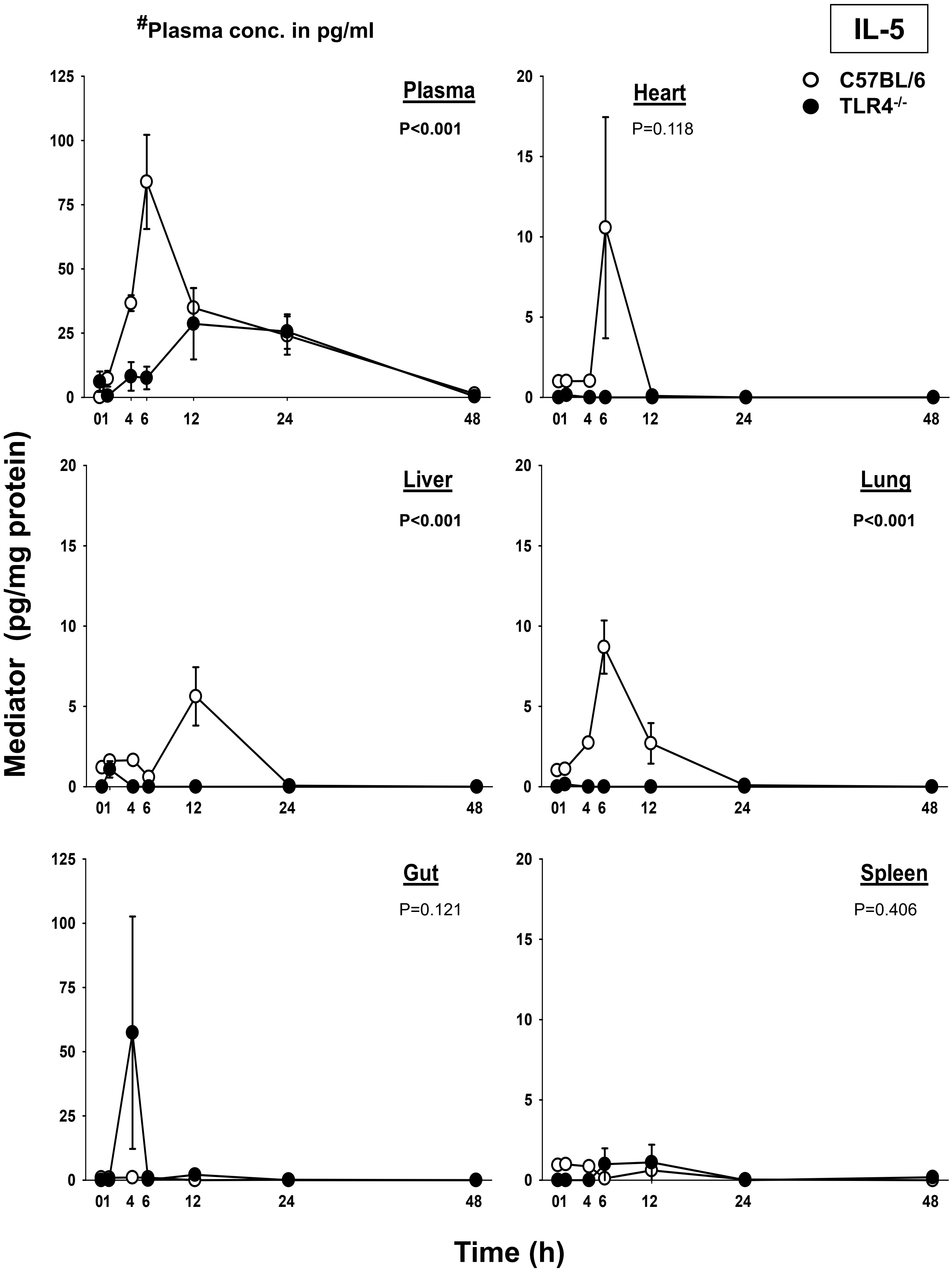

#Plasma conc. in pg/ml
IL-5
C57BL/6
TLR4-/-
Plasma
P<0.001
Heart
P=0.118
Liver
P<0.001
Lung
P<0.001
Gut
P=0.121
Spleen
P=0.406
Mediator (pg/mg protein)
Time (h)

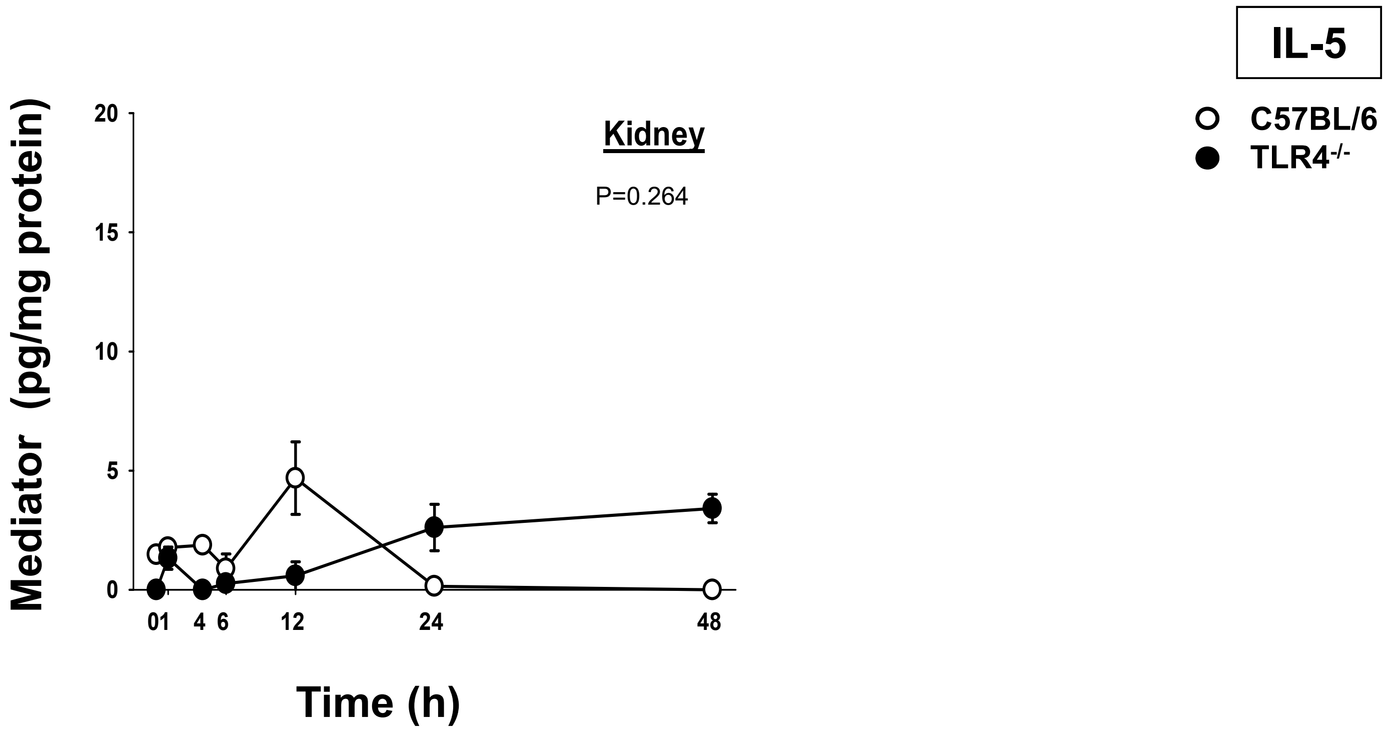
IL-5
C57BL/6
TLR4-/-
Kidney
P=0.264
Mediator (pg/mg protein)
20
15
10
5
0
0 1 4 6 12 24 48
Time (h)

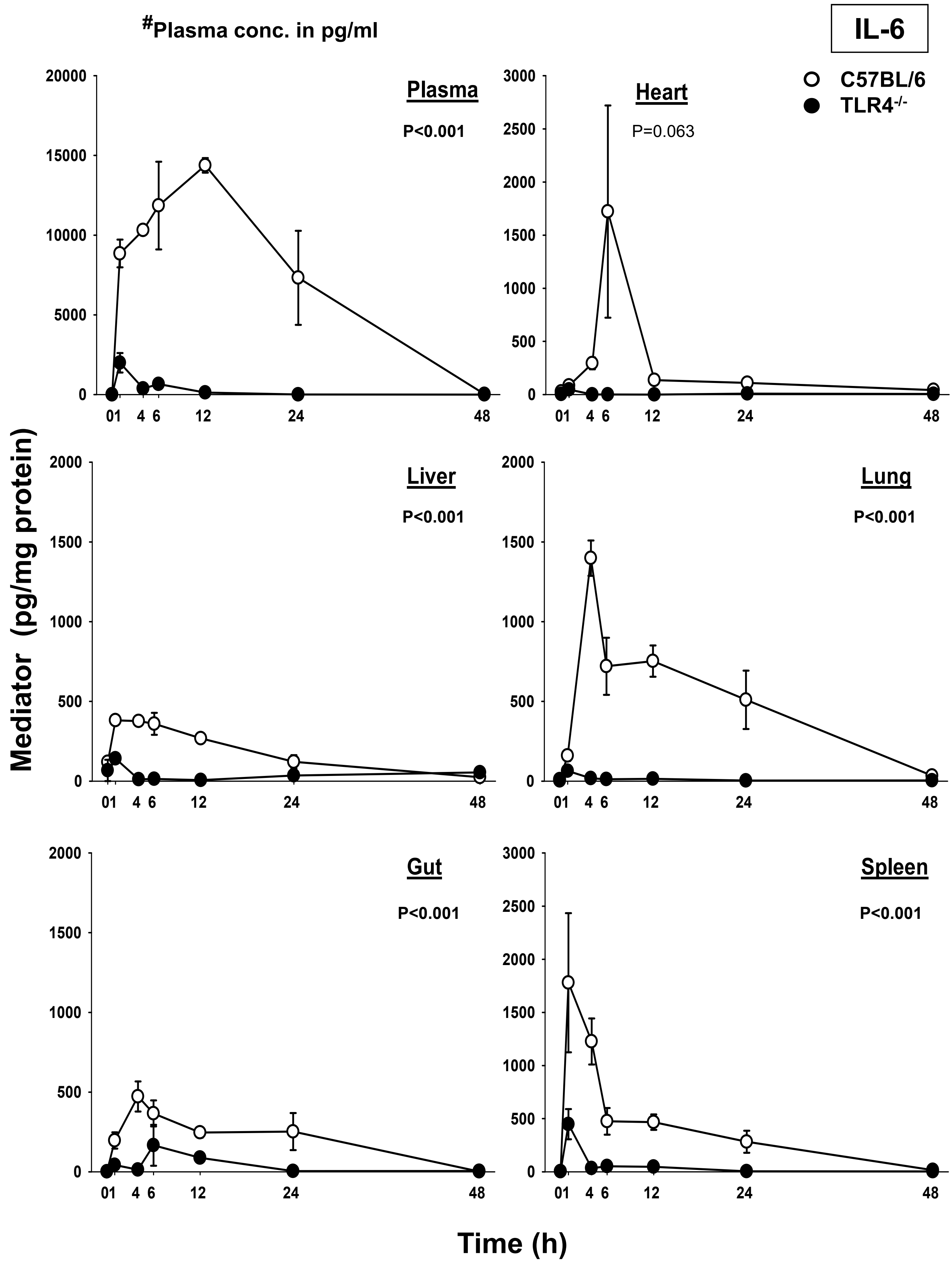

#Plasma conc. in pg/ml
IL-6
C57BL/6
TLR4-/-
Plasma
P<0.001
Heart
P=0.063
Liver
P<0.001
Lung
P<0.001
Gut
P<0.001
Spleen
P<0.001
Mediator (pg/mg protein)
Time (h)

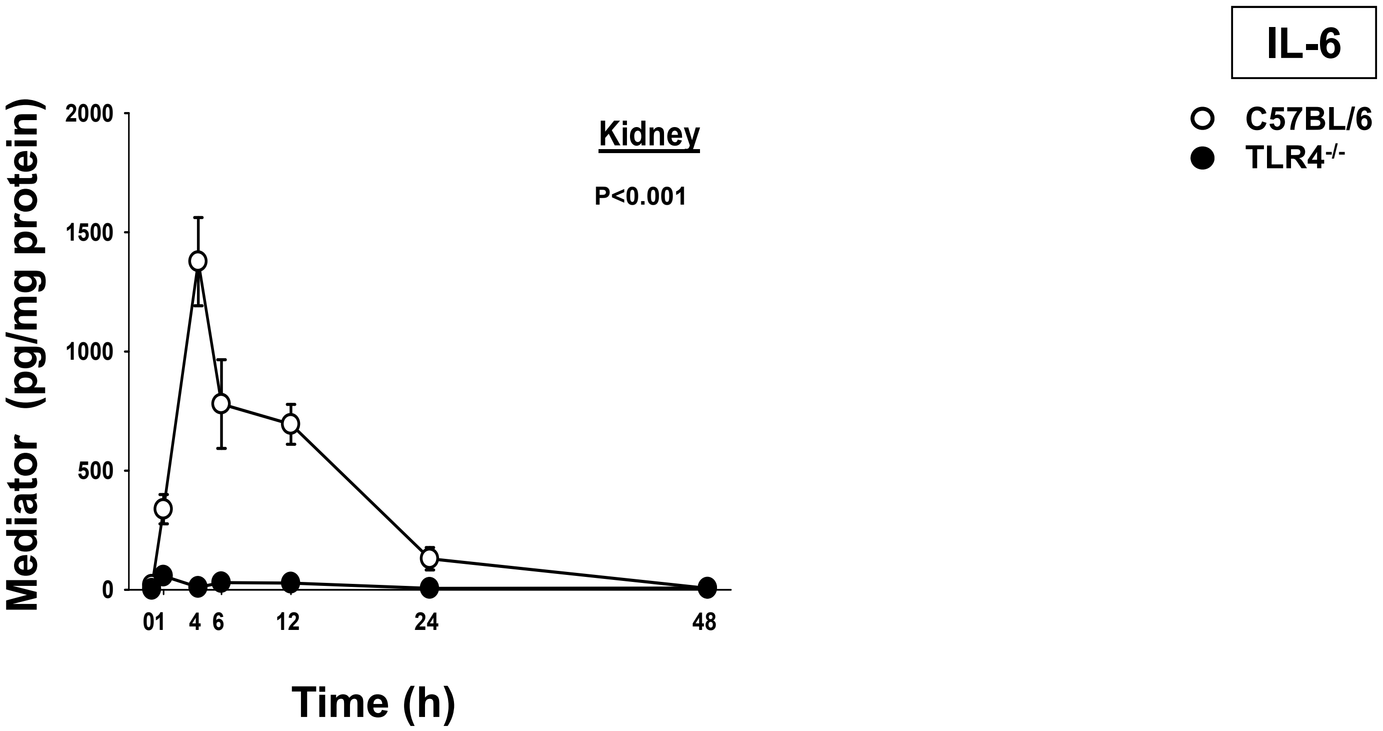
IL-6
C57BL/6
TLR4-/-
Kidney
P<0.001
Mediator (pg/mg protein)
2000
1500
1000
500
0
0 1 4 6 12 24 48
Time (h)

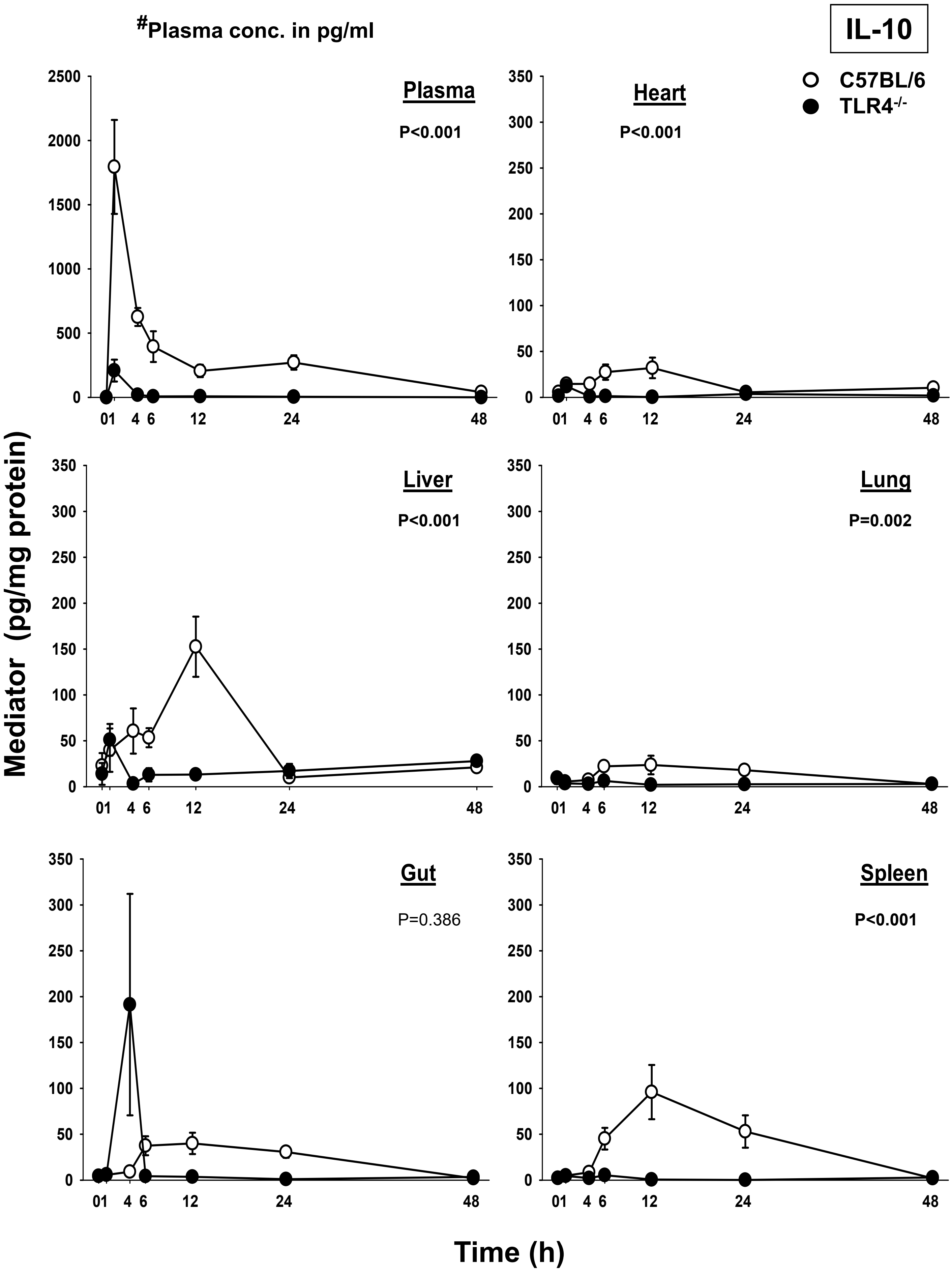

#Plasma conc. in pg/ml
IL-10
C57BL/6
TLR4-/-
Plasma
P<0.001
Heart
P<0.001
Liver
P<0.001
Lung
P=0.002
Gut
P=0.386
Spleen
P<0.001
Mediator (pg/mg protein)
Time (h)

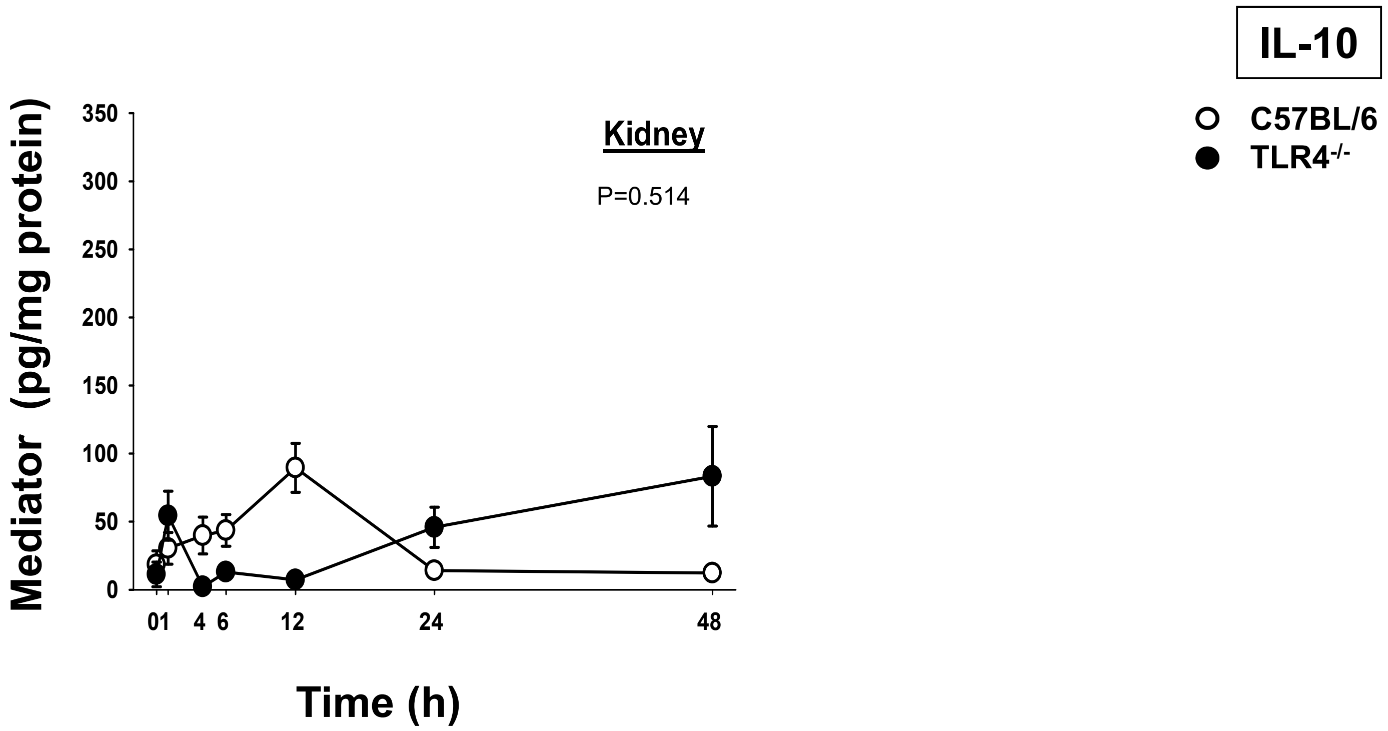
IL-10
C57BL/6
TLR4-/-
Kidney
P=0.514
Mediator (pg/mg protein)
350
300
250
200
150
100
50
0
0
1
4
6
12
24
48
Time (h)

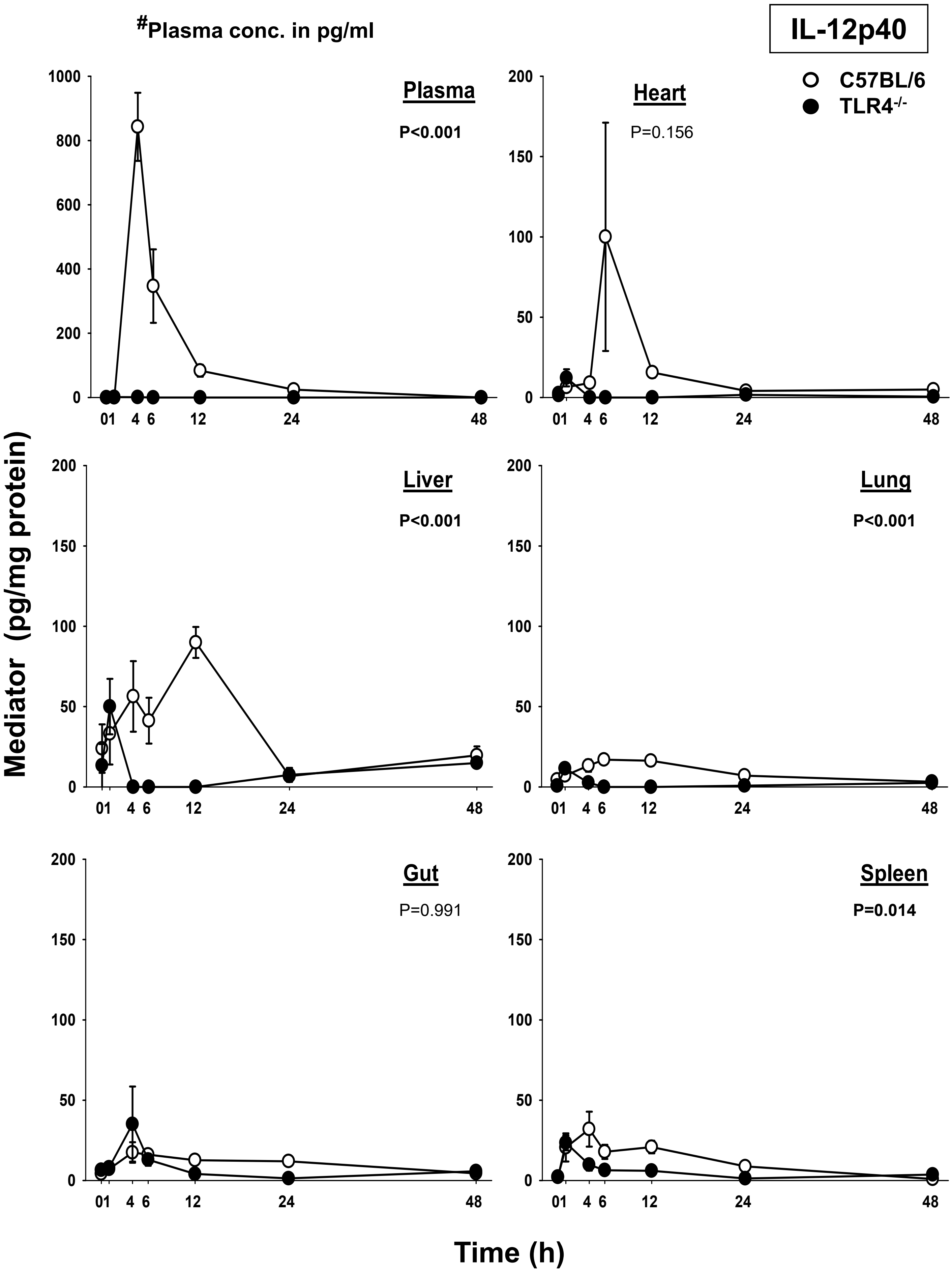

#Plasma conc. in pg/ml
IL-12p40
C57BL/6
TLR4-/-
Plasma
P<0.001
Heart
P=0.156
Liver
P<0.001
Lung
P<0.001
Gut
P=0.991
Spleen
P=0.014
Mediator (pg/mg protein)
Time (h)

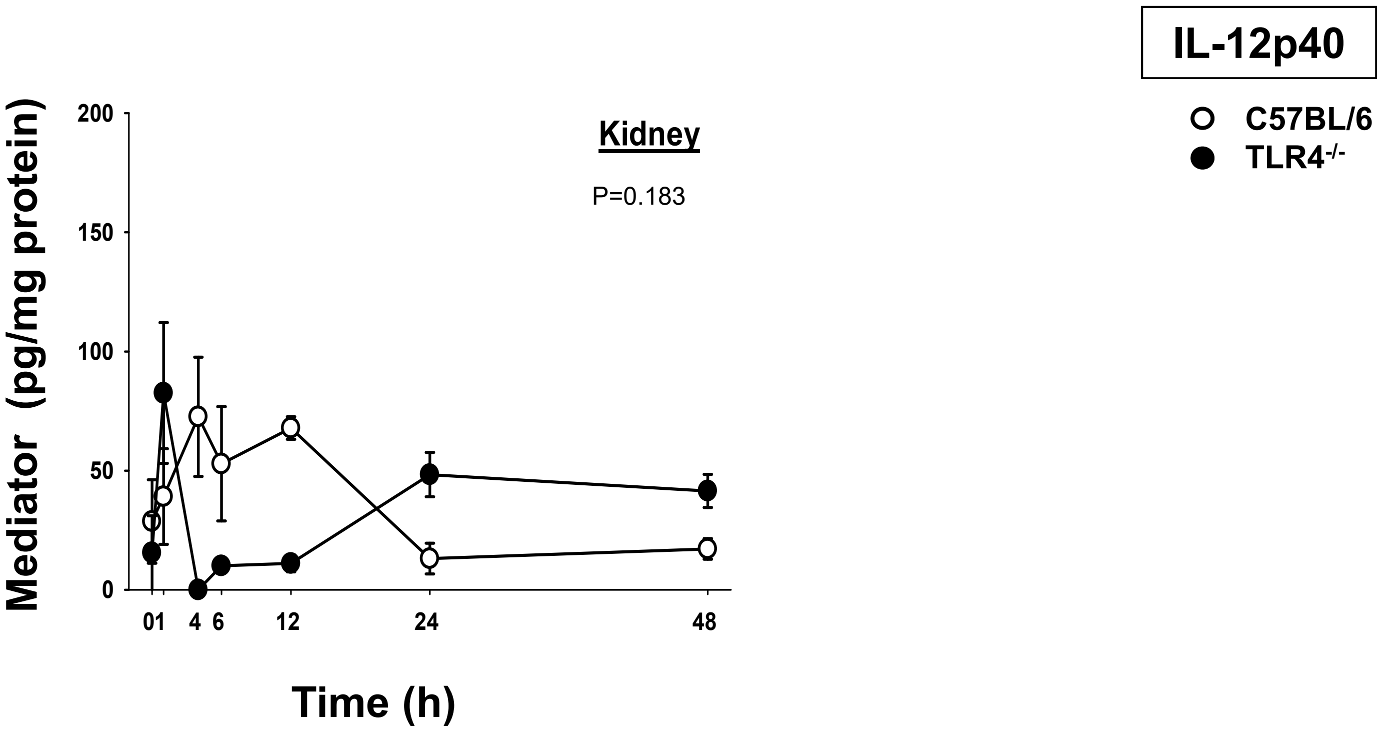
IL-12p40
C57BL/6
TLR4-/-
Kidney
P=0.183
Mediator (pg/mg protein)
200
150
100
50
0
0 1 4 6 12 24 48
Time (h)

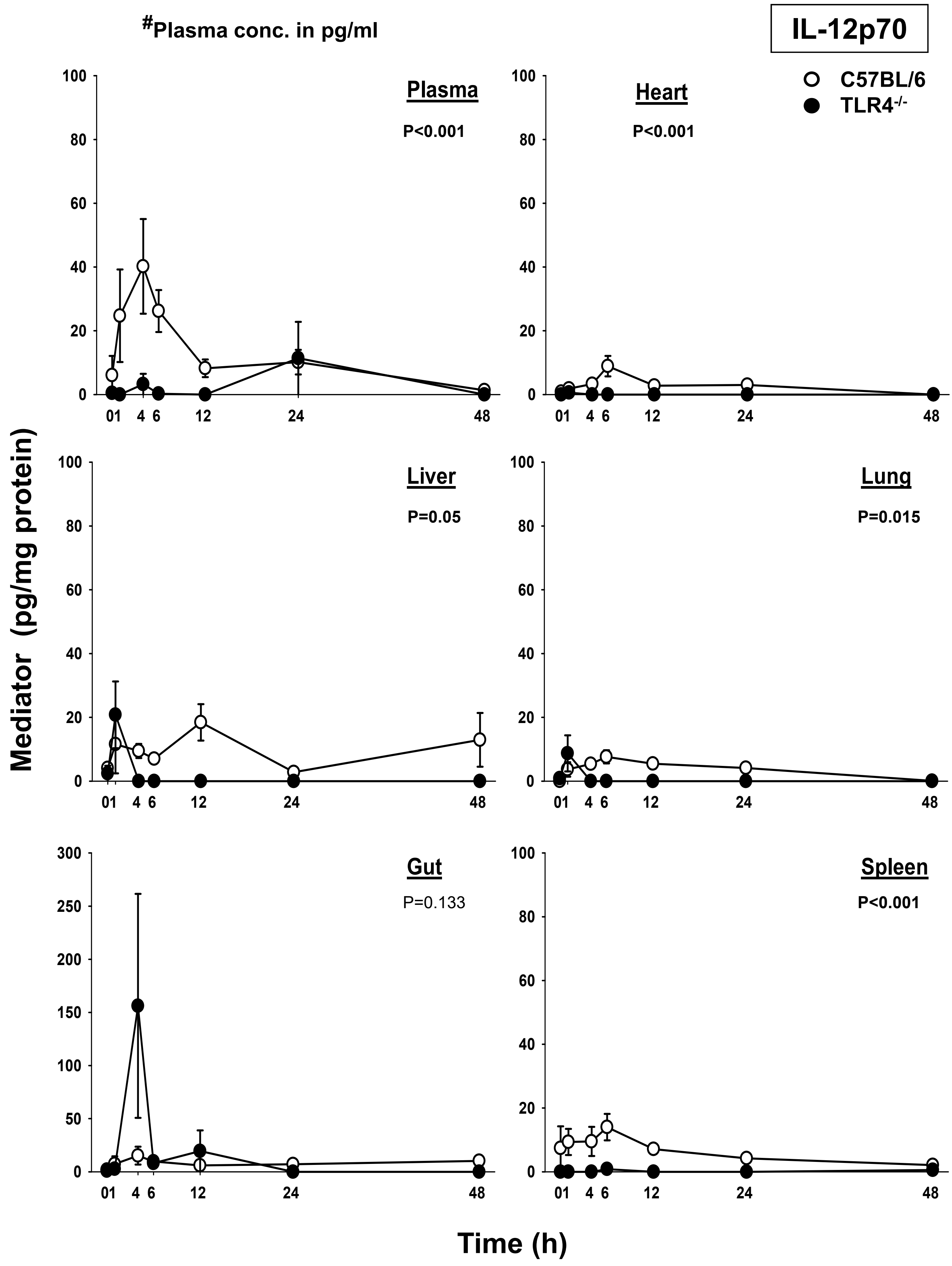

#Plasma conc. in pg/ml
IL-12p70
C57BL/6
TLR4-/-
Plasma
P<0.001
Heart
P<0.001
Liver
P=0.05
Lung
P=0.015
Gut
P=0.133
Spleen
P<0.001
Mediator (pg/mg protein)
Time (h)

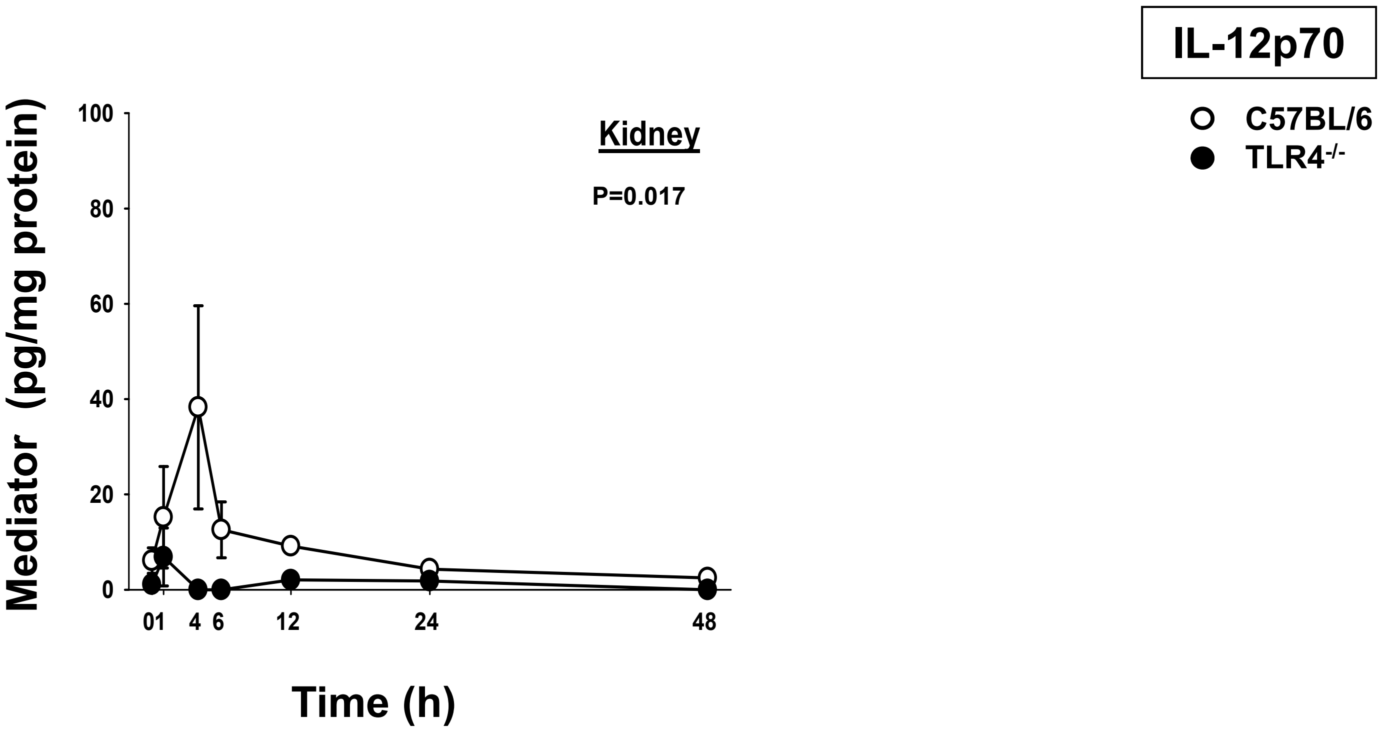
IL-12p70
C57BL/6
TLR4-/-
Kidney
P=0.017
Mediator (pg/mg protein)
100
80
60
40
20
0
01
4
6
12
24
48
Time (h)

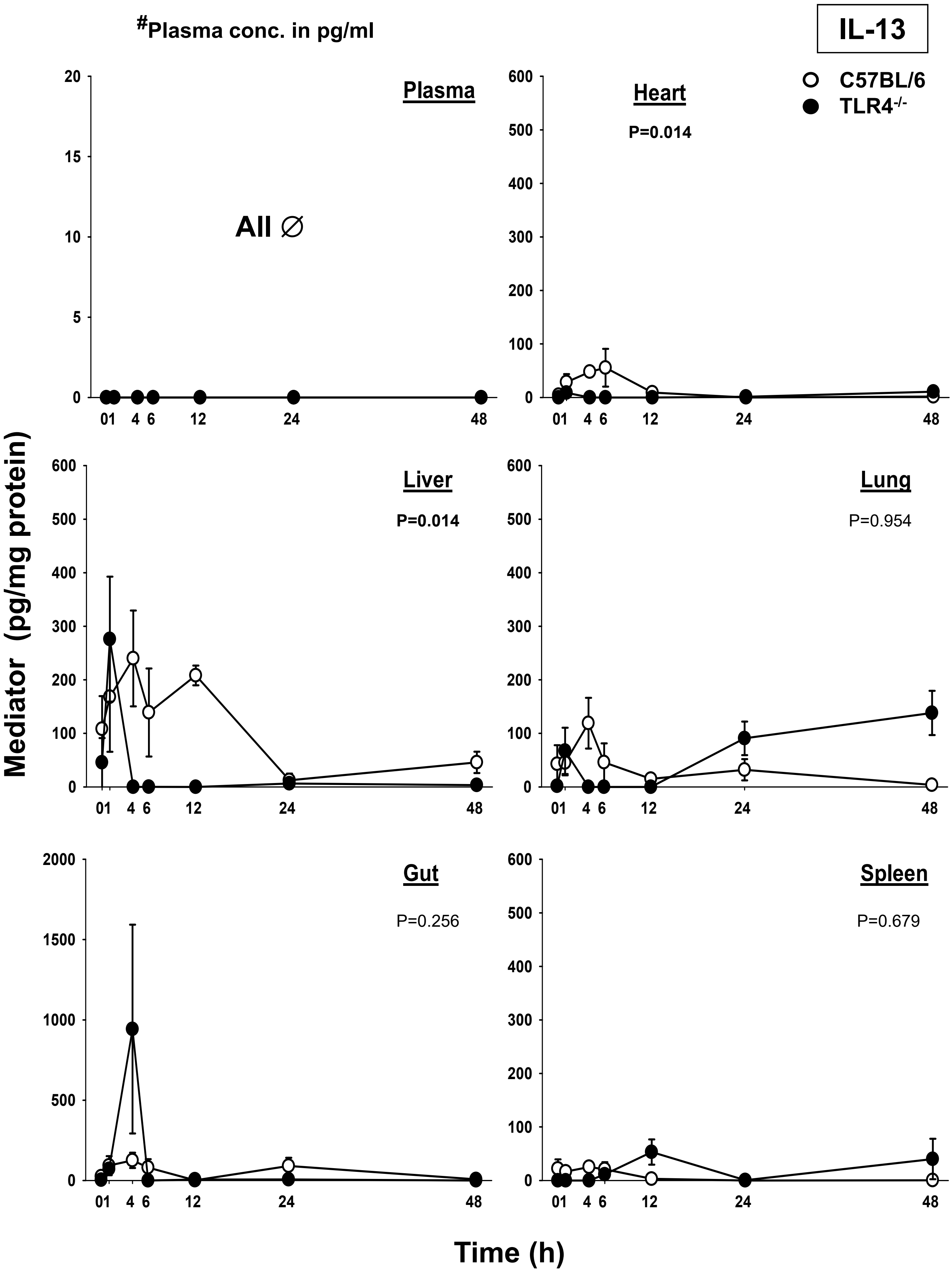

#Plasma conc. in pg/ml
IL-13
C57BL/6
TLR4-/-
Plasma
All ∅
Heart
P=0.014
Liver
P=0.014
Lung
P=0.954
Gut
P=0.256
Spleen
P=0.679
Mediator (pg/mg protein)
Time (h)
0 1 4 6 12 24 48

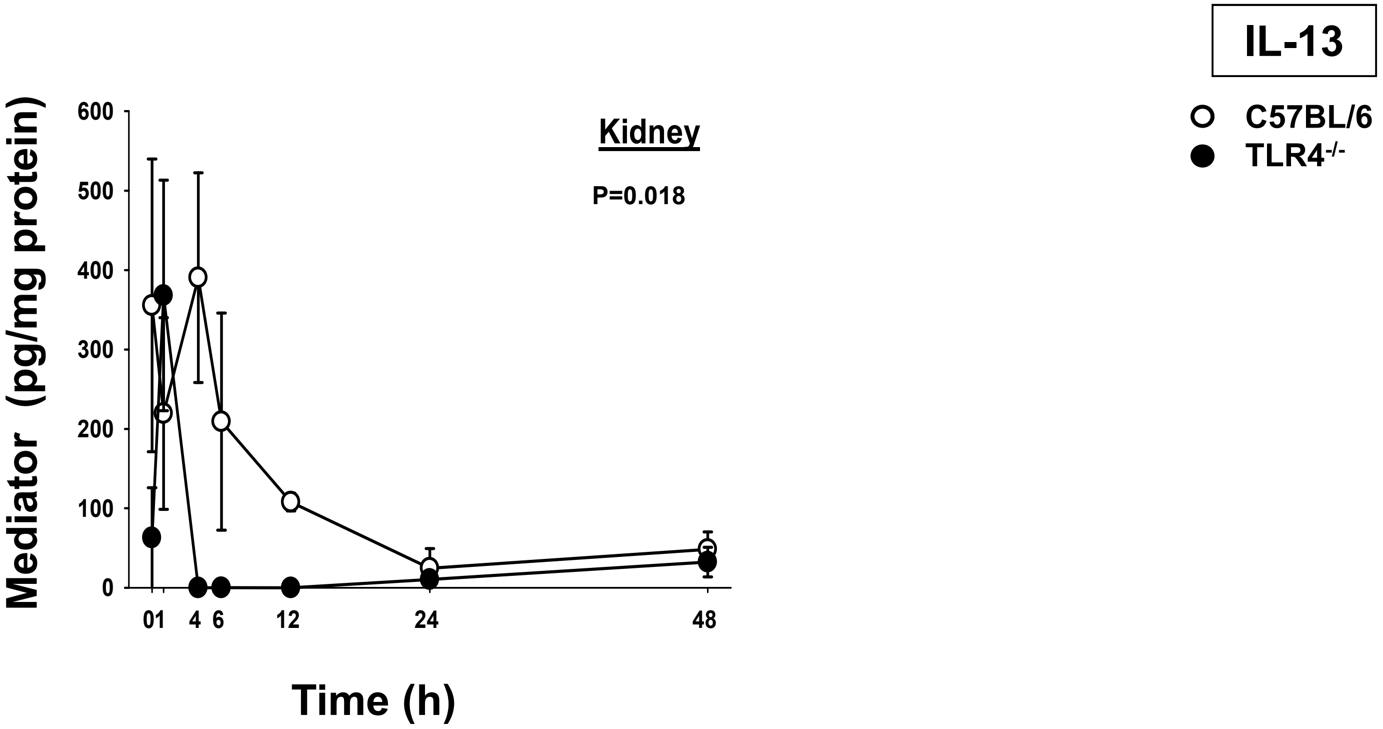
IL-13
C57BL/6
TLR4-/-
Kidney
P=0.018
Mediator (pg/mg protein)
600
500
400
300
200
100
0
0 1 4 6 12 24 48
Time (h)

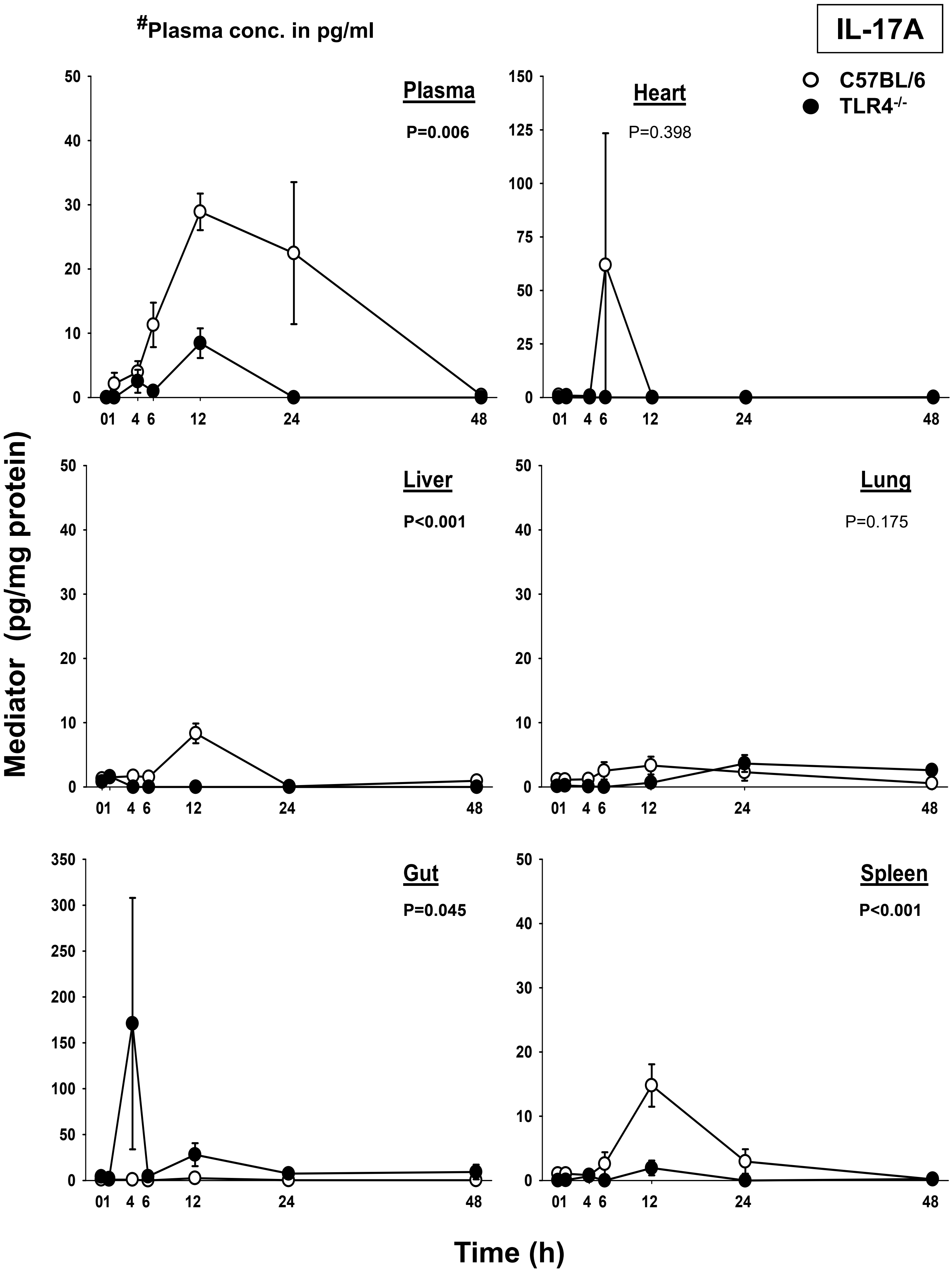

IL-17A
#Plasma conc. in pg/ml
C57BL/6
TLR4-/-
Plasma
P=0.006
Heart
P=0.398
Liver
P<0.001
Lung
P=0.175
Gut
P=0.045
Spleen
P<0.001
Mediator (pg/mg protein)
Time (h)

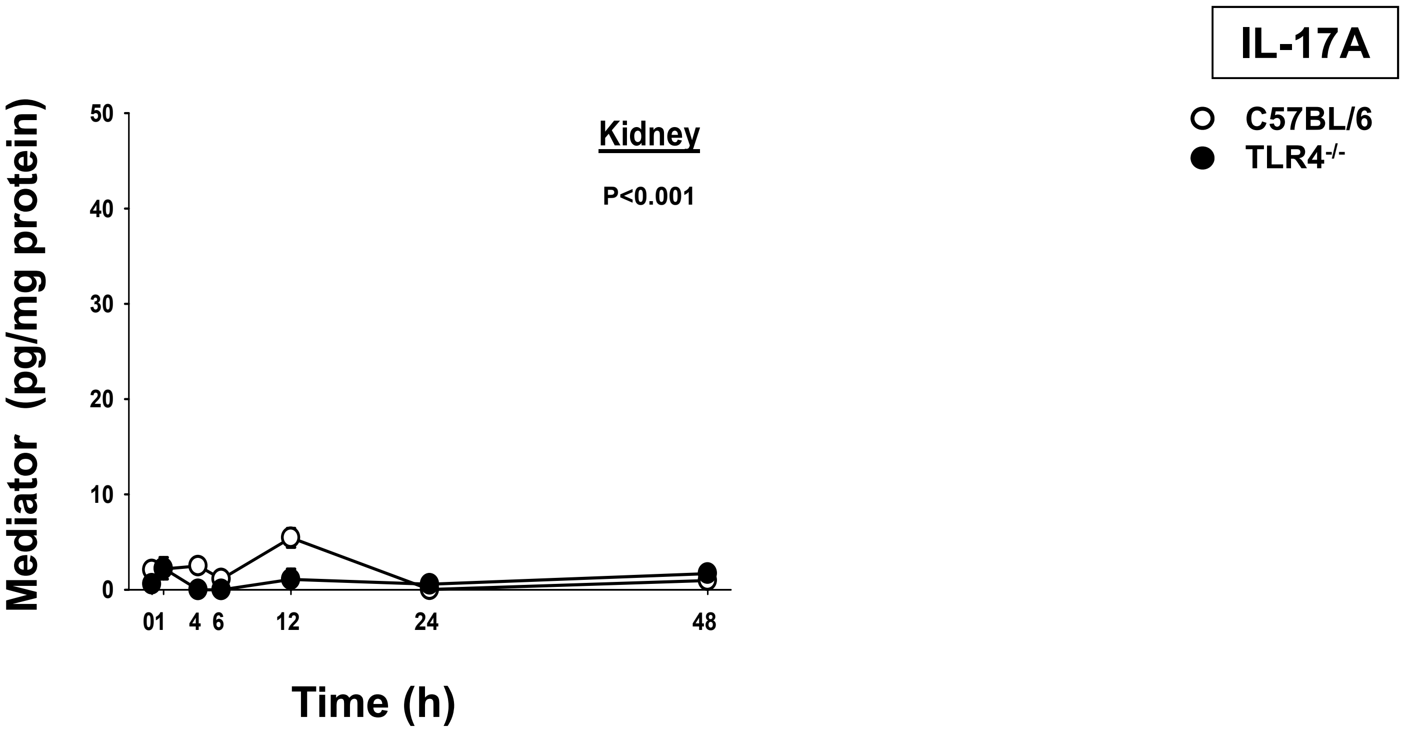

IL-17A
C57BL/6
TLR4-/-
Kidney
P<0.001
Mediator (pg/mg protein)
50
40
30
20
10
0
01
4
6
12
24
48
Time (h)

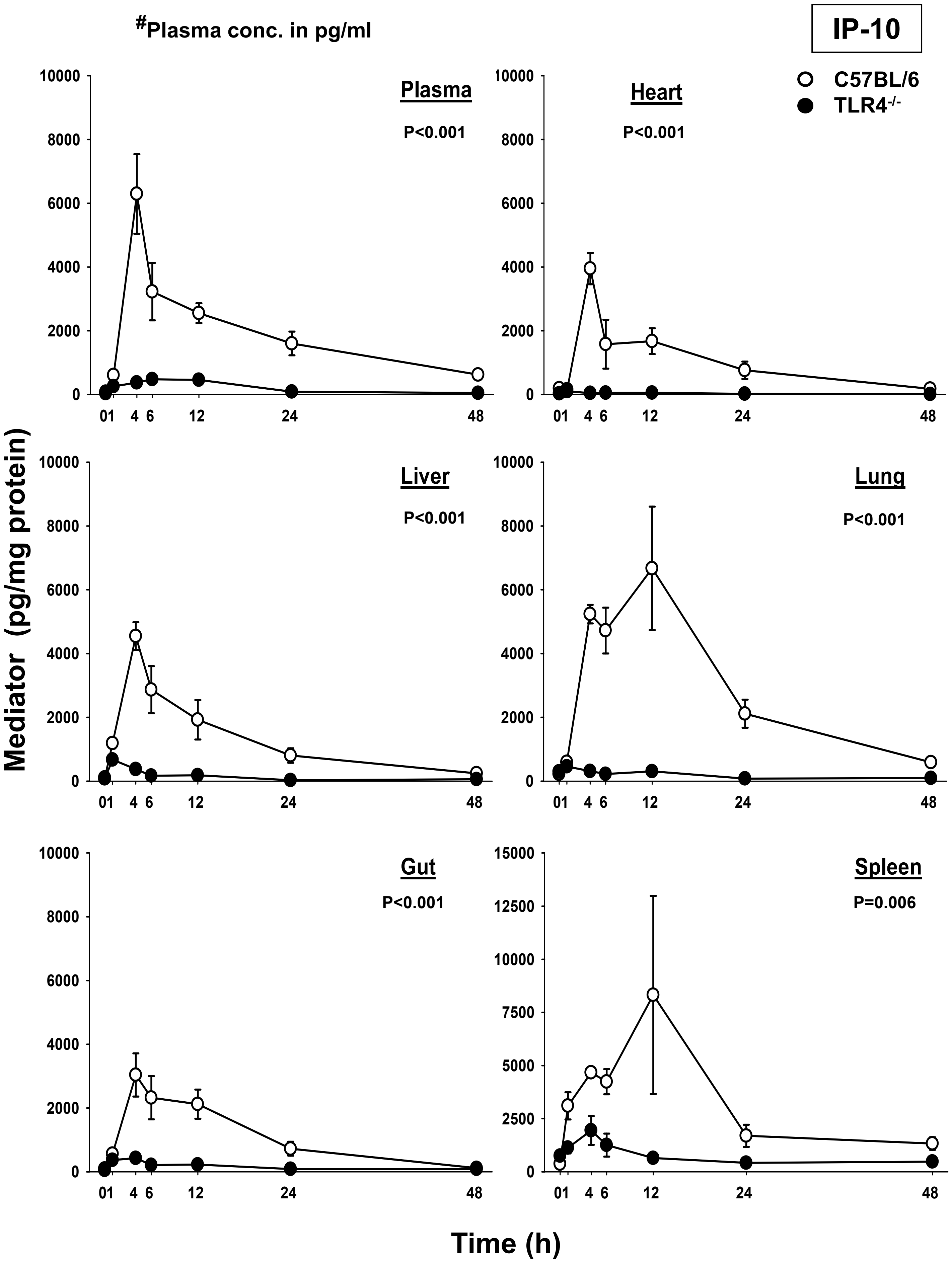

#Plasma conc. in pg/ml
IP-10
C57BL/6
TLR4-/-
Plasma
P<0.001
Heart
P<0.001
Liver
P<0.001
Lung
P<0.001
Gut
P<0.001
Spleen
P=0.006
Mediator (pg/mg protein)
Time (h)

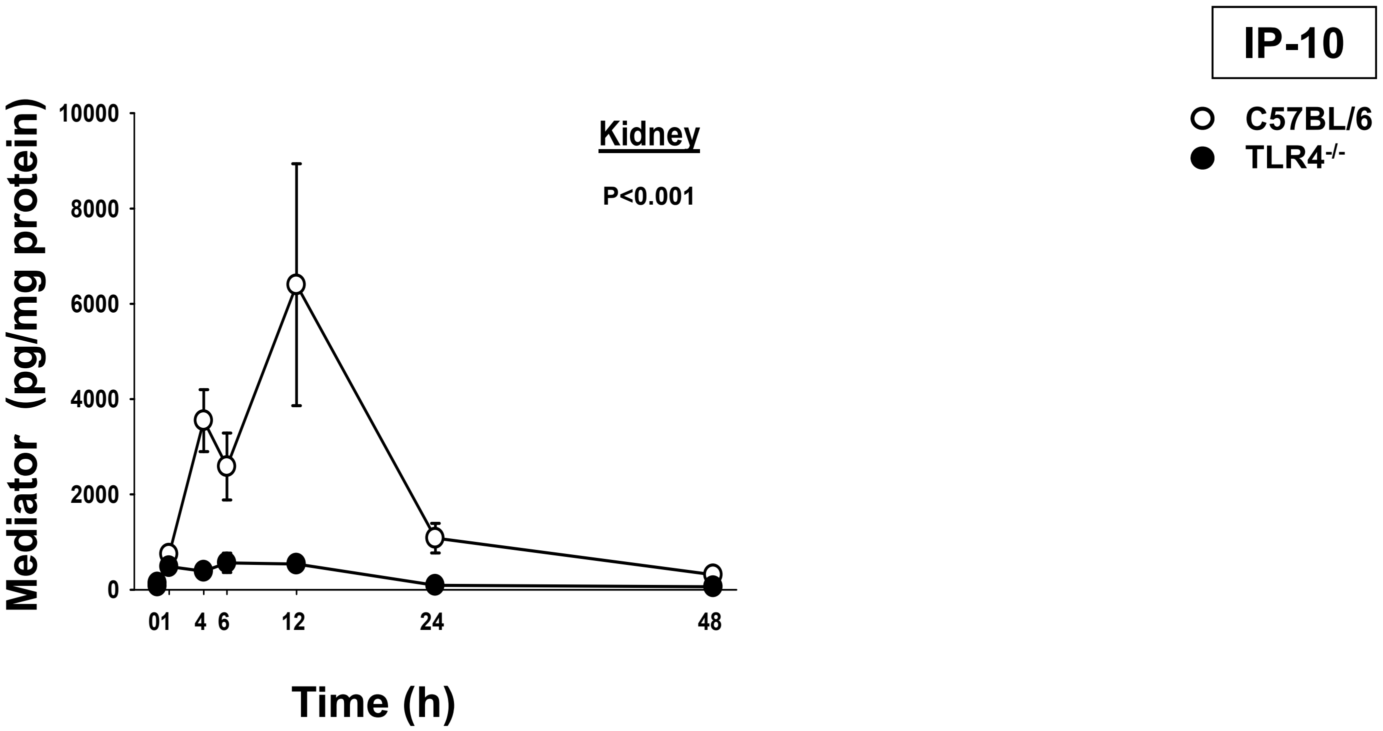
IP-10
C57BL/6
TLR4-/-
Kidney
P<0.001
Mediator (pg/mg protein)
10000
8000
6000
4000
2000
0
0 1 4 6 12 24 48
Time (h)

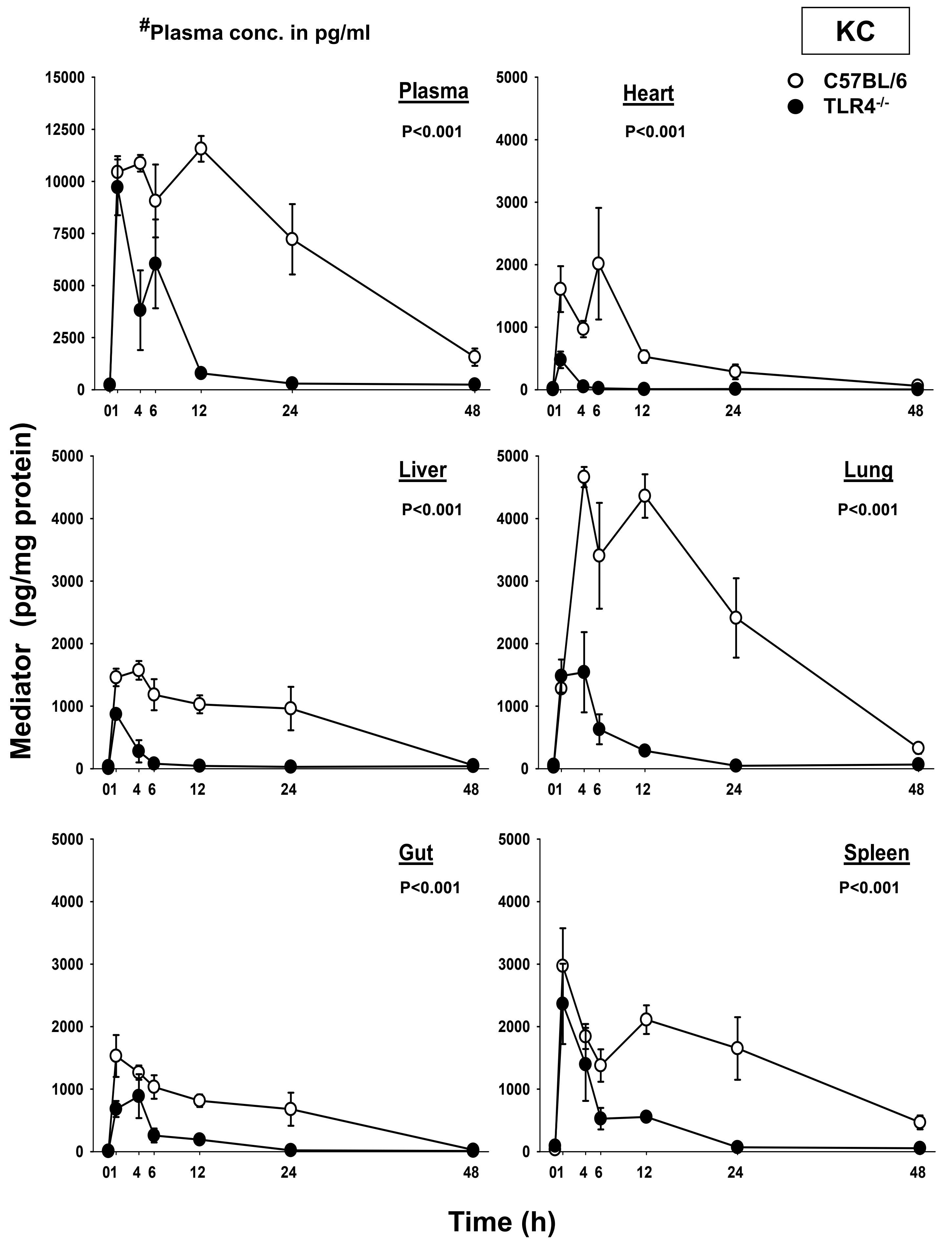

#Plasma conc. in pg/ml
KC
C57BL/6
TLR4-/-
Plasma
P<0.001
Heart
P<0.001
Liver
P<0.001
Lung
P<0.001
Gut
P<0.001
Spleen
P<0.001
Mediator (pg/mg protein)
Time (h)
15000
12500
10000
7500
5000
2500
0
4000
3000
2000
1000
01
4 6
12
24
48

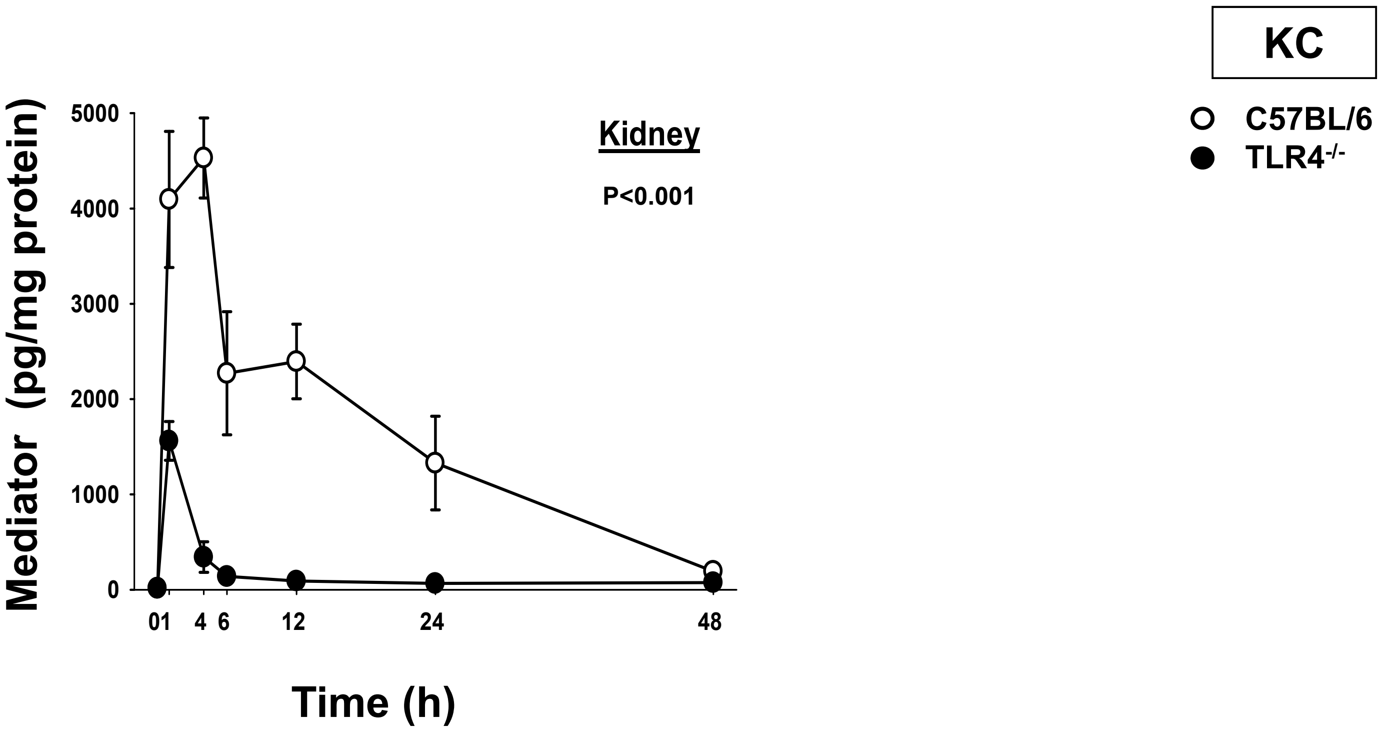
KC
C57BL/6
TLR4-/-
Kidney
P<0.001
Mediator (pg/mg protein)
5000
4000
3000
2000
1000
0
0 1
4 6
12
24
48
Time (h)

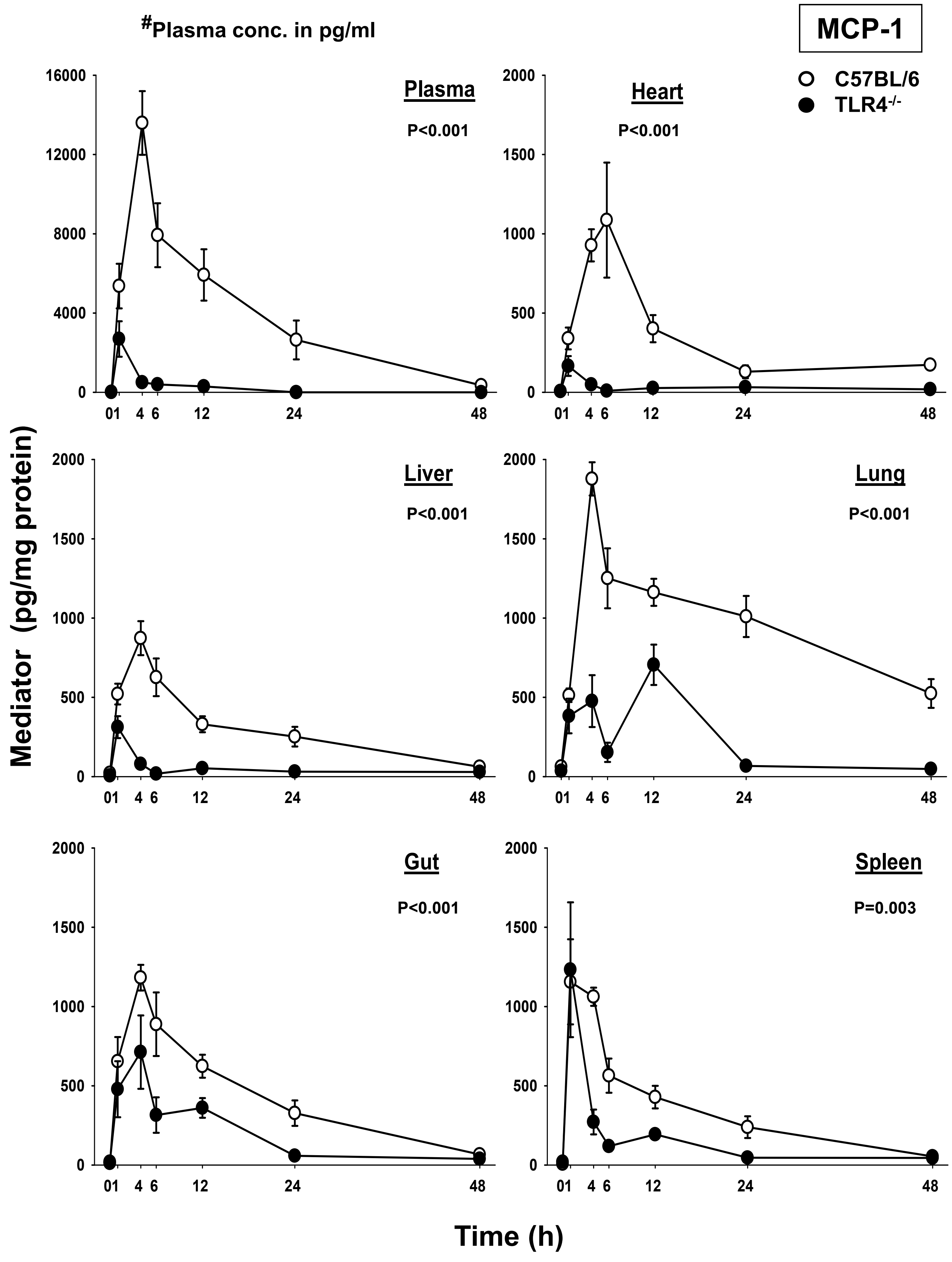

MCP-1
#Plasma conc. in pg/ml
C57BL/6
TLR4-/-
Plasma
P<0.001
Heart
P<0.001
Liver
P<0.001
Lung
P<0.001
Gut
P<0.001
Spleen
P=0.003
Mediator (pg/mg protein)
Time (h)

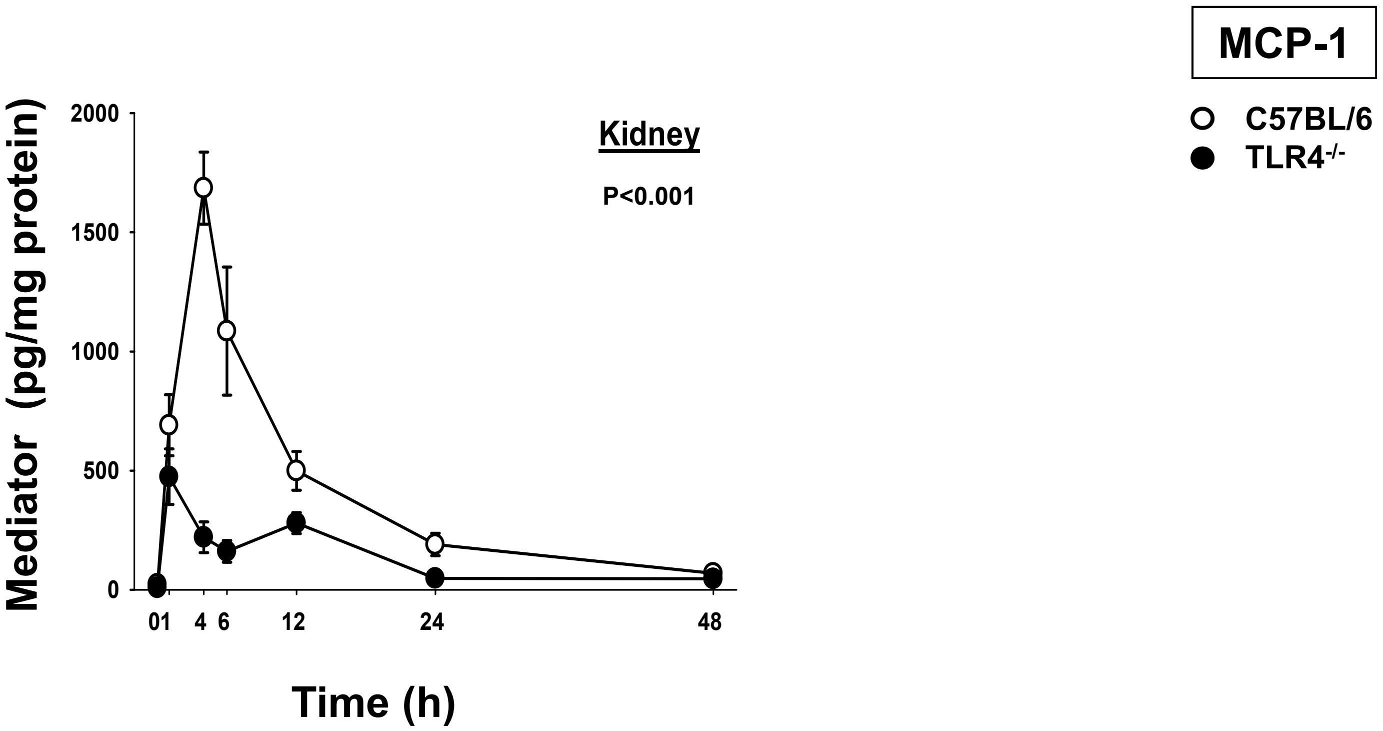

MCP-1
C57BL/6
TLR4-/-
Kidney
P<0.001
Mediator (pg/mg protein)
2000
1500
1000
500
0
0
1
4
6
12
24
48
Time (h)

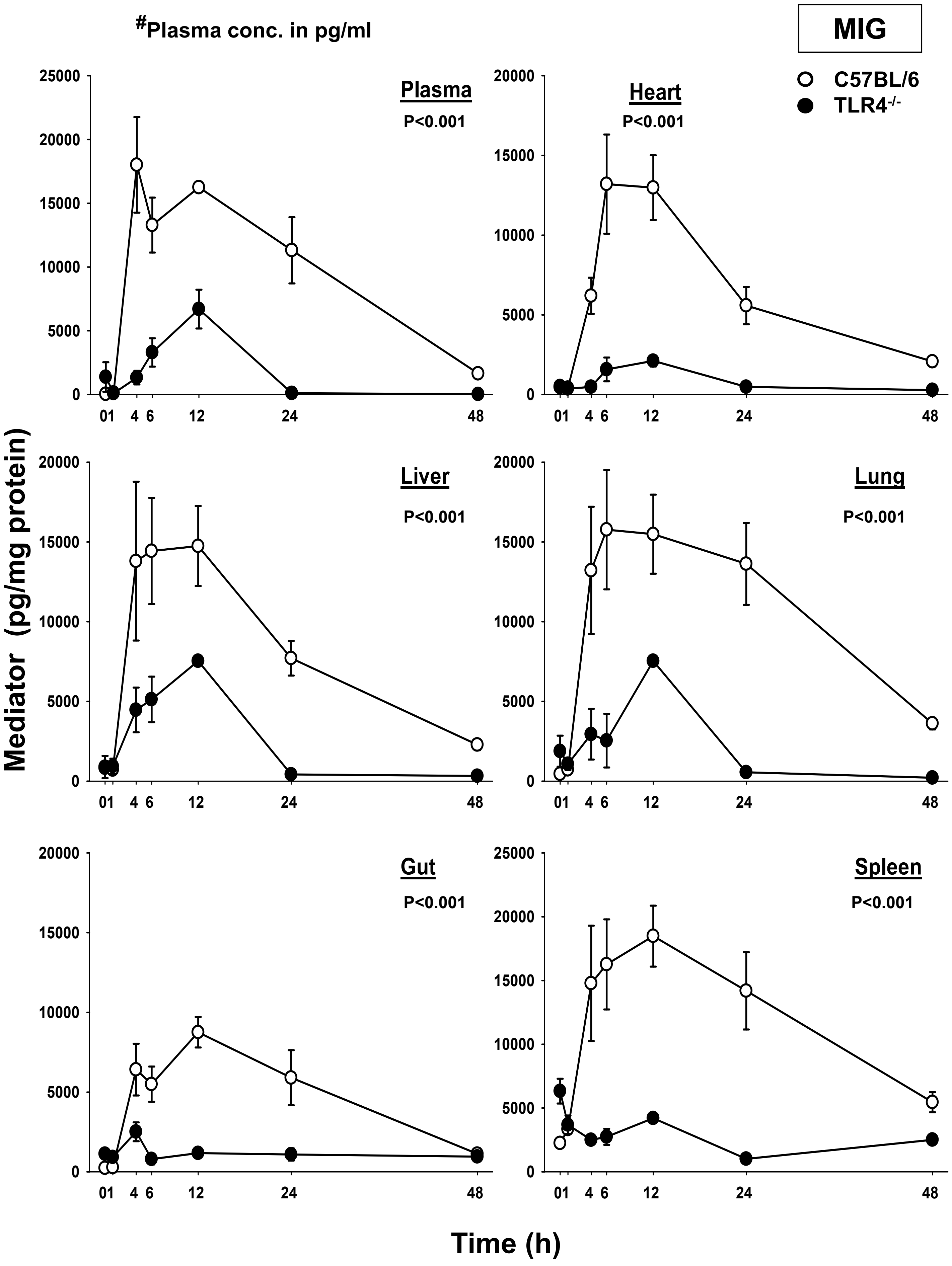

#Plasma conc. in pg/ml
MIG
C57BL/6
TLR4-/-
Plasma
Heart
Liver
Lung
Gut
Spleen
P<0.001
Mediator (pg/mg protein)
Time (h)

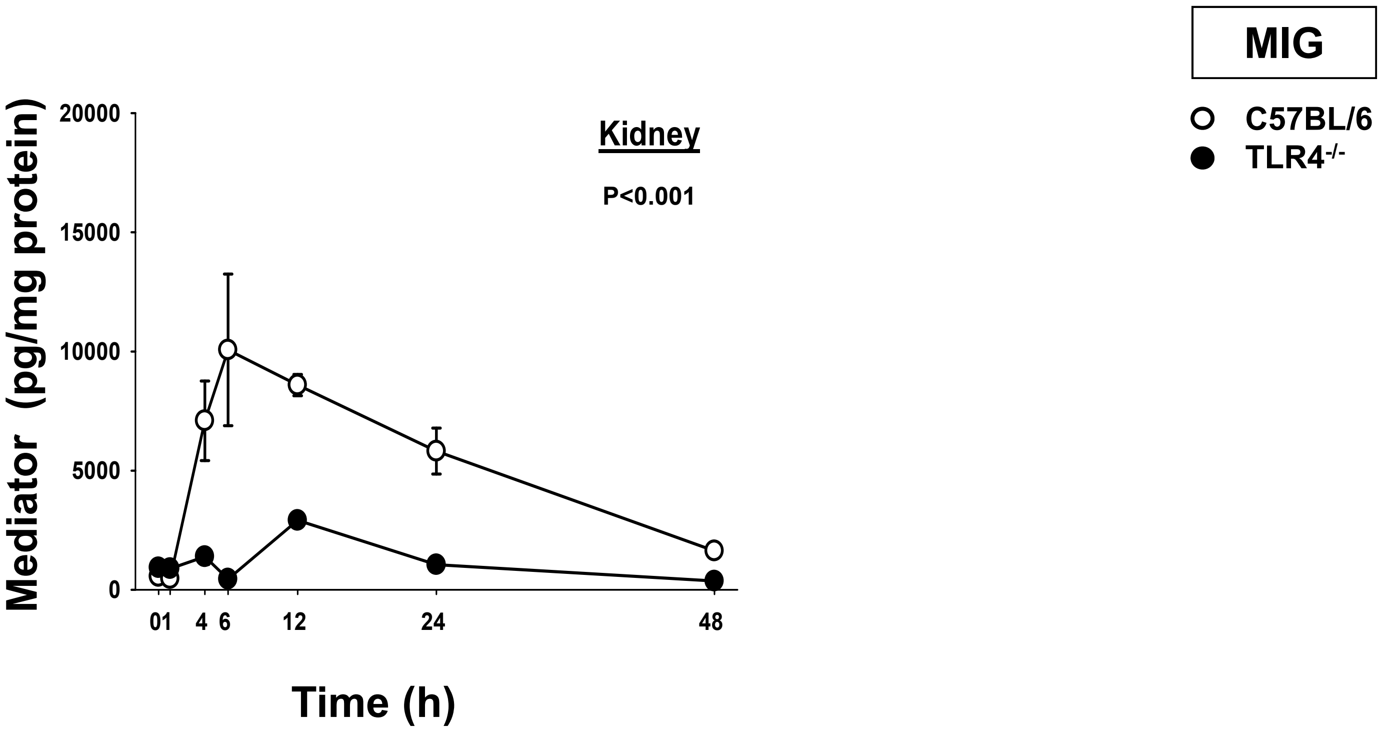

MIG
C57BL/6
TLR4-/-
Kidney
P<0.001
Mediator (pg/mg protein)
20000
15000
10000
5000
0
01
4
6
12
24
48
Time (h)

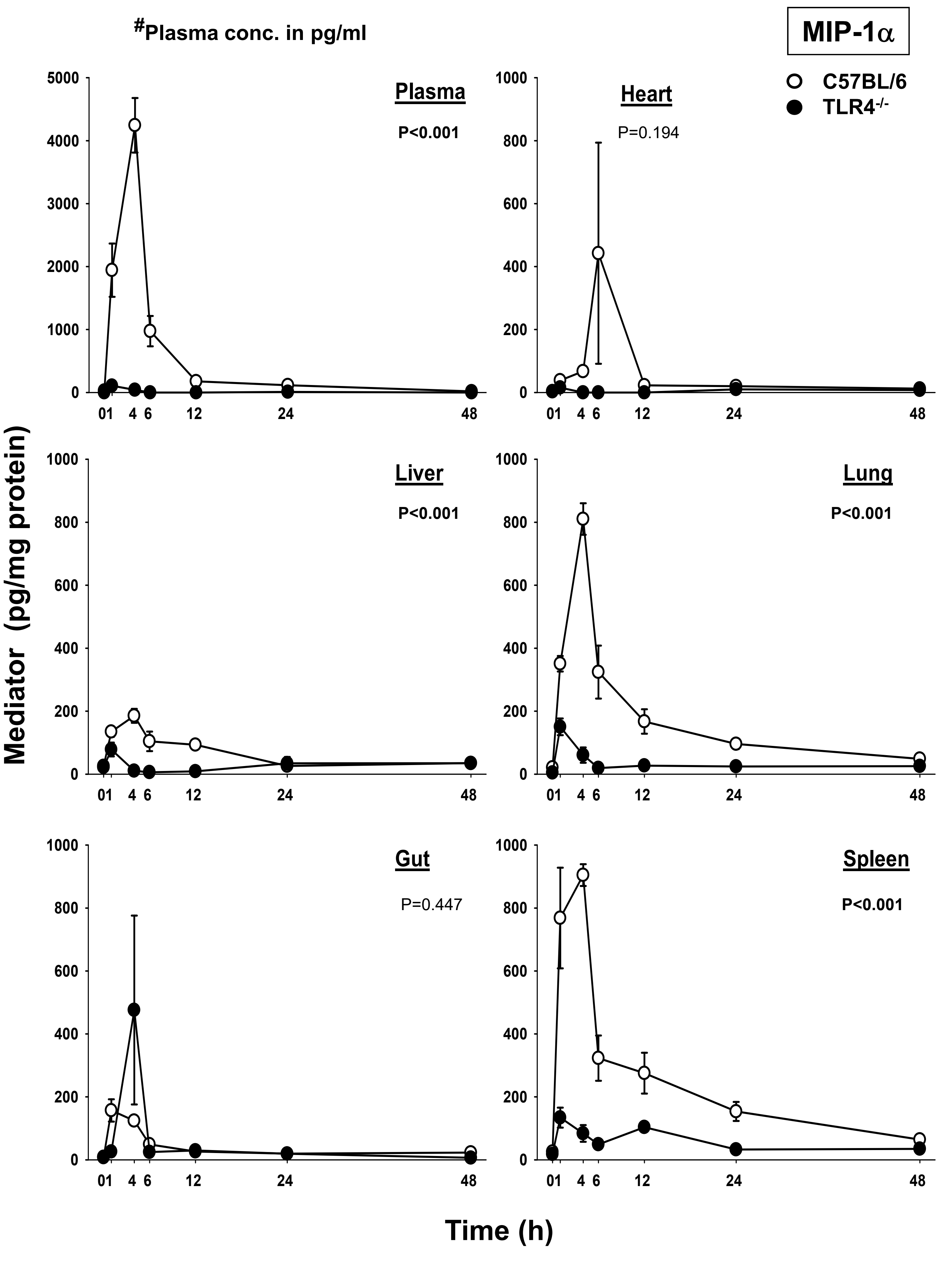

#Plasma conc. in pg/ml
MIP-1α
C57BL/6
TLR4-/-
Plasma
P<0.001
Heart
P=0.194
Liver
P<0.001
Lung
P<0.001
Gut
P=0.447
Spleen
P<0.001
Mediator (pg/mg protein)
Time (h)

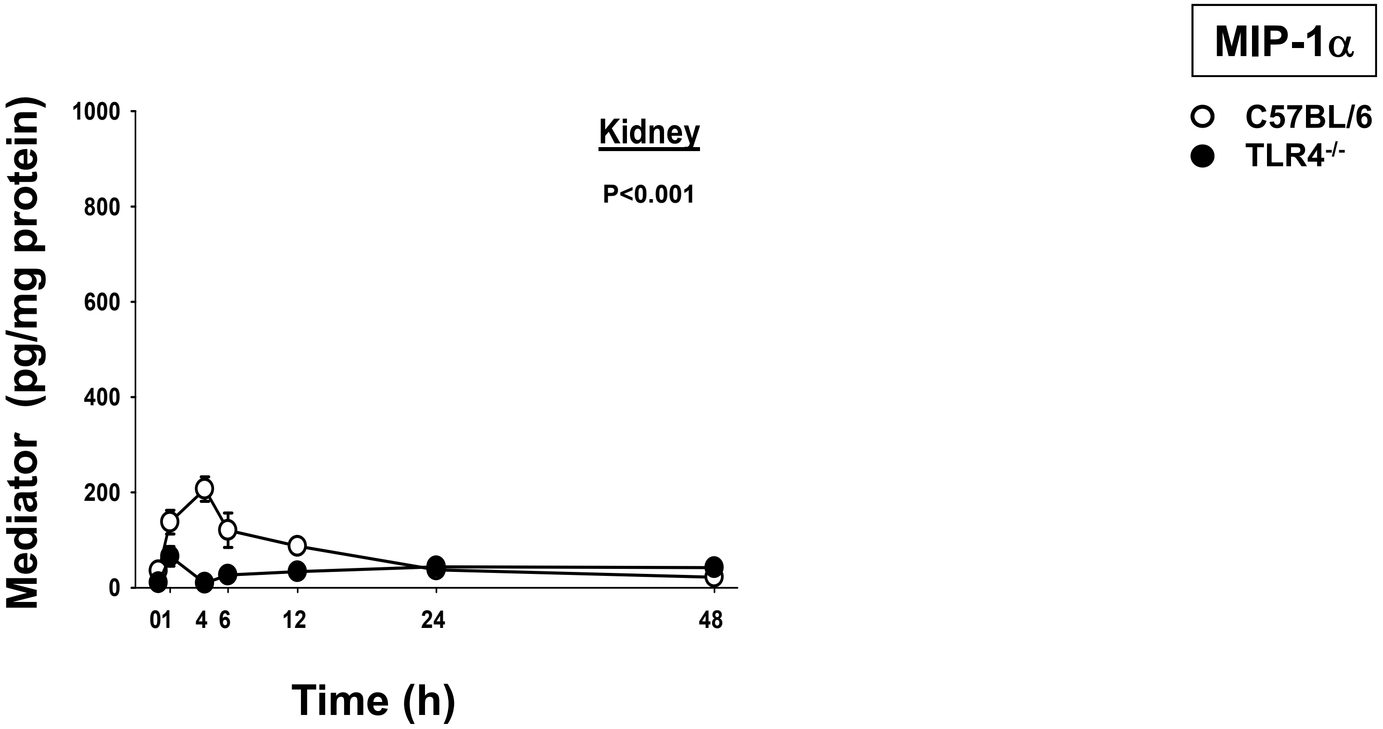
MIP-1α
C57BL/6
TLR4-/-
Kidney
P<0.001
Mediator (pg/mg protein)
1000
800
600
400
200
0
0 1 4 6 12 24 48
Time (h)

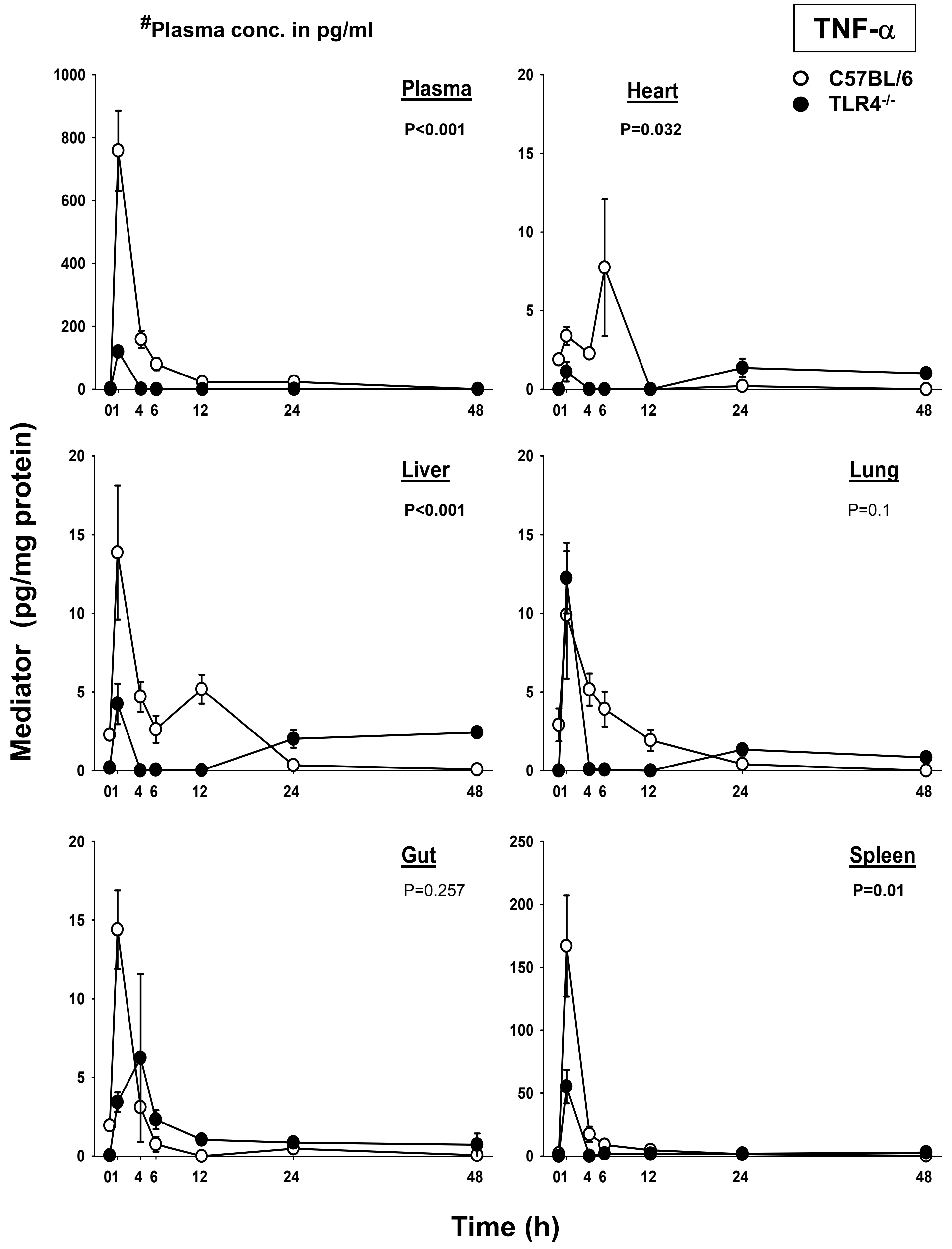

#Plasma conc. in pg/ml
TNF-α
C57BL/6
TLR4-/-
Plasma
P<0.001
Heart
P=0.032
Liver
P<0.001
Lung
P=0.1
Gut
P=0.257
Spleen
P=0.01
Mediator (pg/mg protein)
Time (h)

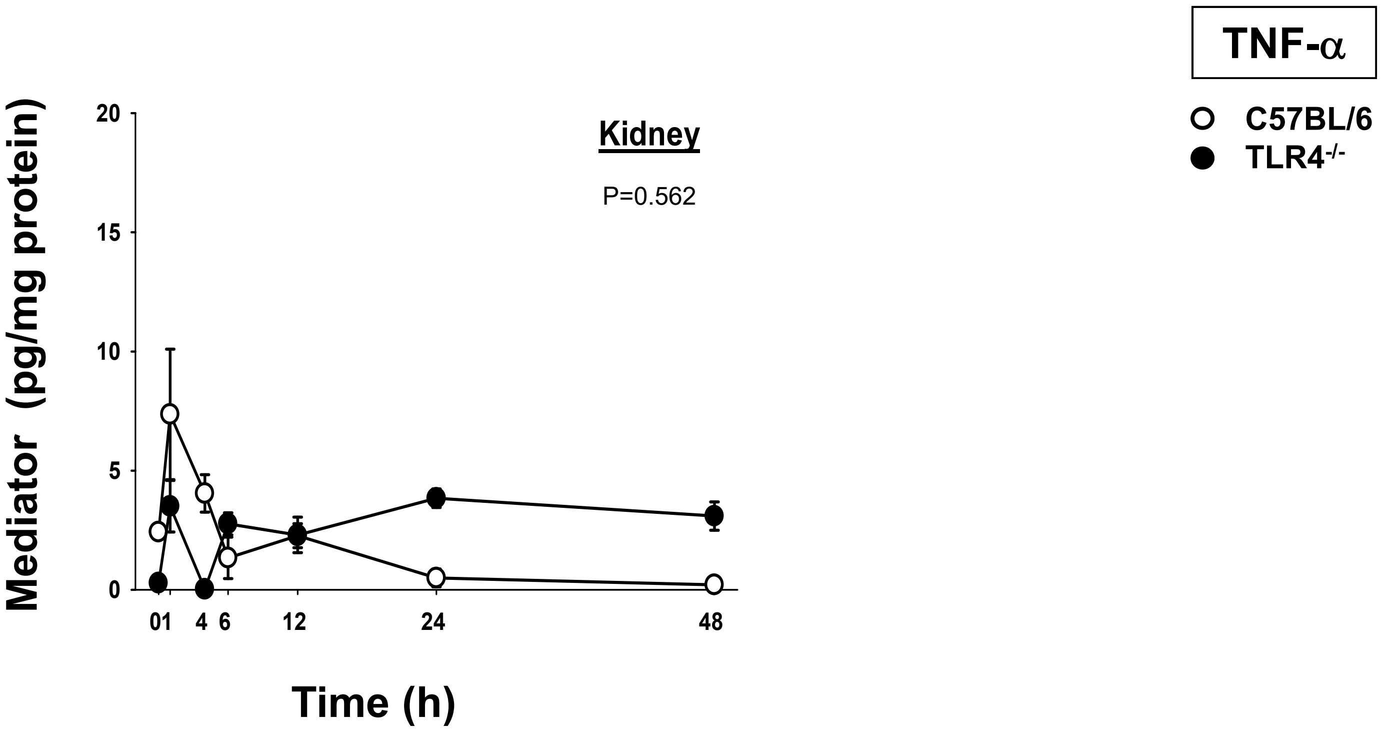

TNF-α
C57BL/6
TLR4-/-
Kidney
P=0.562
Mediator (pg/mg protein)
20
15
10
5
0
0 1 4 6 12 24 48
Time (h)

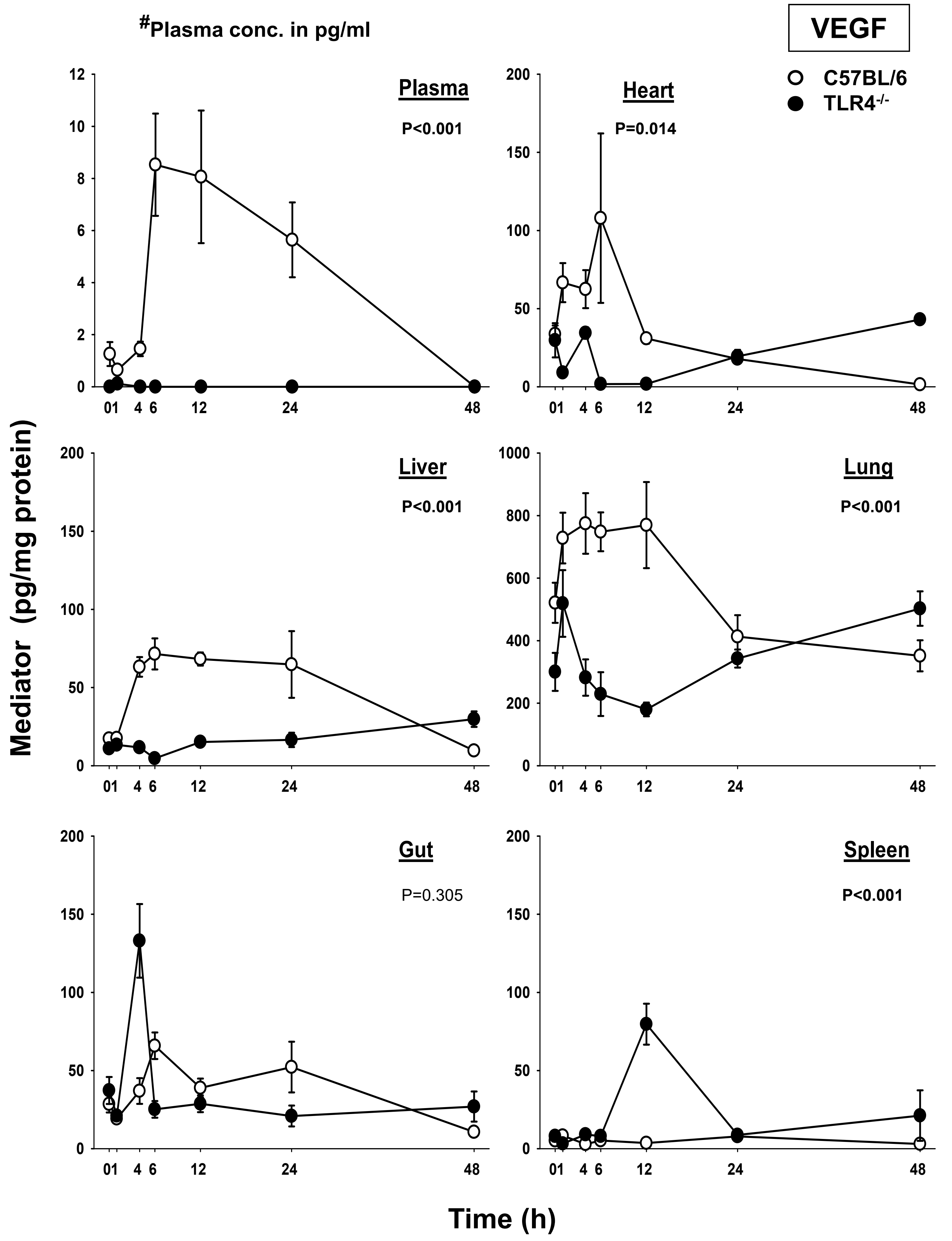

#Plasma conc. in pg/ml
VEGF
C57BL/6
TLR4-/-
Mediator (pg/mg protein)
Plasma
P<0.001
Heart
P=0.014
Liver
P<0.001
Lung
P<0.001
Gut
P=0.305
Spleen
P<0.001
Time (h)

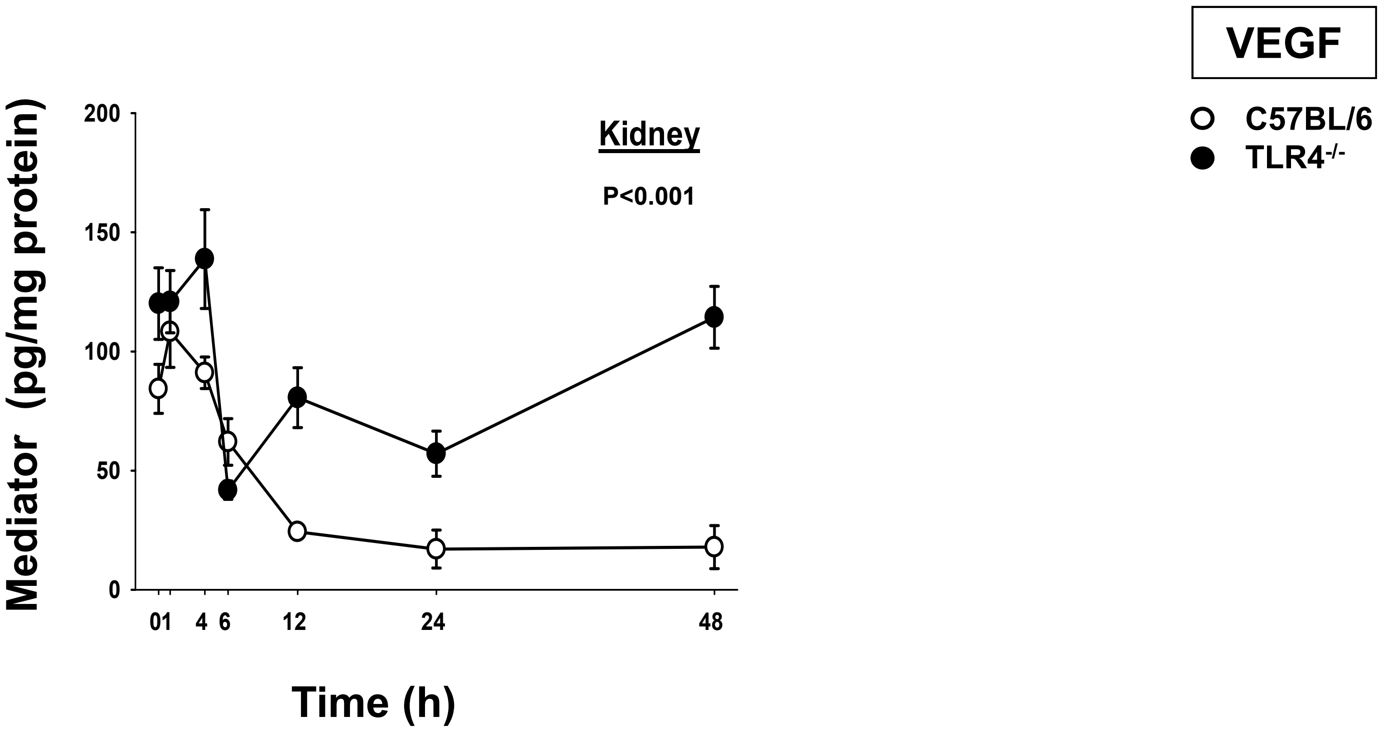
VEGF
C57BL/6
TLR4-/-
Kidney
P<0.001
Mediator (pg/mg protein)
200
150
100
50
0
01
4
6
12
24
48
Time (h)
