# Supplementary figures and images for: A computational analysis of dynamic, multi-organ inflammatory crosstalk induced by endotoxin in mice
Source: PLoS Comput Biol. 2018 Nov 6;14(11):e1006582. doi: 10.1371/journal.pcbi.1006582 (PMC6239343; doi:10.1371/journal.pcbi.1006582)

S2 Fig.

C57BL/6

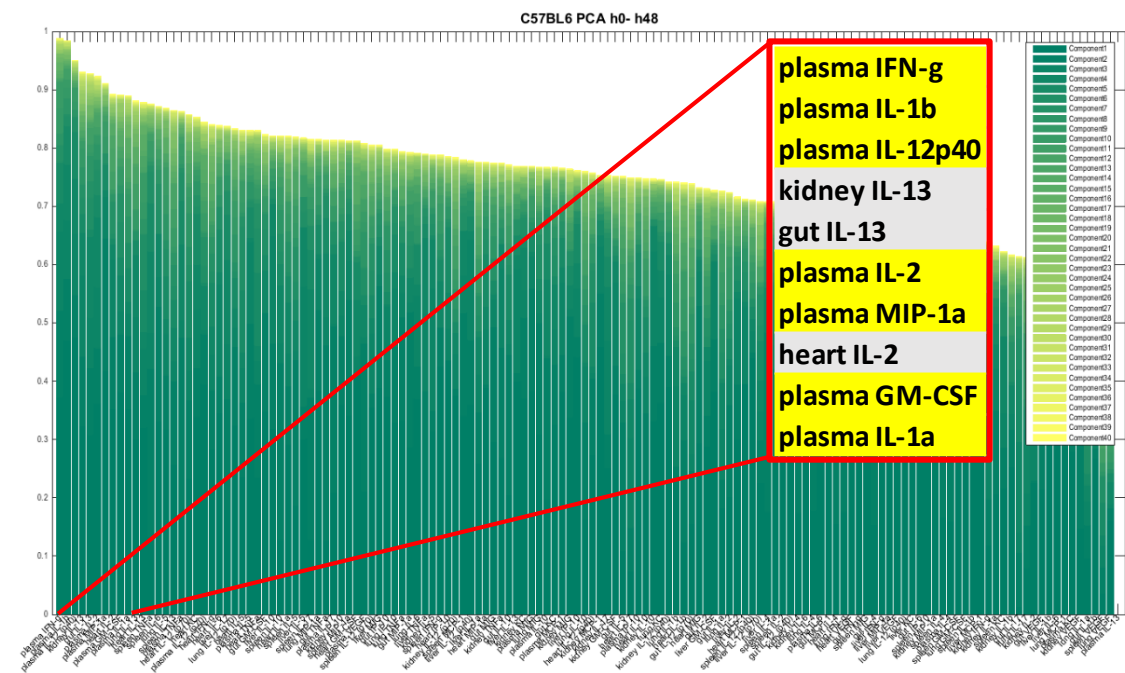

TLR4<sup>-/-</sup>

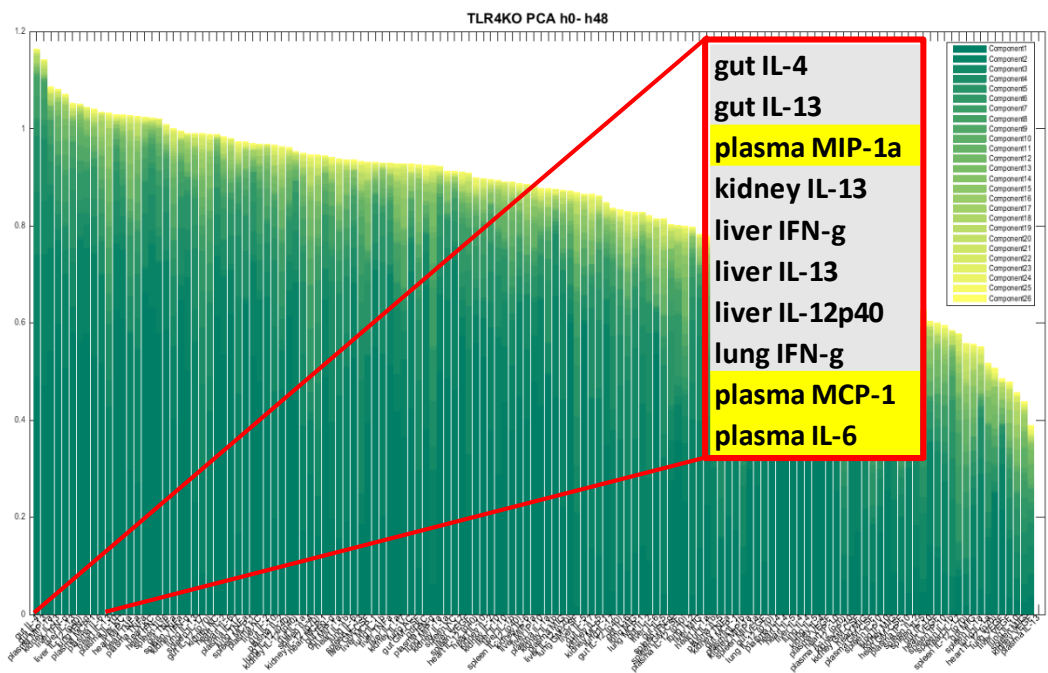

Supplement: S2 Fig — C57BL/6 (n = 5–8 for each experimental group) and TLR4-/- (n = 4 for each experimental group) mice were injected with LPS (3 mg/kg, i.p.). At different time points (0, 1, 4, 6, 12, 24 and 48 h) upon sacrifice, the inflammatory mediators in blood and different organs (liver, heart, gut, lung, spleen and kidney) were measured by Luminex and PCA analysis during the entire time-course (0-48h) was performed as described in Materials and Methods. (PDF) [file pcbi.1006582.s002.pdf]
