# Supplementary material for: A computational analysis of dynamic, multi-organ inflammatory crosstalk induced by endotoxin in mice
Source: PLoS Comput Biol. 2018 Nov 6;14(11):e1006582. doi: 10.1371/journal.pcbi.1006582 (PMC6239343; doi:10.1371/journal.pcbi.1006582)
Supplement: S1 Luminex Data — (PDF) [file pcbi.1006582.s003.pdf]

Data for Comp Analysis incl. AUC

Analysis	Plasma	Time Point [h]	Plasma lasma GM-C	Plasma lasma IFN-	Plasma plasma IL-1	Plasma plasma IL-1	Plasma plasma IL-2	Plasma plasma IL-4	Plasma plasma IL-5	Plasma plasma IL-6
		pg/ml								
	WT-1	h0	33.10	0.00	0.00	0.00	0.00	2.54	0.00	0.89
	WT-1	h1	0.00	0.00	0.00	0.00	0.00	2.27	1.43	8289.65
	WT-1	h4	80.68	18.99	0.00	49.70	0.00	2.50	46.60	10565.46
	WT-1	h6	86.52	2220.65	15.70	61.74	4.41	2.04	90.40	10000.00
	WT-1	h12	24.94	238.43	14.89	0.00	0.00	0.00	66.93	15521.46
	WT-1	h24	0.00	164.25	2.37	0.00	4.32	2.06	25.17	9970.00
	WT-1	h48	13.59	0.00	0.00	0.00	0.00	0.00	0.00	43.38
	WT-2	h0	80.68	0.00	53.05	0.00	0.00	2.24	0.00	0.04
	WT-2	h1	76.48	0.00	0.00	0.00	0.00	3.33	8.76	10441.23
	WT-2	h4	42.42	33.15	0.00	68.61	0.00	2.35	43.29	10450.06
	WT-2	h6	99.63	2100.74	9.60	34.20	5.32	1.92	57.68	10000.00
	WT-2	h12	12.80	107.85	13.09	0.00	0.00	0.00	35.16	14591.52
	WT-2	h24	0.00	0.00	1.34	0.00	4.24	1.81	0.00	3.04
	WT-2	h48	5.67	0.00	0.00	0.00	0.00	0.00	0.00	12.22
	WT-3	h0	19.21	0.00	0.00	0.00	0.00	2.31	0.00	7.03
	WT-3	h1	49.91	0.00	0.00	38.41	0.00	4.57	12.72	10054.96
	WT-3	h4	90.25	56.33	0.00	99.47	0.00	2.52	30.83	10872.13
	WT-3	h6	50.28	1162.62	56.85	49.85	0.00	0.00	100.96	17054.50
	WT-3	h12	57.07	166.93	14.22	0.00	0.00	0.00	42.96	15544.02
	WT-3	h24	0.00	0.00	0.00	0.00	0.00	0.00	13.41	179.89
	WT-3	h48	0.00	0.00	0.00	0.00	0.00	0.00	0.00	14.04
	WT-4	h0	116.67	21.17	0.00	25.63	0.00	3.70	0.00	0.00
	WT-4	h1	0.00	0.00	0.00	14.11	0.00	3.13	18.80	9552.77
	WT-4	h4	95.54	221.26	24.20	224.01	4.75	2.14	26.52	10000.00
	WT-4	h6	83.41	856.11	22.51	36.73	0.00	0.00	161.13	17454.67
	WT-4	h12	24.94	114.84	10.75	1.08	0.00	0.00	31.20	13832.89

WT-4	h24	0.00	0.00	0.00	0.00	0.00	0.00	13.99	174.93
WT-4	h48	0.00	0.00	0.00	0.00	0.00	0.00	0.00	58.84
WT-5	h0	0.00	0.00	1.40	0.00	2.14	0.98	0.46	0.68
WT-5	h1	0.00	0.00	2.58	0.00	4.32	1.92	1.83	4757.47
WT-5	h4	103.50	240.83	19.96	166.78	4.45	2.37	37.48	10000.00
WT-5	h6	0.00	0.00	0.00	0.00	0.00	0.00	33.92	40.41
WT-5	h12	0.00	179.72	4.05	16.48	0.00	0.00	15.98	12592.67
WT-5	h24	0.00	290.39	12.63	0.00	0.00	0.00	50.40	19139.06
WT-5	h48	0.00	0.00	0.00	0.00	0.00	0.00	4.00	2.47
WT-6	h1	0.00	0.00	1.71	0.00	4.37	1.93	0.00	10000.00
WT-6	h4	166.00	479.58	11.88	134.52	4.75	2.35	35.21	10000.00
WT-6	h6	0.00	875.02	13.77	65.65	0.00	0.00	59.25	16583.62
WT-6	h12	0.00	255.49	0.00	18.05	0.00	0.00	16.81	14192.02
**WT-6**	**h24**	**0.00**	**105.44**	**2.51**	**0.00**	**0.00**	**1.64**	**28.95**	**10000.00**
**WT-6**	**h48**	**0.00**	**0.00**	**0.00**	**0.00**	**0.00**	**0.00**	**0.00**	**35.51**

Data for Comp Analysis DyNA with BL-CTRL

			**pg/ml**	**Plasma**	**Plasma**	**Plasma**	**Plasma**	**Plasma**	**Plasma**	**Plasma**	**Plasma**
**Analysis**		**Plasma**	**Time Point [h]**	**pGM-CSF**	**pIFN-g**	**pIL-1a**	**pIL-1b**	**pIL-2**	**pIL-4**	**pIL-5**	**pIL-6**
P8	OBC	WT-1	0h	33.10	0.00	0.00	0.00	0.00	2.54	0.00	0.89
P9	OBC	WT-2	0h	80.68	0.00	53.05	0.00	0.00	2.24	0.00	0.04
P10	OBC	WT-3	0h	19.21	0.00	0.00	0.00	0.00	2.31	0.00	7.03
P11	OBC	WT-4	0h	116.67	21.17	0.00	25.63	0.00	3.70	0.00	0.00
P28	OBC	WT-5	0h	0.00	0.00	1.40	0.00	2.14	0.98	0.46	0.68
		**Plasma**	**Time Point [h]**	**pGM-CSF**	**pIFN-g**	**pIL-1a**	**pIL-1b**	**pIL-2**	**pIL-4**	**pIL-5**	**pIL-6**
P1	LPS	WT-1	1h	0.00	0.00	0.00	0.00	0.00	2.27	1.43	8289.65
P5	LPS	WT-1	4h	80.68	18.99	0.00	49.70	0.00	2.50	46.60	10565.46

P34	LPS	WT-1	6h	86.52	2220.65	15.70	61.74	4.41	2.04	90.40	10000.00
P40	LPS	WT-1	12h	24.94	238.43	14.89	0.00	0.00	0.00	66.93	15521.46
P12	LPS	WT-1	24h	0.00	164.25	2.37	0.00	4.32	2.06	25.17	9970.00
P20	LPS	WT-1	48h	13.59	0.00	0.00	0.00	0.00	0.00	0.00	43.38
P2	LPS	WT-2	1h	76.48	0.00	0.00	0.00	0.00	3.33	8.76	10441.23
P6	LPS	WT-2	4h	42.42	33.15	0.00	68.61	0.00	2.35	43.29	10450.06
P35	LPS	WT-2	6h	99.63	2100.74	9.60	34.20	5.32	1.92	57.68	10000.00
P41	LPS	WT-2	12h	12.80	107.85	13.09	0.00	0.00	0.00	35.16	14591.52
P13	LPS	WT-2	24h	0.00	0.00	1.34	0.00	4.24	1.81	0.00	3.04
P21	LPS	WT-2	48h	5.67	0.00	0.00	0.00	0.00	0.00	0.00	12.22
P3	LPS	WT-3	1h	49.91	0.00	0.00	38.41	0.00	4.57	12.72	10054.96
P7	LPS	WT-3	4h	90.25	56.33	0.00	99.47	0.00	2.52	30.83	10872.13
P36	LPS	WT-3	6h	50.28	1162.62	56.85	49.85	0.00	0.00	100.96	17054.50
P42	LPS	WT-3	12h	57.07	166.93	14.22	0.00	0.00	0.00	42.96	15544.02
P14	LPS	WT-3	24h	0.00	0.00	0.00	0.00	0.00	0.00	13.41	179.89
P22	LPS	WT-3	48h	0.00	0.00	0.00	0.00	0.00	0.00	0.00	14.04
P4	LPS	WT-4	1h	0.00	0.00	0.00	14.11	0.00	3.13	18.80	9552.77
P31	LPS	WT-4	4h	95.54	221.26	24.20	224.01	4.75	2.14	26.52	10000.00
P37	LPS	WT-4	6h	83.41	856.11	22.51	36.73	0.00	0.00	161.13	17454.67
P43	LPS	WT-4	12h	24.94	114.84	10.75	1.08	0.00	0.00	31.20	13832.89
P15	LPS	WT-4	24h	0.00	0.00	0.00	0.00	0.00	0.00	13.99	174.93
P23	LPS	WT-4	48h	0.00	0.00	0.00	0.00	0.00	0.00	0.00	58.84
P29	LPS	WT-5	1h	0.00	0.00	2.58	0.00	4.32	1.92	1.83	4757.47
P32	LPS	WT-5	4h	103.50	240.83	19.96	166.78	4.45	2.37	37.48	10000.00
P38	LPS	WT-5	6h	0.00	0.00	0.00	0.00	0.00	0.00	33.92	40.41
P44	LPS	WT-5	12h	0.00	179.72	4.05	16.48	0.00	0.00	15.98	12592.67
P16	LPS	WT-5	24h	0.00	290.39	12.63	0.00	0.00	0.00	50.40	19139.06
P24	WT	WT-5	48h	0.00	0.00	0.00	0.00	0.00	0.00	4.00	2.47
P30	LPS	WT-6	1h	0.00	0.00	1.71	0.00	4.37	1.93	0.00	10000.00

P33	LPS	WT-6	4h	166.00	479.58	11.88	134.52	4.75	2.35	35.21	10000.00
P39	LPS	WT-6	6h	0.00	875.02	13.77	65.65	0.00	0.00	59.25	16583.62
P45	LPS	WT-6	12h	0.00	255.49	0.00	18.05	0.00	0.00	16.81	14192.02
Md-P17-18-19	LPS	**WT-6**	24h	**0.00**	**105.44**	**2.51**	**0.00**	**0.00**	**1.64**	**28.95**	**10000.00**
Md-P25-26-27	LPS	**WT-6**	48h	**0.00**	**0.00**	**0.00**	**0.00**	**0.00**	**0.00**	**0.00**	**35.51**

**Data for Comp. Anal. Incl. AUC**				plasma GM-C	lasma IFN-	lasma IL-1a	plasma IL-1b	plasma IL-2	plasma IL-4	plasma IL-5	plasma IL-6
			**pg/ml**	**Plasma**	**Plasma**	**Plasma**	**Plasma**	**Plasma**	**Plasma**	**Plasma**	**Plasma**
**Mouse ID**	**Strain**	**Plasma**	**Time Point [h]**	lasma GM-C	lasma IFN-	lasma IL-1a	plasma IL-1b	plasma IL-2	plasma IL-4	plasma IL-5	plasma IL-6
P46	TLR4 Ko	**KO-1**	**h0**	0.0	0.0	0.0	0.0	0.0	0.0	0.0	0.0
P47	TLR4 Ko	**KO-1**	**h1**	0.0	0.0	0.0	0.0	0.0	0.0	0.0	404.6
P52	TLR4 Ko	**KO-1**	**h4**	0	0.73	0	26.59	0	0	24.37	287.03
P58	TLR4 Ko	**KO-1**	**h6**	0	0	0	0	0	0	9.45	1022.59
P62	TLR4 Ko	**KO-1**	**h12**	0	0	0	0	0	0	15.98	103.48
P66	TLR4 Ko	**KO-1**	**h24**	0	0	0	0	0	0	14.64	0
P70	TLR4 Ko	**KO-1**	**h48**	0	0	0	0	0.97	0	1.05	0
P50	TLR4 Ko	**KO-2**	**h0**	0	0	0	0	0	0	7.72	2.59
P51	TLR4 Ko	**KO-3**	**h0**	0	0	0	0	0	0	0	11.65
P56	TLR4 Ko	**KO-4**	**h0**	0	0	0	5.55	0	0	16.66	4.02
P48	TLR4 Ko	**KO-2**	**h1**	0	0	0.99	0	4.24	1.92	1.53	1861.13
P49	TLR4 Ko	**KO-3**	**h1**	0	0	1.46	0	4.07	1.77	0.91	3359.38
P57	TLR4 Ko	**KO-4**	**h1**	0	0	0	0	0	0	0	2352.84
P53	TLR4 Ko	**KO-2**	**h4**	0	0	0	0	0	0	6.46	159.55
P54	TLR4 Ko	**KO-3**	**h4**	13.62	0	0	0	0	0	0	1.62
P55	TLR4 Ko	**KO-4**	**h4**	0	0	0	0	0	0	1.77	1070.69
P59	TLR4 Ko	**KO-2**	**h6**	0	3.06	0	0	0	0	1.57	693.57
P60	TLR4 Ko	**KO-3**	**h6**	0	0	0	0	0	0	0	0

P61	TLR4 Kc	KO-4	h6	22.68	0.88	5.23	0	0	0	19.21	906.64
P63	TLR4 Kc	KO-2	h12	0	0	0	0	0	0	64.78	108.5
P64	TLR4 Kc	KO-3	h12	0	13.72	0	0	0	0	0	214.31
P65	TLR4 Kc	KO-4	h12	0	0	0	0	0	0	33.8	73.45
P67	TLR4 Kc	KO-2	h24	52.72	0	0	5.07	0.88	0	45.23	0
P68	TLR4 Kc	KO-3	h24	52.72	0	0	0	0	0	21.28	6.03
P69	TLR4 Kc	KO-4	h24	0	0	0	0	0	0	21.28	0
P71	TLR4 Kc	KO-2	h48	0	0	0	0	0	0	0	0
P72	TLR4 Kc	KO-3	h48	0	0	0	0	0.43	0	0	0

Data for DyNA with CTRL-BL

			pg/ml	Plasma	Plasma	Plasma	Plasma	Plasma	Plasma	Plasma	Plasma
**Mouse ID**	**Strain**	**Plasma**	**Time Point [h]**	**pGM-CSF**	**pIFN-g**	**pIL-1a**	**pIL-1b**	**pIL-2**	**pIL-4**	**pIL-5**	**pIL-6**
P46	TLR4 Kc	KO-1	0h	0.0	0.0	0.0	0.0	0.0	0.0	0.0	0.0
P50	TLR4 Kc	KO-2	0h	0	0	0	0	0	0	7.72	2.59
P51	TLR4 Kc	KO-3	0h	0	0	0	0	0	0	0	11.65
P56	TLR4 Kc	KO-4	0h	0	0	0	5.55	0	0	16.66	4.02

**Mouse ID**	**Strain**	**Plasma**	**Time Point [h]**	**pGM-CSF**	**pIFN-g**	**pIL-1a**	**pIL-1b**	**pIL-2**	**pIL-4**	**pIL-5**	**pIL-6**
P47	TLR4 Kc	KO-1	1h	0.0	0.0	0.0	0.0	0.0	0.0	0.0	404.6
P52	TLR4 Kc	KO-1	4h	0	0.73	0	26.59	0	0	24.37	287.03
P58	TLR4 Kc	KO-1	6h	0	0	0	0	0	0	9.45	1022.59
P62	TLR4 Kc	KO-1	12h	0	0	0	0	0	0	15.98	103.48
P66	TLR4 Kc	KO-1	24h	0	0	0	0	0	0	14.64	0
P70	TLR4 Kc	KO-1	48h	0	0	0	0	0.97	0	1.05	0
P48	TLR4 Kc	KO-2	1h	0	0	0.99	0	4.24	1.92	1.53	1861.13

P49	TLR4 Ko	KO-3	1h	0	0	1.46	0	4.07	1.77	0.91	3359.38
P57	TLR4 Ko	KO-4	1h	0	0	0	0	0	0	0	2352.84
P53	TLR4 Ko	KO-2	4h	0	0	0	0	0	0	6.46	159.55
P54	TLR4 Ko	KO-3	4h	13.62	0	0	0	0	0	0	1.62
P55	TLR4 Ko	KO-4	4h	0	0	0	0	0	0	1.77	1070.69
P59	TLR4 Ko	KO-2	6h	0	3.06	0	0	0	0	1.57	693.57
P60	TLR4 Ko	KO-3	6h	0	0	0	0	0	0	0	0
P61	TLR4 Ko	KO-4	6h	22.68	0.88	5.23	0	0	0	19.21	906.64
P63	TLR4 Ko	KO-2	12h	0	0	0	0	0	0	64.78	108.5
P64	TLR4 Ko	KO-3	12h	0	13.72	0	0	0	0	0	214.31
P65	TLR4 Ko	KO-4	12h	0	0	0	0	0	0	33.8	73.45
P67	TLR4 Ko	KO-2	24h	52.72	0	0	5.07	0.88	0	45.23	0
P68	TLR4 Ko	KO-3	24h	52.72	0	0	0	0	0	21.28	6.03
P69	TLR4 Ko	KO-4	24h	0	0	0	0	0	0	21.28	0
P71	TLR4 Ko	KO-2	48h	0	0	0	0	0	0	0	0
P72	TLR4 Ko	KO-3	48h	0	0	0	0	0.43	0	0	0

Plasma	Plasma	Plasma	Plasma	Plasma	Plasma	Plasma	Plasma	Plasma	Plasma	Plasma
plasma IL-1	lasma IL-12 (p40	asma IL-12 (p7	plasma IL-13	plasma IL-1	plasma IP-10	plasma KC	plasma MCP-1	plasma MIG	asma MIP-	plasma TNF
0.00	0.00	0.00	0.00	0.00	28.99	180.20	0.00	26.23	47.21	2.86
617.84	0.00	0.00	0.00	0.00	375.42	12199.00	873.54	308.86	239.16	203.15
694.48	974.96	0.00	0.00	0.00	3558.15	11711.34	17191.19	9717.53	3992.70	129.28
350.22	829.79	53.65	0.00	11.20	2520.78	10000.00	10000.00	14926.90	1762.42	146.43
202.29	83.78	11.64	0.00	38.56	2564.92	10100.61	4303.74	15530.90	212.37	25.92
401.94	8.96	9.40	0.00	15.35	1904.37	10000.00	8199.87	19431.37	275.35	46.01
32.77	0.00	0.00	0.00	0.16	546.85	2878.21	444.08	1864.35	24.46	0.00
0.00	0.00	0.00	0.00	0.00	44.93	318.72	13.27	23.67	0.00	2.74
1653.71	0.00	37.41	0.00	2.30	857.98	12012.86	6598.28	101.19	2773.06	942.93
508.57	671.90	0.00	0.00	0.00	4450.40	11434.99	17191.19	10827.98	2542.95	80.40
218.11	467.22	36.20	0.00	9.61	2336.88	10000.00	10000.00	12097.17	1406.55	87.66
292.69	43.99	11.42	0.00	31.27	1660.80	10988.10	3676.67	15478.09	138.52	23.34
0.00	0.00	35.13	0.00	0.00	202.74	256.64	0.00	1179.60	0.00	0.00
15.79	0.00	0.00	0.00	0.11	318.49	480.99	122.27	690.32	52.76	0.00
0.00	0.00	0.00	0.00	0.00	50.89	345.61	0.00	49.05	0.00	2.74
1379.79	0.00	90.33	0.00	10.46	778.45	7122.80	6822.84	37.98	2652.74	624.63
466.15	1181.45	32.54	0.00	3.20	6373.62	12074.42	17191.19	8942.86	5577.30	89.74
379.24	301.45	21.61	0.00	25.58	4386.00	11718.72	10000.00	17930.85	957.24	70.91
272.56	71.75	16.44	0.00	31.19	2140.49	11130.69	4057.27	16490.13	145.88	23.34
218.84	0.00	8.54	0.00	0.00	971.30	2324.59	550.76	8020.03	0.00	7.02
38.24	0.00	4.52	0.00	0.54	606.26	2113.18	290.68	1558.66	16.05	0.00
0.00	0.00	30.33	0.00	0.09	37.39	150.71	18.93	64.62	92.31	4.14
1724.09	0.00	20.47	0.00	0.00	576.01	11323.56	5934.19	38.34	2189.76	771.81
570.51	756.61	90.39	0.00	5.58	10000.00	10000.00	10000.00	22722.98	3893.09	187.89
492.79	258.90	20.75	0.00	7.10	6958.22	11510.34	10000.00	15390.33	739.73	84.69
339.20	43.99	9.88	0.00	22.12	2083.05	10278.78	3499.14	17320.60	162.74	24.77

298.04	0.00	8.99	0.00	0.00	782.67	3636.04	1013.10	4423.07	60.12	1.48
100.30	0.00	0.00	0.00	0.38	1260.05	3094.63	604.20	2573.29	29.03	2.31

0.48	0.43	0.00	0.00	0.00	14.66	154.78	0.00	64.99	0.00	0.80
2060.43	0.00	0.00	0.00	0.00	386.99	10000.00	3387.63	220.43	1117.40	1054.89
939.29	465.79	65.56	0.00	3.81	3380.82	10000.00	10000.00	28083.22	5125.67	238.64
30.26	0.00	8.99	0.00	0.00	499.61	440.95	146.26	3237.19	32.60	0.00
79.09	89.73	0.00	0.00	19.47	3139.25	13196.91	10000.00	16808.41	197.25	17.99
450.52	85.42	8.54	0.00	54.54	3165.83	12470.48	2746.35	15628.55	192.74	47.93
2.85	0.00	6.10	0.00	1.23	256.61	262.12	65.03	505.66	0.00	0.00

3332.31	0.56	0.00	0.00	0.00	676.53	10000.00	8548.08	134.50	2688.22	954.03
576.68	1005.73	52.75	0.00	11.20	10000.00	10000.00	10000.00	27767.26	4338.53	224.08
898.85	222.59	16.00	0.00	14.31	2659.89	10698.37	7428.42	16149.10	950.97	85.09
50.52	167.24	0.00	0.00	30.80	3729.02	13687.21	10000.00	15869.21	207.80	19.29
**330.61**	**45.33**	**0.00**	**0.00**	**25.14**	**2037.63**	**10000.00**	**4061.64**	**14579.34**	**117.27**	**36.88**
**49.22**	**0.00**	**0.00**	**0.00**	**0.00**	**687.32**	**1310.55**	**362.59**	**1906.29**	**7.97**	**0.00**

Plasma	Plasma	Plasma	Plasma	Plasma	Plasma	Plasma	Plasma	Plasma	Plasma	Plasma
**pIL-10**	**pIL-12 (p40)**	**pIL-12 (p70)**	**pIL-13**	**pIL-17**	**pIP-10**	**pKC**	**pMCP-1**	**pMIG**	**pMIP-1a**	**pTNFa**
0.00	0.00	0.00	0.00	0.00	28.99	180.20	0.00	26.23	47.21	2.86
0.00	0.00	0.00	0.00	0.00	44.93	318.72	13.27	23.67	0.00	2.74
0.00	0.00	0.00	0.00	0.00	50.89	345.61	0.00	49.05	0.00	2.74
0.00	0.00	30.33	0.00	0.09	37.39	150.71	18.93	64.62	92.31	4.14
0.48	0.43	0.00	0.00	0.00	14.66	154.78	0.00	64.99	0.00	0.80

pIL-10	pIL-12 (p40)	pIL-12 (p70)	pIL-13	pIL-17	pIP-10	pKC	pMCP-1	pMIG	pMIP-1a	pTNFa
617.84	0.00	0.00	0.00	0.00	375.42	12199.00	873.54	308.86	239.16	203.15
694.48	974.96	0.00	0.00	0.00	3558.15	11711.34	17191.19	9717.53	3992.70	129.28

350.22	829.79	53.65	0.00	11.20	2520.78	10000.00	10000.00	14926.90	1762.42	146.43
202.29	83.78	11.64	0.00	38.56	2564.92	10100.61	4303.74	15530.90	212.37	25.92
401.94	8.96	9.40	0.00	15.35	1904.37	10000.00	8199.87	19431.37	275.35	46.01
32.77	0.00	0.00	0.00	0.16	546.85	2878.21	444.08	1864.35	24.46	0.00

1653.71	0.00	37.41	0.00	2.30	857.98	12012.86	6598.28	101.19	2773.06	942.93
508.57	671.90	0.00	0.00	0.00	4450.40	11434.99	17191.19	10827.98	2542.95	80.40
218.11	467.22	36.20	0.00	9.61	2336.88	10000.00	10000.00	12097.17	1406.55	87.66
292.69	43.99	11.42	0.00	31.27	1660.80	10988.10	3676.67	15478.09	138.52	23.34
0.00	0.00	35.13	0.00	0.00	202.74	256.64	0.00	1179.60	0.00	0.00
15.79	0.00	0.00	0.00	0.11	318.49	480.99	122.27	690.32	52.76	0.00

1379.79	0.00	90.33	0.00	10.46	778.45	7122.80	6822.84	37.98	2652.74	624.63
466.15	1181.45	32.54	0.00	3.20	6373.62	12074.42	17191.19	8942.86	5577.30	89.74
379.24	301.45	21.61	0.00	25.58	4386.00	11718.72	10000.00	17930.85	957.24	70.91
272.56	71.75	16.44	0.00	31.19	2140.49	11130.69	4057.27	16490.13	145.88	23.34
218.84	0.00	8.54	0.00	0.00	971.30	2324.59	550.76	8020.03	0.00	7.02
38.24	0.00	4.52	0.00	0.54	606.26	2113.18	290.68	1558.66	16.05	0.00

1724.09	0.00	20.47	0.00	0.00	576.01	11323.56	5934.19	38.34	2189.76	771.81
570.51	756.61	90.39	0.00	5.58	10000.00	10000.00	10000.00	22722.98	3893.09	187.89
492.79	258.90	20.75	0.00	7.10	6958.22	11510.34	10000.00	15390.33	739.73	84.69
339.20	43.99	9.88	0.00	22.12	2083.05	10278.78	3499.14	17320.60	162.74	24.77
298.04	0.00	8.99	0.00	0.00	782.67	3636.04	1013.10	4423.07	60.12	1.48
100.30	0.00	0.00	0.00	0.38	1260.05	3094.63	604.20	2573.29	29.03	2.31

2060.43	0.00	0.00	0.00	0.00	386.99	10000.00	3387.63	220.43	1117.40	1054.89
939.29	465.79	65.56	0.00	3.81	3380.82	10000.00	10000.00	28083.22	5125.67	238.64
30.26	0.00	8.99	0.00	0.00	499.61	440.95	146.26	3237.19	32.60	0.00
79.09	89.73	0.00	0.00	19.47	3139.25	13196.91	10000.00	16808.41	197.25	17.99
450.52	85.42	8.54	0.00	54.54	3165.83	12470.48	2746.35	15628.55	192.74	47.93
2.85	0.00	6.10	0.00	1.23	256.61	262.12	65.03	505.66	0.00	0.00

3332.31	0.56	0.00	0.00	0.00	676.53	10000.00	8548.08	134.50	2688.22	954.03

576.68	1005.73	52.75	0.00	11.20	10000.00	10000.00	10000.00	27767.26	4338.53	224.08
898.85	222.59	16.00	0.00	14.31	2659.89	10698.37	7428.42	16149.10	950.97	85.09
50.52	167.24	0.00	0.00	30.80	3729.02	13687.21	10000.00	15869.21	207.80	19.29
**330.61**	**45.33**	**0.00**	**0.00**	**25.14**	**2037.63**	**10000.00**	**4061.64**	**14579.34**	**117.27**	**36.88**
**49.22**	**0.00**	**0.00**	**0.00**	**0.00**	**687.32**	**1310.55**	**362.59**	**1906.29**	**7.97**	**0.00**

plasma IL-10	lasma IL-12 (p40	asma IL-12 (p7	plasma IL-13	plasma IL-17	plasma IP-10	plasma KC	plasma MCP-1	plasma MIG	asma MIP-	plasma TNF
**Plasma**	**Plasma**	**Plasma**	**Plasma**	**Plasma**	**Plasma**	**Plasma**	**Plasma**	**Plasma**	**Plasma**	**Plasma**
plasma IL-10	lasma IL-12 (p40	asma IL-12 (p7	plasma IL-13	plasma IL-17	plasma IP-10	plasma KC	plasma MCP-1	plasma MIG	asma MIP-	plasma TNF
0.0	0.0	0.0	0.0	0.0	39.5	337.8	0.0	182.3	0.0	0.0
66.3	0.0	0.0	0.0	0.0	149.3	12684.8	409.2	152.7	22.9	96.1
40.33	0	13.03	0	2.48	503.42	8981.15	931.4	1390.58	59.63	1.4
14.32	0	0	0	1.93	774.66	9695	894.85	2789.45	0	0.12
8.55	0	0	0	3.27	449.12	621.46	284.04	3656.34	0	0
5.31	0	0	0	0	93.05	397.51	0	107.87	0	0
0	0	0	0	0	36.74	166.08	0	32.19	0	0
0	0	0	0	0	69.35	154.43	18.49	84.7	0	0
0	0	0	0	0	77.53	265.47	18.49	415.99	0	0
0	0	1.91	0	0	135.28	145.69	0	4840.48	0	0
451.12	0	0	0	0	305.55	10000	2322.12	111.31	151.57	158.35
195.84	4.52	0	0	0	342.47	10000	3391.59	71.4	192.61	120.37
120.86	0	0	0	0	236.21	6172.75	4631.45	45.53	63.74	100.71
15.98	0	0	0	0	437.45	4222.87	467.1	2759.09	27.24	2.68
1.85	0	0	0	0	94.55	199.19	0	228.51	52.13	0
18.12	3.16	0	0	7.6	464.73	1860.17	601.75	938.16	27.24	0.79
8.17	0	0	0	0	578.41	8460.93	206.83	5858.83	0	0.33
0	0	0	0	0	63.41	99.22	0	552.26	0	0

6.26	0	1.11	0	2.01	497.63	5913.97	490.68	4012.85	0	0

6.45	0	0	0	9.9	462.86	549.1	171.79	9208.99	0	0
5.07	0	0	0	6.6	485.05	1212.39	456.29	4529.12	0	0
12.2	0	0	0	14.04	440.08	773.77	260.1	9409.14	0	0

1.28	0	45.63	0	0	100.42	262.63	0	60.08	39.56	3.62
4.94	0	0	0	0	118.79	329.28	0	161.47	0	0
5.31	0	0	0	0	44.32	185.58	0	115.58	0	0

0	0	0	0	0	77.33	293.37	0	37.27	0	0
0.45	0	0	0	0	32.58	280.19	0	22.44	0	1.01

Plasma	Plasma	Plasma	Plasma	Plasma	Plasma	Plasma	Plasma	Plasma	Plasma	Plasma
**pIL-10**	**pIL-12 (p40)**	**pIL-12 (p70)**	**pIL-13**	**pIL-17**	**pIP-10**	**pKC**	**pMCP-1**	**pMIG**	**pMIP-1a**	**pTNFa**
0.0	0.0	0.0	0.0	0.0	39.5	337.8	0.0	182.3	0.0	0.0
0	0	0	0	0	69.35	154.43	18.49	84.7	0	0
0	0	0	0	0	77.53	265.47	18.49	415.99	0	0
0	0	1.91	0	0	135.28	145.69	0	4840.48	0	0

pIL-10	pIL-12 (p40)	pIL-12 (p70)	pIL-13	pIL-17	pIP-10	pKC	pMCP-1	pMIG	pMIP-1a	pTNFa
66.3	0.0	0.0	0.0	0.0	149.3	12684.8	409.2	152.7	22.9	96.1
40.33	0	13.03	0	2.48	503.42	8981.15	931.4	1390.58	59.63	1.4
14.32	0	0	0	1.93	774.66	9695	894.85	2789.45	0	0.12
8.55	0	0	0	3.27	449.12	621.46	284.04	3656.34	0	0
5.31	0	0	0	0	93.05	397.51	0	107.87	0	0
0	0	0	0	0	36.74	166.08	0	32.19	0	0
451.12	0	0	0	0	305.55	10000	2322.12	111.31	151.57	158.35

195.84	4.52	0	0	0	342.47	10000	3391.59	71.4	192.61	120.37
120.86	0	0	0	0	236.21	6172.75	4631.45	45.53	63.74	100.71
15.98	0	0	0	0	437.45	4222.87	467.1	2759.09	27.24	2.68
1.85	0	0	0	0	94.55	199.19	0	228.51	52.13	0
18.12	3.16	0	0	7.6	464.73	1860.17	601.75	938.16	27.24	0.79
8.17	0	0	0	0	578.41	8460.93	206.83	5858.83	0	0.33
0	0	0	0	0	63.41	99.22	0	552.26	0	0
6.26	0	1.11	0	2.01	497.63	5913.97	490.68	4012.85	0	0
6.45	0	0	0	9.9	462.86	549.1	171.79	9208.99	0	0
5.07	0	0	0	6.6	485.05	1212.39	456.29	4529.12	0	0
12.2	0	0	0	14.04	440.08	773.77	260.1	9409.14	0	0
1.28	0	45.63	0	0	100.42	262.63	0	60.08	39.56	3.62
4.94	0	0	0	0	118.79	329.28	0	161.47	0	0
5.31	0	0	0	0	44.32	185.58	0	115.58	0	0
0	0	0	0	0	77.33	293.37	0	37.27	0	0
0.45	0	0	0	0	32.58	280.19	0	22.44	0	1.01

Plasma	Heart	Heart	Heart	Heart	Heart	Heart	Heart	Heart	Heart	Heart	Heart
lasma VEG	heart GM-CS	heart IFN-g	heart IL-1a	heart IL-1b	heart IL-2	heart IL-4	heart IL-5	heart IL-6	heart IL-10	heart IL-12 (	heart IL-12 (p
1.01	0	25.47	36.48	8.035	8.07	1.58	1.165	19.415	2.535	0.895	0.53
0.73	0	22.29	26.825	5.73	8.6	1.595	1.18	17.02	3.98	0.69	0.665
1.85	11.32	37.72	76.58	9.155	9.855	1.63	1.33	462.155	10.26	2.61	2.295
11.16	43.26	275.63	299.18	40.955	238.445	1.125	0.455	362.95	11.675	13.015	4.7
5.06	14.625	128.245	133.415	6.4076	39.635	0	0	89.12	52.815	24.235	4.385
5.83	0	180.74	220.635	15.34	34.115	0.975	0	201.41	9.2	7.525	0
0.00	0	137.79	117.01	22.66	23.99	0	0	57.96	12.53	5.255	0
1.09	4.645	45.255	64.605	8.035	9.805	1.635	1.235	28.93	3.36	1.31	1.65
0.98	5.595	51.105	67.135	14.75	10.84	1.63	1.255	124.47	16.855	5.145	1.365
1.05	5.595	30.89	36.015	11.355	7.09	1.61	1.295	124.225	2.74	1.98	1.365
10.66	37.985	414.46	325.155	25.755	34.38	1.03	0.125	333.915	14.595	15.81	0
15.60	0	243.405	243.895	39.1013	70.135	0	0.56	122.105	75.205	25.785	5.27
0.12	0	224.71	296.405	22.315	55.25	1.015	0	43.01	12.09	9.87	0
0.00	0	95.275	103.47	18.745	21.43	0	0	28.195	6.465	2.96	0
1.23	0	27.725	26.34	5.73	5.765	1.585	1.18	17.21	3.315	1.035	0.53
0.75	4.645	48.085	75.77	13.51	12.82	1.615	1.22	101.63	12.855	3.145	0.805
1.29	8.38	22.105	26.215	9.895	4.68	1.64	1.36	142.05	4.175	1.865	1.935
3.34	56.99	5838.67	8840.51	13.2625	431.395	0	39.165	5939.18	64.46	449.145	14.21
11.90	0	325.795	5477.075	82.4937	5000.2	0	0	180.005	30.25	15.51	3.15
2.22	16.52	35.01	48.1	0	13.4	0	0	18.94	7.49	3.41	5.82
0.00	0	102.165	99.02	18.745	21.26	0	0	45.71	20.895	4.92	0
2.89	0	39.045	31.075	6.12	6.98	1.59	1.155	18.445	3.805	0.86	1.51
1.05	7.265	45.255	57.36	13.155	10.175	1.635	1.295	111.14	13.545	3.175	1.225
2.52	37.985	185.45	246.295	44.29	54.42	1.125	0.915	277.225	25.195	17.665	9.62
9.94	0	0	0	89.8226	0	0	23.67	3485.88	11.795	96.14	21.975
12.84	0	3260.03	148.265	5000.0	369.9	0	0	90.695	23.205	13.83	3.825

2.80	16.52	28.43	59.425	18.1438	18.1	0	0	19.11	4.8	6.69	6.695
0.00	0	128.99	130.43	24.415	30.045	0	0	61.195	18.475	7.365	0

0.06	0	201.3	346.32	15.34	61.965	1.015	0.21	54.01	15.855	8.84	0
0.15	37.985	179.41	189.32	38.45	36.69	1.115	0.455	75.695	21.78	15.055	4.7
0.54	22.02	255.255	957.525	24.04	533.015	1.09	0.455	406.43	22.63	16.46	4.7
1.92	29.585	142.925	2185.415	171.976	42.065	0	0	32.72	26.75	14.555	6.695
2.97	0	53.585	61.855	25.81	9.94	0	0	183.125	6.545	6.5	0
10.91	16.52	33.455	45.59	8.2004	12.32	0	0	342.39	0	1.31	6.695
0.00	0	206.75	227.01	29.255	47.065	0	0	69.58	15.98	11.455	0

0.22	0	241.615	204.955	27.465	39.96	1.07	0.61	73.76	18.805	11.33	2.445
1.48	37.985	719.34	2112.96	27.465	219.58	1.125	0.765	350.32	22.845	13.87	0
14.14	12.47	124.635	100.105	204.6863	33.37	0	0	177.01	35.625	11.62	5.82
0.00	5.115	45.035	63.31	35.35	10.105	0	0	151.265	4.565	7.535	0
**7.89**	**0**	**35.775**	**42.855**	**10.4995**	**11.83**	**0**	**0**	**89.225**	**1.545**	**0**	**0**
**0.00**	**0**	**78.685**	**77.54**	**17.295**	**18.02**	**0**	**0**	**24.775**	**3.305**	**2.535**	**0**

**Plasma**	**Heart**	**Heart**	**Heart**	**Heart**	**Heart**	**Heart**	**Heart**	**Heart**	**Heart**	**Heart**	**Heart**
**pVEGF**	**hGM-CSF**	**hIFN-g**	**hIL-1a**	**hIL-1b**	**hIL-2**	**hIL-4**	**hIL-5**	**hIL-6**	**hIL-10**	**hIL-12 (p40)**	**hIL-12 (p70)**
1.01	0	25.47	36.48	8.035	8.07	1.58	1.165	19.415	2.535	0.895	0.53
1.09	4.645	45.255	64.605	8.035	9.805	1.635	1.235	28.93	3.36	1.31	1.65
1.23	0	27.725	26.34	5.73	5.765	1.585	1.18	17.21	3.315	1.035	0.53
2.89	0	39.045	31.075	6.12	6.98	1.59	1.155	18.445	3.805	0.86	1.51
0.06	0	201.3	346.32	15.34	61.965	1.015	0.21	54.01	15.855	8.84	0

**pVEGF**	**hGM-CSF**	**hIFN-g**	**hIL-1a**	**hIL-1b**	**hIL-2**	**hIL-4**	**hIL-5**	**hIL-6**	**hIL-10**	**hIL-12 (p40)**	**hIL-12 (p70)**
0.73	0	22.29	26.825	5.73	8.6	1.595	1.18	17.02	3.98	0.69	0.665
1.85	11.32	37.72	76.58	9.155	9.855	1.63	1.33	462.155	10.26	2.61	2.295

11.16	43.26	275.63	299.18	40.955	238.445	1.125	0.455	362.95	11.675	13.015	4.7
5.06	14.625	128.245	133.415	6.4076	39.635	0	0	89.12	52.815	24.235	4.385
5.83	0	180.74	220.635	15.34	34.115	0.975	0	201.41	9.2	7.525	0
0.00	0	137.79	117.01	22.66	23.99	0	0	57.96	12.53	5.255	0
0.98	5.595	51.105	67.135	14.75	10.84	1.63	1.255	124.47	16.855	5.145	1.365
1.05	5.595	30.89	36.015	11.355	7.09	1.61	1.295	124.225	2.74	1.98	1.365
10.66	37.985	414.46	325.155	25.755	34.38	1.03	0.125	333.915	14.595	15.81	0
15.60	0	243.405	243.895	39.1013	70.135	0	0.56	122.105	75.205	25.785	5.27
0.12	0	224.71	296.405	22.315	55.25	1.015	0	43.01	12.09	9.87	0
0.00	0	95.275	103.47	18.745	21.43	0	0	28.195	6.465	2.96	0
0.75	4.645	48.085	75.77	13.51	12.82	1.615	1.22	101.63	12.855	3.145	0.805
1.29	8.38	22.105	26.215	9.895	4.68	1.64	1.36	142.05	4.175	1.865	1.935
3.34	56.99	5838.67	8840.51	13.2625	431.395	0	39.165	5939.18	64.46	449.145	14.21
11.90	0	325.795	5477.075	82.4937	5000.2	0	0	180.005	30.25	15.51	3.15
2.22	16.52	35.01	48.1	0	13.4	0	0	18.94	7.49	3.41	5.82
0.00	0	102.165	99.02	18.745	21.26	0	0	45.71	20.895	4.92	0
1.05	7.265	45.255	57.36	13.155	10.175	1.635	1.295	111.14	13.545	3.175	1.225
2.52	37.985	185.45	246.295	44.29	54.42	1.125	0.915	277.225	25.195	17.665	9.62
9.94	0	0	0	89.8226	0	0	23.67	3485.88	11.795	96.14	21.975
12.84	0	3260.03	148.265	5000.0	369.9	0	0	90.695	23.205	13.83	3.825
2.80	16.52	28.43	59.425	18.1438	18.1	0	0	19.11	4.8	6.69	6.695
0.00	0	128.99	130.43	24.415	30.045	0	0	61.195	18.475	7.365	0
0.15	37.985	179.41	189.32	38.45	36.69	1.115	0.455	75.695	21.78	15.055	4.7
0.54	22.02	255.255	957.525	24.04	533.015	1.09	0.455	406.43	22.63	16.46	4.7
1.92	29.585	142.925	2185.415	171.976	42.065	0	0	32.72	26.75	14.555	6.695
2.97	0	53.585	61.855	25.81	9.94	0	0	183.125	6.545	6.5	0
10.91	16.52	33.455	45.59	8.2004	12.32	0	0	342.39	0	1.31	6.695
0.00	0	206.75	227.01	29.255	47.065	0	0	69.58	15.98	11.455	0
0.22	0	241.615	204.955	27.465	39.96	1.07	0.61	73.76	18.805	11.33	2.445

1.48	37.985	719.34	2112.96	27.465	219.58	1.125	0.765	350.32	22.845	13.87	0
14.14	12.47	124.635	100.105	204.6863	33.37	0	0	177.01	35.625	11.62	5.82
0.00	5.115	45.035	63.31	35.35	10.105	0	0	151.265	4.565	7.535	0
**7.89**	**0**	**35.775**	**42.855**	**10.4995**	**11.83**	**0**	**0**	**89.225**	**1.545**	**0**	**0**
**0.00**	**0**	**78.685**	**77.54**	**17.295**	**18.02**	**0**	**0**	**24.775**	**3.305**	**2.535**	**0**

plasma VEG	heart GM-CS	heart IFN-g	heart IL-1a	heart IL-1b	heart IL-2	heart IL-4	heart IL-5	heart IL-6	heart IL-10	heart IL-12 (	heart IL-12 (p
Plasma	Heart	Heart	Heart	Heart	Heart	Heart	Heart	Heart	Heart	Heart	Heart
plasma VEG	heart GM-CS	heart IFN-g	heart IL-1a	heart IL-1b	heart IL-2	heart IL-4	heart IL-5	heart IL-6	heart IL-10	heart IL-12 (	heart IL-12 (p
0.0	0	49.34	63.845	22.31	9.725	0	0	17.6	4.25	5.42	0
0.0	0	47.39	60.245	21.6	9.295	0	0	17.7	5.83	6.73	0
0	0	0	6.91	7.9	0	0	0	0	0.925	0	0
0	0	0	24.88	0	0.385	0	0	1.34	0.68	0	0
0	0	0	3.695	0	0	0	0	0	0	0	0
0	0	3.295	21.27	62.305	3.76	0	0	1.7	0	0	0
0	0	6.79	28.015	44.74	5.085	0	0	2.76	0.94	0	0
0	0	0	0	0	0	0	0	0	0.045	0	0
0	0	0	73.235	0	0	0	0	0	1.155	0	0
0	0	0	14.895	0.91	0.65	0	0	0	0.805	0	0
0.39	0	181.585	190.475	52.555	27.48	1.07	0.455	61.675	20.93	22.89	2.445
0.08	0	121.135	8.06	42.625	184.135	0.995	0.125	100.28	20.08	19.315	0
0	0	0	9.925	0	0.155	0	0	6.825	1.155	0	0
0	0	0	10.045	0	0.14	0	0	0	0.68	0	0
0	0	0	11.97	2.205	0.28	0	0	0	1.155	0	0
0	0	2.09	90.09	3.825	3.2	0	0	0.08	1.04	0	0
0	0	3.755	21.735	2.205	1.1	0	0	1.91	1.155	0	0
0	0	0	19.49	0	0.295	0	0	0	0.98	0	0

0	0	0	59.055	4.325	1.03	0	0	0.825	2.635	0	0
0	0	0	7.66	0	0.08	0	0	0	0.805	0	0
0	0	0	8.3	0	0.09	0	0	0	0	0	0
0	0	0	11.675	0	0.235	0	0	0	0.555	0	0
0	12.375	10.47	43.56	52.43	6.81	0	0	5.865	2.47	0	0
0	26.36	10.96	92.23	17.32	14.055	0	0	22.19	10.785	6.75	0
0	0	6.52	39.42	68.895	6.24	0	0	4.62	1.715	0	0
0	12.375	10.145	38.145	102.79	6.155	0	0	5.1	1.525	1.61	0
0	0	18.96	77.88	72.92	17.825	0	0	7.525	3.39	0	0
	pg/mg										
Plasma	Heart	Heart	Heart	Heart	Heart	Heart	Heart	Heart	Heart	Heart	Heart
**pVEGF**	**hGM-CSF**	**hIFN-g**	**hIL-1a**	**hIL-1b**	**hIL-2**	**hIL-4**	**hIL-5**	**hIL-6**	**hIL-10**	**hIL-12 (p40)**	**hIL-12 (p70)**
0.0	0	49.34	63.845	22.31	9.725	0	0	17.6	4.25	5.42	0
0	0	0	0	0	0	0	0	0	0.045	0	0
0	0	0	73.235	0	0	0	0	0	1.155	0	0
0	0	0	14.895	0.91	0.65	0	0	0	0.805	0	0
**pVEGF**	**hGM-CSF**	**hIFN-g**	**hIL-1a**	**hIL-1b**	**hIL-2**	**hIL-4**	**hIL-5**	**hIL-6**	**hIL-10**	**hIL-12 (p40)**	**hIL-12 (p70)**
0.0	0	47.39	60.245	21.6	9.295	0	0	17.7	5.83	6.73	0
0	0	0	6.91	7.9	0	0	0	0	0.925	0	0
0	0	0	24.88	0	0.385	0	0	1.34	0.68	0	0
0	0	0	3.695	0	0	0	0	0	0	0	0
0	0	3.295	21.27	62.305	3.76	0	0	1.7	0	0	0
0	0	6.79	28.015	44.74	5.085	0	0	2.76	0.94	0	0
0.39	0	181.585	190.475	52.555	27.48	1.07	0.455	61.675	20.93	22.89	2.445

0.08	0	121.135	8.06	42.625	184.135	0.995	0.125	100.28	20.08	19.315	0
0	0	0	9.925	0	0.155	0	0	6.825	1.155	0	0
0	0	0	10.045	0	0.14	0	0	0	0.68	0	0
0	0	0	11.97	2.205	0.28	0	0	0	1.155	0	0
0	0	2.09	90.09	3.825	3.2	0	0	0.08	1.04	0	0
0	0	3.755	21.735	2.205	1.1	0	0	1.91	1.155	0	0
0	0	0	19.49	0	0.295	0	0	0	0.98	0	0
0	0	0	59.055	4.325	1.03	0	0	0.825	2.635	0	0
0	0	0	7.66	0	0.08	0	0	0	0.805	0	0
0	0	0	8.3	0	0.09	0	0	0	0	0	0
0	0	0	11.675	0	0.235	0	0	0	0.555	0	0
0	12.375	10.47	43.56	52.43	6.81	0	0	5.865	2.47	0	0
0	26.36	10.96	92.23	17.32	14.055	0	0	22.19	10.785	6.75	0
0	0	6.52	39.42	68.895	6.24	0	0	4.62	1.715	0	0
0	12.375	10.145	38.145	102.79	6.155	0	0	5.1	1.525	1.61	0
0	0	18.96	77.88	72.92	17.825	0	0	7.525	3.39	0	0

									liver GM-C	liver IFN-g	liver IL-1a	liver IL-1b
**Heart**	**Heart**	**Heart**	**Heart**	**Heart**	**Heart**	**Heart**	**Heart**	**Heart**	**Liver**	**Liver**	**Liver**	**Liver**
**heart IL-13**	**heart IL-17**	**heart IP-10**	**heart KC**	**heart MCP**	**heart MIG**	**heart MIP-**	**heart TNFa**	**heart VEGF**	**liver GM-C**	**liver IFN-g**	**liver IL-1a**	**liver IL-1b**
1.795	1.34	541.505	50.21	12.895	583.66	5.28	2.09	24.445	9.42	30.58	134.905	49.67
1.61	1.32	20.515	25.575	10.485	365.885	4.66	2.105	98.185	4.645	78.135	140.62	13.865
37.035	1.41	2208.81	1194.19	1260.995	4892.455	65.285	2.19	117.76	9.42	0	803.03	56.12
0	0	5000.0	1077.07	1104.185	11939.02	115.39	2.02	106.295	47.77	276.485	1751.585	104.1
9.285	0	1124.405	606.675	382.86	12071.31	19.715	0	37.045	43.17	141.415	317.55	71.5709
0	0	1029.15	388.365	245.095	9582.91	41.925	0.715	22.525	0	51.66	230.215	18.85
0	0.165	153.685	70.01	251.265	2066.465	18.755	0	0.58	0	6.905	135.36	15.835
19.455	1.425	75.02	5.3	9.48	581.505	6.435	2.11	20.115	8.735	174.055	411.075	47.81
27.19	1.365	105.42	2216.72	393.705	184.505	31.785	2.765	38.575	9.42	65.93	127.99	23.475
28.765	1.405	3295.235	538.895	715.605	3623.705	34.735	2.17	45.31	7.265	64.565	114.705	34.905
0	0	1649.66	1007.645	990.635	10717.89	92.98	1.545	78.91	47.77	194.4	927.055	91.385
45.87	0	793.12	238.975	149.89	9928.695	19.715	0	42.265	45.975	99.505	277.565	51.7588
0	0	39.75	8.755	0	887.985	25.205	0.885	19.93	0	17.585	237.455	29.17
0	0.135	75.615	23.665	115.51	1205.315	11.435	0	1.215	0	0.445	169.72	12.105
0.5	1.355	37.925	2.75	6.81	236.03	2.56	2.075	51.6	0	25.19	87.105	8.785
19.5	1.39	134.105	1811.795	319.025	304.01	31.185	2.62	43.315	2.255	101.255	480.03	11.72
33.97	1.465	3206.06	645.585	599.935	4957.995	48.27	2.22	33.605	11.32	145.985	285.11	40.935
177.685	1.77	481.27	3885.15	1170.46	22265.9	2195.685	21.635	369.845	14.625	22.14	225.195	56.7886
0	0	1568.205	463.435	398.9	22265.86	12.53	0	32.845	49.265	139.525	370.835	69.3548
0	0	168.515	65.155	59.315	5824.805	19.715	0	7.695	25.14	9.67	101.17	18.1438
2.35	0.135	123.98	65.215	255.385	1390.04	13.005	0	0.225	6.795	17.23	175.855	25.115
1.43	1.355	27.28	1.76	4.44	274.28	3.67	2.075	33.225	7.265	86.48	5000.0	30.715
23.365	1.42	184.23	2651.71	467.71	399.09	51.915	2.815	86.615	7.265	32.125	395.3	15.275
82.63	0	5000.0	1007.545	967.255	6566.045	75.785	2.325	62.155	67.27	174.465	711.21	135.535
155.995	369.61	0	5547.905	2680.205	22265.9	177.895	21.205	40.02	27.45	19.105	174.22	233.7031
0	0	891.09	355.865	266.905	14622.84	15.4	0	21.855	51.145	142.925	232.97	108.8545

0	0	181.39	77.645	115.24	2755.775	21.325	0	17.605	12.47	5.86	106.46	14.3922
0	0.295	133.345	93.245	279.91	1810.505	13.005	0	0.575	0	52.21	194.04	20.535
0	0	272.55	53.06	0	926.73	0	1.055	38.935	43.26	278.93	624.76	68.88
100.475	0	353.91	1453.28	398.64	1010.88	60.985	6.055	97.91	47.77	245.02	981.09	94.575
52.18	0	5000.0	1365.51	1132.615	5571.61	98.47	2.48	47.555	37.985	254.525	736.225	132.41
0	0	178.98	23.135	43.015	3318.905	24.39	0	5.96	31.585	25.57	136.21	35.2584
0	0.19	3368.805	942.86	770.245	9308.71	46.83	0	29.02	13.565	194.41	273.48	69.335
0	0	2201.305	997.015	344.28	7429.095	27.29	0	28.94	12.47	8.97	104.965	0
11.535	0.425	355.97	87.835	89.96	4075.76	15.97	0	5.525	0	21.755	251.205	12.105
0	0	158.6	1494.555	446.715	266.715	50.32	3.95	35.25	51.75	241.005	685.975	137.095
53.15	0	5000.0	1072.89	886.18	11537.05	81.025	2.175	68.17	61.71	270.745	4203.37	154.195
0	0	2162.55	559.23	531.47	8710.49	48.16	0	46.34	16.52	23.645	339.165	97.9151
0	0.245	2306.22	567.62	439.34	9689.35	21.4	0	22.21	19.78	217.165	284.355	106.155
**0**	**0**	**1008.675**	**227.405**	**96.96**	**6517.595**	**8.145**	**0**	**19.235**	**0**	**6.73**	**133.535**	**13.2966**
**0**	**0.025**	**183.76**	**57.825**	**142.96**	**2103.71**	**8.025**	**0**	**0.59**	**2.835**	**140.74**	**236.425**	**43.29**
**Heart**	**Heart**	**Heart**	**Heart**	**Heart**	**Heart**	**Heart**	**Heart**	**Heart**	**Liver**	**Liver**	**Liver**	**Liver**
**hIL-13**	**hIL-17**	**hIP-10**	**hKC**	**hMCP-1**	**hMIG**	**hMIP-1a**	**hTNFa**	**hVEGF**	**LGM-CSF**	**LIFN-g**	**LIL-1a**	**LIL-1b**
1.795	1.34	541.505	50.21	12.895	583.66	5.28	2.09	24.445	9.42	30.58	134.905	49.67
19.455	1.425	75.02	5.3	9.48	581.505	6.435	2.11	20.115	8.735	174.055	411.075	47.81
0.5	1.355	37.925	2.75	6.81	236.03	2.56	2.075	51.6	0	25.19	87.105	8.785
1.43	1.355	27.28	1.76	4.44	274.28	3.67	2.075	33.225	7.265	86.48	5000.0	30.715
0	0	272.55	53.06	0	926.73	0	1.055	38.935	43.26	278.93	624.76	68.88
**hIL-13**	**hIL-17**	**hIP-10**	**hKC**	**hMCP-1**	**hMIG**	**hMIP-1a**	**hTNFa**	**hVEGF**	**LGM-CSF**	**LIFN-g**	**LIL-1a**	**LIL-1b**
1.61	1.32	20.515	25.575	10.485	365.885	4.66	2.105	98.185	4.645	78.135	140.62	13.865
37.035	1.41	2208.81	1194.19	1260.995	4892.455	65.285	2.19	117.76	9.42	0	803.03	56.12

0	0	5000.0	1077.07	1104.185	11939.02	115.39	2.02	106.295	47.77	276.485	1751.585	104.1
9.285	0	1124.405	606.675	382.86	12071.31	19.715	0	37.045	43.17	141.415	317.55	71.5709
0	0	1029.15	388.365	245.095	9582.91	41.925	0.715	22.525	0	51.66	230.215	18.85
0	0.165	153.685	70.01	251.265	2066.465	18.755	0	0.58	0	6.905	135.36	15.835
27.19	1.365	105.42	2216.72	393.705	184.505	31.785	2.765	38.575	9.42	65.93	127.99	23.475
28.765	1.405	3295.235	538.895	715.605	3623.705	34.735	2.17	45.31	7.265	64.565	114.705	34.905
0	0	1649.66	1007.645	990.635	10717.89	92.98	1.545	78.91	47.77	194.4	927.055	91.385
45.87	0	793.12	238.975	149.89	9928.695	19.715	0	42.265	45.975	99.505	277.565	51.7588
0	0	39.75	8.755	0	887.985	25.205	0.885	19.93	0	17.585	237.455	29.17
0	0.135	75.615	23.665	115.51	1205.315	11.435	0	1.215	0	0.445	169.72	12.105
19.5	1.39	134.105	1811.795	319.025	304.01	31.185	2.62	43.315	2.255	101.255	480.03	11.72
33.97	1.465	3206.06	645.585	599.935	4957.995	48.27	2.22	33.605	11.32	145.985	285.11	40.935
177.685	1.77	481.27	3885.15	1170.46	22265.9	2195.685	21.635	369.845	14.625	22.14	225.195	56.7886
0	0	1568.205	463.435	398.9	22265.86	12.53	0	32.845	49.265	139.525	370.835	69.3548
0	0	168.515	65.155	59.315	5824.805	19.715	0	7.695	25.14	9.67	101.17	18.1438
2.35	0.135	123.98	65.215	255.385	1390.04	13.005	0	0.225	6.795	17.23	175.855	25.115
23.365	1.42	184.23	2651.71	467.71	399.09	51.915	2.815	86.615	7.265	32.125	395.3	15.275
82.63	0	5000.0	1007.545	967.255	6566.045	75.785	2.325	62.155	67.27	174.465	711.21	135.535
155.995	369.61	0	5547.905	2680.205	22265.9	177.895	21.205	40.02	27.45	19.105	174.22	233.7031
0	0	891.09	355.865	266.905	14622.84	15.4	0	21.855	51.145	142.925	232.97	108.8545
0	0	181.39	77.645	115.24	2755.775	21.325	0	17.605	12.47	5.86	106.46	14.3922
0	0.295	133.345	93.245	279.91	1810.505	13.005	0	0.575	0	52.21	194.04	20.535
100.475	0	353.91	1453.28	398.64	1010.88	60.985	6.055	97.91	47.77	245.02	981.09	94.575
52.18	0	5000.0	1365.51	1132.615	5571.61	98.47	2.48	47.555	37.985	254.525	736.225	132.41
0	0	178.98	23.135	43.015	3318.905	24.39	0	5.96	31.585	25.57	136.21	35.2584
0	0.19	3368.805	942.86	770.245	9308.71	46.83	0	29.02	13.565	194.41	273.48	69.335
0	0	2201.305	997.015	344.28	7429.095	27.29	0	28.94	12.47	8.97	104.965	0
11.535	0.425	355.97	87.835	89.96	4075.76	15.97	0	5.525	0	21.755	251.205	12.105
0	0	158.6	1494.555	446.715	266.715	50.32	3.95	35.25	51.75	241.005	685.975	137.095

53.15	0	5000.0	1072.89	886.18	11537.05	81.025	2.175	68.17	61.71	270.745	4203.37	154.195
0	0	2162.55	559.23	531.47	8710.49	48.16	0	46.34	16.52	23.645	339.165	97.9151
0	0.245	2306.22	567.62	439.34	9689.35	21.4	0	22.21	19.78	217.165	284.355	106.155
**0**	**0**	**1008.675**	**227.405**	**96.96**	**6517.595**	**8.145**	**0**	**19.235**	**0**	**6.73**	**133.535**	**13.2966**
**0**	**0.025**	**183.76**	**57.825**	**142.96**	**2103.71**	**8.025**	**0**	**0.59**	**2.835**	**140.74**	**236.425**	**43.29**

heart IL-13	heart IL-17	heart IP-10	heart KC	heart MCP	heart MIG	heart MIP-	heart TNFa	heart VEGF	liver GM-C	liver IFN-g	liver IL-1a	liver IL-1b
Heart	Heart	Heart	Heart	Heart	Heart	Heart	Heart	Heart	Liver	Liver	Liver	Liver
heart IL-13	heart IL-17	heart IP-10	heart KC	heart MCP	heart MIG	heart MIP-	heart TNFa	heart VEGF	liver GM-C	liver IFN-g	liver IL-1a	liver IL-1b
0	0	79.345	12.705	9.02	1146.07	11.435	0	2.115	11.65	365.33	469.51	90.075
0	0.19	65.86	154.92	22.795	392.35	11.435	0	3.215	23.605	178.1	235.135	91.88
0	0	62.755	80.555	94.34	1047.265	0	0	37.995	3.015	0	21.025	12.195
0	0	47.635	43.45	14.59	1415.155	0	0	3.005	0	0	27.85	5.735
0	0	46.84	7.955	35.815	1515.215	0	0	3.49	0	0	45.195	9.915
0.07	0	22.125	6.94	34.05	440.615	0	0.89	12.525	26.36	111.65	182.125	40.895
0	0	8.44	2.25	13.07	269.215	5.66	0.505	38.205	30.785	47.98	142.59	44.205
0	0	30.33	4.075	4.39	343.365	0	0	45.26	0	0	8.24	0
0	0	15.38	0	1.625	240.81	0	0	49.2	3.015	0	14.015	4.805
0	0	11.55	0	10.885	97.895	0	0	22.3	0	0	9.695	3.31
34.94	0	243.165	588.895	195.725	701.11	50.32	2.175	11.13	67.27	268.185	494.555	119.09
0	0	46.165	775.465	319.015	296.395	0	2.175	9.365	43.26	270.87	832.215	89.79
0	0	44.97	392.46	125.25	80.165	0	0.08	12.44	0	0	33.81	11.07
0	0	54.8	37	54.485	681.245	0	0	27.715	9.31	0	19.02	7.48
0	0	26	1.055	0	113.46	0	0	43.03	0	0	11.325	4.325
0	0	47.17	84.635	45.77	93.715	0	0.02	28.875	3.015	0	9.985	3.31
0	0.18	97.76	34.79	15.285	1161.04	0	0	2.55	0	0	25.825	0.91
0	0	12.26	0	0	89.49	0	0	1.18	3.015	0	43.875	9.12

0	0	54.225	18.12	6.515	3643.645	0	0	0.26	13.1	0.365	132.795	32.895
0	0	48.935	9.055	21.025	2170.285	0	0	0.205	0	12.72	289.275	25.795
0	0	85.055	9.77	39.56	3087.62	0	0	2.2	0	0	34.885	12.93
0	0	37.19	9.2	10.08	1673.215	0	0	1.105	0	6.93	274.62	28.21
2.94	0	10.01	6.01	31.375	286.935	11.785	1.095	31.425	12.375	35.44	123.17	29.86
0	0	45.47	25.52	22.78	1015.13	22.985	3.065	18.67	0	2.97	24.48	107.085
2.575	0	12.42	5.74	39.615	185.8	5.66	0.37	15.375	0	13.02	67.135	10.77
20.505	0	20.67	7.025	22.78	180.17	11.785	1.495	48.525	43.68	61.47	128.73	32.5
11.67	0	8.965	4.605	20.07	388.645	5.66	1.015	42.355	34.555	73.925	77.945	41.735

									pg/mg			
Heart	Heart	Heart	Heart	Heart	Heart	Heart	Heart	Heart	Liver	Liver	Liver	Liver
**hIL-13**	**hIL-17**	**hIP-10**	**hKC**	**hMCP-1**	**hMIG**	**hMIP-1a**	**hTNFa**	**hVEGF**	**LGM-CSF**	**LIFN-g**	**LIL-1a**	**LIL-1b**
0	0	79.345	12.705	9.02	1146.07	11.435	0	2.115	11.65	365.33	469.51	90.075
0	0	30.33	4.075	4.39	343.365	0	0	45.26	0	0	8.24	0
0	0	15.38	0	1.625	240.81	0	0	49.2	3.015	0	14.015	4.805
0	0	11.55	0	10.885	97.895	0	0	22.3	0	0	9.695	3.31

hIL-13	hIL-17	hIP-10	hKC	hMCP-1	hMIG	hMIP-1a	hTNFa	hVEGF	LGM-CSF	LIFN-g	LIL-1a	LIL-1b
0	0.19	65.86	154.92	22.795	392.35	11.435	0	3.215	23.605	178.1	235.135	91.88
0	0	62.755	80.555	94.34	1047.265	0	0	37.995	3.015	0	21.025	12.195
0	0	47.635	43.45	14.59	1415.155	0	0	3.005	0	0	27.85	5.735
0	0	46.84	7.955	35.815	1515.215	0	0	3.49	0	0	45.195	9.915
0.07	0	22.125	6.94	34.05	440.615	0	0.89	12.525	26.36	111.65	182.125	40.895
0	0	8.44	2.25	13.07	269.215	5.66	0.505	38.205	30.785	47.98	142.59	44.205
34.94	0	243.165	588.895	195.725	701.11	50.32	2.175	11.13	67.27	268.185	494.555	119.09

0	0	46.165	775.465	319.015	296.395	0	2.175	9.365	43.26	270.87	832.215	89.79
0	0	44.97	392.46	125.25	80.165	0	0.08	12.44	0	0	33.81	11.07
0	0	54.8	37	54.485	681.245	0	0	27.715	9.31	0	19.02	7.48
0	0	26	1.055	0	113.46	0	0	43.03	0	0	11.325	4.325
0	0	47.17	84.635	45.77	93.715	0	0.02	28.875	3.015	0	9.985	3.31
0	0.18	97.76	34.79	15.285	1161.04	0	0	2.55	0	0	25.825	0.91
0	0	12.26	0	0	89.49	0	0	1.18	3.015	0	43.875	9.12
0	0	54.225	18.12	6.515	3643.645	0	0	0.26	13.1	0.365	132.795	32.895
0	0	48.935	9.055	21.025	2170.285	0	0	0.205	0	12.72	289.275	25.795
0	0	85.055	9.77	39.56	3087.62	0	0	2.2	0	0	34.885	12.93
0	0	37.19	9.2	10.08	1673.215	0	0	1.105	0	6.93	274.62	28.21
2.94	0	10.01	6.01	31.375	286.935	11.785	1.095	31.425	12.375	35.44	123.17	29.86
0	0	45.47	25.52	22.78	1015.13	22.985	3.065	18.67	0	2.97	24.48	107.085
2.575	0	12.42	5.74	39.615	185.8	5.66	0.37	15.375	0	13.02	67.135	10.77
20.505	0	20.67	7.025	22.78	180.17	11.785	1.495	48.525	43.68	61.47	128.73	32.5
11.67	0	8.965	4.605	20.07	388.645	5.66	1.015	42.355	34.555	73.925	77.945	41.735

liver IL-2	liver IL-4	liver IL-5	liver IL-6	liver IL-10	liver IL-12 (	liver IL-12 (	liver IL-13	liver IL-17	liver IP-10	liver KC	liver MCP-1
**Liver**	**Liver**	**Liver**	**Liver**	**Liver**	**Liver**	**Liver**	**Liver**	**Liver**	**Liver**	**Liver**	**Liver**
**liver IL-2**	**liver IL-4**	**liver IL-5**	**liver IL-6**	**liver IL-10**	**liver IL-12 (**	**liver IL-12 (**	**liver IL-13**	**liver IL-17**	**liver IP-10**	**liver KC**	**liver MCP-1**
12.695	1.75	1.31	107.14	12.695	13.145	9.265	29.115	1.765	79.56	12.285	13.36
12.335	1.695	1.285	284.68	9.355	4.645	1.085	18.56	1.39	873.925	906.83	265.935
0.97	1.71	1.405	305.89	9.755	10.9	9.035	43.135	1.715	3013.165	1118.325	606.905
61.855	1.4	1.935	403.77	87.065	94.225	8.14	422.14	1.545	5000.0	1117.6	795.24
66.965	0	9.325	300.14	192.235	93.375	9.19	251.625	9.06	983.065	735.2	234.09
11.785	1.07	0.295	168.83	13.76	10.7	0	0	0	1085.01	918.005	425.755
11.325	0	0	9.82	8.055	8.59	0	4.515	0.27	201.45	35.465	54.53
22.6	1.73	1.31	195.435	14.24	12.515	2.88	75.04	1.79	69.415	15.44	14.505
10.965	1.705	1.295	309.09	8.145	6.595	1.51	28.94	1.625	1915.56	1752.075	530.65
12.055	1.675	1.295	368.085	4.045	6.33	1.65	39.45	1.58	3485.66	1463.31	654.255
41.875	1.28	1.65	433.895	83.46	77.465	6.525	372.61	1.305	5000.0	1174.87	780.305
77.095	0	7.28	252.775	190.93	108.845	11.445	243.925	10.375	722.66	625.57	204.205
12.49	1.05	0.21	10.905	18.385	18.435	0	99.93	0	106.08	19.41	0
26.34	0	0	3.95	6.545	4.64	0	0	0.135	143.735	20.68	32.12
8.55	1.675	1.18	68.725	3.485	2.565	0	16.1	1.32	73.585	6.97	5.66
13.955	1.615	1.22	443.64	2.555	2.205	1.085	6.935	1.365	1309.06	1177.28	439.82
23.305	1.8	1.395	318.13	21.285	14.88	11.88	109.72	1.85	5797.21	2194.99	744.885
18.27	0	0	411.18	33.33	19.03	6.48	0.91	3.205	1942.56	1436.32	606.185
72.7	0	9.56	337.64	231.685	112.56	10.375	215.305	13.555	1864.985	1100.165	327.99
4.47	0	0	21.785	15.54	9.335	6.695	0	0	388.25	220.475	143.775
12.225	0	0	14.39	15.98	11.27	0	16.085	0.74	278.765	30.555	60.65
15.94	1.66	1.255	110.785	7.01	7.55	5.77	70.665	1.48	49.58	5.555	7.905
13.39	1.65	1.275	431.24	3.19	3.205	0.805	11.545	1.425	1153.73	1754.76	524.125
46.575	1.48	2.22	383.83	143.65	125.975	15.905	507.635	2.555	5000.0	1423.355	927.54
15.25	0	0	393.99	32.56	20.405	6.695	0	1.125	2121.405	1431.405	732.26
52.58	0	7.56	268.54	198.75	101.555	9.515	231.045	8.825	1070.335	929.615	289.485

2.385	0	0	17.07	7.49	4.155	5.6	0	0	325.07	155.75	152.025
17.045	0	0	13.72	17.43	17.32	0.975	14.595	0.7	242.885	55.58	55.2
50.11	1.28	0.915	113.605	77.165	83.615	2.445	349.725	0	269.185	147.115	68.71
49.72	1.32	2.08	352.785	69.995	68.84	8.14	331.24	0.535	891.22	1600.235	634.175
33.785	1.4	1.215	425.145	60.855	66.575	4.7	230.18	0.31	5000.0	1746.375	1311.335
23.11	0	0	23.24	49.125	20.86	7.135	38.365	0.59	456.19	63.845	50.88
35.66	0	0	204.575	44.03	51.68	43.625	137.69	3.025	4826.205	1603.325	546.16
3.235	0	0	310.655	3.875	0	5.49	0	0	1708.55	2228.375	529.91
7.63	0	0	1.735	6.465	4.145	0	0	0.165	107.425	12.385	22.355
60.94	1.47	2.5	465.89	145.49	113.425	56.84	611.18	2.645	978.545	1563.345	728.335
189.07	1.43	2.36	455.55	124.355	113.425	13.555	509.765	2.085	5000.0	1487.585	995
19.11	0	0	490.32	34.86	15.51	6.915	0	1.46	2675.325	1874.085	793.445
40.345	0	0	246.38	57.51	71.895	26.425	170.06	5.1	2074.35	1179.52	380.15
**3.41**	**0**	**0**	**207.775**	**5.355**	**4.975**	**0**	**0**	**0**	**934.78**	**1742.42**	**290.57**
**27.135**	**0**	**0**	**57.45**	**36.945**	**42.115**	**37.165**	**115.845**	**1.82**	**311.61**	**72.945**	**94.975**

Liver	Liver	Liver	Liver	Liver	Liver	Liver	Liver	Liver	Liver	Liver	Liver
**LIL-2**	**LIL-4**	**LIL-5**	**LIL-6**	**LIL-10**	**LIL-12 (p40)**	**LIL-12 (p70)**	**LIL-13**	**LIL-17**	**LIP-10**	**LKC**	**LMCP-1**
12.695	1.75	1.31	107.14	12.695	13.145	9.265	29.115	1.765	79.56	12.285	13.36
22.6	1.73	1.31	195.435	14.24	12.515	2.88	75.04	1.79	69.415	15.44	14.505
8.55	1.675	1.18	68.725	3.485	2.565	0	16.1	1.32	73.585	6.97	5.66
15.94	1.66	1.255	110.785	7.01	7.55	5.77	70.665	1.48	49.58	5.555	7.905
50.11	1.28	0.915	113.605	77.165	83.615	2.445	349.725	0	269.185	147.115	68.71
**LIL-2**	**LIL-4**	**LIL-5**	**LIL-6**	**LIL-10**	**LIL-12 (p40)**	**LIL-12 (p70)**	**LIL-13**	**LIL-17**	**LIP-10**	**LKC**	**LMCP-1**
12.335	1.695	1.285	284.68	9.355	4.645	1.085	18.56	1.39	873.925	906.83	265.935
0.97	1.71	1.405	305.89	9.755	10.9	9.035	43.135	1.715	3013.165	1118.325	606.905

61.855	1.4	1.935	403.77	87.065	94.225	8.14	422.14	1.545	5000.0	1117.6	795.24
66.965	0	9.325	300.14	192.235	93.375	9.19	251.625	9.06	983.065	735.2	234.09
11.785	1.07	0.295	168.83	13.76	10.7	0	0	0	1085.01	918.005	425.755
11.325	0	0	9.82	8.055	8.59	0	4.515	0.27	201.45	35.465	54.53
10.965	1.705	1.295	309.09	8.145	6.595	1.51	28.94	1.625	1915.56	1752.075	530.65
12.055	1.675	1.295	368.085	4.045	6.33	1.65	39.45	1.58	3485.66	1463.31	654.255
41.875	1.28	1.65	433.895	83.46	77.465	6.525	372.61	1.305	5000.0	1174.87	780.305
77.095	0	7.28	252.775	190.93	108.845	11.445	243.925	10.375	722.66	625.57	204.205
12.49	1.05	0.21	10.905	18.385	18.435	0	99.93	0	106.08	19.41	0
26.34	0	0	3.95	6.545	4.64	0	0	0.135	143.735	20.68	32.12
13.955	1.615	1.22	443.64	2.555	2.205	1.085	6.935	1.365	1309.06	1177.28	439.82
23.305	1.8	1.395	318.13	21.285	14.88	11.88	109.72	1.85	5797.21	2194.99	744.885
18.27	0	0	411.18	33.33	19.03	6.48	0.91	3.205	1942.56	1436.32	606.185
72.7	0	9.56	337.64	231.685	112.56	10.375	215.305	13.555	1864.985	1100.165	327.99
4.47	0	0	21.785	15.54	9.335	6.695	0	0	388.25	220.475	143.775
12.225	0	0	14.39	15.98	11.27	0	16.085	0.74	278.765	30.555	60.65
13.39	1.65	1.275	431.24	3.19	3.205	0.805	11.545	1.425	1153.73	1754.76	524.125
46.575	1.48	2.22	383.83	143.65	125.975	15.905	507.635	2.555	5000.0	1423.355	927.54
15.25	0	0	393.99	32.56	20.405	6.695	0	1.125	2121.405	1431.405	732.26
52.58	0	7.56	268.54	198.75	101.555	9.515	231.045	8.825	1070.335	929.615	289.485
2.385	0	0	17.07	7.49	4.155	5.6	0	0	325.07	155.75	152.025
17.045	0	0	13.72	17.43	17.32	0.975	14.595	0.7	242.885	55.58	55.2
49.72	1.32	2.08	352.785	69.995	68.84	8.14	331.24	0.535	891.22	1600.235	634.175
33.785	1.4	1.215	425.145	60.855	66.575	4.7	230.18	0.31	5000.0	1746.375	1311.335
23.11	0	0	23.24	49.125	20.86	7.135	38.365	0.59	456.19	63.845	50.88
35.66	0	0	204.575	44.03	51.68	43.625	137.69	3.025	4826.205	1603.325	546.16
3.235	0	0	310.655	3.875	0	5.49	0	0	1708.55	2228.375	529.91
7.63	0	0	1.735	6.465	4.145	0	0	0.165	107.425	12.385	22.355
60.94	1.47	2.5	465.89	145.49	113.425	56.84	611.18	2.645	978.545	1563.345	728.335

189.07	1.43	2.36	455.55	124.355	113.425	13.555	509.765	2.085	5000.0	1487.585	995
19.11	0	0	490.32	34.86	15.51	6.915	0	1.46	2675.325	1874.085	793.445
40.345	0	0	246.38	57.51	71.895	26.425	170.06	5.1	2074.35	1179.52	380.15
**3.41**	**0**	**0**	**207.775**	**5.355**	**4.975**	**0**	**0**	**0**	**934.78**	**1742.42**	**290.57**
**27.135**	**0**	**0**	**57.45**	**36.945**	**42.115**	**37.165**	**115.845**	**1.82**	**311.61**	**72.945**	**94.975**
**liver IL-2**	**liver IL-4**	**liver IL-5**	**liver IL-6**	**liver IL-10**	**liver IL-12 (**	**liver IL-12 (**	**liver IL-13**	**liver IL-17**	**liver IP-10**	**liver KC**	**liver MCP-**
**Liver**	**Liver**	**Liver**	**Liver**	**Liver**	**Liver**	**Liver**	**Liver**	**Liver**	**Liver**	**Liver**	**Liver**
**liver IL-2**	**liver IL-4**	**liver IL-5**	**liver IL-6**	**liver IL-10**	**liver IL-12 (**	**liver IL-12 (**	**liver IL-13**	**liver IL-17**	**liver IP-10**	**liver KC**	**liver MCP-**
83.725	0	0	267.52	49.58	53.36	9.64	182.345	3.21	116.835	19.08	23.015
34.905	0	0.445	121.1	42.97	53.925	40.03	167.895	2.84	608.22	664.86	113.225
0.36	0	0	11.095	4.6	0	0	0	0	495.14	181.33	108.135
1.075	0	0	19.535	6.055	0	0	0	0	214.435	123.04	30.895
1.76	0	0	7.13	6.81	0	0	0	0	121.72	33.815	34.855
41.055	0	0	63.615	33.34	17.46	0	2.755	0	29.25	46.225	27.41
28.76	0	0	50.07	30.665	17.98	0	3.475	0	59.52	28.575	25.2
0	0	0	0.49	1.49	0	0	0	0	78.495	11.18	3.785
0.06	0	0	0	1.915	0	0	0	0	75.48	2.14	0
0.14	0	0	0.31	2.885	0	0	0	0	47.74	2.985	0
48.03	1.43	2.08	173.645	81.885	72.635	37.51	497.165	2.115	674.735	1036.11	372.75
58.425	1.205	1.795	207.41	73.575	73.395	5.645	438.785	1.515	702.765	890.44	431.335
0.68	0	0	64.935	5.83	0	0	0	0	703.965	891.095	330.705
0.06	0	0	10.04	3.515	0	0	0	0	551.53	125.555	97.51
0.015	0	0	0.18	3.325	0	0	0	0	66.6	4.14	3.14
0	0	0	22.425	1.705	0	0	0	0	380.495	799.09	108.735
0.81	0	0	14.25	2.93	0	0	0	0	231.17	93.62	13.885
1.485	0	0	0.005	8.465	0	0	0	0	29.555	6.255	0

8.835	0	0	20.05	34.295	0	0	0.495	0	196.37	93.845	27.775
9.35	0	0	8.31	17.855	0	0	0	0	142.74	33.125	40.47
1.015	0	0	6.145	8.29	0	0	0	0	365.175	77.77	87.215
3.625	0	0	4.455	19.885	0	0	0	0	104.8	31.985	48.745
24.485	0	0	57.735	27.18	12.205	0	0	0	39.23	42.435	22.78
8.055	0	0	2.105	1.14	0	0	23.08	0	22.59	13.445	57.85
11.08	0	0	17.67	6.935	0.41	0	0	0	25.555	20.3	16.935
25.83	0	0	54.485	22.3	12.735	0	2.575	0	72.045	48.035	34.9
24.655	0	0	59.7	30.98	13.97	0	4.01	0	46.56	44.36	27.41

Liver	Liver	Liver	Liver	Liver	Liver	Liver	Liver	Liver	Liver	Liver	Liver
**LIL-2**	**LIL-4**	**LIL-5**	**LIL-6**	**LIL-10**	**LIL-12 (p40)**	**LIL-12 (p70)**	**LIL-13**	**LIL-17**	**LIP-10**	**LKC**	**LMCP-1**
83.725	0	0	267.52	49.58	53.36	9.64	182.345	3.21	116.835	19.08	23.015
0	0	0	0.49	1.49	0	0	0	0	78.495	11.18	3.785
0.06	0	0	0	1.915	0	0	0	0	75.48	2.14	0
0.14	0	0	0.31	2.885	0	0	0	0	47.74	2.985	0

LIL-2	LIL-4	LIL-5	LIL-6	LIL-10	LIL-12 (p40)	LIL-12 (p70)	LIL-13	LIL-17	LIP-10	LKC	LMCP-1
34.905	0	0.445	121.1	42.97	53.925	40.03	167.895	2.84	608.22	664.86	113.225
0.36	0	0	11.095	4.6	0	0	0	0	495.14	181.33	108.135
1.075	0	0	19.535	6.055	0	0	0	0	214.435	123.04	30.895
1.76	0	0	7.13	6.81	0	0	0	0	121.72	33.815	34.855
41.055	0	0	63.615	33.34	17.46	0	2.755	0	29.25	46.225	27.41
28.76	0	0	50.07	30.665	17.98	0	3.475	0	59.52	28.575	25.2
48.03	1.43	2.08	173.645	81.885	72.635	37.51	497.165	2.115	674.735	1036.11	372.75

58.425	1.205	1.795	207.41	73.575	73.395	5.645	438.785	1.515	702.765	890.44	431.335
0.68	0	0	64.935	5.83	0	0	0	0	703.965	891.095	330.705
0.06	0	0	10.04	3.515	0	0	0	0	551.53	125.555	97.51
0.015	0	0	0.18	3.325	0	0	0	0	66.6	4.14	3.14
0	0	0	22.425	1.705	0	0	0	0	380.495	799.09	108.735
0.81	0	0	14.25	2.93	0	0	0	0	231.17	93.62	13.885
1.485	0	0	0.005	8.465	0	0	0	0	29.555	6.255	0
8.835	0	0	20.05	34.295	0	0	0.495	0	196.37	93.845	27.775
9.35	0	0	8.31	17.855	0	0	0	0	142.74	33.125	40.47
1.015	0	0	6.145	8.29	0	0	0	0	365.175	77.77	87.215
3.625	0	0	4.455	19.885	0	0	0	0	104.8	31.985	48.745
24.485	0	0	57.735	27.18	12.205	0	0	0	39.23	42.435	22.78
8.055	0	0	2.105	1.14	0	0	23.08	0	22.59	13.445	57.85
11.08	0	0	17.67	6.935	0.41	0	0	0	25.555	20.3	16.935
25.83	0	0	54.485	22.3	12.735	0	2.575	0	72.045	48.035	34.9
24.655	0	0	59.7	30.98	13.97	0	4.01	0	46.56	44.36	27.41

liver MIG	liver MIP-1	liver TNFa	liver VEGF	lung GM-C!	lung IFN-g	lung IL-1a	lung IL-1b	lung IL-2	lung IL-4	lung IL-5	lung IL-6	lung IL-10
**Liver**	**Liver**	**Liver**	**Liver**	**Lung**	**Lung**	**Lung**	**Lung**	**Lung**	**Lung**	**Lung**	**Lung**	**Lung**
**liver MIG**	**liver MIP-1**	**liver TNFa**	**liver VEGF**	**ung GM-CS**	**lung IFN-g**	**lung IL-1a**	**lung IL-1b**	**lung IL-2**	**lung IL-4**	**lung IL-5**	**lung IL-6**	**lung IL-10**
1240.805	21.835	2.345	11.105	2.255	3.755	15.595	15.975	1.43	1.59	1.245	13.45	4.085
500.86	101.64	6.145	16.235	2.255	13.66	26.095	27.12	1.905	1.61	1.285	33.79	1.57
636.03	131.275	2.68	66.56	44.8	11.29	2607.165	196.045	3.975	2.04	2.13	1311.715	2.065
24877.6	136.08	4.8	71.745	89.5	103.48	77.605	121.445	12.275	1.32	10.235	734.62	15.645
22265.9	70.185	6.45	63.13	40.185	36.355	51.32	0	13.255	0	5.075	739.96	30.25
13663.32	64.185	0.97	71.01	22.02	53.41	60.21	27.465	7.855	1.07	0	436.285	12.09
1824.88	13.005	0	5.49	0	21.405	41.135	14.355	2.205	0	0	34.5	1.19
1017.09	19.35	2.265	17.465	0	4.61	14	11.355	1.31	1.57	1.22	7.675	4
1108.985	89.11	7.855	17.465	18.08	12.315	26.705	17.705	3.765	1.715	1.295	282.765	1.555
3334.575	188.6	2.465	51.155	48.94	9.615	32.84	87.95	1.505	1.82	3.015	1782.12	1.535
24877.6	117.235	4.235	79.115	67.27	40.36	32.145	62.38	6.19	1.09	3.665	461.035	7.155
22265.9	88.595	7.16	63.5	22.615	38.825	55.97	0	14.375	0	0	349.215	6.605
4761.805	16.75	1.385	11.015	0	52.01	64.055	14.46	6.09	1.105	0.765	11.05	37.735
1289.96	8.025	0	7.7	0	9.25	46.495	16.565	3.24	0	0	14.365	0.805
762.265	4.975	2.09	10.585	0	4.54	14.7	13.155	1.565	1.575	1.275	7.085	3.485
514.81	117.05	6.455	16.285	16.18	10.455	24.735	20.445	2.51	1.705	1.33	174.34	2.455
4181.37	143.59	2.845	51.305	45.62	8.68	23.635	53.52	1.885	1.73	3.4	1647.9	1.57
7957.565	55.585	0	73.86	60.235	38.93	46.145	75.53	10.22	0	13.43	1105.455	39.415
14630.66	88.18	6.545	67.5	44.595	53.72	56.645	0	15.21	0	6.995	978.05	45.415
9064.32	27.29	0	23.38	45.29	17.57	23.96	30.8594	0	0	0	147.275	25.965
2327.3	26.38	0	7.705	2.835	86.555	74.875	15.1	17.985	0	0	24.245	12.37
517.225	12.685	2.21	17.755	1.395	16.97	25.245	15.275	2.48	1.6	1.2	10.22	5.885
569.385	136	8.32	11.21	18.08	13.96	27.105	28.43	2.825	1.73	1.295	180.61	1.685
24877.6	196.635	7.685	85.42	100.09	99.915	99.165	186.555	19.105	1.24	2.775	1314.605	13.76
10817.42	50.335	3.2	94.45	36.97	81.13	155.275	66.2423	19.24	0	9.325	716.98	18.4
10593.14	86.92	6.265	67.235	45.975	73.905	62.795	11.6283	19.295	0	4.105	973.82	58.665

6699.22	27.29	0	15.2	19.79	12.885	19.475	425.292	0	0	0	114.61	9.235
2435.34	26.38	0	9.825	0	32.265	42.17	14.355	5.64	0	0	54.335	3.855
586.77	70.195	2.48	29.575	22.02	69.375	85.685	24.04	15.215	1.07	0.125	17.12	31.225
768.49	191.945	28.89	19.435	69.82	63.375	58.19	73.735	11.175	1.28	0.915	127.415	14.385
24877.64	283.235	7.01	76.36	99.33	32.255	43.875	223.155	5.565	1.38	2.57	1030.785	9.61
8166.815	31.4	0	24.585	33.47	209.965	1006.24	125.413	43.98	0	11.61	45.08	37.145
9879.13	119.9	1.95	59.6	24.2	16.88	24.265	51.705	1.045	0	0	623.715	0.345
9042.265	34.65	0	93.43	29.585	11.86	29.925	58.1498	0	0	0	1408.625	22.41
1057.91	8.025	0	5.67	0	65.26	172.785	14.355	18.49	0	0	15.37	2.205
804.335	173.375	25.515	25.27	47.77	26.035	43.345	45.945	7.69	1.165	0.455	160.185	10.85
24877.6	168.12	5.5	48.335	92.095	110.63	128.025	113.58	24.89	1.32	2.5	1306.005	15.435
9890.885	234.445	3.51	85.4	43.17	56.12	67.85	328.8543	13.78	0	3.89	1258.7	15.13
8830.74	106.13	2.685	88.42	6.795	66.575	57.345	40	13.53	0	0	852.915	0.65
**7249.625**	**17.38**	**0**	**122.42**	**21.115**	**16.465**	**36.465**	**65.8024**	**3.105**	**0**	**0**	**403.06**	**2.675**
**3384.305**	**73.085**	**0**	**11.535**	**0**	**49.455**	**41.21**	**19.465**	**2.73**	**0**	**0**	**50.985**	**0.96**

**Liver**	**Liver**	**Liver**	**Liver**	**Lung**	**Lung**	**Lung**	**Lung**	**Lung**	**Lung**	**Lung**	**Lung**	**Lung**
**LMIG**	**LMIP-1a**	**LTNFa**	**LVEGF**	**UGM-CSF**	**UIFN-g**	**UIL-1a**	**UIL-1b**	**UIL-2**	**UIL-4**	**UIL-5**	**UIL-6**	**UIL-10**
1240.805	21.835	2.345	11.105	2.255	3.755	15.595	15.975	1.43	1.59	1.245	13.45	4.085
1017.09	19.35	2.265	17.465	0	4.61	14	11.355	1.31	1.57	1.22	7.675	4
762.265	4.975	2.09	10.585	0	4.54	14.7	13.155	1.565	1.575	1.275	7.085	3.485
517.225	12.685	2.21	17.755	1.395	16.97	25.245	15.275	2.48	1.6	1.2	10.22	5.885
586.77	70.195	2.48	29.575	22.02	69.375	85.685	24.04	15.215	1.07	0.125	17.12	31.225
**LMIG**	**LMIP-1a**	**LTNFa**	**LVEGF**	**UGM-CSF**	**UIFN-g**	**UIL-1a**	**UIL-1b**	**UIL-2**	**UIL-4**	**UIL-5**	**UIL-6**	**UIL-10**
500.86	101.64	6.145	16.235	2.255	13.66	26.095	27.12	1.905	1.61	1.285	33.79	1.57
636.03	131.275	2.68	66.56	44.8	11.29	2607.165	196.045	3.975	2.04	2.13	1311.715	2.065

24877.6	136.08	4.8	71.745	89.5	103.48	77.605	121.445	12.275	1.32	10.235	734.62	15.645
22265.9	70.185	6.45	63.13	40.185	36.355	51.32	0	13.255	0	5.075	739.96	30.25
13663.32	64.185	0.97	71.01	22.02	53.41	60.21	27.465	7.855	1.07	0	436.285	12.09
1824.88	13.005	0	5.49	0	21.405	41.135	14.355	2.205	0	0	34.5	1.19
1108.985	89.11	7.855	17.465	18.08	12.315	26.705	17.705	3.765	1.715	1.295	282.765	1.555
3334.575	188.6	2.465	51.155	48.94	9.615	32.84	87.95	1.505	1.82	3.015	1782.12	1.535
24877.6	117.235	4.235	79.115	67.27	40.36	32.145	62.38	6.19	1.09	3.665	461.035	7.155
22265.9	88.595	7.16	63.5	22.615	38.825	55.97	0	14.375	0	0	349.215	6.605
4761.805	16.75	1.385	11.015	0	52.01	64.055	14.46	6.09	1.105	0.765	11.05	37.735
1289.96	8.025	0	7.7	0	9.25	46.495	16.565	3.24	0	0	14.365	0.805
514.81	117.05	6.455	16.285	16.18	10.455	24.735	20.445	2.51	1.705	1.33	174.34	2.455
4181.37	143.59	2.845	51.305	45.62	8.68	23.635	53.52	1.885	1.73	3.4	1647.9	1.57
7957.565	55.585	0	73.86	60.235	38.93	46.145	75.53	10.22	0	13.43	1105.455	39.415
14630.66	88.18	6.545	67.5	44.595	53.72	56.645	0	15.21	0	6.995	978.05	45.415
9064.32	27.29	0	23.38	45.29	17.57	23.96	30.8594	0	0	0	147.275	25.965
2327.3	26.38	0	7.705	2.835	86.555	74.875	15.1	17.985	0	0	24.245	12.37
569.385	136	8.32	11.21	18.08	13.96	27.105	28.43	2.825	1.73	1.295	180.61	1.685
24877.6	196.635	7.685	85.42	100.09	99.915	99.165	186.555	19.105	1.24	2.775	1314.605	13.76
10817.42	50.335	3.2	94.45	36.97	81.13	155.275	66.2423	19.24	0	9.325	716.98	18.4
10593.14	86.92	6.265	67.235	45.975	73.905	62.795	11.6283	19.295	0	4.105	973.82	58.665
6699.22	27.29	0	15.2	19.79	12.885	19.475	425.292	0	0	0	114.61	9.235
2435.34	26.38	0	9.825	0	32.265	42.17	14.355	5.64	0	0	54.335	3.855
768.49	191.945	28.89	19.435	69.82	63.375	58.19	73.735	11.175	1.28	0.915	127.415	14.385
24877.64	283.235	7.01	76.36	99.33	32.255	43.875	223.155	5.565	1.38	2.57	1030.785	9.61
8166.815	31.4	0	24.585	33.47	209.965	1006.24	125.413	43.98	0	11.61	45.08	37.145
9879.13	119.9	1.95	59.6	24.2	16.88	24.265	51.705	1.045	0	0	623.715	0.345
9042.265	34.65	0	93.43	29.585	11.86	29.925	58.1498	0	0	0	1408.625	22.41
1057.91	8.025	0	5.67	0	65.26	172.785	14.355	18.49	0	0	15.37	2.205
804.335	173.375	25.515	25.27	47.77	26.035	43.345	45.945	7.69	1.165	0.455	160.185	10.85

24877.6	168.12	5.5	48.335	92.095	110.63	128.025	113.58	24.89	1.32	2.5	1306.005	15.435
9890.885	234.445	3.51	85.4	43.17	56.12	67.85	328.8543	13.78	0	3.89	1258.7	15.13
8830.74	106.13	2.685	88.42	6.795	66.575	57.345	40	13.53	0	0	852.915	0.65
**7249.625**	**17.38**	**0**	**122.42**	**21.115**	**16.465**	**36.465**	**65.8024**	**3.105**	**0**	**0**	**403.06**	**2.675**
**3384.305**	**73.085**	**0**	**11.535**	**0**	**49.455**	**41.21**	**19.465**	**2.73**	**0**	**0**	**50.985**	**0.96**
**liver MIG**	**liver MIP-1**	**liver TNFa**	**liver VEGF**	**ung GM-CS**	**lung IFN-g**	**lung IL-1a**	**lung IL-1b**	**lung IL-2**	**lung IL-4**	**lung IL-5**	**lung IL-6**	**lung IL-10**
**Liver**	**Liver**	**Liver**	**Liver**	**Lung**	**Lung**	**Lung**	**Lung**	**Lung**	**Lung**	**Lung**	**Lung**	**Lung**
**liver MIG**	**liver MIP-1**	**liver TNFa**	**liver VEGF**	**ung GM-CS**	**lung IFN-g**	**lung IL-1a**	**lung IL-1b**	**lung IL-2**	**lung IL-4**	**lung IL-5**	**lung IL-6**	**lung IL-10**
2972.84	87.775	0.72	16.42	0	28.69	31.385	21.6	3.91	0	0	10.48	1.89
2329.1	90.14	3.815	14.85	6.795	32.96	35.66	31.3	5.165	0	0	98.535	0.045
7531.65	24.415	0	15.735	9.31	0	20.85	5.735	0.03	0	0	32.035	4.37
7531.65	0	0	5.3	6.81	2.735	34.405	5.735	1.37	0	0	16.605	3.225
7531.65	0	0	11.045	0	0	16.485	3.825	0.045	0	0	12.005	2.93
641.97	92.34	2.275	9.29	0	1.57	48.05	73.335	15.43	0	0	2.5	2.655
242.795	28.595	2.39	39.76	12.375	9.145	48.8	151.735	20.385	0	0	5.215	1.715
258.035	0	0	10.17	3.015	0	20.85	0.51	0	0	0	2.125	27.47
211.48	0	0	10.505	0	0	5.755	0	0.18	0	0	0.3	5.105
115.315	0	0	6.695	0	0	3.81	0	0	0	0	0.735	2.02
519.215	109.68	6.465	16.295	0	29.025	28.585	86.59	5.38	1.09	0.295	42.395	4.935
675.2	99.535	5.915	16.775	0	80.115	78.895	187.32	13.085	1.05	0.295	57.94	5.135
219.195	13.62	0.755	5.49	5.215	0	8.53	18.485	0.095	0	0	57.63	4.46
4706.28	17.54	0.01	18.225	0	0	12.145	0	0.225	0	0	10.1	1.705
736.42	0	0	7.34	0	0	25.885	0	0.185	0	0	1	2.535
4885.775	0	0	5.42	0	0	8.705	2.205	0.12	0	0	28.735	3.325
3482.65	0	0	2.53	0	0	32.14	6.62	1.01	0	0	16.825	8.465
1950.29	0	0	6.165	0	5.7	255.42	8.31	13.39	0	0	0.045	7.03

7531.65	24.415	0.195	4.81	0	1.9	54.345	5.04	1.43	0	0	14.66	6.455
7531.65	18.67	0	12.385	0	0	27.015	2.205	0.255	0	0	13.72	3.71
7531.65	0	0	21.22	0	0	9.23	0	0.14	0	0	17.395	1.04
7531.65	16.335	0.1	16.085	0	0	19.02	0	0.265	0	0	15.8	0.805
424.895	28.595	3.4	12.75	12.375	2.75	33.93	148.875	27.54	0	0	4.86	1.04
121.285	11.785	1.695	30.05	0	3.4	31.345	171.53	23.42	0	0	3.145	3.02
500.9	5.66	0.72	14.12	12.375	3.185	34.83	228.63	26.715	0	0	3.21	3.75
466.74	40.025	2.84	24.915	0	4.225	38.865	111.315	20.65	0	0	3.52	3.57
270.075	35.77	2.045	24.72	17.21	4.625	43.945	201.705	38.85	0	0	4.38	3.57

				Pg/Mg								
Liver	Liver	Liver	Liver	Lung	Lung	Lung	Lung	Lung	Lung	Lung	Lung	Lung
LMIG	LMIP-1a	LTNFa	LVEGF	UGM-CSF	UIFN-g	UIL-1a	UIL-1b	UIL-2	UIL-4	UIL-5	UIL-6	UIL-10
2972.84	87.775	0.72	16.42	0	28.69	31.385	21.6	3.91	0	0	10.48	1.89
258.035	0	0	10.17	3.015	0	20.85	0.51	0	0	0	2.125	27.47
211.48	0	0	10.505	0	0	5.755	0	0.18	0	0	0.3	5.105
115.315	0	0	6.695	0	0	3.81	0	0	0	0	0.735	2.02
LMIG	LMIP-1a	LTNFa	LVEGF	UGM-CSF	UIFN-g	UIL-1a	UIL-1b	UIL-2	UIL-4	UIL-5	UIL-6	UIL-10
2329.1	90.14	3.815	14.85	6.795	32.96	35.66	31.3	5.165	0	0	98.535	0.045
7531.65	24.415	0	15.735	9.31	0	20.85	5.735	0.03	0	0	32.035	4.37
7531.65	0	0	5.3	6.81	2.735	34.405	5.735	1.37	0	0	16.605	3.225
7531.65	0	0	11.045	0	0	16.485	3.825	0.045	0	0	12.005	2.93
641.97	92.34	2.275	9.29	0	1.57	48.05	73.335	15.43	0	0	2.5	2.655
242.795	28.595	2.39	39.76	12.375	9.145	48.8	151.735	20.385	0	0	5.215	1.715
519.215	109.68	6.465	16.295	0	29.025	28.585	86.59	5.38	1.09	0.295	42.395	4.935

675.2	99.535	5.915	16.775	0	80.115	78.895	187.32	13.085	1.05	0.295	57.94	5.135
219.195	13.62	0.755	5.49	5.215	0	8.53	18.485	0.095	0	0	57.63	4.46
4706.28	17.54	0.01	18.225	0	0	12.145	0	0.225	0	0	10.1	1.705
736.42	0	0	7.34	0	0	25.885	0	0.185	0	0	1	2.535
4885.775	0	0	5.42	0	0	8.705	2.205	0.12	0	0	28.735	3.325
3482.65	0	0	2.53	0	0	32.14	6.62	1.01	0	0	16.825	8.465
1950.29	0	0	6.165	0	5.7	255.42	8.31	13.39	0	0	0.045	7.03
7531.65	24.415	0.195	4.81	0	1.9	54.345	5.04	1.43	0	0	14.66	6.455
7531.65	18.67	0	12.385	0	0	27.015	2.205	0.255	0	0	13.72	3.71
7531.65	0	0	21.22	0	0	9.23	0	0.14	0	0	17.395	1.04
7531.65	16.335	0.1	16.085	0	0	19.02	0	0.265	0	0	15.8	0.805
424.895	28.595	3.4	12.75	12.375	2.75	33.93	148.875	27.54	0	0	4.86	1.04
121.285	11.785	1.695	30.05	0	3.4	31.345	171.53	23.42	0	0	3.145	3.02
500.9	5.66	0.72	14.12	12.375	3.185	34.83	228.63	26.715	0	0	3.21	3.75
466.74	40.025	2.84	24.915	0	4.225	38.865	111.315	20.65	0	0	3.52	3.57
270.075	35.77	2.045	24.72	17.21	4.625	43.945	201.705	38.85	0	0	4.38	3.57

lung IL-12 (	lung IL-12 (	lung IL-13	lung IL-17	lung IP-10	lung KC	lung MCP-1	lung MIG	lung MIP-1	lung TNFa	lung VEGF	gut GM-CS	gut IFN-g
**Lung**	**Lung**	**Lung**	**Lung**	**Lung**	**Lung**	**Lung**	**Lung**	**Lung**	**Lung**	**Lung**	**gut**	**gut**
**ng IL-12 (p4**	**ng IL-12 (p7**	**lung IL-13**	**lung IL-17**	**lung IP-10**	**lung KC**	**lung MCP-1**	**lung MIG**	**lung MIP-1a**	**lung TNFa**	**lung VEGF**	**gut GM-CS**	**gut IFN-g**
2.35	0.26	10.925	1.455	511.415	32.7	72.87	779.955	8.035	2.09	563.5	0	7.175
4.585	0.53	3.775	1.385	201.34	1315.53	436.05	102.605	320.495	2.745	548.53	0	2.185
5.21	3.685	52.545	1.765	6381.32	4186.805	2307.52	5521.375	930.04	2.88	1090.19	12.49	3.455
23.68	15.905	219.43	1.845	5000.0	5000.0	1691.585	24877.6	558.7	6.395	937.94	64.57	49.555
18.335	8	16.64	2.72	4982.09	3467.135	973.295	17871.34	101.525	1.53	609.17	36.97	13.335
9.46	0	119.205	0	1593.725	2038.19	1038.905	24877.6	163.395	1.865	298.01	69.82	22.945
2.535	0.74	7.715	0.665	810.915	391.34	644.3	3767.555	45.84	0	275.355	2.835	5.32
2.12	0	4.475	1.34	269.32	40.325	77.16	280.585	4.01	2.09	427	0	0
2.12	1.085	15.34	1.4	667.93	1578.995	676.62	1316.81	381.02	3.71	896.725	0	2.75
5.145	2.66	31.845	1.605	5721.665	4594.55	1958.425	4288.98	948.895	2.87	470.295	5.595	6.13
14.73	0	0	0	5000.0	2011.295	1227.74	24877.6	370.615	4.09	823.31	69.82	19.63
9.85	6.26	0	0	2844.78	3184.475	857.175	9242.475	76.54	0	874.07	28.535	17.57
12.805	0	126.985	0	539.8	111.085	145.905	2875.95	37.105	1.385	395.85	0	4.145
3.28	0	2.59	0.55	418.22	95.01	258.12	2559.675	35.49	0	340.73	16.89	2.005
2.09	0	7.015	1.39	115.8	8.875	35.805	493.625	6.435	2.065	482.76	0	8.025
4.3	1.085	9.365	1.41	726.36	1127.13	468.315	1606.345	346.345	6.925	599.65	3.57	4.395
4.395	1.65	46.345	1.895	4334.685	4198.465	1765.51	5143.765	813.69	2.9	898.73	4.645	24.02
20.52	8	5.165	5.725	5745.195	5156.34	1458.505	9330.48	344.43	7.415	591.525	36.125	12.43
22.22	9.085	16.295	9.825	4652.455	4970.88	1350.5	22265.9	142.395	1.53	730.71	28.535	15.58
8.82	7.135	0	0	1940.975	1318.885	1007.37	16878.53	58.635	0	224.275	16.52	10.83
2.33	0	4.56	0.215	631.93	338	564.505	3602.405	40.78	0	187.825	2.835	2.835
3.265	0.005	8.795	1.4	83.555	6.945	21.145	193.32	5.28	2.09	384.695	0	2.13
2.815	0.945	11.005	1.415	690.585	1319.2	610.305	440.795	396.53	4.445	917.265	0	1.705
24.02	9.62	255.525	1.06	5000.0	5000.0	1897.045	24877.6	823.66	7.955	855.16	45.595	17.695
14.435	7.57	41.365	0	4696.4	2813.79	1097.305	8027.43	134.545	2.06	556.165	16.52	11.63
27.865	8.435	32.125	2.245	4581.14	4990.685	1373.405	22265.9	186.94	5.04	704.53	35.265	11.29

3.255	7.57	0	0	1196.335	883.475	982.675	9134.755	87.34	0	573.19	16.52	12.885
2.33	0	3.4	0.245	772.865	496.295	747.74	4301.91	41.81	0	309.745	0	3.665
12.595	0	182.99	0	141.8	56.805	107.765	495.95	81.025	1.055	748.29	37.985	13.395
18.215	14.75	145.395	1.06	625.4	1163.47	457.37	618.795	411.125	28.765	907.455	0	20.315
17.995	8.14	47.065	0.815	5000.0	5000.0	1528.835	14597.66	715.27	6.875	816.49	55.35	27.415
15.035	7.135	9.73	0	1383.28	208.935	423.965	5257.545	30.735	0	859.32	31.585	5.12
10.97	0	0	3.375	15949.19	4361.525	1258.42	10143.45	344.76	1.755	1357.095	13.565	6.495
8.555	7.46	0	10.13	3800.52	4943.47	1240.6	22265.9	147.41	0	599.485	34.38	8.97
1.715	0	11.335	0.295	433.73	73.285	212.195	2461.48	56.355	0	452.745	0	0
9.975	3.655	86.46	0	667.66	1179.06	429.395	249.675	247.655	12.825	499.495	0	17.355
22.78	6.525	280.83	0	5000.0	5000.0	1810.93	24877.6	631.41	7.415	518.435	43.26	12.155
13.345	7.135	0	7.495	6511.23	5244.425	1607.955	22265.9	505.7	3.51	721.205	21.245	10.83
8.47	0.975	26.02	1.8	7029.765	5192.055	1161.84	11131.6	151.21	1.755	343.13	0	1.585
**2.635**	**5.16**	**0**	**2.86**	**1893.33**	**4071.805**	**1209.2**	**10771.06**	**110**	**0**	**399.24**	**36.97**	**9.685**
**4.31**	**0**	**0**	**0.825**	**613.965**	**389.32**	**774.3**	**4826.865**	**52.145**	**0**	**411.58**	**0**	**2.125**
**Lung**	**Lung**	**Lung**	**Lung**	**Lung**	**Lung**	**Lung**	**Lung**	**Lung**	**Lung**	**Lung**	**gut**	**gut**
**UIL-12 (p40)**	**UIL-12 (p70)**	**UIL-13**	**UIL-17**	**UIP-10**	**UKC**	**UMCP-1**	**UMIG**	**UMIP-1a**	**UTNFa**	**UVEGF**	**gGM-CSF**	**gIFN-g**
2.35	0.26	10.925	1.455	511.415	32.7	72.87	779.955	8.035	2.09	563.5	0	7.175
2.12	0	4.475	1.34	269.32	40.325	77.16	280.585	4.01	2.09	427	0	0
2.09	0	7.015	1.39	115.8	8.875	35.805	493.625	6.435	2.065	482.76	0	8.025
3.265	0.005	8.795	1.4	83.555	6.945	21.145	193.32	5.28	2.09	384.695	0	2.13
12.595	0	182.99	0	141.8	56.805	107.765	495.95	81.025	1.055	748.29	37.985	13.395
**UIL-12 (p40)**	**UIL-12 (p70)**	**UIL-13**	**UIL-17**	**UIP-10**	**UKC**	**UMCP-1**	**UMIG**	**UMIP-1a**	**UTNFa**	**UVEGF**	**gGM-CSF**	**gIFN-g**
4.585	0.53	3.775	1.385	201.34	1315.53	436.05	102.605	320.495	2.745	548.53	0	2.185
5.21	3.685	52.545	1.765	6381.32	4186.805	2307.52	5521.375	930.04	2.88	1090.19	12.49	3.455

23.68	15.905	219.43	1.845	5000.0	5000.0	1691.585	24877.6	558.7	6.395	937.94	64.57	49.555
18.335	8	16.64	2.72	4982.09	3467.135	973.295	17871.34	101.525	1.53	609.17	36.97	13.335
9.46	0	119.205	0	1593.725	2038.19	1038.905	24877.6	163.395	1.865	298.01	69.82	22.945
2.535	0.74	7.715	0.665	810.915	391.34	644.3	3767.555	45.84	0	275.355	2.835	5.32
2.12	1.085	15.34	1.4	667.93	1578.995	676.62	1316.81	381.02	3.71	896.725	0	2.75
5.145	2.66	31.845	1.605	5721.665	4594.55	1958.425	4288.98	948.895	2.87	470.295	5.595	6.13
14.73	0	0	0	5000.0	2011.295	1227.74	24877.6	370.615	4.09	823.31	69.82	19.63
9.85	6.26	0	0	2844.78	3184.475	857.175	9242.475	76.54	0	874.07	28.535	17.57
12.805	0	126.985	0	539.8	111.085	145.905	2875.95	37.105	1.385	395.85	0	4.145
3.28	0	2.59	0.55	418.22	95.01	258.12	2559.675	35.49	0	340.73	16.89	2.005
4.3	1.085	9.365	1.41	726.36	1127.13	468.315	1606.345	346.345	6.925	599.65	3.57	4.395
4.395	1.65	46.345	1.895	4334.685	4198.465	1765.51	5143.765	813.69	2.9	898.73	4.645	24.02
20.52	8	5.165	5.725	5745.195	5156.34	1458.505	9330.48	344.43	7.415	591.525	36.125	12.43
22.22	9.085	16.295	9.825	4652.455	4970.88	1350.5	22265.9	142.395	1.53	730.71	28.535	15.58
8.82	7.135	0	0	1940.975	1318.885	1007.37	16878.53	58.635	0	224.275	16.52	10.83
2.33	0	4.56	0.215	631.93	338	564.505	3602.405	40.78	0	187.825	2.835	2.835
2.815	0.945	11.005	1.415	690.585	1319.2	610.305	440.795	396.53	4.445	917.265	0	1.705
24.02	9.62	255.525	1.06	5000.0	5000.0	1897.045	24877.6	823.66	7.955	855.16	45.595	17.695
14.435	7.57	41.365	0	4696.4	2813.79	1097.305	8027.43	134.545	2.06	556.165	16.52	11.63
27.865	8.435	32.125	2.245	4581.14	4990.685	1373.405	22265.9	186.94	5.04	704.53	35.265	11.29
3.255	7.57	0	0	1196.335	883.475	982.675	9134.755	87.34	0	573.19	16.52	12.885
2.33	0	3.4	0.245	772.865	496.295	747.74	4301.91	41.81	0	309.745	0	3.665
18.215	14.75	145.395	1.06	625.4	1163.47	457.37	618.795	411.125	28.765	907.455	0	20.315
17.995	8.14	47.065	0.815	5000.0	5000.0	1528.835	14597.66	715.27	6.875	816.49	55.35	27.415
15.035	7.135	9.73	0	1383.28	208.935	423.965	5257.545	30.735	0	859.32	31.585	5.12
10.97	0	0	3.375	15949.19	4361.525	1258.42	10143.45	344.76	1.755	1357.095	13.565	6.495
8.555	7.46	0	10.13	3800.52	4943.47	1240.6	22265.9	147.41	0	599.485	34.38	8.97
1.715	0	11.335	0.295	433.73	73.285	212.195	2461.48	56.355	0	452.745	0	0
9.975	3.655	86.46	0	667.66	1179.06	429.395	249.675	247.655	12.825	499.495	0	17.355

22.78	6.525	280.83	0	5000.0	5000.0	1810.93	24877.6	631.41	7.415	518.435	43.26	12.155
13.345	7.135	0	7.495	6511.23	5244.425	1607.955	22265.9	505.7	3.51	721.205	21.245	10.83
8.47	0.975	26.02	1.8	7029.765	5192.055	1161.84	11131.6	151.21	1.755	343.13	0	1.585
**2.635**	**5.16**	**0**	**2.86**	**1893.33**	**4071.805**	**1209.2**	**10771.06**	**110**	**0**	**399.24**	**36.97**	**9.685**
**4.31**	**0**	**0**	**0.825**	**613.965**	**389.32**	**774.3**	**4826.865**	**52.145**	**0**	**411.58**	**0**	**2.125**
**ng IL-12 (p4**	**ng IL-12 (p7**	**lung IL-13**	**lung IL-17**	**lung IP-10**	**lung KC**	**lung MCP-1**	**lung MIG**	**lung MIP-1a**	**lung TNFa**	**lung VEGF**	**gut GM-CS**	**gut IFN-g**
**Lung**	**Lung**	**Lung**	**Lung**	**Lung**	**Lung**	**Lung**	**Lung**	**Lung**	**Lung**	**Lung**	**gut**	**gut**
**ng IL-12 (p4**	**ng IL-12 (p7**	**lung IL-13**	**lung IL-17**	**lung IP-10**	**lung KC**	**lung MCP-1**	**lung MIG**	**lung MIP-1a**	**lung TNFa**	**lung VEGF**	**gut GM-CS**	**gut IFN-g**
2.96	3.43	6.92	0.515	859.11	110.335	23.015	3442.13	18.755	0	377.445	0	6.025
8.825	23.78	18.765	0.875	572.7	916.51	75.585	2072.575	77.26	17.165	699.73	0	16.995
8.55	0	0	0	440.175	2785.99	873.305	7531.65	118.7	0.32	443.86	109.135	75.07
0	0	0	0	288.39	1073.51	285.92	7531.65	42.28	0.185	422.64	20.805	13.1
0	0	0	0	345.37	300.39	1056.54	7531.65	37.975	0	205.96	0	0
0	0	25.17	1.025	70.205	34.45	45.17	325.61	11.785	0.805	275.635	12.375	6.425
1.02	0	101.115	2.755	76.42	56.525	50.13	190.14	22.985	0.505	525.665	0	0.435
0	0	0	0	227.53	100.32	67.11	3676.005	0	0	429.87	0	0
0	0	0	0	51.125	11.22	13.885	199.775	0	0	204.895	0	0.64
0	0	0	0	67.05	16.33	39.56	189.175	0	0	188.69	0	0
15.81	11.005	58.675	0	472.845	1254.645	380.16	1258.55	146.945	10.61	588.79	0	24.43
16.9	0	191.82	0	404.45	1596.715	565.57	578.76	198.94	14.375	577.545	0	12.605
4.515	0	0	0	384.575	2155.93	507.65	422.53	178.365	6.83	210.5	0	0
1.38	0	0	0	356.07	936.555	358.675	1314.195	68.945	0.01	169.395	0	0
0	0	0	0	130.4	35.12	100.635	423.34	0	0	266.575	0	0
0.63	0	0	0.34	325.08	2413.39	572.2	2508.19	54.98	0.01	248.205	306.83	175.475
0	0	0	0	238.955	513.83	98.92	1269.26	10.23	0	104.44	26.36	13.33
0	0	0	0	47.635	5.05	11.665	223.02	0	0	152.315	12.375	26.5

0	0	0	0	317.56	925.605	216.035	1132.735	24.415	0.045	237.205	0	10.47
0	0	0	1.915	302.4	315.5	724.47	7531.65	27.625	0	225.68	0	0.25
0	0	0	0.695	387.295	260.855	555.425	7531.65	20.755	0	130.095	0	18.82
0	0	0	0	188.87	266.755	484.82	7531.65	21.725	0	158.045	0	1.82
0	0	73.74	7.33	82.78	46.6	65.62	303.46	28.595	2.045	365.405	34.555	0.435
0.615	0	88.43	2.92	98.135	51.635	80.585	925.435	33.5	1.93	320.23	0	0
2.385	0	175.535	3.3	71.71	50.825	78.275	666.785	22.985	0.59	409.585	12.375	0.9
1.22	0	92.76	2.29	145.22	69.095	52.445	236.305	24.475	1.495	397.87	0	14.33
5.375	0	220.59	2.72	65.755	73.64	41.78	217.75	28.595	0.505	584.7	0	37.71
											**Pg/Mg**	
**Lung**	**Lung**	**Lung**	**Lung**	**Lung**	**Lung**	**Lung**	**Lung**	**Lung**	**Lung**	**Lung**	**gut**	**gut**
**UIL-12 (p40**	**UIL-12 (p70**	**UIL-13**	**UIL-17**	**UIP-10**	**UKC**	**UMCP-1**	**UMIG**	**UMIP-1a**	**UTNFa**	**UVEGF**	**gGM-CSF**	**gIFN-g**
2.96	3.43	6.92	0.515	859.11	110.335	23.015	3442.13	18.755	0	377.445	0	6.025
0	0	0	0	227.53	100.32	67.11	3676.005	0	0	429.87	0	0
0	0	0	0	51.125	11.22	13.885	199.775	0	0	204.895	0	0.64
0	0	0	0	67.05	16.33	39.56	189.175	0	0	188.69	0	0
**UIL-12 (p40**	**UIL-12 (p70**	**UIL-13**	**UIL-17**	**UIP-10**	**UKC**	**UMCP-1**	**UMIG**	**UMIP-1a**	**UTNFa**	**UVEGF**	**gGM-CSF**	**gIFN-g**
8.825	23.78	18.765	0.875	572.7	916.51	75.585	2072.575	77.26	17.165	699.73	0	16.995
8.55	0	0	0	440.175	2785.99	873.305	7531.65	118.7	0.32	443.86	109.135	75.07
0	0	0	0	288.39	1073.51	285.92	7531.65	42.28	0.185	422.64	20.805	13.1
0	0	0	0	345.37	300.39	1056.54	7531.65	37.975	0	205.96	0	0
0	0	25.17	1.025	70.205	34.45	45.17	325.61	11.785	0.805	275.635	12.375	6.425
1.02	0	101.115	2.755	76.42	56.525	50.13	190.14	22.985	0.505	525.665	0	0.435
15.81	11.005	58.675	0	472.845	1254.645	380.16	1258.55	146.945	10.61	588.79	0	24.43

16.9	0	191.82	0	404.45	1596.715	565.57	578.76	198.94	14.375	577.545	0	12.605
4.515	0	0	0	384.575	2155.93	507.65	422.53	178.365	6.83	210.5	0	0
1.38	0	0	0	356.07	936.555	358.675	1314.195	68.945	0.01	169.395	0	0
0	0	0	0	130.4	35.12	100.635	423.34	0	0	266.575	0	0
0.63	0	0	0.34	325.08	2413.39	572.2	2508.19	54.98	0.01	248.205	306.83	175.475
0	0	0	0	238.955	513.83	98.92	1269.26	10.23	0	104.44	26.36	13.33
0	0	0	0	47.635	5.05	11.665	223.02	0	0	152.315	12.375	26.5
0	0	0	0	317.56	925.605	216.035	1132.735	24.415	0.045	237.205	0	10.47
0	0	0	1.915	302.4	315.5	724.47	7531.65	27.625	0	225.68	0	0.25
0	0	0	0.695	387.295	260.855	555.425	7531.65	20.755	0	130.095	0	18.82
0	0	0	0	188.87	266.755	484.82	7531.65	21.725	0	158.045	0	1.82
0	0	73.74	7.33	82.78	46.6	65.62	303.46	28.595	2.045	365.405	34.555	0.435
0.615	0	88.43	2.92	98.135	51.635	80.585	925.435	33.5	1.93	320.23	0	0
2.385	0	175.535	3.3	71.71	50.825	78.275	666.785	22.985	0.59	409.585	12.375	0.9
1.22	0	92.76	2.29	145.22	69.095	52.445	236.305	24.475	1.495	397.87	0	14.33
5.375	0	220.59	2.72	65.755	73.64	41.78	217.75	28.595	0.505	584.7	0	37.71

gut IL-1a	gut IL-1b	gut IL-2	gut IL-4	gut IL-5	gut IL-6	gut IL-10	gut IL-12 (p	gut IL-12 (p70)	gut IL-13	gut IL-17	gut IP-10	gut KC
**gut**	**gut**	**gut**	**gut**	**gut**	**gut**	**gut**	**gut**	**gut**	**gut**	**gut**	**gut**	**gut**
gut IL-1a	gut IL-1b	gut IL-2	gut IL-4	gut IL-5	gut IL-6	gut IL-10	gut IL-12 (p	gut IL-12 (p70)	gut IL-13	gut IL-17	gut IP-10	gut KC
18.195	8.035	1.835	1.63	1.145	2.565	2.025	1.895	1.795	0.78	1.34	89.67	8.335
7.175	2.885	1.22	1.55	1.145	76.395	1.245	1.06	0	0	1.28	103.205	274.23
17.915	26.13	1.51	1.725	1.74	420.575	1.425	4.57	0.665	19.33	2.73	2268.605	1079.415
97.625	40.955	28.415	1.125	0.765	292.97	21.565	17.775	9.62	286.74	0	825.17	1177.4
28.955	20.2437	7.005	0	0	389.615	59.025	16.93	8.87	0	4.485	3533.57	780.17
823.91	30.87	8.595	1.205	0.915	335.985	31.655	19.76	15.33	411.63	0	1357.85	799.53
24.425	27.195	3.875	0	0	8.63	4.25	10.25	31.195	10.22	0.75	195.18	39.775
2.43	1.795	1.395	1.575	1.125	0.93	1.795	1.645	1.58	0	1.42	42.54	4.205
10.21	8.035	1.215	1.79	1.22	380.425	1.315	5.18	0	0.1	1.31	797.045	2143.89
14.465	23.475	2.61	1.58	1.295	193.58	1.89	4.25	1.935	23.495	1.545	2167.49	822.305
36.96	24.9	5.135	1.185	1.36	526.56	26.915	24.475	21.19	197.315	0	5000.0	1279.115
38.275	26.3774	17.365	0	0	161.685	54.28	18.565	7.785	0	2.845	660.64	489.04
47.54	2.55	3.305	0.985	0	3.625	9.61	6.525	0	104.25	0	80.07	19.12
22.57	12.86	0.54	0	0	0.81	1.735	4.31	2.26	1.695	0.205	72.73	28.745
12.325	4.13	1.9	1.6	1.135	1.55	3.19	2.005	0.945	0.85	1.32	35.64	2.395
12.125	14.57	1.665	1.695	1.23	261.78	1.665	4.425	0.395	0.64	1.31	993.75	2005.5
104.51	18.395	16.365	1.59	1.405	366.445	1.46	3.815	0.945	16.605	1.505	2910.77	1447.77
20.86	21.7958	3.005	0	0	329.095	81.935	19.49	6.915	0	0	2291.525	1234.62
28.34	16.0107	10.56	0	0	208.445	48.755	8.03	7.35	0	2.76	1533.11	739.16
6.14	15.4795	0	0	0	4.7	10.945	7.5	7.675	0	0	100.28	53.825
73.95	10.58	3.38	0	0	0.13	1.735	7.305	9.64	22.72	0.165	64.23	19.86
4.335	2.455	1.8	1.715	1.125	0.865	1.95	1.84	2.37	0	1.285	33.165	2.81
7.985	7.275	1.21	1.69	1.2	267.5	1.425	4.19	0	0	1.295	812.62	2467.975
325.035	46.775	12.885	1.105	0.295	345.665	14.595	29.28	4.7	222.975	0	871.66	1210.995
42.905	16.5502	7.31	0	0	377.045	53.55	12.115	6.04	0	0	2522.12	1064.665
25.445	21.2812	8.295	0	0	277.13	69.49	20.86	7.785	0	3.045	1325.415	918.1

43.42	15.4795	9.37	0	0	35.39	48.385	10.99	7.135	46.59	0	362.78	140.96
43.36	18.385	5.695	0	0	2.425	4.405	8.06	23.92	24.235	0.5	72.065	18.395

83.755	22.315	5.775	1.205	0.295	1.7	13.55	13.87	2.445	123.835	0	67.91	14.845
75.295	46.775	8.225	1.105	0.61	86.035	16.695	13.98	43.425	289.545	0	370.59	1049.15
49.01	36.775	6.04	1.24	0.765	748.88	17.96	40.68	37.51	208.775	0	5000.0	1528.44
23.78	22.3104	3.96	0	3.665	622.18	19.205	13.585	7.35	0	0.59	2962.275	1338.26
30.875	15.835	1.575	0	0	232.3	6.78	8.94	4.18	6.23	1.895	3090.86	1228.33
12.665	3.2619	0	0	0	532.05	23.995	5.865	7.135	0	0	1103.445	1848.62
16.045	17.295	0.505	0	0	0.97	1.425	1.87	10.125	0	0.95	156.585	19.375

107.675	17.1	7.04	1.185	0	107.175	11.675	13.87	0	266.135	0	302.28	1242.26
53.24	25.755	5.74	1.125	0.765	759.61	17.54	21.99	45.55	261.935	0	5000.0	1495.93
27.54	16.0107	7.435	0	0	49.405	21.615	9.335	7.35	0	0	344.15	111.485
18.29	8.24	1.97	0	0	211.64	1.735	2.485	0	0	0.565	2587.28	734.835
**24.635**	**13.57**	**5.145**	**0**	**0**	**168.83**	**45.415**	**14.085**	**7.025**	**0**	**0**	**744.025**	**571.63**
**22.325**	**8.635**	**1.05**	**0**	**0**	**0.535**	**0.5**	**1.615**	**1.425**	**0**	**0.11**	**150.78**	**30.645**

**gut**	**gut**	**gut**	**gut**	**gut**	**gut**	**gut**	**gut**	**gut**	**gut**	**gut**	**gut**	**gut**
**gIL-1a**	**gIL-1b**	**gIL-2**	**gIL-4**	**gIL-5**	**gIL-6**	**gIL-10**	**gIL-12 (p40)**	**gIL-12 (p70)**	**gIL-13**	**gIL-17**	**gIP-10**	**gKC**
18.195	8.035	1.835	1.63	1.145	2.565	2.025	1.895	1.795	0.78	1.34	89.67	8.335
2.43	1.795	1.395	1.575	1.125	0.93	1.795	1.645	1.58	0	1.42	42.54	4.205
12.325	4.13	1.9	1.6	1.135	1.55	3.19	2.005	0.945	0.85	1.32	35.64	2.395
4.335	2.455	1.8	1.715	1.125	0.865	1.95	1.84	2.37	0	1.285	33.165	2.81
83.755	22.315	5.775	1.205	0.295	1.7	13.55	13.87	2.445	123.835	0	67.91	14.845

**gIL-1a**	**gIL-1b**	**gIL-2**	**gIL-4**	**gIL-5**	**gIL-6**	**gIL-10**	**gIL-12 (p40)**	**gIL-12 (p70)**	**gIL-13**	**gIL-17**	**gIP-10**	**gKC**
7.175	2.885	1.22	1.55	1.145	76.395	1.245	1.06	0	0	1.28	103.205	274.23
17.915	26.13	1.51	1.725	1.74	420.575	1.425	4.57	0.665	19.33	2.73	2268.605	1079.415

97.625	40.955	28.415	1.125	0.765	292.97	21.565	17.775	9.62	286.74	0	825.17	1177.4
28.955	20.2437	7.005	0	0	389.615	59.025	16.93	8.87	0	4.485	3533.57	780.17
823.91	30.87	8.595	1.205	0.915	335.985	31.655	19.76	15.33	411.63	0	1357.85	799.53
24.425	27.195	3.875	0	0	8.63	4.25	10.25	31.195	10.22	0.75	195.18	39.775
10.21	8.035	1.215	1.79	1.22	380.425	1.315	5.18	0	0.1	1.31	797.045	2143.89
14.465	23.475	2.61	1.58	1.295	193.58	1.89	4.25	1.935	23.495	1.545	2167.49	822.305
36.96	24.9	5.135	1.185	1.36	526.56	26.915	24.475	21.19	197.315	0	5000.0	1279.115
38.275	26.3774	17.365	0	0	161.685	54.28	18.565	7.785	0	2.845	660.64	489.04
47.54	2.55	3.305	0.985	0	3.625	9.61	6.525	0	104.25	0	80.07	19.12
22.57	12.86	0.54	0	0	0.81	1.735	4.31	2.26	1.695	0.205	72.73	28.745
12.125	14.57	1.665	1.695	1.23	261.78	1.665	4.425	0.395	0.64	1.31	993.75	2005.5
104.51	18.395	16.365	1.59	1.405	366.445	1.46	3.815	0.945	16.605	1.505	2910.77	1447.77
20.86	21.7958	3.005	0	0	329.095	81.935	19.49	6.915	0	0	2291.525	1234.62
28.34	16.0107	10.56	0	0	208.445	48.755	8.03	7.35	0	2.76	1533.11	739.16
6.14	15.4795	0	0	0	4.7	10.945	7.5	7.675	0	0	100.28	53.825
73.95	10.58	3.38	0	0	0.13	1.735	7.305	9.64	22.72	0.165	64.23	19.86
7.985	7.275	1.21	1.69	1.2	267.5	1.425	4.19	0	0	1.295	812.62	2467.975
325.035	46.775	12.885	1.105	0.295	345.665	14.595	29.28	4.7	222.975	0	871.66	1210.995
42.905	16.5502	7.31	0	0	377.045	53.55	12.115	6.04	0	0	2522.12	1064.665
25.445	21.2812	8.295	0	0	277.13	69.49	20.86	7.785	0	3.045	1325.415	918.1
43.42	15.4795	9.37	0	0	35.39	48.385	10.99	7.135	46.59	0	362.78	140.96
43.36	18.385	5.695	0	0	2.425	4.405	8.06	23.92	24.235	0.5	72.065	18.395
75.295	46.775	8.225	1.105	0.61	86.035	16.695	13.98	43.425	289.545	0	370.59	1049.15
49.01	36.775	6.04	1.24	0.765	748.88	17.96	40.68	37.51	208.775	0	5000.0	1528.44
23.78	22.3104	3.96	0	3.665	622.18	19.205	13.585	7.35	0	0.59	2962.275	1338.26
30.875	15.835	1.575	0	0	232.3	6.78	8.94	4.18	6.23	1.895	3090.86	1228.33
12.665	3.2619	0	0	0	532.05	23.995	5.865	7.135	0	0	1103.445	1848.62
16.045	17.295	0.505	0	0	0.97	1.425	1.87	10.125	0	0.95	156.585	19.375
107.675	17.1	7.04	1.185	0	107.175	11.675	13.87	0	266.135	0	302.28	1242.26

53.24	25.755	5.74	1.125	0.765	759.61	17.54	21.99	45.55	261.935	0	5000.0	1495.93
27.54	16.0107	7.435	0	0	49.405	21.615	9.335	7.35	0	0	344.15	111.485
18.29	8.24	1.97	0	0	211.64	1.735	2.485	0	0	0.565	2587.28	734.835
**24.635**	**13.57**	**5.145**	**0**	**0**	**168.83**	**45.415**	**14.085**	**7.025**	**0**	**0**	**744.025**	**571.63**
**22.325**	**8.635**	**1.05**	**0**	**0**	**0.535**	**0.5**	**1.615**	**1.425**	**0**	**0.11**	**150.78**	**30.645**
**gut IL-1a**	**gut IL-1b**	**gut IL-2**	**gut IL-4**	**gut IL-5**	**gut IL-6**	**gut IL-10**	**gut IL-12 (p**	**gut IL-12 (p70)**	**gut IL-13**	**gut IL-17**	**gut IP-10**	**gut KC**
**gut**	**gut**	**gut**	**gut**	**gut**	**gut**	**gut**	**gut**	**gut**	**gut**	**gut**	**gut**	**gut**
**gut IL-1a**	**gut IL-1b**	**gut IL-2**	**gut IL-4**	**gut IL-5**	**gut IL-6**	**gut IL-10**	**gut IL-12 (p**	**gut IL-12 (p70)**	**gut IL-13**	**gut IL-17**	**gut IP-10**	**gut KC**
57.685	6.225	2.29	0	0	4.655	2.99	5.03	3.43	15.62	0.165	111.485	23.48
31.345	12.105	2.5	0	0	19.845	0.195	6.1	10.92	26.285	0.27	550.45	346.16
566.7	152.44	33.965	24.325	39.48	13.825	255.19	37.385	178.53	1013.515	99.925	452.96	1263.29
39.1	22.53	5.715	0	0.525	550.67	1.62	6.93	0	0	16.33	570.595	538.905
16.74	13.19	1.505	0	2.34	85.98	0.54	2.575	0	0	11.685	195.14	202.675
21.83	82.66	7.975	0	0	2.24	1.04	0	0	25.89	16.93	110.68	22.705
13.77	13.19	2.785	0	0	1.97	0.94	4.545	0	0	25.245	117.905	11.78
0.545	0	0	0	0	0.895	3.71	5.42	0	0	9.99	77.92	12.055
2.785	0	0	0	0	0.765	5.65	9.765	0	0	2.07	73.385	2.84
3.75	0.91	0	0	0	0.835	5.2	6.005	0	0	4.695	114.165	7.325
69.04	22.315	9.035	1.03	0.125	93.305	12.51	12.595	0	171.385	0	375.635	741.77
25.725	6.335	6.115	0.96	0	32.93	7.56	7.425	0	86.46	0	249.43	671.665
4.895	1.595	0	0	0	24.945	4.18	6.155	0	0	9.095	302.675	968.67
8.185	0	0.02	0	0	4.255	1.265	2.545	0	0	7.655	370.995	150.3
9.285	3.825	0	0	0	11.24	2.735	0.135	0	0	0	497.7	468.79
1564.28	741.235	67.94	80.235	190.14	23.585	506.24	100.36	445.89	2756.705	576.265	393.37	1669.055
27.925	17.32	6.935	0	0	43.02	2.84	18.67	10.25	0	0	117.34	175.805
95.4	14.295	12.605	0	0	7.07	9.515	5.925	9.28	0	0.7	38.2	13.445

51.77	20.885	12.26	0	0	64.615	3.02	20.225	12.965	0	0.505	136.15	306.48
6.28	14.295	1.04	0	5.985	121.9	0.54	0	0	0	65.145	330.915	250.825
34.75	20.885	8.25	0	0	93.595	12.885	11.5	78.01	24.525	14.425	270.53	197.185
7.55	14.825	1.93	0	0	51.225	0.54	2.19	0	0	21.195	109.5	119
9.935	14.295	2.145	0	0	6.035	1.14	2.095	0	0	13.065	84.81	20.75
13.245	10.77	2.145	0	0	7.915	1.335	0	0	0	0	104.375	33.035
12.385	12.02	1.47	0	0	1.06	0.54	3.145	0	0	0	47.905	12.78
26.79	0	10.845	0	0	3.335	3.02	6.475	0	0	0	75.81	10.475
54.185	7.915	14.925	0	0	9.795	6.59	6.2	0	0	2.575	68.475	10.065

**gut**	**gut**	**gut**	**gut**	**gut**	**gut**	**gut**	**gut**	**gut**	**gut**	**gut**	**gut**	**gut**
**gIL-1a**	**gIL-1b**	**gIL-2**	**gIL-4**	**gIL-5**	**gIL-6**	**gIL-10**	**gIL-12 (p40)**	**gIL-12 (p70)**	**gIL-13**	**gIL-17**	**gIP-10**	**gKC**
57.685	6.225	2.29	0	0	4.655	2.99	5.03	3.43	15.62	0.165	111.485	23.48
0.545	0	0	0	0	0.895	3.71	5.42	0	0	9.99	77.92	12.055
2.785	0	0	0	0	0.765	5.65	9.765	0	0	2.07	73.385	2.84
3.75	0.91	0	0	0	0.835	5.2	6.005	0	0	4.695	114.165	7.325

**gIL-1a**	**gIL-1b**	**gIL-2**	**gIL-4**	**gIL-5**	**gIL-6**	**gIL-10**	**gIL-12 (p40)**	**gIL-12 (p70)**	**gIL-13**	**gIL-17**	**gIP-10**	**gKC**
31.345	12.105	2.5	0	0	19.845	0.195	6.1	10.92	26.285	0.27	550.45	346.16
566.7	152.44	33.965	24.325	39.48	13.825	255.19	37.385	178.53	1013.515	99.925	452.96	1263.29
39.1	22.53	5.715	0	0.525	550.67	1.62	6.93	0	0	16.33	570.595	538.905
16.74	13.19	1.505	0	2.34	85.98	0.54	2.575	0	0	11.685	195.14	202.675
21.83	82.66	7.975	0	0	2.24	1.04	0	0	25.89	16.93	110.68	22.705
13.77	13.19	2.785	0	0	1.97	0.94	4.545	0	0	25.245	117.905	11.78
69.04	22.315	9.035	1.03	0.125	93.305	12.51	12.595	0	171.385	0	375.635	741.77

25.725	6.335	6.115	0.96	0	32.93	7.56	7.425	0	86.46	0	249.43	671.665
4.895	1.595	0	0	0	24.945	4.18	6.155	0	0	9.095	302.675	968.67
8.185	0	0.02	0	0	4.255	1.265	2.545	0	0	7.655	370.995	150.3
9.285	3.825	0	0	0	11.24	2.735	0.135	0	0	0	497.7	468.79
1564.28	741.235	67.94	80.235	190.14	23.585	506.24	100.36	445.89	2756.705	576.265	393.37	1669.055
27.925	17.32	6.935	0	0	43.02	2.84	18.67	10.25	0	0	117.34	175.805
95.4	14.295	12.605	0	0	7.07	9.515	5.925	9.28	0	0.7	38.2	13.445
51.77	20.885	12.26	0	0	64.615	3.02	20.225	12.965	0	0.505	136.15	306.48
6.28	14.295	1.04	0	5.985	121.9	0.54	0	0	0	65.145	330.915	250.825
34.75	20.885	8.25	0	0	93.595	12.885	11.5	78.01	24.525	14.425	270.53	197.185
7.55	14.825	1.93	0	0	51.225	0.54	2.19	0	0	21.195	109.5	119
9.935	14.295	2.145	0	0	6.035	1.14	2.095	0	0	13.065	84.81	20.75
13.245	10.77	2.145	0	0	7.915	1.335	0	0	0	0	104.375	33.035
12.385	12.02	1.47	0	0	1.06	0.54	3.145	0	0	0	47.905	12.78
26.79	0	10.845	0	0	3.335	3.02	6.475	0	0	0	75.81	10.475
54.185	7.915	14.925	0	0	9.795	6.59	6.2	0	0	2.575	68.475	10.065

gut MCP-1	gut MIG	gut MIP-1a	gut TNFa	gut VEGF	spleen GM-C	spleen IFN	spleen IL-1	spleen IL-1	spleen IL-2	spleen IL-4	spleen IL-5	spleen IL-6
gut	gut	gut	gut	gut	Spleen	Spleen	Spleen	Spleen	Spleen	Spleen	Spleen	Spleen
gut MCP-1	gut MIG	gut MIP-1a	gut TNFa	gut VEGF	spleen GM-C	spleen IFN	spleen IL-1	spleen IL-1	spleen IL-2	spleen IL-4	spleen IL-5	spleen IL-6
4.755	224.675	12.25	2.09	19.815	0	17.965	13.41	9.895	3.41	1.825	1.145	9.505
107.645	156.6	9.52	2.135	23.505	0	6.985	20.41	117.725	1.315	1.84	1.255	319.28
952.465	4234.44	129.195	2.51	16.69	27.755	5.53	21.645	101.53	1.645	1.925	1.48	926.35
1467.6	3362.73	73.04	2.325	84.26	57.03	175.67	261.72	124.585	4.235	1.45	0.61	813.47
831.065	13060.58	28.69	0	65.5	49.9	73.32	236.905	83.7968	14.825	0	3.665	523.07
686.995	15715.28	46.29	1.705	60.785	64.57	25.35	601.89	151.095	3.545	1.3	0.295	505.435
124.38	3321.795	53.56	0	7.98	13.565	15.43	84.92	27.195	0.54	0	0	28.385
3.795	348.395	0	2.065	25.47	0	7.645	6.075	6.89	1.605	1.825	1.145	7.815
1101.45	287.155	204.275	17.945	20.81	20.305	16.555	44.225	84.71	2.645	2.185	1.425	1617.785
972.11	4764.5	61.85	2.41	42.44	33.22	41.395	58.865	204.235	4.195	1.94	1.405	399.87
1375.09	8919.835	101.635	2.175	77.54	55.35	178.93	233.935	97.755	3.985	1.34	0	618.825
445.1	6015.505	27.29	0	32.88	35.265	31.68	115.645	33.3079	5.075	0	0	110.765
0	348.9	0	0.885	13.065	0	26.955	27.215	6.335	4.22	1.125	0	9.265
82.795	262.425	31.055	0	13.83	0	15.545	44.2	16.565	0.13	0	0	4.05
2.395	203.71	0	2.055	27.81	0	6.89	9.46	6.505	1.195	1.845	1.145	1.335
918.49	375.17	246.96	15.27	13.405	29.7	7.505	34.695	119.45	1.98	2.72	1.535	2948.085
1127.69	4106.27	104.49	2.55	27.095	20.305	35.32	55.68	119.45	2.155	2.005	1.275	1132.73
867.04	6506.875	41.36	0	60.755	46.65	60.8	221.495	150.5371	3.59	0	0	709.39
427.065	8356.235	21.325	0	23.71	51.145	45.055	216.91	114.6811	6.435	0	0	487.255
78.175	1376.06	21.325	0	18.385	35.265	9.67	176.395	56.7886	0.51	0	0	85.905
64.36	645.09	9.785	0	2.275	0	24.53	59.14	21.6	0.96	0	0	21.35
3.12	183.21	0	2.115	20.33	3.57	7.365	10.14	5.73	1.31	1.87	1.2	0.765
844.67	289.525	207.11	16.12	18.12	48.42	5.96	44.645	199.295	1.74	2.695	1.59	4394.45
1391.065	2452.045	137.675	3.95	20.645	95.405	134.295	351.48	360.795	4.565	1.97	0.69	1667.505
653.13	7186.695	30.06	0	85.92	43.17	32.1	310.94	190.7589	5.74	0	0	489.55
645.765	9080.615	27.29	0	39.34	40.185	28.745	234.025	133.1735	5.55	0	0	549.385

292.92	5058.795	32.72	0	34.675	0	2.7	133.065	42.9027	0	0	0	10.565
34.465	1004.205	50.235	0	4.705	2.835	24.53	139.11	29.255	1.345	0	0	22.115
47.2	212.845	25.205	1.385	49.34	0	25.92	17.515	22.315	3.765	1.38	0.03	4.415
338.675	319.19	101.635	17.76	25.945	37.985	16.9	103.445	120.66	3.965	1.56	0	416.745
1248.185	11522.34	157.14	3.95	71.435	72.255	129.985	309.305	229.225	4.445	1.665	0.125	1872.72
838.61	5556.99	30.06	0	54.4	6.4	18.12	60.69	35.2584	3.555	0.35	0	28.895
826.61	8250.47	20.09	0	42.82	15.295	111.265	134.695	82.22	0.76	0	0	543.215
412.18	8400.19	21.325	0	157.48	0	7.31	402.235	163.2859	0	0	0	529.36
72.83	925.865	13.77	0.485	22.055	0	8.485	22.65	7.44	0.495	0	0	2.4
614.445	258.24	172.075	17.19	13.425	67.27	23.17	181.715	248.88	3.72	2.6	0.125	980.01
1401.225	11396.48	155.72	3.22	43.205	78.945	135.975	249.075	203.375	4.02	1.745	0.125	1362.405
128.19	1437.67	18.045	0	32.215	39.405	19.54	235.705	97.9151	2.275	0	0	189.26
564.67	7777.545	33.3	0	28.835	19.78	84.905	206.75	107.63	0.4	0	0	589.165
**339.155**	**4104.585**	**16.3**	**0**	**44.505**	**40.185**	**13.785**	**267.08**	**129.0982**	**1.545**	**0**	**0**	**294.465**
**52.84**	**771.74**	**11.435**	**0**	**5.97**	**0**	**11.06**	**59.71**	**19.465**	**0.12**	**0**	**0**	**16.715**

gut	gut	gut	gut	gut	Spleen	Spleen	Spleen	Spleen	Spleen	Spleen	Spleen	Spleen
**gMCP-1**	**gMIG**	**gMIP-1a**	**gTNFa**	**gVEGF**	**sGM-CSF**	**sIFN-g**	**sIL-1a**	**sIL-1b**	**sIL-2**	**sIL-4**	**sIL-5**	**sIL-6**
4.755	224.675	12.25	2.09	19.815	0	17.965	13.41	9.895	3.41	1.825	1.145	9.505
3.795	348.395	0	2.065	25.47	0	7.645	6.075	6.89	1.605	1.825	1.145	7.815
2.395	203.71	0	2.055	27.81	0	6.89	9.46	6.505	1.195	1.845	1.145	1.335
3.12	183.21	0	2.115	20.33	3.57	7.365	10.14	5.73	1.31	1.87	1.2	0.765
47.2	212.845	25.205	1.385	49.34	0	25.92	17.515	22.315	3.765	1.38	0.03	4.415

gMCP-1	gMIG	gMIP-1a	gTNFa	gVEGF	sGM-CSF	sIFN-g	sIL-1a	sIL-1b	sIL-2	sIL-4	sIL-5	sIL-6
107.645	156.6	9.52	2.135	23.505	0	6.985	20.41	117.725	1.315	1.84	1.255	319.28
952.465	4234.44	129.195	2.51	16.69	27.755	5.53	21.645	101.53	1.645	1.925	1.48	926.35

1467.6	3362.73	73.04	2.325	84.26	57.03	175.67	261.72	124.585	4.235	1.45	0.61	813.47
831.065	13060.58	28.69	0	65.5	49.9	73.32	236.905	83.7968	14.825	0	3.665	523.07
686.995	15715.28	46.29	1.705	60.785	64.57	25.35	601.89	151.095	3.545	1.3	0.295	505.435
124.38	3321.795	53.56	0	7.98	13.565	15.43	84.92	27.195	0.54	0	0	28.385
1101.45	287.155	204.275	17.945	20.81	20.305	16.555	44.225	84.71	2.645	2.185	1.425	1617.785
972.11	4764.5	61.85	2.41	42.44	33.22	41.395	58.865	204.235	4.195	1.94	1.405	399.87
1375.09	8919.835	101.635	2.175	77.54	55.35	178.93	233.935	97.755	3.985	1.34	0	618.825
445.1	6015.505	27.29	0	32.88	35.265	31.68	115.645	33.3079	5.075	0	0	110.765
0	348.9	0	0.885	13.065	0	26.955	27.215	6.335	4.22	1.125	0	9.265
82.795	262.425	31.055	0	13.83	0	15.545	44.2	16.565	0.13	0	0	4.05
918.49	375.17	246.96	15.27	13.405	29.7	7.505	34.695	119.45	1.98	2.72	1.535	2948.085
1127.69	4106.27	104.49	2.55	27.095	20.305	35.32	55.68	119.45	2.155	2.005	1.275	1132.73
867.04	6506.875	41.36	0	60.755	46.65	60.8	221.495	150.5371	3.59	0	0	709.39
427.065	8356.235	21.325	0	23.71	51.145	45.055	216.91	114.6811	6.435	0	0	487.255
78.175	1376.06	21.325	0	18.385	35.265	9.67	176.395	56.7886	0.51	0	0	85.905
64.36	645.09	9.785	0	2.275	0	24.53	59.14	21.6	0.96	0	0	21.35
844.67	289.525	207.11	16.12	18.12	48.42	5.96	44.645	199.295	1.74	2.695	1.59	4394.45
1391.065	2452.045	137.675	3.95	20.645	95.405	134.295	351.48	360.795	4.565	1.97	0.69	1667.505
653.13	7186.695	30.06	0	85.92	43.17	32.1	310.94	190.7589	5.74	0	0	489.55
645.765	9080.615	27.29	0	39.34	40.185	28.745	234.025	133.1735	5.55	0	0	549.385
292.92	5058.795	32.72	0	34.675	0	2.7	133.065	42.9027	0	0	0	10.565
34.465	1004.205	50.235	0	4.705	2.835	24.53	139.11	29.255	1.345	0	0	22.115
338.675	319.19	101.635	17.76	25.945	37.985	16.9	103.445	120.66	3.965	1.56	0	416.745
1248.185	11522.34	157.14	3.95	71.435	72.255	129.985	309.305	229.225	4.445	1.665	0.125	1872.72
838.61	5556.99	30.06	0	54.4	6.4	18.12	60.69	35.2584	3.555	0.35	0	28.895
826.61	8250.47	20.09	0	42.82	15.295	111.265	134.695	82.22	0.76	0	0	543.215
412.18	8400.19	21.325	0	157.48	0	7.31	402.235	163.2859	0	0	0	529.36
72.83	925.865	13.77	0.485	22.055	0	8.485	22.65	7.44	0.495	0	0	2.4
614.445	258.24	172.075	17.19	13.425	67.27	23.17	181.715	248.88	3.72	2.6	0.125	980.01

1401.225	11396.48	155.72	3.22	43.205	78.945	135.975	249.075	203.375	4.02	1.745	0.125	1362.405
128.19	1437.67	18.045	0	32.215	39.405	19.54	235.705	97.9151	2.275	0	0	189.26
564.67	7777.545	33.3	0	28.835	19.78	84.905	206.75	107.63	0.4	0	0	589.165
**339.155**	**4104.585**	**16.3**	**0**	**44.505**	**40.185**	**13.785**	**267.08**	**129.0982**	**1.545**	**0**	**0**	**294.465**
**52.84**	**771.74**	**11.435**	**0**	**5.97**	**0**	**11.06**	**59.71**	**19.465**	**0.12**	**0**	**0**	**16.715**
**gut MCP-1**	**gut MIG**	**gut MIP-1a**	**gut TNFa**	**gut VEGF**	**spleen GM-C**	**spleen IFN-**	**spleen IL-1**	**spleen IL-1**	**spleen IL-2**	**spleen IL-4**	**spleen IL-5**	**spleen IL-6**
**gut**	**gut**	**gut**	**gut**	**gut**	**Spleen**	**Spleen**	**Spleen**	**Spleen**	**Spleen**	**Spleen**	**Spleen**	**Spleen**
**gut MCP-1**	**gut MIG**	**gut MIP-1a**	**gut TNFa**	**gut VEGF**	**spleen GM-C**	**spleen IFN-**	**spleen IL-1**	**spleen IL-1**	**spleen IL-2**	**spleen IL-4**	**spleen IL-5**	**spleen IL-6**
14.14	671.85	6.125	0	14.49	0	11	16.385	6.635	0.355	0	0	10.35
90.685	1452.935	40.78	4.065	29.585	0	12.4	23.46	28.57	0.265	0	0	95.895
947.82	1760.985	518.435	2.815	175.6	9.31	0	106.04	32.61	0.18	0	0	34.05
590.67	1652.235	40.025	1.89	34.025	12.375	13.41	52.8	33.77	4.875	0	0	40.12
301.565	971.125	24.475	1.095	35.05	0	1.04	67.27	87.34	4.855	0	0	47.485
39.615	1764.86	22.985	0.085	21.41	0	10.39	27.49	44.74	5.56	0	0	1.97
54.665	1153.875	19.78	2.16	39.475	12.375	18.82	30.245	45.805	6.625	3.825	0	2.89
17.285	938.65	0	0	36.225	9.31	0	6.45	4.805	0.21	0	0	3.255
17.61	940.48	0	0	56.015	0	0	6.22	0	0.34	0	0	0.62
28.83	1960.45	29.105	0.165	42.235	3.015	0	8.59	4.325	0.615	3.815	0	0.105
387.445	713.71	0	4.235	16.495	65.94	22.83	31.585	68.88	3.72	1.645	0	564.565
491.745	342.765	0	3.805	19.545	31.425	15.09	28.585	81.785	3.86	1.42	0	761.795
943.355	1174	64.105	1.575	18.515	43.74	0	46.515	85.795	0.225	0	0	369.265
221.09	4250.55	31.87	0	70.26	6.81	0	135.565	19.15	0.11	0	0	18.125
443.915	1781.115	44.29	0	124.435	0	0	11.56	3.825	0.07	0	0	0.245
1237.02	2258.13	1309.805	22.155	161.85	0	0	130.46	22.85	0.08	0	0	83.515
283.84	553.365	16.145	0.805	34.43	20.805	14.785	52.8	48.925	5.625	0	0	118.51
46.455	62.465	22.985	3.4	18.71	20.805	19.32	43.79	99.89	9.445	0.225	0	7.185

339.78	911.05	19.78	3.175	13.49	12.375	15.31	47.75	61.855	7.29	0.39	3.965	42.835
412.64	1583.56	37.94	0.03	14.79	12.375	2.97	79.135	140.685	10.37	0.295	4.415	54.395
504.955	1290.28	33.5	1.495	39.02	0	2.97	72.995	308.97	17.515	0.36	0	58.695
222.75	854.01	22.985	1.535	26.15	20.805	3.185	102.53	132.845	9.63	0.735	0	28.555
73.865	482.09	31.115	0.68	12.54	0	3.185	27.23	23.325	5.67	0	0	8.085
66.535	1751.62	11.785	1.775	10.06	0	3.4	18.315	14.295	3.99	0.025	0	5.1
52.445	356.055	11.785	0.89	39.545	0	0	29.565	58.18	7.245	0	0	3.89
33.185	1042.02	0	0	8.03	12.375	10.555	29.395	15.345	6.065	0.595	0.525	5.92
27.41	659.735	0	0	33.35	20.805	3.08	25.11	316.845	14.845	1.28	0	1.835

pg/mg												
**gut**	**gut**	**gut**	**gut**	**gut**	**Spleen**	**Spleen**	**Spleen**	**Spleen**	**Spleen**	**Spleen**	**Spleen**	**Spleen**
**gMCP-1**	**gMIG**	**gMIP-1a**	**gTNFa**	**gVEGF**	**sGM-CSF**	**sIFN-g**	**sIL-1a**	**sIL-1b**	**sIL-2**	**sIL-4**	**sIL-5**	**sIL-6**
14.14	671.85	6.125	0	14.49	0	11	16.385	6.635	0.355	0	0	10.35
17.285	938.65	0	0	36.225	9.31	0	6.45	4.805	0.21	0	0	3.255
17.61	940.48	0	0	56.015	0	0	6.22	0	0.34	0	0	0.62
28.83	1960.45	29.105	0.165	42.235	3.015	0	8.59	4.325	0.615	3.815	0	0.105

**gMCP-1**	**gMIG**	**gMIP-1a**	**gTNFa**	**gVEGF**	**sGM-CSF**	**sIFN-g**	**sIL-1a**	**sIL-1b**	**sIL-2**	**sIL-4**	**sIL-5**	**sIL-6**
90.685	1452.935	40.78	4.065	29.585	0	12.4	23.46	28.57	0.265	0	0	95.895
947.82	1760.985	518.435	2.815	175.6	9.31	0	106.04	32.61	0.18	0	0	34.05
590.67	1652.235	40.025	1.89	34.025	12.375	13.41	52.8	33.77	4.875	0	0	40.12
301.565	971.125	24.475	1.095	35.05	0	1.04	67.27	87.34	4.855	0	0	47.485
39.615	1764.86	22.985	0.085	21.41	0	10.39	27.49	44.74	5.56	0	0	1.97
54.665	1153.875	19.78	2.16	39.475	12.375	18.82	30.245	45.805	6.625	3.825	0	2.89
387.445	713.71	0	4.235	16.495	65.94	22.83	31.585	68.88	3.72	1.645	0	564.565

491.745	342.765	0	3.805	19.545	31.425	15.09	28.585	81.785	3.86	1.42	0	761.795
943.355	1174	64.105	1.575	18.515	43.74	0	46.515	85.795	0.225	0	0	369.265
221.09	4250.55	31.87	0	70.26	6.81	0	135.565	19.15	0.11	0	0	18.125
443.915	1781.115	44.29	0	124.435	0	0	11.56	3.825	0.07	0	0	0.245
1237.02	2258.13	1309.805	22.155	161.85	0	0	130.46	22.85	0.08	0	0	83.515
283.84	553.365	16.145	0.805	34.43	20.805	14.785	52.8	48.925	5.625	0	0	118.51
46.455	62.465	22.985	3.4	18.71	20.805	19.32	43.79	99.89	9.445	0.225	0	7.185
339.78	911.05	19.78	3.175	13.49	12.375	15.31	47.75	61.855	7.29	0.39	3.965	42.835
412.64	1583.56	37.94	0.03	14.79	12.375	2.97	79.135	140.685	10.37	0.295	4.415	54.395
504.955	1290.28	33.5	1.495	39.02	0	2.97	72.995	308.97	17.515	0.36	0	58.695
222.75	854.01	22.985	1.535	26.15	20.805	3.185	102.53	132.845	9.63	0.735	0	28.555
73.865	482.09	31.115	0.68	12.54	0	3.185	27.23	23.325	5.67	0	0	8.085
66.535	1751.62	11.785	1.775	10.06	0	3.4	18.315	14.295	3.99	0.025	0	5.1
52.445	356.055	11.785	0.89	39.545	0	0	29.565	58.18	7.245	0	0	3.89
33.185	1042.02	0	0	8.03	12.375	10.555	29.395	15.345	6.065	0.595	0.525	5.92
27.41	659.735	0	0	33.35	20.805	3.08	25.11	316.845	14.845	1.28	0	1.835

spleen IL-1	spleen IL-1	spleen IL-1	spleen IL-1	spleen IL-1	spleen IP-10	spleen KC	spleen MCI	spleen MIG	spleen MIF	spleen TNF	spleen VEG
Spleen	Spleen	Spleen	Spleen	Spleen	Spleen	Spleen	Spleen	Spleen	Spleen	Spleen	Spleen
spleen IL-1	spleen IL-1	spleen IL-1	spleen IL-1	spleen IL-1	spleen IP-10	spleen KC	spleen MCI	spleen MIG	spleen MIF	spleen TNF	spleen VEG
1.425	1.565	1.51	2.85	1.32	377.51	26.785	12.66	2280.2	10.23	2.135	4.74
2.2	3.055	0.945	4.165	1.31	1420.575	833.285	300.55	3018.78	199.005	19.965	12.77
1.39	8.62	1.085	32.465	1.55	4875.13	2395.82	1280.005	3929.455	917.37	4.115	3.01
14.175	23.34	25.01	56.885	0	5000.0	1459.075	795.24	24877.6	496.205	15.275	3.315
156.68	36.61	9.73	18.495	29.355	4575.275	2762.195	571.545	22265.9	218.855	7.075	3.88
18.17	16.68	2.445	0	1.725	5000.0	2686.715	530.415	24877.6	318.29	3.805	7.44
0.73	1.37	1.85	0	0.65	1407.425	725.53	72.52	8197.025	73.925	0.355	2.52
1.445	1.4	0	3.31	1.285	395.56	65.125	14.73	1791.01	10.92	2.115	6.175
3.09	12.33	2.01	26.93	1.605	3040.265	3436.085	1067.68	3617.245	814.13	91.81	6.51
1.95	6.65	1.795	21.815	1.66	4528.16	2494.96	1092.405	5407.135	978.79	4.66	2.14
8.79	21.545	29.015	68.53	0.815	5000.0	1290.96	762.045	24877.6	414.145	12.045	9.225
112.85	12.24	8	0	5.28	2110.18	1140.825	124.62	22265.9	67.385	0	3.33
6.75	2.8	0	0	0	540.155	93.645	0	4051.805	105.725	1.055	4.94
5.355	1.03	0	0	0.08	756.95	169.165	41.775	4632.385	50.715	0.83	1.555
1.245	1.375	0.005	9.2	1.305	337.25	12.415	5.06	2933.845	23.545	2.15	3.815
2.2	12.11	5.32	14.88	1.855	3018.45	4383.43	1536.61	3878.39	1014.41	157.085	5.8
1.39	10.485	1.935	35.835	1.82	3617.02	1355.73	880.765	4711.645	860.22	6.535	3.255
74.85	32.92	6.695	0	11.3	4845.895	1904.51	655.62	10838.23	488.175	10.145	4.275
154.02	18.915	8.435	0	16.39	3563.52	1824.65	335.195	22265.9	181.535	5.04	7.03
90.015	8.165	7.24	0	0	1263.59	610.755	75.67	10970.97	72.94	0	11.6
5.355	2.02	0	0.245	0.11	1200.555	450.72	56.535	6448.13	55.43	0.485	6.98
1.46	1.165	0.805	3.54	1.365	313.035	5.8	3.12	2571.14	8.035	2.16	2.84
2.105	16.455	3.54	15.175	1.5	4781.015	4692.03	2207.06	4043.03	1331.82	209.83	11.575
17.96	55.375	25.47	61.27	0	5000.0	1549.7	1119.86	24877.6	985.465	35.495	4.095
74.85	17.165	8.65	0	3.36	4418.565	1942.02	644.58	22265.9	252.72	11.91	5.22
143.33	27.755	10.16	0	10.915	3019.99	2443.11	566.775	22265.9	309.85	10.145	5.195

21.615	0	2.7	0	0	387.91	261.545	54.895	5254.115	44.82	0	4.185
2.83	2.275	2.055	3.255	0.345	3153.495	775.585	63.56	7477.715	73.925	0.485	3.1
5.94	6.03	34.785	91.345	0	453.35	62.62	0	1678.77	75.785	1.705	8.035
8.585	16.025	23.135	38.93	0	1363.64	1988.85	740.64	3022.12	519.9	286.65	6.11
13.34	67.205	22.175	0	0	5000.0	1816.235	1059.965	24877.6	928.035	35.37	2.35
41.675	1.99	8.435	0	0	1316.305	190.055	92.565	6319.825	41.36	0	5.105
4.09	17.13	2.26	0	14.265	31520.14	2255.255	532.59	10849.38	529.285	3.345	0.565
57.205	0	3.825	0	6.75	1146.46	2108.42	371.63	9361.89	185.445	0	3.555
0	0.3	0	0	0.01	374.965	54.135	33.69	1875.975	26.98	0	3.335
11.26	63.32	21.19	0	0	5000.0	2492.18	1083.285	2431.75	730.64	236.75	6.83
15.435	43.435	4.7	0	0	5000.0	1443.495	938.18	24877.6	758.635	17.315	2.275
56.84	10.105	6.26	0	0	4849.91	1478.73	433.085	8414.34	245.645	3.8	4.29
5.04	12.24	4.18	0	12.48	5143.78	2244.385	439.52	10996.08	345.99	2.505	1.89
**63.375**	**15.99**	**8.435**	**0**	**0.145**	**1528.165**	**1651.82**	**343.905**	**22265.9**	**162.655**	**2.49**	**10.38**
**0.115**	**0**	**2.26**	**0**	**0.025**	**1431.365**	**562.985**	**46.615**	**5708.48**	**70.54**	**0**	**2.1**

**Spleen**	**Spleen**	**Spleen**	**Spleen**	**Spleen**	**Spleen**	**Spleen**	**Spleen**	**Spleen**	**Spleen**	**Spleen**	**Spleen**
**sIL-10**	**sIL-12 (p40)**	**sIL-12 (p70)**	**sIL-13**	**sIL-17**	**sIP-10**	**sKC**	**sMCP-1**	**sMIG**	**sMIP-1a**	**sTNFa**	**sVEGF**
1.425	1.565	1.51	2.85	1.32	377.51	26.785	12.66	2280.2	10.23	2.135	4.74
1.445	1.4	0	3.31	1.285	395.56	65.125	14.73	1791.01	10.92	2.115	6.175
1.245	1.375	0.005	9.2	1.305	337.25	12.415	5.06	2933.845	23.545	2.15	3.815
1.46	1.165	0.805	3.54	1.365	313.035	5.8	3.12	2571.14	8.035	2.16	2.84
5.94	6.03	34.785	91.345	0	453.35	62.62	0	1678.77	75.785	1.705	8.035

**sIL-10**	**sIL-12 (p40)**	**sIL-12 (p70)**	**sIL-13**	**sIL-17**	**sIP-10**	**sKC**	**sMCP-1**	**sMIG**	**sMIP-1a**	**sTNFa**	**sVEGF**
2.2	3.055	0.945	4.165	1.31	1420.575	833.285	300.55	3018.78	199.005	19.965	12.77
1.39	8.62	1.085	32.465	1.55	4875.13	2395.82	1280.005	3929.455	917.37	4.115	3.01

14.175	23.34	25.01	56.885	0	5000.0	1459.075	795.24	24877.6	496.205	15.275	3.315
156.68	36.61	9.73	18.495	29.355	4575.275	2762.195	571.545	22265.9	218.855	7.075	3.88
18.17	16.68	2.445	0	1.725	5000.0	2686.715	530.415	24877.6	318.29	3.805	7.44
0.73	1.37	1.85	0	0.65	1407.425	725.53	72.52	8197.025	73.925	0.355	2.52
3.09	12.33	2.01	26.93	1.605	3040.265	3436.085	1067.68	3617.245	814.13	91.81	6.51
1.95	6.65	1.795	21.815	1.66	4528.16	2494.96	1092.405	5407.135	978.79	4.66	2.14
8.79	21.545	29.015	68.53	0.815	5000.0	1290.96	762.045	24877.6	414.145	12.045	9.225
112.85	12.24	8	0	5.28	2110.18	1140.825	124.62	22265.9	67.385	0	3.33
6.75	2.8	0	0	0	540.155	93.645	0	4051.805	105.725	1.055	4.94
5.355	1.03	0	0	0.08	756.95	169.165	41.775	4632.385	50.715	0.83	1.555
2.2	12.11	5.32	14.88	1.855	3018.45	4383.43	1536.61	3878.39	1014.41	157.085	5.8
1.39	10.485	1.935	35.835	1.82	3617.02	1355.73	880.765	4711.645	860.22	6.535	3.255
74.85	32.92	6.695	0	11.3	4845.895	1904.51	655.62	10838.23	488.175	10.145	4.275
154.02	18.915	8.435	0	16.39	3563.52	1824.65	335.195	22265.9	181.535	5.04	7.03
90.015	8.165	7.24	0	0	1263.59	610.755	75.67	10970.97	72.94	0	11.6
5.355	2.02	0	0.245	0.11	1200.555	450.72	56.535	6448.13	55.43	0.485	6.98
2.105	16.455	3.54	15.175	1.5	4781.015	4692.03	2207.06	4043.03	1331.82	209.83	11.575
17.96	55.375	25.47	61.27	0	5000.0	1549.7	1119.86	24877.6	985.465	35.495	4.095
74.85	17.165	8.65	0	3.36	4418.565	1942.02	644.58	22265.9	252.72	11.91	5.22
143.33	27.755	10.16	0	10.915	3019.99	2443.11	566.775	22265.9	309.85	10.145	5.195
21.615	0	2.7	0	0	387.91	261.545	54.895	5254.115	44.82	0	4.185
2.83	2.275	2.055	3.255	0.345	3153.495	775.585	63.56	7477.715	73.925	0.485	3.1
8.585	16.025	23.135	38.93	0	1363.64	1988.85	740.64	3022.12	519.9	286.65	6.11
13.34	67.205	22.175	0	0	5000.0	1816.235	1059.965	24877.6	928.035	35.37	2.35
41.675	1.99	8.435	0	0	1316.305	190.055	92.565	6319.825	41.36	0	5.105
4.09	17.13	2.26	0	14.265	31520.14	2255.255	532.59	10849.38	529.285	3.345	0.565
57.205	0	3.825	0	6.75	1146.46	2108.42	371.63	9361.89	185.445	0	3.555
0	0.3	0	0	0.01	374.965	54.135	33.69	1875.975	26.98	0	3.335
11.26	63.32	21.19	0	0	5000.0	2492.18	1083.285	2431.75	730.64	236.75	6.83

15.435	43.435	4.7	0	0	5000.0	1443.495	938.18	24877.6	758.635	17.315	2.275
56.84	10.105	6.26	0	0	4849.91	1478.73	433.085	8414.34	245.645	3.8	4.29
5.04	12.24	4.18	0	12.48	5143.78	2244.385	439.52	10996.08	345.99	2.505	1.89
**63.375**	**15.99**	**8.435**	**0**	**0.145**	**1528.165**	**1651.82**	**343.905**	**22265.9**	**162.655**	**2.49**	**10.38**
**0.115**	**0**	**2.26**	**0**	**0.025**	**1431.365**	**562.985**	**46.615**	**5708.48**	**70.54**	**0**	**2.1**
**spleen IL-1**	**spleen IL-1**	**spleen IL-1**	**spleen IL-1**	**spleen IL-1**	**spleen IP-10**	**spleen KC**	**spleen MCI**	**spleen MIG**	**spleen MIF**	**spleen TNF**	**spleen VEG**
**Spleen**	**Spleen**	**Spleen**	**Spleen**	**Spleen**	**Spleen**	**Spleen**	**Spleen**	**Spleen**	**Spleen**	**Spleen**	**Spleen**
**spleen IL-1**	**spleen IL-1**	**spleen IL-1**	**spleen IL-1**	**spleen IL-1**	**spleen IP-10**	**spleen KC**	**spleen MCI**	**spleen MIG**	**spleen MIF**	**spleen TNF**	**spleen VEG**
0.5	3.71	0	0	0.11	961.055	87.455	17.565	6764.43	27.575	0	0.17
0.88	10.31	0	0	0.245	1905.695	1455.01	354.42	5419.895	43.84	65.17	0.345
2.38	15.655	0	0	0	3658.835	2581.685	415.505	2440.9	130.45	0.08	12.3
2.19	1.315	0	0	0	841.89	614.3	120.61	1958.38	50.34	1.38	5.665
0.115	2.385	0	12.83	0.365	428.765	498.385	182.885	3705.255	87.185	0.55	67.055
0	2.955	0	0.495	0	238.36	39.99	28.455	780.755	25.9	2.12	6.935
3.02	1.415	0	4.54	0	546.135	54.64	46.455	3008.8	32.325	1.495	6.375
3.71	1.515	0	0	0	728.67	178.73	27.24	7531.65	16.335	0.01	13.745
1.265	0	0	0	0	689.71	51.77	12.425	3470.215	4.945	0	13.85
3.61	3.355	0	0	0	614.495	57.435	23.365	7531.65	22.655	0.345	4.44
11.26	25.155	0	0	0	921.145	1884.65	1018.605	3413.17	136.08	82.295	3.18
3.15	32.53	0	0	0	974.85	1846.63	1162.335	4031.015	172.075	54.43	2.66
2.93	25.925	0	0	0	716.365	4272.47	2394.12	1892.26	184.175	19.12	6.915
3.37	15.335	0	0	0	2272.405	756.465	266.215	2810.245	97.43	0.24	7.725
1.595	0.545	0	0	0	461.945	94.79	54.485	1936.675	8.03	0.005	8.405
1.38	7.57	0	0	2.715	1398.13	2151.245	349.95	2816.5	99.69	0.185	7.795
3.84	9.9	0	0	0	2791.39	752.255	184.6	3194.575	57.76	3.62	6.36
10.87	5.47	0	38.365	0	265.075	21.32	29.46	1518.67	33.5	1.135	12.39

4.65	8.825	3.41	6.79	0	1130.45	723.065	140.835	4306.205	54.55	1.93	7.705
0.74	7.925	0	40.58	1.025	714.015	600.27	189.855	5000	102.595	3.03	62.95
0.94	6.84	0	121.385	5.365	880.53	693.76	241.735	4444.09	131.055	1.46	118.785
0.74	7.02	0	38.05	1.025	564.5	427.495	157.425	3664.58	94.01	1.93	69.97
0.33	1.805	0	0	0	403.69	56.885	68.765	755.16	35.77	1.34	8.595
0.33	0.305	0	0	0	460.24	111.4	43.845	1286.365	31.115	2.005	8.18
0.33	0	0	1.465	0	594.015	68.71	42.48	1254.28	38.99	2.085	11.34
1.335	5.745	1.705	0	0	443.55	61.89	48.935	2422.495	43.975	4.56	3.7
4.29	3.895	0	115.67	0.7	437.145	49.265	38.87	2102.115	28.595	2.58	53.475

Spleen	Spleen	Spleen	Spleen	Spleen	Spleen	Spleen	Spleen	Spleen	Spleen	Spleen	Spleen
sIL-10	sIL-12 (p40)	sIL-12 (p70)	sIL-13	sIL-17	sIP-10	sKC	sMCP-1	sMIG	sMIP-1a	sTNFa	sVEGF
0.5	3.71	0	0	0.11	961.055	87.455	17.565	6764.43	27.575	0	0.17
3.71	1.515	0	0	0	728.67	178.73	27.24	7531.65	16.335	0.01	13.745
1.265	0	0	0	0	689.71	51.77	12.425	3470.215	4.945	0	13.85
3.61	3.355	0	0	0	614.495	57.435	23.365	7531.65	22.655	0.345	4.44

sIL-10	sIL-12 (p40)	sIL-12 (p70)	sIL-13	sIL-17	sIP-10	sKC	sMCP-1	sMIG	sMIP-1a	sTNFa	sVEGF
0.88	10.31	0	0	0.245	1905.695	1455.01	354.42	5419.895	43.84	65.17	0.345
2.38	15.655	0	0	0	3658.835	2581.685	415.505	2440.9	130.45	0.08	12.3
2.19	1.315	0	0	0	841.89	614.3	120.61	1958.38	50.34	1.38	5.665
0.115	2.385	0	12.83	0.365	428.765	498.385	182.885	3705.255	87.185	0.55	67.055
0	2.955	0	0.495	0	238.36	39.99	28.455	780.755	25.9	2.12	6.935
3.02	1.415	0	4.54	0	546.135	54.64	46.455	3008.8	32.325	1.495	6.375
11.26	25.155	0	0	0	921.145	1884.65	1018.605	3413.17	136.08	82.295	3.18

3.15	32.53	0	0	0	974.85	1846.63	1162.335	4031.015	172.075	54.43	2.66
2.93	25.925	0	0	0	716.365	4272.47	2394.12	1892.26	184.175	19.12	6.915
3.37	15.335	0	0	0	2272.405	756.465	266.215	2810.245	97.43	0.24	7.725
1.595	0.545	0	0	0	461.945	94.79	54.485	1936.675	8.03	0.005	8.405
1.38	7.57	0	0	2.715	1398.13	2151.245	349.95	2816.5	99.69	0.185	7.795
3.84	9.9	0	0	0	2791.39	752.255	184.6	3194.575	57.76	3.62	6.36
10.87	5.47	0	38.365	0	265.075	21.32	29.46	1518.67	33.5	1.135	12.39
4.65	8.825	3.41	6.79	0	1130.45	723.065	140.835	4306.205	54.55	1.93	7.705
0.74	7.925	0	40.58	1.025	714.015	600.27	189.855	5000	102.595	3.03	62.95
0.94	6.84	0	121.385	5.365	880.53	693.76	241.735	4444.09	131.055	1.46	118.785
0.74	7.02	0	38.05	1.025	564.5	427.495	157.425	3664.58	94.01	1.93	69.97
0.33	1.805	0	0	0	403.69	56.885	68.765	755.16	35.77	1.34	8.595
0.33	0.305	0	0	0	460.24	111.4	43.845	1286.365	31.115	2.005	8.18
0.33	0	0	1.465	0	594.015	68.71	42.48	1254.28	38.99	2.085	11.34
1.335	5.745	1.705	0	0	443.55	61.89	48.935	2422.495	43.975	4.56	3.7
4.29	3.895	0	115.67	0.7	437.145	49.265	38.87	2102.115	28.595	2.58	53.475

kidney GM-C	kidney IFN	kidney IL-1	kidney IL-1	kidney IL-2	kidney IL-4	kidney IL-5	kidney IL-6	kidney IL-1	kidney IL-1	kidney IL-1	kidney IL-1	kidney IL-1
**Kidney**	**Kidney**	**Kidney**	**Kidney**	**Kidney**	**Kidney**	**Kidney**	**Kidney**	**Kidney**	**Kidney**	**Kidney**	**Kidney**	**Kidney**
kidney GM-C	kidney IFN	kidney IL-1	kidney IL-1	kidney IL-2	kidney IL-4	kidney IL-5	kidney IL-6	kidney IL-1	kidney IL-1	kidney IL-1	kidney IL-1	kidney IL-1
5.595	5785.58	419.14	62.65	79.27	1.65	1.22	7.295	4.925	9.68	2.225	68.185	1.58
2.255	108.315	230.855	40.15	12.94	1.675	1.255	135.905	15.155	10.615	0.26	36.055	1.49
9.42	169.89	1000.025	143.485	15.935	1.705	1.385	1332.355	10.315	18.835	3.465	35.79	1.855
64.57	184.245	748.735	323.74	50.535	1.46	3.665	1182.775	97.92	157.21	41.62	760.695	3.37
35.265	136.02	223.725	55.4191	42.475	0	5.93	635.565	101.835	57.125	7.675	74.46	5.12
0	28.45	250.41	52.555	9.615	1.03	0.125	296.245	16.275	16.245	0	0	0
0	17.58	37.905	9.805	0.93	0	0	1.08	1.115	1.92	0	0	0.19
4.645	195.42	609.585	80.43	108.9	1.62	1.235	6.555	7.25	11.78	0.53	1020.32	1.645
7.265	82.57	219.935	20.105	11.825	1.68	1.32	474.275	10.72	6.43	1.085	21.125	1.425
9.42	115.655	227.545	109.79	22.095	1.66	1.33	685.485	17.13	19.99	30.06	198.485	1.82
55.35	163.01	482.785	149.54	34.465	1.28	1.65	945.25	51.335	88.775	10.325	494.45	2.38
51.145	133.465	260.375	47.5922	58.91	0	8.09	511.485	126.14	69.91	7.675	100.16	9.005
58.65	98.485	535.12	124.585	22.195	1.205	1.065	5.875	39.48	55.62	4.7	197.315	0
0	103.13	270.275	81.61	15.385	0	0	1.99	17.75	26.13	3.05	21.625	1.295
7.265	147.99	268.7	71.515	17.38	1.725	1.295	49.62	17.3	12.44	4.95	128.36	1.905
9.42	102.49	251.855	86.625	15.44	1.74	1.435	509.68	19.315	10.65	2.08	56.12	1.66
7.265	124.56	724.66	56.275	24.38	1.645	1.33	1343.15	4.815	17.735	1.51	64.59	1.535
22.615	28.745	148.62	54.0496	20.455	0	0	543.87	34.095	24.46	6.48	0	1
39.405	99.025	236.04	33.7976	49.835	0	6.25	806.9	127.16	72.485	9.085	93.445	5.44
33.47	13.785	63.22	16.5502	1.94	0	0	22.51	22.41	17.05	7.24	0	0
0	45.725	222.775	18.745	6.56	0	0	3.24	3.935	5.14	0	0	0.245
6.46	23.6	249.08	47.81	6.615	1.68	1.275	9.895	4.725	10.795	6.98	66.315	1.935
5.595	28.08	305.365	11.54	11.065	1.65	1.22	378.435	4.065	3.325	0.395	6.255	1.375
74.575	226.78	624.49	183.485	49.865	1.36	2.775	1178.375	66.2	119.28	19.675	684.61	3.72
0	9.67	88.135	186.086	10.765	0	0	722.975	20.01	9.335	4.27	0	0
48.625	204.545	261.015	72.8906	46.115	0	7.825	429.04	114.22	83.71	10.375	97.93	7.38

0	14.125	24.23	16.0107	0	0	0	6.99	5.71	1.31	4.495	0	0
11.65	33.13	241.895	36.015	10.505	0	0	13.26	9.01	12.18	0.975	2.92	0.675
57.03	225.44	638.19	198.795	43.685	1.38	2.36	21.68	57.75	98.535	15.905	494.175	3.285
55.35	233.83	617.255	226.19	47.045	1.42	2.915	188.945	68.655	112.895	21.685	551.39	3.95
79.985	225.075	610.175	248.88	54.565	1.54	2.775	1998.81	83.01	156.385	141.395	694.945	4.35
16.52	42.205	190.51	157.7249	20.97	0	0	25.825	30.25	14.555	6.04	0	0
21.115	177.305	255.415	166.685	28.39	0	0	807.645	31.095	72.04	11.865	147.935	2.535
6.4	8.025	14.145	5.8017	0	0	0	302.76	3.875	2.155	6.26	0	0
0	18.565	230.375	41.32	7.085	0	0	0	4.72	7.075	0	0.245	0.475
73.43	242.345	630.92	174.265	51.335	1.36	2.5	339.79	64.64	90.72	65.705	645.78	3.2
77.885	186.3	565.41	177.34	41.295	1.42	1.65	1724.745	57.525	103.385	33.59	664.045	1.635
31.585	19.325	120.56	18.675	13.985	0	0	1255.45	27.92	22.785	6.695	0	0
20.455	178.21	252.33	180.35	30.965	0	0	974.455	36.78	51.89	8.335	133.175	3.19
**0**	**25.25**	**36.635**	**10.58**	**2.165**	**0**	**0**	**164.15**	**7.49**	**4.155**	**5.16**	**0**	**0**
**9.46**	**123.825**	**192.645**	**97.555**	**17.985**	**0**	**0**	**6.365**	**20.895**	**30.55**	**7.335**	**126.07**	**1.62**

Kidney	Kidney	Kidney	Kidney	Kidney	Kidney	Kidney	Kidney	Kidney	Kidney	Kidney	Kidney	Kidney
**kGM-CSF**	**kIFN-g**	**kIL-1a**	**kIL-1b**	**kIL-2**	**kIL-4**	**kIL-5**	**kIL-6**	**kIL-10**	**kIL-12 (p40**	**kIL-12 (p70)**	**kIL-13**	**kIL-17**
5.595	5785.58	419.14	62.65	79.27	1.65	1.22	7.295	4.925	9.68	2.225	68.185	1.58
4.645	195.42	609.585	80.43	108.9	1.62	1.235	6.555	7.25	11.78	0.53	1020.32	1.645
7.265	147.99	268.7	71.515	17.38	1.725	1.295	49.62	17.3	12.44	4.95	128.36	1.905
6.46	23.6	249.08	47.81	6.615	1.68	1.275	9.895	4.725	10.795	6.98	66.315	1.935
57.03	225.44	638.19	198.795	43.685	1.38	2.36	21.68	57.75	98.535	15.905	494.175	3.285

**kGM-CSF**	**kIFN-g**	**kIL-1a**	**kIL-1b**	**kIL-2**	**kIL-4**	**kIL-5**	**kIL-6**	**kIL-10**	**kIL-12 (p40**	**kIL-12 (p70)**	**kIL-13**	**kIL-17**
2.255	108.315	230.855	40.15	12.94	1.675	1.255	135.905	15.155	10.615	0.26	36.055	1.49
9.42	169.89	1000.025	143.485	15.935	1.705	1.385	1332.355	10.315	18.835	3.465	35.79	1.855

64.57	184.245	748.735	323.74	50.535	1.46	3.665	1182.775	97.92	157.21	41.62	760.695	3.37
35.265	136.02	223.725	55.4191	42.475	0	5.93	635.565	101.835	57.125	7.675	74.46	5.12
0	28.45	250.41	52.555	9.615	1.03	0.125	296.245	16.275	16.245	0	0	0
0	17.58	37.905	9.805	0.93	0	0	1.08	1.115	1.92	0	0	0.19
7.265	82.57	219.935	20.105	11.825	1.68	1.32	474.275	10.72	6.43	1.085	21.125	1.425
9.42	115.655	227.545	109.79	22.095	1.66	1.33	685.485	17.13	19.99	30.06	198.485	1.82
55.35	163.01	482.785	149.54	34.465	1.28	1.65	945.25	51.335	88.775	10.325	494.45	2.38
51.145	133.465	260.375	47.5922	58.91	0	8.09	511.485	126.14	69.91	7.675	100.16	9.005
58.65	98.485	535.12	124.585	22.195	1.205	1.065	5.875	39.48	55.62	4.7	197.315	0
0	103.13	270.275	81.61	15.385	0	0	1.99	17.75	26.13	3.05	21.625	1.295
9.42	102.49	251.855	86.625	15.44	1.74	1.435	509.68	19.315	10.65	2.08	56.12	1.66
7.265	124.56	724.66	56.275	24.38	1.645	1.33	1343.15	4.815	17.735	1.51	64.59	1.535
22.615	28.745	148.62	54.0496	20.455	0	0	543.87	34.095	24.46	6.48	0	1
39.405	99.025	236.04	33.7976	49.835	0	6.25	806.9	127.16	72.485	9.085	93.445	5.44
33.47	13.785	63.22	16.5502	1.94	0	0	22.51	22.41	17.05	7.24	0	0
0	45.725	222.775	18.745	6.56	0	0	3.24	3.935	5.14	0	0	0.245
5.595	28.08	305.365	11.54	11.065	1.65	1.22	378.435	4.065	3.325	0.395	6.255	1.375
74.575	226.78	624.49	183.485	49.865	1.36	2.775	1178.375	66.2	119.28	19.675	684.61	3.72
0	9.67	88.135	186.086	10.765	0	0	722.975	20.01	9.335	4.27	0	0
48.625	204.545	261.015	72.8906	46.115	0	7.825	429.04	114.22	83.71	10.375	97.93	7.38
0	14.125	24.23	16.0107	0	0	0	6.99	5.71	1.31	4.495	0	0
11.65	33.13	241.895	36.015	10.505	0	0	13.26	9.01	12.18	0.975	2.92	0.675
55.35	233.83	617.255	226.19	47.045	1.42	2.915	188.945	68.655	112.895	21.685	551.39	3.95
79.985	225.075	610.175	248.88	54.565	1.54	2.775	1998.81	83.01	156.385	141.395	694.945	4.35
16.52	42.205	190.51	157.7249	20.97	0	0	25.825	30.25	14.555	6.04	0	0
21.115	177.305	255.415	166.685	28.39	0	0	807.645	31.095	72.04	11.865	147.935	2.535
6.4	8.025	14.145	5.8017	0	0	0	302.76	3.875	2.155	6.26	0	0
0	18.565	230.375	41.32	7.085	0	0	0	4.72	7.075	0	0.245	0.475
73.43	242.345	630.92	174.265	51.335	1.36	2.5	339.79	64.64	90.72	65.705	645.78	3.2

77.885	186.3	565.41	177.34	41.295	1.42	1.65	1724.745	57.525	103.385	33.59	664.045	1.635
31.585	19.325	120.56	18.675	13.985	0	0	1255.45	27.92	22.785	6.695	0	0
20.455	178.21	252.33	180.35	30.965	0	0	974.455	36.78	51.89	8.335	133.175	3.19
**0**	**25.25**	**36.635**	**10.58**	**2.165**	**0**	**0**	**164.15**	**7.49**	**4.155**	**5.16**	**0**	**0**
**9.46**	**123.825**	**192.645**	**97.555**	**17.985**	**0**	**0**	**6.365**	**20.895**	**30.55**	**7.335**	**126.07**	**1.62**

kidney GM-C!	kidney IFN·	kidney IL-1	kidney IL-1	kidney IL-2	kidney IL-4	kidney IL-5	kidney IL-6	kidney IL-1	kidney IL-1	kidney IL-1	kidney IL-1	kidney IL-1
**Kidney**	**Kidney**	**Kidney**	**Kidney**	**Kidney**	**Kidney**	**Kidney**	**Kidney**	**Kidney**	**Kidney**	**Kidney**	**Kidney**	**Kidney**
**kidney GM-C!**	**kidney IFN·**	**kidney IL-1**	**kidney IL-1**	**kidney IL-2**	**kidney IL-4**	**kidney IL-5**	**kidney IL-6**	**kidney IL-1**	**kidney IL-1**	**kidney IL-1**	**kidney IL-1**	**kidney IL-1**
23.605	222.935	508.36	153.175	36.065	0	0	8.075	38.325	61.99	4.36	251.81	2.38
41.51	247.155	437.865	244.69	57.78	0	2	38.94	69.805	118.945	25.04	300.285	5.255
0	0	8.415	2.205	0	0	0	10.405	1.81	0	0	0	0
17.21	30.66	106.975	37.135	20.15	0	0	54.855	8.49	9.005	0	0	0
26.36	3.82	54.48	19.6	3.635	0	0	18.735	3.57	4.455	0	0	0
42.32	32.375	98.54	69.755	13.825	0	0.525	4.015	18.49	25.79	0.48	0	0
46.26	245.235	412.8	171.225	43.615	0	4.415	10.88	155.07	48.97	0	68.715	3.015
0	0	3.465	0	0	0	0	0.22	1.81	0	0	0	0
0	0	15.425	0.91	0	0	0	0	2.43	0	0	0	0
0	0	19.37	2.205	0	0	0	0	2.43	0	0	0	0
58.65	657.325	1916.155	449.645	106.105	1.29	1.935	56.805	90	130.815	0	680.47	2.555
43.26	265.985	610.445	251.895	36.84	1.22	1.36	109.085	52.215	80.535	2.445	491.725	1.185
3.015	0	28.445	7.9	0.165	0	0	24.92	5.515	0	0	0	0
0	0	13.08	4.805	0	0	0	3.9	2.33	0	0	0	0
5.215	0	17.425	4.325	0	0	0	0	3.37	0	0	0	0
0	0	4.495	0	0	0	0	26.695	1.595	0	0	0	0
34.555	33.445	136.225	37.43	21.09	0	0.525	33.07	20.115	17.46	0	0.07	0
30.785	49.535	131.725	32.82	22.98	0	0	1.21	12.72	4.455	0	0	0

26.36	83.59	162.875	35.015	26.365	0	0.525	31.685	10.87	9.185	0	0	0
36.265	10.96	88.65	60.49	9.705	0	2.34	30.63	13.05	18.15	4.805	0	0
37.89	5.965	68.51	49.435	6.54	0	0	35.26	8.575	16.33	3.41	0	0
20.805	13.1	73.195	43.115	7.37	0	0	27.54	3.75	5.375	0	0	4.35
50.96	101.145	202.42	192.605	38.935	0	5.235	6.67	87.575	71.12	1.705	32.9	1.715
43.68	91.32	153.68	91.535	26.415	0	2.34	8.195	40.975	46.33	1.705	6.36	0.615
46.26	56.945	132.49	73.13	22.22	0	2.34	5.1	36.625	50.045	3.41	2.575	0
36.265	47.17	126.55	92.855	23.11	0	2.34	4.56	35.53	27.58	0	6.53	0.7
50.96	98.82	231.715	120.12	31.355	0	3.48	5.63	59.47	47.815	0	21.635	1.37

pg/mg

Kidney	Kidney	Kidney	Kidney	Kidney	Kidney	Kidney	Kidney	Kidney	Kidney	Kidney	Kidney	Kidney
**kGM-CSF**	**kIFN-g**	**kIL-1a**	**kIL-1b**	**kIL-2**	**kIL-4**	**kIL-5**	**kIL-6**	**kIL-10**	**kIL-12 (p40**	**kIL-12 (p70)**	**kIL-13**	**kIL-17**
23.605	222.935	508.36	153.175	36.065	0	0	8.075	38.325	61.99	4.36	251.81	2.38
0	0	3.465	0	0	0	0	0.22	1.81	0	0	0	0
0	0	15.425	0.91	0	0	0	0	2.43	0	0	0	0
0	0	19.37	2.205	0	0	0	0	2.43	0	0	0	0

kGM-CSF	kIFN-g	kIL-1a	kIL-1b	kIL-2	kIL-4	kIL-5	kIL-6	kIL-10	kIL-12 (p40	kIL-12 (p70)	kIL-13	kIL-17
41.51	247.155	437.865	244.69	57.78	0	2	38.94	69.805	118.945	25.04	300.285	5.255
0	0	8.415	2.205	0	0	0	10.405	1.81	0	0	0	0
17.21	30.66	106.975	37.135	20.15	0	0	54.855	8.49	9.005	0	0	0
26.36	3.82	54.48	19.6	3.635	0	0	18.735	3.57	4.455	0	0	0
42.32	32.375	98.54	69.755	13.825	0	0.525	4.015	18.49	25.79	0.48	0	0
46.26	245.235	412.8	171.225	43.615	0	4.415	10.88	155.07	48.97	0	68.715	3.015
58.65	657.325	1916.155	449.645	106.105	1.29	1.935	56.805	90	130.815	0	680.47	2.555

43.26	265.985	610.445	251.895	36.84	1.22	1.36	109.085	52.215	80.535	2.445	491.725	1.185
3.015	0	28.445	7.9	0.165	0	0	24.92	5.515	0	0	0	0
0	0	13.08	4.805	0	0	0	3.9	2.33	0	0	0	0
5.215	0	17.425	4.325	0	0	0	0	3.37	0	0	0	0
0	0	4.495	0	0	0	0	26.695	1.595	0	0	0	0
34.555	33.445	136.225	37.43	21.09	0	0.525	33.07	20.115	17.46	0	0.07	0
30.785	49.535	131.725	32.82	22.98	0	0	1.21	12.72	4.455	0	0	0
26.36	83.59	162.875	35.015	26.365	0	0.525	31.685	10.87	9.185	0	0	0
36.265	10.96	88.65	60.49	9.705	0	2.34	30.63	13.05	18.15	4.805	0	0
37.89	5.965	68.51	49.435	6.54	0	0	35.26	8.575	16.33	3.41	0	0
20.805	13.1	73.195	43.115	7.37	0	0	27.54	3.75	5.375	0	0	4.35
50.96	101.145	202.42	192.605	38.935	0	5.235	6.67	87.575	71.12	1.705	32.9	1.715
43.68	91.32	153.68	91.535	26.415	0	2.34	8.195	40.975	46.33	1.705	6.36	0.615
46.26	56.945	132.49	73.13	22.22	0	2.34	5.1	36.625	50.045	3.41	2.575	0
36.265	47.17	126.55	92.855	23.11	0	2.34	4.56	35.53	27.58	0	6.53	0.7
50.96	98.82	231.715	120.12	31.355	0	3.48	5.63	59.47	47.815	0	21.635	1.37

kidney IP-1	kidney KC	kidney MC	kidney MIC	kidney MIF	kidney TNF	kidney VEGF
Kidney	Kidney	Kidney	Kidney	Kidney	Kidney	Kidney
kidney IP-1	kidney KC	kidney MC	kidney MIC	kidney MIF	kidney TNF	kidney VEGF
82.88	10.825	8.965	580.025	16.735	2.145	56.365
135.455	543.545	121.48	524.07	31.785	2.235	96.83
2317.065	4770.065	1612.5	2943.105	147.58	2.38	98.26
5000.0	5000.0	2118.17	24877.6	249.54	4.8	77.17
2129.995	2389.965	428.535	6598.065	61.615	0.74	20.635
1238.17	1386.595	283.575	8455.09	81.025	1.055	12.535
211.755	152.71	137.15	583.41	13.005	0	0.425
48.99	7.23	9.48	420.125	10.92	2.17	62.17
1058.45	4725.385	983.05	546.375	132.83	3.47	90.315
2071.84	2438.125	1069.175	3974.785	139.9	2.365	63
1578.365	1779.58	1278.03	12753.67	152.845	3.22	47.745
2573.285	1113.37	328.68	8599.65	71.11	3.2	20.855
165.195	20.87	54.385	2518.32	37.105	2.925	71.13
164.925	26.55	52.84	913.23	23.935	0.605	19.215
64.765	11.06	11.465	749.895	47.76	2.2	99.5
965.065	4703.79	787.81	408.67	153.4	4.405	77.33
1909.835	4974.19	1756.275	4504.77	178.66	2.255	84.035
2575.69	2264.375	901.18	5340.695	83.095	0	37.57
2908.035	1947.02	406.035	8426.85	60.13	2.49	27.92
441.14	197.865	93.43	10010.66	44.82	0	19.74
418.845	202.84	61.135	1281.34	9.785	0	4.245
51.795	4.005	11.945	688.27	18.985	2.22	99.45
640.94	4602.97	653.615	325.12	119.57	2.975	76.45
5000.0	5000.0	1694.16	8260.13	232.095	6.33	90.25
1561.215	1958.14	1148.02	5716.02	36.535	0	37.39
3159.905	1899.49	355.275	9676.105	99.555	4.07	27.05

197.495	135.425	130.715	1816.77	21.325	0	9.035
379.67	389.11	76.195	1902.675	22.68	0	0.215

159.295	53.28	76.485	381.28	83.54	3.37	104.06
820.625	5000.0	637.745	646.89	206.84	18.84	157.925
5000.0	5000.0	2223.385	13779.69	302.8	5.915	103.08
557.2	100.25	78.33	5219.34	22.88	0	80.7
17540.54	3652.96	842.825	9484.07	144.94	1.855	31.035
2129.085	3247.215	460.145	5746.87	34.01	0	11.245
210.86	18.935	40.495	1408.99	8.92	0	49.665

852.665	5000.0	957.845	374.06	182.25	12.24	150.825
5000.0	5000.0	1760.955	9110.17	240.955	5.01	107.725
4245.365	2527.375	991.29	6508.58	178.505	0	91.675
10087.72	3366.615	632.93	8780.24	85.39	1.26	18.565
**2020.48**	**2452.52**	**202.525**	**5828.205**	**22.88**	**0**	**4.185**
**366.75**	**112.955**	**55.87**	**2321.31**	**32.18**	**0**	**3.61**

**Kidney**	**Kidney**	**Kidney**	**Kidney**	**Kidney**	**Kidney**	**Kidney**
**kIP-10**	**kKC**	**kMCP-1**	**kMIG**	**kMIP-1a**	**kTNFa**	**kVEGF**
82.88	10.825	8.965	580.025	16.735	2.145	56.365
48.99	7.23	9.48	420.125	10.92	2.17	62.17
64.765	11.06	11.465	749.895	47.76	2.2	99.5
51.795	4.005	11.945	688.27	18.985	2.22	99.45
159.295	53.28	76.485	381.28	83.54	3.37	104.06

**kIP-10**	**kKC**	**kMCP-1**	**kMIG**	**kMIP-1a**	**kTNFa**	**kVEGF**
135.455	543.545	121.48	524.07	31.785	2.235	96.83
2317.065	4770.065	1612.5	2943.105	147.58	2.38	98.26

5000.0	5000.0	2118.17	24877.6	249.54	4.8	77.17
2129.995	2389.965	428.535	6598.065	61.615	0.74	20.635
1238.17	1386.595	283.575	8455.09	81.025	1.055	12.535
211.755	152.71	137.15	583.41	13.005	0	0.425
1058.45	4725.385	983.05	546.375	132.83	3.47	90.315
2071.84	2438.125	1069.175	3974.785	139.9	2.365	63
1578.365	1779.58	1278.03	12753.67	152.845	3.22	47.745
2573.285	1113.37	328.68	8599.65	71.11	3.2	20.855
165.195	20.87	54.385	2518.32	37.105	2.925	71.13
164.925	26.55	52.84	913.23	23.935	0.605	19.215
965.065	4703.79	787.81	408.67	153.4	4.405	77.33
1909.835	4974.19	1756.275	4504.77	178.66	2.255	84.035
2575.69	2264.375	901.18	5340.695	83.095	0	37.57
2908.035	1947.02	406.035	8426.85	60.13	2.49	27.92
441.14	197.865	93.43	10010.66	44.82	0	19.74
418.845	202.84	61.135	1281.34	9.785	0	4.245
640.94	4602.97	653.615	325.12	119.57	2.975	76.45
5000.0	5000.0	1694.16	8260.13	232.095	6.33	90.25
1561.215	1958.14	1148.02	5716.02	36.535	0	37.39
3159.905	1899.49	355.275	9676.105	99.555	4.07	27.05
197.495	135.425	130.715	1816.77	21.325	0	9.035
379.67	389.11	76.195	1902.675	22.68	0	0.215
820.625	5000.0	637.745	646.89	206.84	18.84	157.925
5000.0	5000.0	2223.385	13779.69	302.8	5.915	103.08
557.2	100.25	78.33	5219.34	22.88	0	80.7
17540.54	3652.96	842.825	9484.07	144.94	1.855	31.035
2129.085	3247.215	460.145	5746.87	34.01	0	11.245
210.86	18.935	40.495	1408.99	8.92	0	49.665
852.665	5000.0	957.845	374.06	182.25	12.24	150.825

5000.0	5000.0	1760.955	9110.17	240.955	5.01	107.725
4245.365	2527.375	991.29	6508.58	178.505	0	91.675
10087.72	3366.615	632.93	8780.24	85.39	1.26	18.565
**2020.48**	**2452.52**	**202.525**	**5828.205**	**22.88**	**0**	**4.185**
**366.75**	**112.955**	**55.87**	**2321.31**	**32.18**	**0**	**3.61**

**kidney IP-1**	**kidney KC**	**kidney MC**	**kidney MI(**	**kidney MIF**	**kidney TNF**	**kidney VEGF**
**Kidney**	**Kidney**	**Kidney**	**Kidney**	**Kidney**	**Kidney**	**Kidney**
**kidney IP-1**	**kidney KC**	**kidney MC**	**kidney MI(**	**kidney MIF**	**kidney TNF**	**kidney VEGF**
369.83	42.47	27.265	1609.335	42.83	1.05	85.995
533.31	1242.425	143.09	1355.285	106.13	5.125	128.02
491.83	444.365	269.635	1996.69	20.755	0	132.86
583.12	213.485	210.935	473.31	25.9	3.875	32.295
547.345	79.21	301.295	2483.195	27.27	2.35	103.93
90.15	41.305	45.17	1228.06	37.94	3.29	62.25
66.81	98.34	45.17	424.78	45.86	4.27	90.46
79.08	14.985	4.39	709.665	0	0	150.865
64.16	5.36	0	966.385	0	0	104.52
51.97	3.655	3.14	435.23	0	0.07	139.045
480.54	1350.95	509.82	653.86	85.985	4.94	122.13
426.695	1502.3	557.08	518.355	60.985	3.515	85.42
499.16	2148.545	687.235	1026	10.23	0.445	148.025
441.985	156.545	221.775	1738.935	18.67	0	104.745
141.135	22.56	42.045	499.425	0	0.1	198.505
482.645	746.175	346.995	1323.205	0	0	119.085
1010.395	170.11	236.85	598.85	29.875	3.105	38.66
25.125	29.69	32.29	90.065	25.9	1.735	48.69

642.105	148.375	163.97	638.435	25.9	2.35	47.465
388.92	73.05	189.11	3266.135	42.035	2.58	47.09
899.455	116.585	392.7	3434.875	40.025	3.945	95.25
321.01	101.76	236.055	2472.155	25.9	0.325	76.205
69.66	100.255	51.3	370.945	56.17	4.95	29.16
112.855	68.535	54.665	1532.455	43.975	3.835	71.22
100.48	57.785	39.615	1083.75	37.94	3.29	65.705
74.56	58.985	45.17	428.8	38.99	2.35	117.515
50.85	71.355	48.935	250.26	42.035	2.655	135.135

Kidney	Kidney	Kidney	Kidney	Kidney	Kidney	Kidney
**kIP-10**	**kKC**	**kMCP-1**	**kMIG**	**kMIP-1a**	**kTNFa**	**kVEGF**
369.83	42.47	27.265	1609.335	42.83	1.05	85.995
79.08	14.985	4.39	709.665	0	0	150.865
64.16	5.36	0	966.385	0	0	104.52
51.97	3.655	3.14	435.23	0	0.07	139.045

kIP-10	kKC	kMCP-1	kMIG	kMIP-1a	kTNFa	kVEGF
533.31	1242.425	143.09	1355.285	106.13	5.125	128.02
491.83	444.365	269.635	1996.69	20.755	0	132.86
583.12	213.485	210.935	473.31	25.9	3.875	32.295
547.345	79.21	301.295	2483.195	27.27	2.35	103.93
90.15	41.305	45.17	1228.06	37.94	3.29	62.25
66.81	98.34	45.17	424.78	45.86	4.27	90.46
480.54	1350.95	509.82	653.86	85.985	4.94	122.13

426.695	1502.3	557.08	518.355	60.985	3.515	85.42
499.16	2148.545	687.235	1026	10.23	0.445	148.025
441.985	156.545	221.775	1738.935	18.67	0	104.745
141.135	22.56	42.045	499.425	0	0.1	198.505
482.645	746.175	346.995	1323.205	0	0	119.085
1010.395	170.11	236.85	598.85	29.875	3.105	38.66
25.125	29.69	32.29	90.065	25.9	1.735	48.69
642.105	148.375	163.97	638.435	25.9	2.35	47.465
388.92	73.05	189.11	3266.135	42.035	2.58	47.09
899.455	116.585	392.7	3434.875	40.025	3.945	95.25
321.01	101.76	236.055	2472.155	25.9	0.325	76.205
69.66	100.255	51.3	370.945	56.17	4.95	29.16
112.855	68.535	54.665	1532.455	43.975	3.835	71.22
100.48	57.785	39.615	1083.75	37.94	3.29	65.705
74.56	58.985	45.17	428.8	38.99	2.35	117.515
50.85	71.355	48.935	250.26	42.035	2.655	135.135
