# Supplementary material for: A computational analysis of dynamic, multi-organ inflammatory crosstalk induced by endotoxin in mice
Source: PLoS Comput Biol. 2018 Nov 6;14(11):e1006582. doi: 10.1371/journal.pcbi.1006582 (PMC6239343; doi:10.1371/journal.pcbi.1006582)
Supplement: S1 MATLAB Code — (DOCX) [file pcbi.1006582.s004.docx]

MATLAB Code of PCA and TI-PCA

function varargout = PCAGUI(varargin)

% PCAGUI MATLAB code for PCAGUI.fig

% PCAGUI, by itself, creates a new PCAGUI or raises the existing

% singleton*.

%

% H = PCAGUI returns the handle to a new PCAGUI or the handle to

% the existing singleton*.

%

% PCAGUI('CALLBACK',hObject,eventData,handles,...) calls the local

% function named CALLBACK in PCAGUI.M with the given input arguments.

%

% PCAGUI('Property','Value',...) creates a new PCAGUI or raises the

% existing singleton*. Starting from the left, property value pairs are

% applied to the GUI before PCAGUI_OpeningFcn gets called. An

% unrecognized property name or invalid value makes property application

% stop. All inputs are passed to PCAGUI_OpeningFcn via varargin.

%

% *See GUI Options on GUIDE's Tools menu. Choose "GUI allows only one

% instance to run (singleton)".

%

% See also: GUIDE, GUIDATA, GUIHANDLES

% Edit the above text to modify the response to help PCAGUI

% Last Modified by GUIDE v2.5 29-Nov-2013 23:03:31

% Begin initialization code - DO NOT EDIT

gui_Singleton = 1;

gui_State = struct('gui_Name', mfilename, ...

'gui_Singleton', gui_Singleton, ...

'gui_OpeningFcn', @PCAGUI_OpeningFcn, ...

'gui_OutputFcn', @PCAGUI_OutputFcn, ...

'gui_LayoutFcn', [] , ...

'gui_Callback', []);

if nargin && ischar(varargin{1})

gui_State.gui_Callback = str2func(varargin{1});

end

if nargout

[varargout{1:nargout}] = gui_mainfcn(gui_State, varargin{:});

else

gui_mainfcn(gui_State, varargin{:});

end

% End initialization code - DO NOT EDIT

% --- Executes just before PCAGUI is made visible.

function PCAGUI_OpeningFcn(hObject, eventdata, handles, varargin)

% This function has no output args, see OutputFcn.

% hObject handle to figure

% eventdata reserved - to be defined in a future version of MATLAB

% handles structure with handles and user data (see GUIDATA)

% varargin command line arguments to PCAGUI (see VARARGIN)

% Choose default command line output for PCAGUI

handles.output = hObject;

% Update handles structure

guidata(hObject, handles);

% UIWAIT makes PCAGUI wait for user response (see UIRESUME)

% uiwait(handles.figure1);

addpath(genpath('F:\Users\Qi Mi\Documents\My project\graphViz4Matlab'))

% --- Outputs from this function are returned to the command line.

function varargout = PCAGUI_OutputFcn(hObject, eventdata, handles)

% varargout cell array for returning output args (see VARARGOUT);

% hObject handle to figure

% eventdata reserved - to be defined in a future version of MATLAB

% handles structure with handles and user data (see GUIDATA)

% Get default command line output from handles structure

varargout{1} = handles.output;

% --- Executes on button press in pushbutton1.

function pushbutton1_Callback(hObject, eventdata, handles)

% hObject handle to pushbutton1 (see GCBO)

% eventdata reserved - to be defined in a future version of MATLAB

% handles structure with handles and user data (see GUIDATA)

global raw

global data

global txt

[filename path]=uigetfile('*.xlsx');

[data txt raw] = xlsread(filename, -1);

set(handles.uitable1,'Data',raw)

% --- Executes on button press in pushbutton2.

function pushbutton2_Callback(hObject, eventdata, handles)

% hObject handle to pushbutton2 (see GCBO)

% eventdata reserved - to be defined in a future version of MATLAB

% handles structure with handles and user data (see GUIDATA)

numberoftime = get(handles.popupmenu5,'Value');

timeinterval = get(handles.popupmenu6,'Value');

checkboxvalue = get(handles.checkbox2,'Value');

corrthreshold = get(handles.edit4,'String');

corrthreshold = str2double(corrthreshold);

projecttitle = get(handles.edit5,'String');

global raw

global data

global txt

global rawControl

if checkboxvalue == 0

rawControl = []; % clear the control data. Matlab seems to save this value from previous run.

end

label = raw(1,3:end);

days = raw(:,2); % The Time information is saved in the second column

k = (numberoftime);

kt = (timeinterval);

newdata =raw(1,1:end);

for i=1:k

p= cell(size(raw,1),1);

%para= cell(size(raw,1),1);

p(1:end) = days(i+1);

%para(1:end) = {'4'};

index = cellfun(@strcmp, days,p);

newdata = [newdata

raw(index,1:end)];

dynadata{i}= raw(index,3:end);

end

%newdata = newdata(2:end,:);

set(handles.uitable1,'Data',newdata)

%% Calculate the adjacent matrix, there are total k - kt + 1 number of network

for i=1: (k-kt+1)

dynaMatrix = [];

good=[];

dynatitle{i} = [days{i+1} '-' days{i+kt}];

for j = 1:kt

dynaMatrix = [dynaMatrix

dynadata{i+j-1} ];

end

dynaMatrix = cell2mat(dynaMatrix);

%dynaMatrixSave = dynaMatrix;

dynaMatrix = corr(dynaMatrix);

dynaMatrix = dynaMatrix - eye(size(dynaMatrix,1));

adjMatrix = zeros(size(dynaMatrix,1));

[i1 i2] = find(abs(dynaMatrix) >= corrthreshold );

for e=1:length(i1)

adjMatrix(i1(e),i2(e))=1;

end

%% Select the significant altered nodes for display

if ~isempty(rawControl)

% [n1, m1] = size(dynaMatrixSave);

for r=1:length(label)

for t = 1:kt

tempdata=cell2mat(dynadata{i+t-1}) ;

tempcontrol= cell2mat(rawControl(2:end,r+2));

h(t) = ttest2(tempcontrol, tempdata(:,r));

end

if sum(h) > 0

good =[good r];

end

end

adjMatrix = adjMatrix(good,good);

label = label(good);

else

good = 1:length(label);

end

if ~isempty(good)

%[i11 i22] = find(dynaMatrix >= corrthreshold );

ee = length(label);

if ee > 1

numberofedge(i) = sum(sum(adjMatrix)) /2;

networkcomplex(i) = numberofedge(i)*2 /(ee*(ee-1))*ee;

else

networkcomplex(i)= 0;

end

dynaMatrix = dynaMatrix(good,good);

[i11 i22] = find(dynaMatrix <= -1*corrthreshold );

edgecolor = [];

for k2=1:length(i11)

edgecolor = [ edgecolor

[label(i11(k2)) label(i22(k2)) 'r' ]];

end

if ~isempty(edgecolor)

graphViz4Matlab('-adjMat',adjMatrix,'-nodeLabels',label,'-edgeColors',edgecolor)

else

graphViz4Matlab('-adjMat',adjMatrix,'-nodeLabels',label)

end

title([projecttitle ' DyNA' dynatitle{i}],'fontsize',15)

%% Write number of nodes information into a file

filename = [projecttitle ' DyNA'];

nodeconnection = [];

for k3=1:length(label)

nodeconnection = [nodeconnection

label(k3) sum(adjMatrix(k3,:))];

end

xlswrite(filename,nodeconnection,dynatitle{i})

else

networkcomplex(i) = 0;

end

end

figure

plot(networkcomplex,'LineWidth',2,'Marker','o','MarkerSize',10)

title([projecttitle ' DyNA Network Complexity'],'fontsize',15)

set(gca,'XTick',[1:i])

set(gca,'XTickLabel',dynatitle,'fontsize',15);

ylabel('Network Complexity')

function edit1_Callback(hObject, eventdata, handles)

% hObject handle to edit1 (see GCBO)

% eventdata reserved - to be defined in a future version of MATLAB

% handles structure with handles and user data (see GUIDATA)

% Hints: get(hObject,'String') returns contents of edit1 as text

% str2double(get(hObject,'String')) returns contents of edit1 as a double

% --- Executes during object creation, after setting all properties.

function edit1_CreateFcn(hObject, eventdata, handles)

% hObject handle to edit1 (see GCBO)

% eventdata reserved - to be defined in a future version of MATLAB

% handles empty - handles not created until after all CreateFcns called

% Hint: edit controls usually have a white background on Windows.

% See ISPC and COMPUTER.

if ispc && isequal(get(hObject,'BackgroundColor'), get(0,'defaultUicontrolBackgroundColor'))

set(hObject,'BackgroundColor','white');

end

function edit2_Callback(hObject, eventdata, handles)

% hObject handle to edit2 (see GCBO)

% eventdata reserved - to be defined in a future version of MATLAB

% handles structure with handles and user data (see GUIDATA)

% Hints: get(hObject,'String') returns contents of edit2 as text

% str2double(get(hObject,'String')) returns contents of edit2 as a double

% --- Executes during object creation, after setting all properties.

function edit2_CreateFcn(hObject, eventdata, handles)

% hObject handle to edit2 (see GCBO)

% eventdata reserved - to be defined in a future version of MATLAB

% handles empty - handles not created until after all CreateFcns called

% Hint: edit controls usually have a white background on Windows.

% See ISPC and COMPUTER.

if ispc && isequal(get(hObject,'BackgroundColor'), get(0,'defaultUicontrolBackgroundColor'))

set(hObject,'BackgroundColor','white');

end

function edit4_Callback(hObject, eventdata, handles)

% hObject handle to edit4 (see GCBO)

% eventdata reserved - to be defined in a future version of MATLAB

% handles structure with handles and user data (see GUIDATA)

% Hints: get(hObject,'String') returns contents of edit4 as text

% str2double(get(hObject,'String')) returns contents of edit4 as a double

% --- Executes during object creation, after setting all properties.

function edit4_CreateFcn(hObject, eventdata, handles)

% hObject handle to edit4 (see GCBO)

% eventdata reserved - to be defined in a future version of MATLAB

% handles empty - handles not created until after all CreateFcns called

% Hint: edit controls usually have a white background on Windows.

% See ISPC and COMPUTER.

if ispc && isequal(get(hObject,'BackgroundColor'), get(0,'defaultUicontrolBackgroundColor'))

set(hObject,'BackgroundColor','white');

end

% --------------------------------------------------------------------

function Help_Callback(hObject, eventdata, handles)

% hObject handle to Help (see GCBO)

% eventdata reserved - to be defined in a future version of MATLAB

% handles structure with handles and user data (see GUIDATA)

% --- If Enable == 'on', executes on mouse press in 5 pixel border.

% --- Otherwise, executes on mouse press in 5 pixel border or over text4.

function text4_ButtonDownFcn(hObject, eventdata, handles)

% hObject handle to text4 (see GCBO)

% eventdata reserved - to be defined in a future version of MATLAB

% handles structure with handles and user data (see GUIDATA)

% --- Executes on button press in pushbutton3.

function pushbutton3_Callback(hObject, eventdata, handles)

% hObject handle to pushbutton3 (see GCBO)

% eventdata reserved - to be defined in a future version of MATLAB

% handles structure with handles and user data (see GUIDATA)

global raw

global data

global txt

[filename, path]=uigetfile('*.xlsx');

[data, txt, raw] = xlsread(filename, -1);

set(handles.uitable1,'Data',raw)

% --- Executes on selection change in popupmenu5.

function popupmenu5_Callback(hObject, eventdata, handles)

% hObject handle to popupmenu5 (see GCBO)

% eventdata reserved - to be defined in a future version of MATLAB

% handles structure with handles and user data (see GUIDATA)

% Hints: contents = cellstr(get(hObject,'String')) returns popupmenu5 contents as cell array

% contents{get(hObject,'Value')} returns selected item from popupmenu5

% --- Executes during object creation, after setting all properties.

function popupmenu5_CreateFcn(hObject, eventdata, handles)

% hObject handle to popupmenu5 (see GCBO)

% eventdata reserved - to be defined in a future version of MATLAB

% handles empty - handles not created until after all CreateFcns called

% Hint: popupmenu controls usually have a white background on Windows.

% See ISPC and COMPUTER.

if ispc && isequal(get(hObject,'BackgroundColor'), get(0,'defaultUicontrolBackgroundColor'))

set(hObject,'BackgroundColor','white');

end

% --- Executes on selection change in popupmenu6.

function popupmenu6_Callback(hObject, eventdata, handles)

% hObject handle to popupmenu6 (see GCBO)

% eventdata reserved - to be defined in a future version of MATLAB

% handles structure with handles and user data (see GUIDATA)

% Hints: contents = cellstr(get(hObject,'String')) returns popupmenu6 contents as cell array

% contents{get(hObject,'Value')} returns selected item from popupmenu6

% --- Executes during object creation, after setting all properties.

function popupmenu6_CreateFcn(hObject, eventdata, handles)

% hObject handle to popupmenu6 (see GCBO)

% eventdata reserved - to be defined in a future version of MATLAB

% handles empty - handles not created until after all CreateFcns called

% Hint: popupmenu controls usually have a white background on Windows.

% See ISPC and COMPUTER.

if ispc && isequal(get(hObject,'BackgroundColor'), get(0,'defaultUicontrolBackgroundColor'))

set(hObject,'BackgroundColor','white');

end

% --- Executes on button press in pushbutton5.

function pushbutton5_Callback(hObject, eventdata, handles)

% hObject handle to pushbutton5 (see GCBO)

% eventdata reserved - to be defined in a future version of MATLAB

% handles structure with handles and user data (see GUIDATA)

global rawControl

global dataControl

global txtControl

[filename, path]=uigetfile('*.xlsx');

[dataControl, txtControl, rawControl] = xlsread(filename, -1);

set(handles.uitable1,'Data',rawControl)

% --- Executes on button press in checkbox2.

function checkbox2_Callback(hObject, eventdata, handles)

% hObject handle to checkbox2 (see GCBO)

% eventdata reserved - to be defined in a future version of MATLAB

% handles structure with handles and user data (see GUIDATA)

% Hint: get(hObject,'Value') returns toggle state of checkbox2

function edit5_Callback(hObject, eventdata, handles)

% hObject handle to edit5 (see GCBO)

% eventdata reserved - to be defined in a future version of MATLAB

% handles structure with handles and user data (see GUIDATA)

% Hints: get(hObject,'String') returns contents of edit5 as text

% str2double(get(hObject,'String')) returns contents of edit5 as a double

% --- Executes during object creation, after setting all properties.

function edit5_CreateFcn(hObject, eventdata, handles)

% hObject handle to edit5 (see GCBO)

% eventdata reserved - to be defined in a future version of MATLAB

% handles empty - handles not created until after all CreateFcns called

% Hint: edit controls usually have a white background on Windows.

% See ISPC and COMPUTER.

if ispc && isequal(get(hObject,'BackgroundColor'), get(0,'defaultUicontrolBackgroundColor'))

set(hObject,'BackgroundColor','white');

end

% --- Executes on button press in pushbutton6.

function pushbutton6_Callback(hObject, eventdata, handles)

% hObject handle to pushbutton6 (see GCBO)

% eventdata reserved - to be defined in a future version of MATLAB

% handles structure with handles and user data (see GUIDATA)

global raw

global data

global txt

[filename, path]=uigetfile('*.xlsx');

[data txt raw] = xlsread(filename, -1); % Reads in your data from an excel

% file. This program expects the

% first two columns to be sample

% names and timepoints,

% respectively and the first row

% to be a header with

% cytokine names.

set(handles.uitable1,'Data',raw)

% --- Executes during object creation, after setting all properties.

function edit6_CreateFcn(hObject, eventdata, handles)

% hObject handle to edit6 (see GCBO)

% eventdata reserved - to be defined in a future version of MATLAB

% handles empty - handles not created until after all CreateFcns called

% Hint: edit controls usually have a white background on Windows.

% See ISPC and COMPUTER.

if ispc && isequal(get(hObject,'BackgroundColor'), get(0,'defaultUicontrolBackgroundColor'))

set(hObject,'BackgroundColor','white');

end

function edit6_Callback(hObject, eventdata, handles)

% hObject handle to edit5 (see GCBO)

% eventdata reserved - to be defined in a future version of MATLAB

% handles structure with handles and user data (see GUIDATA)

% Hints: get(hObject,'String') returns contents of edit5 as text

% str2double(get(hObject,'String')) returns contents of edit5 as a double

% --- Executes on button press in pushbutton8.

function pushbutton8_Callback(hObject, eventdata, handles)

% hObject handle to pushbutton8 (see GCBO)

% eventdata reserved - to be defined in a future version of MATLAB

% handles structure with handles and user data (see GUIDATA)

global raw

global data

projecttitle = get(handles.edit6,'String');

head = size(raw,2) - size(data,2);

sampnames = cell(size(raw,1)-1,1);

for j = 2:size(raw,1)

% for i = 1:head-2

% sampnames(j-1) = strcat(sampnames(j-1),raw(j,i),' - ');

% end

sampnames(j-1) = strcat(sampnames(j-1),raw(j,head-1));

end

cytokines = raw(1, head+1:end);

N = length(sampnames);

C = size(data,2);

if get(handles.radiobutton1,'Value') == 1

PctVar = str2double(get(handles.edit4,'String'));%input('What percentage of variance shall we cover in the principal components? \n Enter a number between 0 and 100:');

PctVar = PctVar/100;

ComponentsCutoff = 0;

else

NumComp = get(handles.popupmenu6,'Value'); %input('How many principal components shall we consider? \n Enter a number between 1 and the number of cytokines:');

ComponentsCutoff =1;

if NumComp > C

x = sprintf('%d',C);

NumComp = input(strcat('That is too many components. Choose a number <= ',' ',x,':'));

end

end

%% Create a list of unique Sample Names

for k = 1:N

if ischar(sampnames{k});

else sampnames{k} = num2str(sampnames{k});

end

end

% Code from Cordlia's original PCA. but it will has error if the data sheet

% has blank between the first and second samples, since Matlab seems look

% NaN as a string

% i = 1;

% j = 1;

% samples(1) = sampnames(1);

% while j <= N

% for k = 1:i

% r(k) = strcmp(sampnames{j}, samples{k});

%

% end

% rows = find(r);

% if isempty(rows);

% i = i+1;

% samples(i) = sampnames(j);

% j = j+1;

% else j = j+1;

% end

% end

i = 0;

for j=1:N

if ~strcmp(sampnames{j},'NaN')

samples{i+1,1} = sampnames{j}; % remove NaN from sampnames

i = i + 1;

end

end

samples= unique(samples,'stable');

S = length(samples);

% p = cellstr('mean');

% samples(S+1) = p;

%% Normalize data over each sample, for each cytokine. Then find variance.

for j = 1:S

samp = samples(j);

for i = 1:N

rows(i) = strcmp(char(samp), char(sampnames(i)));

end

rows = find(rows);

for i = 1:C

n = norm(data(rows, i));

if n ~= 0

NormData(rows,i) = data(rows,i)/n;

else

NormData(rows,i) = data(rows,i);

end

SampleVar(j,i) = var(NormData(rows,i));

end

if get(handles.checkbox6,'Value') == 1 % We will do the subject specific PCA and we should skip the sliding window PCA part

specificMatrix{j} = NormData(rows,:);

end

end

SampleVar(j+1,:) = mean(SampleVar(1:j,:));

SampleVar(j+2,:) = -SampleVar(j+1,:);

%% Sort NormData by Time points, code from DyNA by Qi Mi

% Get data from Normalization

if get(handles.checkbox6,'Value') == 0 % We only prform time sorting if not doing subject specific PCA

raw(2:end,3:end) = num2cell(NormData);

numberoftime = get(handles.popupmenu7,'Value');

timeinterval = get(handles.popupmenu8,'Value');

label = raw(1,3:end);

days = raw(:,2); % The Time information is saved in the second column

k = (numberoftime);

kt = (timeinterval);

newdata =raw(1,1:end);

for i=1:k

p= cell(size(raw,1),1);

%para= cell(size(raw,1),1);

p(1:end) = days(i+1);

%para(1:end) = {'4'};

index = cellfun(@strcmp, days,p);

newdata = [newdata

raw(index,1:end)];

dyPCAdata{i}= raw(index,3:end);

end

%newdata = newdata(2:end,:);

set(handles.uitable1,'Data',newdata)

%% Perform PCA and organize data

for i1=1: (k-kt+1)

dyPCAMatrix = [];

dyPCAtitle{i1} = [days{i1+1} '- ' days{i1+kt}];

for j = 1:kt

dyPCAMatrix = [dyPCAMatrix

dyPCAdata{i1+j-1} ];

end

dyPCAMatrix = cell2mat(dyPCAMatrix);

[vecs x eigs] = princomp(dyPCAMatrix);

eigs(:,2) = eigs(:,1)/sum(eigs(:,1));

for i = 1:C

eigs(i,3) = sum(eigs(1:i,2));

end

%rows = 1:3;

if ComponentsCutoff == 0

last = find(eigs(:,3) > PctVar);

elseif ComponentsCutoff == 1

last = NumComp;

end

rows = 1:last(1);

factors = eigs(rows,1);

vectors = vecs(:,rows);

R= length(rows);

for i = 1:R

top(:,i) = abs(vectors(:,i)*factors(i));

end

for j = 1:C

top(j,i+1) = -1*sum(top(j,1:i));

end

top2 = top;

components = {};

for k = 1:R

components(k) = {strcat('Component ', num2str(k))};

end

%% Make Plots: PCA then Variance

% PCA plot:

[top PCAInd] = sortrows(top,i+1);

pcaLabel = cytokines(PCAInd);

h1 = figure, bar(top(:,1:i), 'stack');

xlim([0 C+1]);

set(gcf, 'PaperOrientation', 'Landscape');

set(gcf, 'PaperPosition', [0.25, 0.25, 10.5, 8]);

set(gca, 'FontName', 'Arial Narrow');

set(gca, 'FontSize', 8);

set(gca, 'XTickLabel', pcaLabel);

xticklabel_rotate([1:C],45,pcaLabel,'interpreter', 'none');

%t = title (char(tx1));

titlename = [projecttitle ' PCA ' dyPCAtitle{i1}];

t = title(titlename);

set(t, 'FontName', 'Arial');

set(t, 'FontSize', 13);

set(t, 'FontWeight', 'bold');

colormap Summer;

legend(components);

%% save the PCA output to excel file

tempoutput = [pcaLabel' num2cell(top(:,1:i))];

xlswrite([titlename '.xlsx'], tempoutput)

saveas(h1, titlename, 'tiff');

end

end

%% The rest code is for subject specific PCA and Clustering

columnlabel = cytokines;

for i2=1:length(samples)

rowlabel{i2} = [projecttitle '-' num2str(i2)];

end

if get(handles.checkbox6,'Value') == 1

specificClustering = [];

for j1=1:S % loop through each subject

[vecs x eigs] = princomp(specificMatrix{j1});

eigs(:,2) = eigs(:,1)/sum(eigs(:,1));

for i = 1:C

eigs(i,3) = sum(eigs(1:i,2));

end

%rows = 1:3;

if ComponentsCutoff == 0

last = find(eigs(:,3) > PctVar);

elseif ComponentsCutoff == 1

last = NumComp;

end

rows = 1:last(1);

factors = eigs(rows,1);

vectors = vecs(:,rows);

R= length(rows);

for i = 1:R

top(:,i) = abs(vectors(:,i)*factors(i));

end

for j = 1:C

top(j,i+1) = -1*sum(top(j,1:i));

end

top2 = top;

specificClustering = [specificClustering -top2(:,i+1)];

components = {};

for k = 1:R

components(k) = {strcat('Component ', num2str(k))};

end

%% Make Plots: PCA then Variance

% PCA plot:

if get(handles.checkbox7,'Value') == 1

[top PCAInd] = sortrows(top,i+1);

pcaLabel = cytokines(PCAInd);

h1 = figure, bar(top(:,1:i), 'stack');

xlim([0 C+1]);

set(gcf, 'PaperOrientation', 'Landscape');

set(gcf, 'PaperPosition', [0.25, 0.25, 10.5, 8]);

set(gca, 'FontName', 'Arial Narrow');

set(gca, 'FontSize', 8);

set(gca, 'XTickLabel', pcaLabel);

xticklabel_rotate([1:C],45,pcaLabel,'interpreter', 'none');

%t = title (char(tx1));

titlename = [rowlabel{j1} ' PCA '];

t = title(titlename);

set(t, 'FontName', 'Arial');

set(t, 'FontSize', 13);

set(t, 'FontWeight', 'bold');

colormap Summer;

legend(components);

saveas(h1, titlename, 'tiff');

end

%specificcluster=[top()]

end

% Clustering for subject specific PCA

cgo = clustergram(specificClustering','Cluster',1,'RowPDist','corr','Columnlabels',columnlabel,'Rowlabels',rowlabel) ;

end

%set(cgo,'Colormap',redbluecmap);

set(cgo,'Standardize',2)

projecttitle = get(handles.edit6,'String');

addTitle(cgo,[projecttitle ' Subject Specific Clustering'],'fontsize',15)

save(projecttitle,'columnlabel','rowlabel','specificClustering')

% --- Executes on button press in radiobutton1.

function radiobutton1_Callback(hObject, eventdata, handles)

% hObject handle to radiobutton1 (see GCBO)

% eventdata reserved - to be defined in a future version of MATLAB

% handles structure with handles and user data (see GUIDATA)

% Hint: get(hObject,'Value') returns toggle state of radiobutton1

set(handles.radiobutton3,'Value',0)

% --- Executes on button press in radiobutton3.

function radiobutton3_Callback(hObject, eventdata, handles)

% hObject handle to radiobutton3 (see GCBO)

% eventdata reserved - to be defined in a future version of MATLAB

% handles structure with handles and user data (see GUIDATA)

% Hint: get(hObject,'Value') returns toggle state of radiobutton3

set(handles.radiobutton1,'Value',0)

% --- Executes on selection change in popupmenu7.

function popupmenu7_Callback(hObject, eventdata, handles)

% hObject handle to popupmenu7 (see GCBO)

% eventdata reserved - to be defined in a future version of MATLAB

% handles structure with handles and user data (see GUIDATA)

% Hints: contents = cellstr(get(hObject,'String')) returns popupmenu7 contents as cell array

% contents{get(hObject,'Value')} returns selected item from popupmenu7

% --- Executes during object creation, after setting all properties.

function popupmenu7_CreateFcn(hObject, eventdata, handles)

% hObject handle to popupmenu7 (see GCBO)

% eventdata reserved - to be defined in a future version of MATLAB

% handles empty - handles not created until after all CreateFcns called

% Hint: popupmenu controls usually have a white background on Windows.

% See ISPC and COMPUTER.

if ispc && isequal(get(hObject,'BackgroundColor'), get(0,'defaultUicontrolBackgroundColor'))

set(hObject,'BackgroundColor','white');

end

% --- Executes on selection change in popupmenu8.

function popupmenu8_Callback(hObject, eventdata, handles)

% hObject handle to popupmenu8 (see GCBO)

% eventdata reserved - to be defined in a future version of MATLAB

% handles structure with handles and user data (see GUIDATA)

% Hints: contents = cellstr(get(hObject,'String')) returns popupmenu8 contents as cell array

% contents{get(hObject,'Value')} returns selected item from popupmenu8

% --- Executes during object creation, after setting all properties.

function popupmenu8_CreateFcn(hObject, eventdata, handles)

% hObject handle to popupmenu8 (see GCBO)

% eventdata reserved - to be defined in a future version of MATLAB

% handles empty - handles not created until after all CreateFcns called

% Hint: popupmenu controls usually have a white background on Windows.

% See ISPC and COMPUTER.

if ispc && isequal(get(hObject,'BackgroundColor'), get(0,'defaultUicontrolBackgroundColor'))

set(hObject,'BackgroundColor','white');

end

% --- Executes on button press in checkbox6.

function checkbox6_Callback(hObject, eventdata, handles)

% hObject handle to checkbox6 (see GCBO)

% eventdata reserved - to be defined in a future version of MATLAB

% handles structure with handles and user data (see GUIDATA)

% Hint: get(hObject,'Value') returns toggle state of checkbox6

% --- Executes on button press in checkbox7.

function checkbox7_Callback(hObject, eventdata, handles)

% hObject handle to checkbox7 (see GCBO)

% eventdata reserved - to be defined in a future version of MATLAB

% handles structure with handles and user data (see GUIDATA)

% Hint: get(hObject,'Value') returns toggle state of checkbox7
